# Clinical efficacy and safety of medicines procured through China’s national volume-based procurement policy: a systematic review of 81 studies

**DOI:** 10.3389/fpubh.2026.1820505

**Published:** 2026-10-15

**Authors:** Lele Zhang, Youjie Zhang

**Affiliations:** School of International Pharmaceutical Business, China Pharmaceutical University, Nanjing, Jiangsu, China

**Keywords:** China health policy, generic substitution, NVBP, risk of bias, systematic review, therapeutic equivalence, volume-based procurement

## Abstract

**Background:**

China’s National Volume-Based Procurement (NVBP) policy, launched in 2018, has sharply cut drug prices through competitive bidding among manufacturers of quality-consistent generics. Concerns persist that reductions sometimes exceeding 90% may compromise efficacy or safety, and this evidence has not been systematically synthesised.

**Objective:**

To evaluate the efficacy, safety and pharmacokinetics of NVBP-selected medicines.

**Methods:**

Six sources (PubMed, Web of Science, Cochrane CENTRAL, CNKI, Wanfang Data, VIP) were searched in two arms for reports published between January 2018 and January 2026; CENTRAL records could not be exported. Titles and abstracts were screened by one reviewer with a second checking; all 862 full-text reports were screened in duplicate. Risk of bias was assessed in eight domains, three specific to this policy setting, and certainty with GRADE. Results were synthesised without meta-analysis by effect direction within comparator strata. PROSPERO: CRD420261303351.

**Results:**

Eighty-one studies were included, 72 from the database searches and nine from citation searching; 72 were retrospective or before-and-after and 62 single-centre. Sixty-eight studies plus one comparison from a six-arm study formed the main analysis against the originator. There, 60 of 62 counted efficacy comparisons and 55 of 65 safety comparisons found no significant difference, and no efficacy comparison carries an assigned direction. Four safety comparisons favoured the originator (moxifloxacin tablets, olanzapine, paediatric ceftriaxone, atorvastatin) and three favoured the selected product (linezolid tablets, ceftriaxone, bortezomib); no direction could be assigned in three. Acquisition costs were 44% to more than 90% lower wherever reported. The median smallest detectable fall in response rate was 16.5 percentage points and the median smallest detectable relative risk for harm 2.67, reflecting the sample sizes available. Certainty was very low for efficacy, safety and pharmacokinetics, and low for economic outcomes.

**Conclusion:**

The available evidence does not show that NVBP-selected generics perform worse than their comparators, but its certainty is very low and the concordance of null findings cannot be read as demonstrated equivalence. Most studies reported no effect estimate, many were small and single-centre, and six reported statistics irreconcilable with their own data. Prospective, adequately powered studies reporting estimates with confidence intervals remain necessary.

**Systematic review registration:**

https://www.crd.york.ac.uk/PROSPERO/view/CRD420261303351; Identifier: CRD420261303351.

## 1 Introduction

China’s pharmaceutical expenditure has grown rapidly over the past two decades, placing increasing pressure on both the national healthcare budget and household out-of-pocket spending. In response, the State Council launched the National Volume-Based Procurement (NVBP) policy in November 2018, beginning with a pilot program known as the “4 + 7” trial in 11 cities (1). The policy requires manufacturers of generic drugs that have passed national bioequivalence consistency evaluations to compete on price for guaranteed procurement volumes from public hospitals. By the end of 2025, 11 rounds of NVBP had been completed, covering 491 products, with average price reductions exceeding 50% and in some cases surpassing 90% (2).

The NVBP policy has been widely recognized for its success in reducing drug prices and alleviating the financial burden on patients and the healthcare system. However, its rapid implementation has also generated significant concerns among clinicians, patients, and the public (2). The central question is whether generic medicines procured at dramatically reduced prices maintain the same quality, therapeutic efficacy, and safety as their originator counterparts or previously used branded generics. These concerns are particularly acute for narrow therapeutic index drugs (NTIDs) such as tacrolimus, olanzapine, and digoxin, where small variations in bioavailability could theoretically translate into clinically meaningful differences in patient outcomes.

Although China’s generic consistency evaluation program mandates bioequivalence (BE) testing as a prerequisite for NVBP participation, BE studies are typically conducted in healthy volunteers under controlled conditions, with standardized endpoints such as area under the curve (AUC) and maximum plasma concentration (Cmax) (3, 4). These pharmacokinetic parameters do not fully capture the real-world clinical performance of generic substitutions in diverse patient populations with comorbidities, polypharmacy, and variable adherence. As a result, post-marketing clinical evidence is essential to bridge the gap between regulatory approval and real-world therapeutic equivalence.

Beyond scattered single-center reports, a large coordinated real-world research program–led by the National Healthcare Security Administration together with tertiary-hospital pharmacy departments–has evaluated successive batches of NVBP-selected generics across multiple provinces and tens of thousands of patients (2, 5, 6). However, much of this evidence remains dispersed across single-drug reports, Chinese-language journals, and grey literature, and has not been brought together under a transparent, reproducible framework. To date, no study has systematically searched, critically appraised, and synthesized this body of evidence using internationally recognized methods–namely PRISMA-guided searching, design-specific risk-of-bias appraisal, and GRADE certainty rating. The absence of such a synthesis leaves clinicians without a reliable evidence base to guide prescribing decisions, policymakers without a clear picture of the policy’s clinical impact, and patients without reassurance about the medicines they are asked to use.

This systematic review addresses this gap by providing the first systematic identification, appraisal, and synthesis of all available original research evaluating the clinical efficacy, safety, and pharmacokinetic characteristics of medicines selected through China’s NVBP policy. Given that this is a recent and still-developing research field where the earliest included clinical study was published in 2020, we set out to synthesize the current evidence, map the methodological context, identify critical knowledge gaps, and establish a baseline against which future evidence accumulation can be measured.

## 2 Methods

### 2.1 Protocol and registration

The review was registered with PROSPERO on 9 February 2026 (CRD420261303351). The searches filed with the registration were executed on 7 February 2026; the searches reported in this review were re-executed between 9 August and 8 September 2026 and are documented in Supplementary Table S1. The record was amended during this revision and the current version is Version 3.1, published on 15 September 2026; all amendments made after registration are reported in full in Supplementary Table S5, item 24c. A separate protocol document was not prepared, and the registration record therefore serves as the protocol.

One feature of the registration should be stated plainly. The record was created through the register’s training route. PROSPERO designates training records as not for publication, so the record does not enter the register’s published series, is not returned by its search interface, and carries a TRAINING watermark. It can nonetheless be viewed directly at https://www.crd.york.ac.uk/PROSPERO/view/CRD420261303351, where the full version history and the text of each version are visible, and it is reproduced in full as Supplementary material 1. The search strategy filed with the registration is available at https://www.crd.york.ac.uk/PROSPEROFILES/b95dfd58d4898ba1fd91303d3caf348e.pdf. This systematic review was conducted and is reported in accordance with the PRISMA 2020 statement (7).

### 2.2 Eligibility criteria

Eligibility was defined using the PICOS framework. Studies were eligible if they evaluated patients prescribed a medicine selected through China’s NVBP policy in any completed round and any therapeutic area; if the medicine under investigation was an NVBP-selected generic that had passed the national consistency evaluation; if the comparator was one of the three types set out below; if at least one clinical efficacy, safety or pharmacokinetic outcome was reported; and if the report was original research of any design.

Eligibility of the medicine was judged against the official list of products procured in the first 11 rounds of national centralised procurement, comprising 491 products, taken from the results notices of each round. That list was checked in full against the official results during this revision. A report was excluded when the active ingredient did not appear on the list, or when the dosage form did not correspond to the procurement unit under which the ingredient was listed. Dosage-form equivalence was judged against the procurement unit used in the national list, such as “solid oral immediate-release form”, rather than against the pharmacopoeial dosage form; a comparison of a selected dispersible tablet with an originator capsule of the same ingredient and strength was therefore eligible, whereas a comparison across different procurement units was not. The round-three supply list, which places cefdinir capsules and cefdinir dispersible tablets under a single procurement item, supports this reading. Eligibility was determined by the substance of the medicine evaluated rather than by whether the source article used procurement terminology, so as to capture studies framed as conventional generic-versus-originator comparisons and to avoid a terminology-based selection bias.

The first round was procured in two stages of different geographical scope, and verification was adapted accordingly. The pilot of December 2018 applied to 11 cities and produced a single award table. The expansion of September 2019 applied to 25 provinces and autonomous regions; the municipalities that had taken part in the pilot were not part of it, nor were two provinces that had already adopted the pilot prices. Each medicine in the expansion had at most three winning suppliers, which chose the provinces they would serve, so the winning manufacturer differs between provinces and no consolidated national award table exists for that stage. Pilot cities continued to purchase under the pilot award until the pilot contracts were renewed. For first-round products, verification therefore required the source to name the manufacturer and the study site to be matched either to the pilot award table or to the award list of its own province. Where a province published no consolidated award list, manufacturer-level verification was not obtainable; this is stated for the studies concerned rather than resolved by inference.

A report was excluded on timing grounds when the recruitment window closed before the announcement of the relevant round, or when the overlap between the recruitment window and the post-announcement period was shorter than 1 month. Announcement dates were taken from the official results notices of each round. Studies whose recruitment window spanned the announcement were retained and were assessed for exposure misclassification in the risk-of-bias table.

Three comparator types were pre-specified: the originator product, including imported originator preparations; the same medicine at the same institution before the policy took effect; and a non-selected generic of the same ingredient and dosage form. These were analysed as separate strata. Studies in which the same patients were observed before and after substitution were analysed as a fourth, within-patient stratum. A comparison in which both products had won procurement, in different stages or rounds, was reported with the non-selected-generic stratum, identified as a within-policy substitution, and did not enter the originator-comparator synthesis. Where the official supply list shows that the originator product was itself among the winning suppliers of that round, the comparison is described as being between a selected generic and a selected originator and is identified as such; for first-round products this determination is province-specific. Comparisons against a non-selected generic were retained but analysed separately and were not combined with comparisons against the originator, because equivalence to another generic does not support inference of equivalence to the reference product. Studies in which the comparator arm could not be resolved into one of these types would have been classified as awaiting classification; none was.

Where a study compared more than two arms, or more than one medicine, only the arms or medicines that met the eligibility criteria were extracted, and each eligible pair was entered as a separate comparison. Arms that did not meet the criteria, such as a different active ingredient or a different regimen, were disregarded. Study-level and comparison-level counts are reported separately.

In this review, “quality” denotes real-world clinical quality performance, that is, whether consistency-evaluated NVBP-selected generics perform equivalently to their comparators in routine clinical practice, rather than the chemical or manufacturing quality that is the remit of China’s generic consistency-evaluation programme and of regulatory post-market sampling. The regulatory dimension of quality is summarised as contextual background in Section 4.2.

Exclusion criteria were as follows: bioequivalence studies conducted in healthy volunteers, whose population and outcomes do not meet the PICO; reports that were not original research, including conference abstracts, single case reports, commentary, narrative reviews, editorials and letters without original data; systematic reviews and meta-analyses, whose reference lists were nonetheless screened for eligible primary studies; studies reporting only drug utilisation or expenditure metrics without a clinical outcome; studies evaluating attitudes, preferences or prescribing intentions without objective clinical measurement; studies evaluating traditional Chinese medicine products, medical devices procured through volume-based procurement, or biologics and biosimilars outside the NVBP catalogue; reports that duplicated an already included study; reports whose full text could not be obtained; and reports whose earliest formal publication date, including online-first publication, fell outside the search window. One exception was made to the exclusion of reports whose full text could not be obtained: a multicentre randomised trial published in an indexed journal, whose abstract allowed every screening question to be answered and reported extractable outcome data, was retained, is identified as abstract-only wherever it is cited, and is removed in a sensitivity analysis (Supplementary Table S7). Reports were treated as duplicate publications of the same study when they drew on the same patient cohort, the same data source and an overlapping study period for the same active ingredient; where this applied, the report with the more complete sample was designated the main report and any companion report was listed rather than discarded. Reports from the same research group or the same hospital information system that evaluated different active ingredients were counted as separate studies, with the overlap in data source assessed in the risk-of-bias table and acknowledged in Section 4.1. No restriction on sample size was applied.

### 2.3 Information sources and search strategy

Six bibliographic sources were searched: CNKI, Wanfang Data, VIP, PubMed, the Web of Science Core Collection, and the Cochrane Central Register of Controlled Trials. Two search arms were applied at each source. The first arm combined procurement terminology, including the colloquial term jicai, with clinical outcome terms. The second arm was anchored on generic-versus-originator terminology without procurement terms, so that studies evaluating NVBP-selected products but not using procurement wording could be retrieved. The Chinese-language sources were searched on 9 August 2026 and the English-language sources on 8 September 2026. No language restriction was applied, and eligibility was restricted at screening to reports whose earliest formal publication date fell between 1 January 2018 and 31 January 2026. The complete strategy for each source and arm, with the date searched and the number of records retrieved, is reported in Supplementary Table S1. Reference lists of the included studies and of a broad review of the field (2) were additionally searched; reports identified only in this way entered the review through the separate identification route shown in Figure 1. Embase was listed as an information source in the registered protocol but could not be searched because institutional access was unavailable; this is reported as an amendment in Supplementary Table S5, item 24c, and as a limitation in Section 4.1.

**Figure 1:**
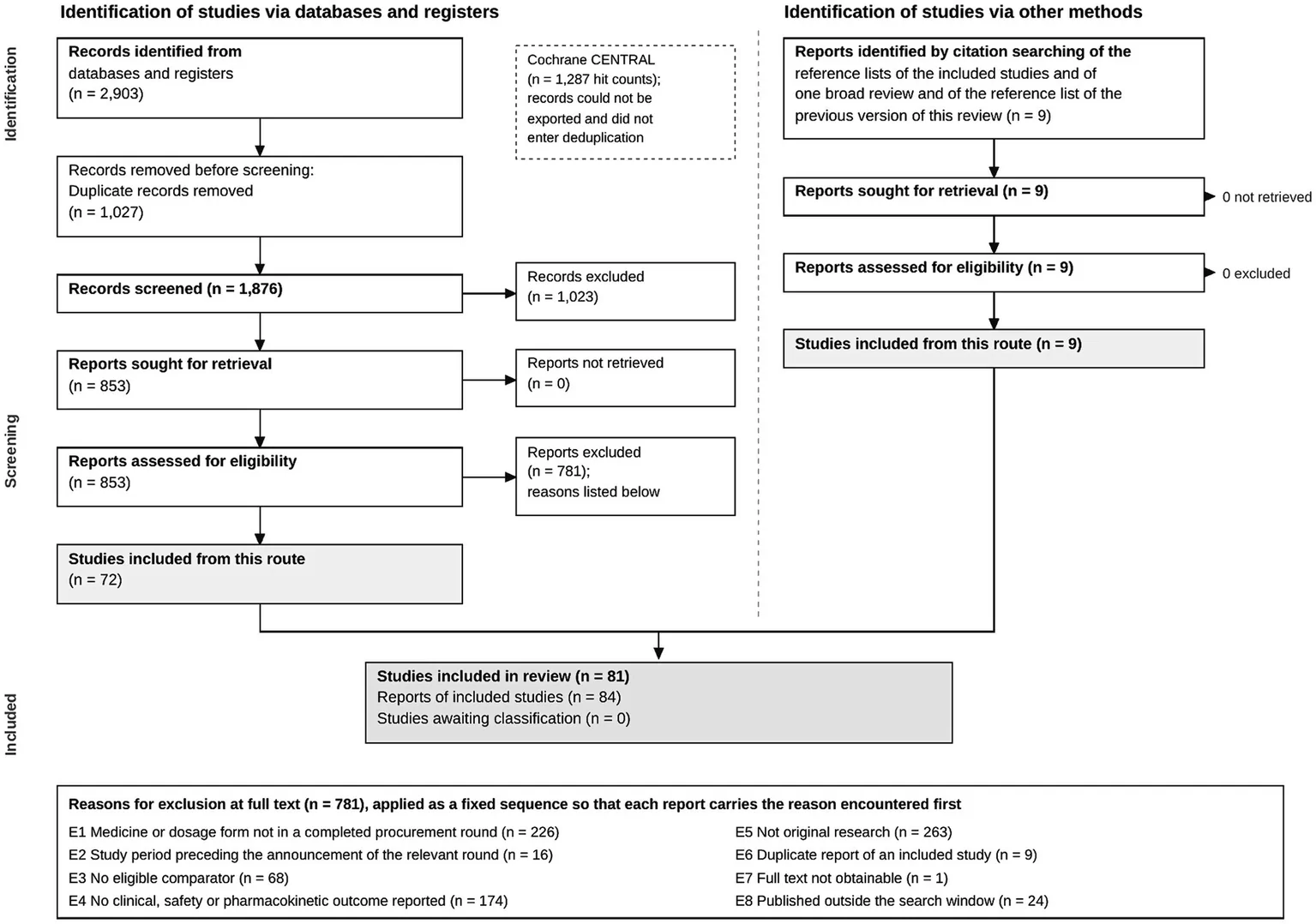
PRISMA 2020 flow diagram of study identification and selection. Bibliographic records could not be exported from Cochrane CENTRAL, so its hit count is reported for completeness only and those records did not enter deduplication or screening. Per-source record counts are given in Supplementary Table S1.

The review was carried out iteratively rather than as a single sequential pass. Screening, full-text retrieval, data extraction and risk-of-bias appraisal began on the records returned by the Chinese-language sources and proceeded alongside them; the English-language sources were searched subsequently, and the records they returned were processed through the same procedures and merged with the earlier set before eligibility was finalised. The latest date on which any source was searched, and therefore the currency date of this review, is 8 September 2026.

### 2.4 Study selection

Records retrieved from all sources were imported into reference-management software and deduplicated by matching digital object identifiers and standardised titles. Titles and abstracts of the deduplicated records were screened by one reviewer and checked by a second, as specified in the registered protocol, with disagreements resolved by discussion; records classified as potentially eligible or uncertain by either reviewer proceeded to full-text assessment. The reliability of this stage was assessed *post hoc*: a random sample of 500 of the 1,876 deduplicated records was drawn with a fixed seed and re-screened independently and in duplicate by two reviewers blind to the original decision. Raw agreement was 89.2% (446 of 500) and Cohen’s kappa was 0.78 (95% CI 0.73 to 0.84).

All 862 reports that reached full-text assessment were then screened in duplicate by two reviewers working independently, against a fixed eight-question sequence applied in a set order, so that each excluded report carries the reason encountered first. The eight questions, in order, concern whether the medicine or dosage form belongs to a completed national procurement round; whether the study period precedes the announcement of the relevant round; whether an eligible comparator is present; whether a clinical, safety or pharmacokinetic outcome is reported; whether the report is original research; whether it duplicates an already included study; whether the full text is obtainable; and whether the publication date falls within the search window. Disagreements arose for 18 reports and were resolved by discussion, with the reason for every exclusion recorded (Supplementary Table S6). Eligibility of every included study was confirmed against the full text, with one exception identified in Section 3.9. As a check on the excluded records, 400 of the 781 excluded reports, just over half, were drawn at random and independently re-assessed against the full text by two reviewers who were blind to the recorded decision. Their judgements agreed with the recorded decision for 393 reports (98.25%); the seven discrepant reports were discussed and the recorded decision was upheld in every case.

### 2.5 Data extraction

Data were extracted using a standardised form that captured study identification, design, setting, data source and collection period, procurement round and specific medicines evaluated, sample size and participant characteristics, comparator details and manufacturer, outcomes with effect estimates and confidence intervals, adverse event data, statistical methods, funding and declared conflicts of interest. Data were extracted by one reviewer and independently checked against the source article by a second reviewer; discrepancies were resolved by returning to the full text and agreeing a single value. Corresponding authors were contacted by email to request data missing from the reports, such as an effect estimate or the counts from which one could be derived, a denominator for a reported proportion, or a statistic that could not be reconciled with the values printed in the same report. No additional data were obtained by this route, and every value reported here is taken from the published reports as they stand. Where a report gave a result only in a figure, or only as a statement of significance, the value was recorded as reported and no estimate was derived or imputed. Where a source reported group sizes together with outcome percentages but did not test an eligible comparison, frequencies were reconstructed from the reported values and chi-square tests were applied by the review authors; such analyses are identified as *post hoc* in the Results and in Supplementary Table S3, and the rounding uncertainty introduced by reconstruction is stated. Counts were derived from reported percentages only where they corresponded exactly to those percentages at the reported group size; no denominator was inferred from a percentage.

### 2.6 Risk of bias assessment

Risk of bias was assessed in eight domains. Five follow the domains of ROBINS-I and were applied through the instrument appropriate to each design: the Cochrane Risk of Bias 2 tool for the randomised trial; the Newcastle–Ottawa Scale for retrospective cohort studies; a modified ROBINS-I for before-and-after studies; and the Institute of Health Economics checklist for pharmacovigilance case series. Three further domains are specific to this policy setting and are not covered by any existing instrument. Exposure misclassification arises where the recruitment window precedes the announcement of the relevant round, so that patients recorded in the selected-product arm during that period cannot have received a product holding selected status. Comparator identifiability arises where the comparator product is not named at manufacturer level, so that the contrast cannot be characterised. Independence of data sources arises where several included studies originate from one research group or multicentre platform. Assessments were made by one reviewer and checked by a second, with disagreements resolved by discussion. Detailed results are provided in Supplementary Table S2.

### 2.7 Evidence synthesis

Because of substantial clinical and methodological heterogeneity across the included studies, spanning multiple therapeutic areas, more than 40 distinct medicines, six study designs and heterogeneous comparator frameworks, a quantitative meta-analysis was neither feasible nor appropriate, since pooling effect estimates across unrelated drug classes would generate spurious precision and could mask clinically important between-class differences. We therefore conducted a structured synthesis without meta-analysis (SWiM), following the SWiM reporting guideline (8).

Studies were first allocated to comparator strata as defined in Section 2.2. The originator-comparator stratum constitutes the main analysis; the pre-policy, non-selected-generic and within-patient strata are reported separately in Section 3.7 and are not pooled with it. Within the main stratum, studies were allocated to pre-specified outcome domains (therapeutic efficacy, comparative safety, pharmacokinetics and economic outcomes) and, within each domain, stratified by therapeutic area and drug class; no quantitative pooling was performed across drug classes.

Each study contributes at most one entry to each outcome domain: where a study compared several medicines or several arms, its comparisons within one domain were combined into a single entry, and a study whose only comparative endpoint was serum concentration or prescribed dose was entered in the pharmacokinetic domain and not in the efficacy domain. For each study and outcome domain, the direction of effect relative to the comparator was recorded as favouring the NVBP-selected generic, favouring the comparator, or no statistically significant difference, and these directions were summarised in effect-direction tables organised by outcome domain and therapeutic area. The standardised metric for vote-counting was the direction of effect, adopted because the reported effect measures were not commensurable across studies. The direction for each domain follows the primary outcome specified by the study; where none was specified, it follows the outcome on which the study’s own conclusion rests. Secondary outcomes, surrogate markers and utilisation measures such as dose, persistence, switching and cost are recorded in Supplementary Table S3 but do not determine direction. In the safety domain, a statistically significant difference in any pre-specified safety outcome determines the direction; where significant differences within one cohort point in opposite directions, no direction is assigned. Each study is counted once in each domain to which it contributes, and multiple reports of one study are counted once. Classification by statistical significance does not reflect the magnitude or the clinical relevance of an effect, and a non-significant result in a small study cannot be read as evidence of equivalence; these limitations are carried into the interpretation of the results and into the certainty ratings, and are stated in Section 4.1. The same information is displayed graphically in a harvest plot, in which each study is positioned by direction of effect and coloured by its overall risk-of-bias rating.

We then performed a structured cross-study appraisal, examining how methodological differences related to the observed findings and explicitly identifying and seeking to explain divergent results, including any less-favourable efficacy or safety signals reported for individual generics. Eleven sensitivity analyses were conducted. The first two are those specified in the original submission and are reported first. The original specified restriction to studies at low or moderate overall risk of bias; because risk of bias is now assessed in eight domains rather than as a single overall rating, that analysis is implemented here as the exclusion of any study rated high in any domain. The second, restriction to multicentre studies, is unchanged. The remaining nine were added during this revision and are identified as not pre-specified: restriction to the originator-comparator stratum; exclusion of studies at high risk of exposure misclassification; exclusion of studies whose arms are drawn from non-overlapping calendar windows; exclusion of studies without matching or adequate adjustment; exclusion of the single study extracted from an abstract only; exclusion of studies with incomplete or irreconcilable outcome reporting; retention of a single study per research group or platform; exclusion of comparisons tested only by the review authors; and assignment of a direction wherever any outcome of a study’s principal analysis differed significantly, rather than by the primary outcome alone. Results are reported in Supplementary Table S7.

## 3 Results

Effect estimates were sought for every comparison and are reported in Supplementary Table S3. An effect estimate is available for 39 of the 81 included studies: four were reported by the source with a confidence interval, and for 37 the reported event counts or summary statistics allowed the review team to calculate one, two studies contributing both. For the remaining 42 studies the source reported only a test of significance or a statement of no difference, and no estimate is calculable. The standardised metric for the synthesis is therefore the direction of effect rather than a pooled estimate. The certainty of the body of evidence for each outcome domain was rated using the GRADE framework (Supplementary Table S4), considering risk of bias, inconsistency, indirectness, imprecision and publication bias, and an overall strength-of-evidence statement was derived for each domain.

### 3.1 Study selection

The database searches retrieved 2,903 bibliographic records across the two arms, of which 1,027 were duplicates removed before screening, leaving 1,876 unique records. A further 1,287 records were identified in Cochrane CENTRAL; bibliographic records for these could not be exported and they did not enter deduplication. Screening of titles and abstracts excluded 1,023 records, and the remaining 853 reports were sought for retrieval and assessed in full text. Citation searching of the reference lists of the included studies and of a broad review of the field (2) identified a further nine reports that the database searches did not independently retrieve; these entered the review through the separate identification route shown in Figure 1 and were assessed in full text alongside the others.

Of the 862 reports assessed in full text, 781 were excluded. Reasons were: the medicine or dosage form did not belong to a completed national procurement round (*n* = 226); the study period preceded the announcement of the relevant round (*n* = 16); no eligible comparator (*n* = 68); no clinical, safety or pharmacokinetic outcome reported (*n* = 174); not original research (*n* = 263); duplicate report of an included study (*n* = 9); full text not obtainable (*n* = 1); and publication date outside the search window (*n* = 24). Eighty-one studies met all eligibility criteria, 72 of them identified by the database searches and nine by citation searching. No report remained awaiting classification.

All 862 reports were screened in duplicate against the fixed eight-question sequence; disagreements arose for 18 and were resolved by discussion, and the reason for every exclusion was recorded (Supplementary Table S6). Eligibility of every included study was confirmed against the full text, with one exception identified in Section 3.9. Of the excluded records, 400 were drawn at random and independently re-assessed against the full text by two reviewers; their judgements agreed with the recorded decision in 393 of 400 cases, and the seven discrepancies were resolved by discussion without altering any recorded decision. The reliability of title and abstract screening was assessed *post hoc* in a random sample of 500 records, with raw agreement of 89.2% and a Cohen’s kappa of 0.78 (95% CI 0.73 to 0.84).

Three operating rules governed the sequence and are stated here because they determine the counts above. First, eligibility of the medicine was decided against the official list of products procured in rounds 1–11, comprising 491 products; that list was checked in full during this revision, and no exclusion under the first question rested on an omission from it. Second, a study was excluded on timing grounds only when its recruitment window closed before the announcement of the relevant round, or when the overlap with the post-announcement period was shorter than 1 month; this rule excluded one population-based cohort whose observation window extended only 14 days beyond the first-round announcement. Third, dosage-form equivalence was judged against the procurement unit used in the national list rather than against the pharmacopoeial dosage form, so that a comparison of a selected dispersible tablet with an originator capsule of the same molecule and strength remained eligible.

Three reports were excluded because the comparator described as procured had in fact been supplied through provincial or inter-provincial alliance procurement, which in two cases preceded the national programme by more than a year. The study selection process is shown in Figure 1.

### 3.2 Characteristics of included studies

Characteristics of the 81 included studies are given in Table 1 (5, 9–88). One was a multicentre randomised controlled trial, seven allocated patients prospectively by randomisation, random number table, admission sequence or visit sequence, one was a prospective observational cohort, and the remaining 72 were retrospective or before-and-after in design, including eight before-and-after studies and seven master’s theses. Sixty-two were conducted at a single centre and 19 at more than one; the largest involved 19 institutions and the largest by sample size screened 359,159 patients before matching. Propensity score matching or inverse probability weighting was applied in 33 studies and randomisation or prospective allocation in eight; one applied multivariable regression adjustment without matching. In the remaining 39 no formal control of confounding was applied, although in most of these the arms did not differ materially at baseline; 12 studies in total were graded at high risk in the confounding domain (Section 3.9).

**Table 1:** Characteristics of the 81 included studies.

Ref.	Study	Design	Setting	Study period	Medicine (round)	Stratum	Selected vs comparator (n)	Outcomes assessed	Key findings	Notes on risk of bias
(9)	Cai 2024 [75]	Retrospective cohort, PSM	Single centre, Fuzhou	2020-10-20 to 2022-10-20	Levofloxacin sodium chloride injection (round 5)	I	117 vs. 117	Day-7 response, WBC, NEUT%, CRP, PCT, duration, hepatic/renal function	No significant difference in any outcome	First 8 months precede announcement
(10)	Chen 2021 [198]	Prospective allocation by visit sequence	Single centre, Kunming	2020-01 to 2021-01	Amlodipine besylate (round 1)	I	50 vs. 50	BP, response rate, BP variability, heart rate, ADR	Overall response 44/50 vs. 46/50; adverse reactions 3/50 vs. 5/50, *p* = 0.461; control of morning systolic blood-pressure variability better with the imported originator (*p* < 0.05)	Master’s thesis; allocated by attendance order. Morning blood-pressure variability is a measure of blood-pressure control, not a safety outcome. Manufacturer of the selected arm recorded as probable, not verified
(11)	Chen 2023 [144]	Multi-medicine retrospective analysis with active surveillance	Single centre, Guangzhou	2020 to 2022	20 medicines in 10 groups (multiple rounds)	I	Not stated	ADR incidence	ADR rates comparable for most of the twenty medicines; lower with the selected product for a minority, which the source does not identify	Reported in aggregate across twenty medicines with no denominator by medicine, so no direction is assigned. Medicines span several rounds, so exposure misclassification is indeterminate
(12)	Chen 2023 [751]	Retrospective cohort, PSM	Single centre, Beijing	2020-11 to 2021-11	Capecitabine (round 3)	I	118 vs. 118 after PSM	ORR, DCR, adverse reactions	No significant difference in any outcome	Same centre and team as Yang 2021
(13)	Cheng 2024 [823]	Prospective randomised allocation	Single centre, Chongqing	2020-08 to 2021-07	Budesonide suspension for inhalation (round 5)	I	46 vs. 48	Time to resolution of dyspnoea, wheeze, cough; FEV1, PEF; control rate	Clinical control grading comparable (*p* = 0.326); dyspnoea and rhonchi resolved faster and FEV1 was higher with the selected product (all *p* < 0.05)	Randomised by random-number table. The Discussion section of the source contradicts its own tables
(14)	Cheng 2026 [1415]	Multicentre randomised controlled trial, three arms	Multicentre	Not stated	Amoxicillin as regimen component (rounds 2 and 3)	III	450 randomised	*H. pylori* eradication (PP and ITT), adverse events, adherence, cost	Eradication 90.7% vs. 92.1% (PP) and 84.7% vs. 85.3% (ITT); cost per course fell 44%	Extracted from abstract; arm-specific safety data unavailable
(15)	Feng 2024 [218]	Two-centre retrospective cohort, PSM	Two centres	2021-11 to 2022-03	Levetiracetam concentrate for injection (round 3)	I	18 vs. 17 (treatment); 76 vs. 76 (prophylaxis)	Response rate, ADR, daily cost	No significant difference in either subgroup; no ADR in either arm	The originator product was itself a round-three winner; treatment arm and centre completely collinear
(16)	Gao 2026 [97]	Retrospective cohort, PSM	Single centre, Wuhan	Selected arm 2023-01 to 2024-12; comparator 2020-01 to 2022-12	Meropenem injection (round 7)	I	116 vs. 116 after PSM	Clinical and 72-h response, AEs, cost	Response 73.3% vs. 70.7% (*p* = 0.661); AEs comparable; costs markedly lower	Non-overlapping periods
(17)	Ge 2024 [840]	Before-and-after retrospective analysis	Single centre, Shanghai	Pre 2021-12 to 2022-05; post 2022-12 to 2023–05	Tigecycline for injection (round 7)	II and III	76 vs. 86	Clinical response, ADR reports, cost	Response 89.5% vs. 88.4%; ADR comparable; unit price fell from 430 to 28.2 CNY	Non-overlapping periods
(18)	Guo 2023 [37]	Retrospective mirror-image cohort, PSM	Single centre, Beijing	First year after policy	Sertraline (round 3)	I	5,973 vs. 7,686; 1,758 vs. 1,758 after PSM	Serum concentration, prescribed daily dose, prolactin, liver/renal function, lipids	Dose-adjusted serum concentration lower with generic (*p* = 0.009); laboratory abnormality rates comparable	No clinical efficacy endpoint; same platform as Jia 2022
(19)	Han 2021 [730]	Registry cohort (NCT04184583)	Single centre, Beijing	12-month follow-up	Clopidogrel (round 1)	I	1,657 vs. 1,753	12-month MACCE, BARC ≥2 bleeding, expenditure	MACCE 5.07% vs. 4.91% (*p* = 0.83); bleeding comparable	Single-endpoint figures transposed in source
(20)	He 2023 [228]	Retrospective cohort, PSM	Single centre, Nanchong	2020-01 to 2022-04	Linezolid glucose injection (round 5)	I	30 vs. 30 after PSM	30-day and in-hospital mortality, bacteraemia duration, length of stay, ADR	Mortality and ADR comparable; length of stay longer in exposed group	Small sample; first 18 months precede announcement
(21)	Hong 2025 [145]	Thesis; before-and-after, four medicines	Single centre, Mudanjiang	2019-12-10 to 2022-12-31	Atorvastatin; capecitabine; pregabalin; budesonide	II	133/150; 50/76; 56/75; 241/139	LDL-C attainment, ORR/DCR, VAS, FEV1, ADR	No significant difference for any of the four medicines	Four medicines; entered once per outcome domain (Section 2.7)
(22)	Huang 2022 [606]	Registry-based cohort (switch vs. no switch)	Single centre, Zhengzhou	2019-01-01 to 2021-05-18	Entecavir (round 1)	III	156 vs. 104	Complete virological response, ALT normalisation, HBeAg clearance and seroconversion, ADR	Response 96.8% vs. 97.1% at 24 weeks and 100% in both arms at 48 weeks; no adverse events in either arm	Ceiling effect; zero events
(23)	Jia 2023 [107]	Before-and-after cohort, PSM	Single centre, Beijing	23 Mar to 23 Sep 2019, with 23 Mar to 23 Sep 2018 as the preceding-year reference	Paroxetine tablets (round 1)	I	1,168 vs. 1,168 after matching	Dose, serum concentration, hepatic/renal, lipid, glucose, prolactin	No significant difference in any outcome; drug cost fell	Selected product verified to manufacturer level (Zhejiang Huahai, pilot winner); the efficacy and safety comparison is between arms within the later period, while the before-and-after contrast is used only for switching and cost
(24)	Jiang 2022 [251]	Prospective allocation by admission sequence	Single centre, Changzhou	2020-01 to 2022-01	Esomeprazole sodium for injection (round 5)	I	43 vs. 43	Response rate, haematemesis and melaena counts, ADR	Treatment response 39/43 vs. 41/43, *p* = 0.397; haematemesis and melaena episodes fewer with the selected product at all ten time points (*p* = 0.037 to <0.001); adverse reactions 4/43 vs. 6/43	Allocated by admission order, no matching. The source states the arms were comparable at baseline but reports mean ages of 60.50 and 47.34 years, which differ at *t* = 14.0
(25)	Li 2024 [753]	Retrospective pharmacoeconomic comparison	Single centre paediatrics, Urumqi	2020-01 to 2022-08	Ceftriaxone sodium for injection (round 5)	I	140 vs. 152	Clinical response, time to defervescence, ADR, cost	Clinical response 122/140 vs. 136/152, *p* = 0.753; defervescence slower and adverse reactions more frequent with the selected product (8/140 vs. 2/152)	The adverse-reaction comparison is borderline: chi-square *p* = 0.039 but Fisher *p* = 0.052. The cost table of the source is internally inconsistent and its economic figures are not cited
(26)	Li 2023b [X9]	Retrospective cohort, PSM	Three centres, Shanghai, Zhengzhou and Wuhu	2020-01-01 to 2021-12-31	Glimepiride tablets (round 2)	I	103 vs. 103 after PSM (229 vs. 113 before)	HbA1c change (primary), FPG change and HbA1c attainment, hypoglycaemia, transaminases, eGFR	No difference in any efficacy or safety outcome	Recruitment opens 21 days before the announcement of round two. Originator named (Amaryl); selected arm pools three manufacturers. Entered by citation searching
(27)	Li 2024 [X5]	Retrospective cohort	Single centre, Nanjing	Pre 2020-01 to 2020-11; post 2020-12 to 2021-11	Ticagrelor tablets (round 3)	I	88 vs. 93	ADP inhibition and attainment, six-month ischaemic and bleeding events, GOS, mRS	No significant difference in any outcome	Selected product matched directly to the round-three list; arms drawn from non-overlapping periods
(28)	Li 2025 [102]	Retrospective cohort	Xiamen Third Hospital (reported as an unnamed tertiary hospital)	2022-01 to 2023-12	Olanzapine tablets (round 1)	I	288 vs. 174	Depression rating scale score, daily dose, lipid and glucose abnormality, price	Depression rating scale score after treatment comparable (20.57 vs. 19.73, source reports *p* = 0.952); glucose abnormality 29/288 vs. 8/174, *p* = 0.036; lipid abnormality comparable	No matching and baseline age distribution differed (*p* = 0.019). The test statistics reported by this source are not reproducible from its own summary statistics and are mutually inconsistent throughout, and its abstract and Results section disagree on whether daily dose differed; outcome reporting is at high risk of bias
(29)	Li 2025 [164]	Retrospective cohort, PSM	Single centre, Huangshi	2021-01 to 2024-06	Meropenem injection (round 7)	I	193 vs. 193 after PSM	Cure rate, ADR, cost	Cure 86.0% vs. 91.2% and ADR 7.3% vs. 5.7%, both non-significant	First 18 months precede announcement
(30)	Li 2025 [83]	Multicentre retrospective cohort, PSM	Four centres	2021-09 to 2023-08	Saxagliptin (round 5)	I	178 vs. 164; 141 vs. 141 after PSM	HbA1c and FPG change, target attainment, hypoglycaemia, hepatic/renal function	No significant difference in any outcome	—
(31)	Li 2025 [96]	Retrospective cohort, PSM	Single centre, Huangshi	2020-01 to 2024-06	Caspofungin acetate injection (round 9)	I	117 vs. 117 after PSM	Cure rate, ADR, cost	Cure 69.4% vs. 72.7% and ADR 14.5% vs. 12.0%, both non-significant	Nearly four years precede announcement
(32)	Li 2026 [82]	Retrospective cohort (DRG group ES13)	Single centre, Linyi	2019-01 to 2023-12	Voriconazole tablets (round 4)	I	90 vs. 78	Response rate, length of stay, unplanned readmission, ALT elevation	No significant difference; cost-effectiveness favoured generic	First two years precede announcement
(33)	Liang 2022 [236]	Retrospective cohort, 1:1 PSM	Two centres, Guangzhou	2018-01-01 to 2021-03-31	Atorvastatin (round 1)	I	363 vs. 302	Stroke/TIA recurrence, NIHSS, mRS, new coronary disease, AST/ALT, composite AE	No significant difference in any outcome	First 11 months precede announcement
(34)	Liang 2022 [244]	Single-arm before-and-after switch	Single centre, Guangzhou	2019-05 to 2020-12	Levetiracetam (round 1)	IV	154	Seizure control, frequency, ADR	Control improved from 77.3 to 83.8% after switch (*p* < 0.05); adverse reactions comparable	No parallel control group
(35)	Liang 2024 [718]	Prospective cohort, six parallel arms; thesis	Single centre, Zhanjiang	2022-01 to 2024-01	Tenofovir disoproxil fumarate; tenofovir alafenamide (rounds 1 and 7)	I and III	107 vs. 81; 101 vs. 71	48-week sustained virological response, HBeAg clearance, ALT normalisation, renal function, lipids	48-week sustained virological response 48.5% vs. 66.7% for the disoproxil pair, not tested by the source and significant on *post hoc* reconstruction; renal function did not differ; lipids higher in the selected alafenamide arm	The source tested every arm against tenofovir amibufenamide only. Arm sizes used in the reconstruction are consistent with all reported percentages but were not read directly from the source
(36)	Liu 2023 [853]	Retrospective cohort, PSM	Single centre, Shijiazhuang	2020-10 to 2021-10	Moxifloxacin sodium chloride injection (round 3)	I	61 vs. 111 after PSM	72-h and discharge response, temperature, CRP, PCT, ADR	No significant difference in any outcome	—
(37)	Liu 2024 [618]	Multicentre retrospective cohort, PSM	19 centres, Kunming	2020-01 to 2022-09	Cefazolin, cefuroxime and ceftazidime injections (rounds 3 and 5)	III	394/394; 472/472; 504/590	Clinical and 72-h response, bacterial clearance, infection markers, ADR	Clinical response comparable for all three medicines; CRP recovery more frequent with selected ceftazidime; platelet reduction with selected cefazolin and eosinophil elevation with selected ceftazidime more frequent	Non-selected-generic stratum. Laboratory denominators differ by index and are not reported; the cefazolin AST entry is incompatible with its group size
(38)	Liu 2025 [264]	Retrospective cohort, three parallel arms	Single centre, Tianjin	2019-09 to 2024-09	Tenofovir alafenamide (round 7)	I	57 vs. 58	HBV-DNA conversion, HBeAg and HBsAg clearance, ALT normalisation, hepatotoxicity, nephrotoxicity, lipids	No significant difference in any efficacy or safety endpoint; triglycerides rose in the selected arm while falling in the comparator	Sample size powered for a different contrast; approval numbers apparently transposed
(39)	Luo 2022 [73]	Before-and-after cohort	Single centre psychiatric department	Control 2018-03 to 2019-02; intervention 2019-03 to 2020-02	Six psychiatric medicines (round 1)	II	1,000 vs. 1,000	Composite efficacy score, ADR score, cost-effectiveness	Composite efficacy and ADR scores comparable; annual drug spend fell 32.8%	Composite scores rather than per-patient events; non-overlapping periods
(40)	Luo 2023 [739]	Retrospective cohort, PSM	Single centre, Beijing	2021-01-01 to 2021-09-30	Metformin (round 3)	I	200 vs. 200 for direct indices; 4,077 pairs for indirect indices	HbA1c and FPG change, attainment, ALT, AST, creatinine	HbA1c and fasting glucose change and HbA1c attainment (163/200 vs. 155/200) comparable; liver and renal abnormality comparable; originator arm had higher persistence and the selected arm higher switching (*p* < 0.001)	Safety indices use three different denominators (188/192, 159/162, 199/198). Arm order verified against the source
(41)	Luo 2023b [X8]	Retrospective cohort, PSM	Single centre, Beijing	Originator 2019-01-01 to 2019-12-30; selected 2020-10-01 to 2021-09-30	Glimepiride tablets (round 2)	I	81 vs. 81 after PSM for direct indices; 1,350 vs. 1,350 for indirect indices	HbA1c and FPG change, HbA1c attainment, AST, ALT, creatinine; persistence, dose escalation, cost	HbA1c and FPG change comparable; HbA1c attainment 50/81 vs. 34/81 (*p* = 0.018); liver and renal abnormality comparable	Arms drawn from non-overlapping calendar windows. Abstract and Results give opposite directions for persistence. Safety indices use three different denominators. Entered by citation searching
(42)	Miao 2024 [143]	Retrospective cohort	Single centre	2021-07 to 2023-06	Ticagrelor tablets (round 3)	I	156 vs. 264	12-month NACE, MACCE, event-free survival, bleeding	No significant difference in any outcome	—
(43)	Pang 2023 [215]	Retrospective cohort	Single centre outpatient, Lianyungang	2021-01 to 2021-12	Moxifloxacin hydrochloride tablets (round 2)	I	1,121 vs. 1,058	Response rate, time to resolution, duration, re-attendance, ADR	Longer time to resolution and higher rates of total, allergic, gastrointestinal and glucose-related adverse reactions with selected product (*p* < 0.05)	The originator product was itself a round-two winner; reports differences unfavourable to the selected product
(44)	Peng 2023 [X1]	Retrospective comparison	Single centre, Shanghai	2020-04 to 2021-03	Atorvastatin calcium tablets (round 1)	I	36 vs. 37	LDL-C attainment at 4-6 weeks, lipid change, adverse events	Attainment 50.0% vs. 43.2%, non-significant; adverse events comparable	Selected product verified to manufacturer level from the source (Lepu Pharmaceutical Technology); Shanghai was a pilot city outside the expansion alliance and took the product through renewal of the pilot contract on 30 March 2020, immediately before the study window; small sample, no matching, sex distribution differed at baseline (*p* = 0.019); laboratory endpoints only
(45)	Qian 2024 [104]	Retrospective cohort	Single centre	2022-01 to 2023-06	Omeprazole (round 3)	I	158 vs. 158	*H. pylori* eradication, ADR, cost	Eradication 88.6% vs. 93.0%, non-significant; ADR comparable	—
(46)	Si 2024 [X6]	Multicentre retrospective cohort, 1:1 PSM	Three centres, Beijing	2014-01 to 2022-02	Febuxostat tablets (round 3)	I	57 vs. 57 after PSM	12-week urate target attainment, urate reduction, ADR, cost	No significant difference in attainment or reduction; daily cost markedly lower	Six years and eight months precede announcement
(47)	Song 2023 [240]	Retrospective real-world study	Single centre, Xi an	2019-04 to 2020-05	Levetiracetam (round 1)	I	125 vs. 475	Serum concentration, response rate overall and by co-medication	No significant difference in concentration or response	Unmatched, markedly unequal arms
(48)	Song 2024 [89]	Multicentre retrospective cohort, PSM	17 centres, Kunming	2020-01 to 2022-09	Ceftriaxone injection (round 5)	I	546 vs. 1,275 before matching; 510 vs. 510 after matching	Clinical and 72-h response, bacterial clearance, infection markers, AEs	Efficacy comparable throughout; neutrophil reduction less frequent with the selected product (10/510 vs. 28/510, *p* = 0.003 on recalculation); the AST and creatinine differences reported as significant are not supported by the source’s own counts	Adverse-event denominators are pre-matching (546 and 1,275); laboratory denominators are the 510 matched pairs. *p* values throughout the safety table are not reproducible from the printed counts
(49)	Sun 2025 [72]	Retrospective cohort, PSM	Single centre, Fuzhou	2020-11 to 2022-11	Cefuroxime injection (round 5)	I	1,042 vs. 1,520 before matching; 1:1 matched within each department	Prophylactic efficacy, ADR, cost	No significant difference; cost lower with selected product	Selected product verified to manufacturer level (Zhejiang Huidisen, round-five winner); recruitment opened 8 months before the round was announced
(50)	Wang 2023 [142]	Multicentre retrospective cohort, 1:3 PSM	Three centres	2019-01-01 to 2021-12-31	Tegafur (round 2)	I	695 vs. 63	Tumour markers, haemoglobin, attainment rates, ADR, discontinuation	No significant difference in any outcome	Both generic manufacturers matched directly to the round-two list; Xuanwu platform
(51)	Wang 2023 [183]	Multicentre retrospective cohort, PSM	Three centres	2019-01-01 to 2022-03-31	Azacitidine injection (round 3)	I	93 vs. 53	Two-cycle remission, myelosuppression, infection, GI, hepatic, fever, fatigue	No significant difference in any outcome	Manufacturers anonymised; round-three winners were Sichuan Huiyu and Chia Tai Tianqing only; Xuanwu platform
(52)	Wang 2023 [186]	Retrospective cohort, PSM	Single centre, Beijing	2020-01-01 to 2021-12-31	Cefdinir (round 3)	I	1,268 vs. 1,268 after PSM	Daily dose, duration, use intensity, hepatic/renal indices, ADR	No between-group efficacy comparison performed; laboratory indices and ADR comparable	Capsule and dispersible tablet are listed under one procurement item in the official list; no between-group efficacy comparison performed; Xuanwu platform
(53)	Wang 2023 [720]	Retrospective cohort with regression adjustment	Single centre, Linyi	2018-01-01 to 2021-12-31	Clopidogrel (round 1)	I	705 vs. 915	All-cause and cardiac mortality, MACCE, stroke, cost	No significant difference in any endpoint	Baseline imbalance in five variables; no matching
(54)	Wang 2023 [X3]	Multicentre retrospective cohort, PSM	Multicentre	2020-05-01 to 2021-12-31	Linezolid tablets (round 3)	I	60 vs. 35 after 1:3 PSM (95 vs. 59 before)	Total use, duration, clinical response, hepatic injury, cytopenia, GI, rash, diarrhoea	Clinical response 48/60 vs. 32/35, *p* > 0.05; hepatic injury 0/60 vs. 3/35, *p* < 0.05; daily dose higher with the selected product in respiratory and other-site infection	Pre-matching arms differed in sex, insurance type, comorbidity count and treating department. Originator was itself a winning supplier of round three
(55)	Wang 2025 [119]	Retrospective cohort	Single centre, Beijing	2022-06 to 2023-06	Empagliflozin (round 4)	I	197 vs. 209	FPG, HbA1c, NT-proBNP, LVEF, GFR, creatinine clearance	Fasting glucose, eGFR, NT-proBNP and LVEF comparable; the index printed as GREA differed (*p* = 0.002) but the source defines it inconsistently; HbA1c *p* values are not consistent with the source’s own test statistics	No matching or adjustment; comorbidity and concomitant heart-failure therapy differed between arms
(56)	Wang 2025 [200]	Retrospective cohort, IPTW	Single centre, Kunming	2019-07 to 2022-06	Rivaroxaban (round 5)	I	529	Stroke, systemic embolism, readmission, bleeding, all-cause death (IPTW, Cox)	No significant difference in any endpoint: systemic embolism HR 0.280 (0.029–2.723), stroke HR 1.830 (0.552–6.070), all-cause death HR 1.183 (0.491–2.855), total bleeding HR 0.921 (0.531-1.596); subgroup analyses concordant	First two years precede announcement
(57)	Wang 2024 [223]	Retrospective cohort	Single centre, Xuyi	2022-01 to 2023-12	Atorvastatin (round 1)	I	263 vs. 255	TC, TG, LDL-C, HDL-C, AST, ALT, CK, ADR	Lipid indices comparable at 12 weeks; creatine kinase higher with the domestic product (*p* < 0.05); adverse drug reactions 37/263 vs. 25/255, *p* = 0.135	No matching; baseline body mass index (*p* = 0.010) and hypertension prevalence (*p* = 0.009) differed
(58)	Wang 2025 [154]	Retrospective cohort, 1:1 PSM	Single centre, Beijing	2018-07-01 to 2023-06-30	Pramipexole tablets (round 4)	I	738 vs. 738 after 1:1 PSM (935 vs. 750 before); 188 vs. 188 for the efficacy analysis	Levodopa equivalent dose trajectory, adverse events	No significant difference in either outcome	738 vs. 738 after 1:1 PSM (935 vs. 750 before); 188 vs. 188 for the efficacy analysis
(59)	Wang 2025 [423]	Retrospective cohort, PSM	Single centre, Beijing	2019-01 to 2023-06	Linezolid (rounds 3 and 5)	I	137 vs. 137 after PSM	Thrombocytopenia, severe thrombocytopenia, transfusion-threshold anaemia, platelet and haemoglobin change	Thrombocytopenia 28.44% versus 21.17%, severe thrombocytopenia 6.42% versus 4.95%, anaemia 2.75% versus 3.15%, all non-significant; changes in platelets and haemoglobin comparable	Xuanwu platform
(60)	Wen 2023 [220]	Multicentre retrospective cohort, PSM	Four centres, Guangdong	2020-01 to 2021-12	Vildagliptin tablets (round 3)	I	3,039 vs. 1,472	HbA1c and FPG reduction, adverse events, hypoglycaemia	No significant difference in any outcome	First 8 months precede announcement
(61)	Wu 2024 [X4]	Retrospective cohort	Single centre	2023-05 to 2023-12	Atorvastatin calcium tablets (round 1)	I	44 vs. 29	One-month TC, TG, LDL-C, non-HDL-C reduction; hepatic enzymes	No significant difference in lipid reduction or hepatic enzyme abnormality; treatment cost markedly lower with the selected product	Unmatched, unequal arms
(62)	Xiao 2024 [194]	Before-and-after retrospective cohort	Single centre, Leshan	Comparator 2017-03 to 2019-08; selected 2019-12 to 2022-03	Amlodipine besylate (round 1)	III	387 vs. 395	Response rate, ADR reporting	Response 96.9% vs. 98.0%; no ADR reported in either arm	Zero events; non-overlapping periods; Manufacturer-level verification not obtainable: Sichuan did not publish an award table and neither arm is identified by manufacturer in the source; excluded in sensitivity analysis S2
(63)	Xie 2021 [257]	Prospective randomised allocation	Single centre, Qianjiang	2017-01 to 2019-12	Atorvastatin calcium tablets (round 1)	I	75 vs. 75	Lipid profile, overall response, ADR, cost-effectiveness	Response 92.0% vs. 94.7% and ADR 10.7% vs. 12.0%, both non-significant	Two thirds of period precede announcement; Selected product verified to manufacturer level (Beijing Jialin, pilot winner); Chongqing was a pilot city
(5)	Xing 2025 [X7]	Multicentre retrospective cohort, 1:1 PSM	16 centres	2020-04-01 to 2022-03-31	Atorvastatin (round 1)	I	39,742 vs. 39,742 (treatment patterns); 3,610 vs. 3,610 (efficacy)	Lipid reduction, LDL-C reduction ≥50%, hepatotoxicity, CPK, SAMS, FPG, new-onset diabetes	Lipid outcomes comparable; AST elevation slightly more frequent with generic (*p* = 0.014); new-onset diabetes more frequent with originator (*p* < 0.001)	Text and tables transposed in source; a published correction (Lipids Health Dis 2025;24:345, doi:10.1186/s12944-025-02777-2) amends the text on hyperglycaemia, and the tables were followed
(64)	Xu 2024 [760]	Retrospective cohort	Single centre, Tengzhou	2021-05 to 2023-05	Valsartan/amlodipine (round 4)	I	100 vs. 100	Overall response, systolic and diastolic BP	Response 87.0% vs. 92.0% (*p* = 0.249); BP reduction comparable	Single adverse event overall
(65)	Xue 2024 [78]	Prospective randomised allocation	Single centre, Shenzhen	2021-01 to 2021-03	Amlodipine besylate (round 1)	I	100 vs. 100	Overall response, BP control, ADR, adherence	Overall response and ADR comparable; BP control 78.0% vs. 62.0% (*p* < 0.05)	Co-intervention: selected arm also received remote pharmaceutical care
(66)	Yan 2024 [243]	Retrospective cohort with outpatient follow-up	Single centre, Suzhou	2020-01 to 2022-06	Levetiracetam tablets (round 1)	I	53 vs. 72	Seizure control, seizure frequency reduction, ADR	Seizure control 44/53 (83.0%) vs. 63/72 (87.5%), chi-square 0.497, *p* = 0.481; all four adverse reactions occurred in originator-treated patients on monotherapy	The source performs no test of the adverse-reaction comparison and states that the number of reports is too small to support a conclusion; this study is therefore not counted as showing a safety difference
(67)	Yang 2021 [752]	Retrospective cohort, PSM	Single centre, Beijing	2019-03 to 2019-12	Pemetrexed (round 1)	I	38 vs. 38 after PSM	ORR, DCR, adverse events	After matching, objective response 10/38 vs. 12/38, disease control 34/38 vs. 36/38 and adverse events 18/38 vs. 9/38 (*p* = 0.055) did not differ	Pre-matching arms differed in stage, treatment intent, dose and number of cycles; the pre-matching adverse-event differences are not attributed to the medicine
(68)	Yang 2023 [185]	Retrospective cohort	Single centre, Jinan	2010-01 to 2022-12 (the hospital first stocked the selected capsule in December 2019)	Entecavir (round 1)	I	30 vs. 30	HBV-DNA, HBsAg and HBeAg clearance, ALT normalisation	No significant difference; generic favoured on cost-effectiveness	Selected product verified to manufacturer level (Fujian Guangshengtang, an expansion winner); the generic arm can only have been drawn from December 2019 onwards while the originator arm spans the whole period, so the calendar windows are not comparable
(69)	Yang 2023 [219]	Retrospective cohort, PSM	Single centre, Dalian	2020-01 to 2021-07	Ticagrelor tablets (round 3)	I	690 vs. 690 after PSM	12-month primary and secondary endpoints, bleeding	Primary 12.6% vs. 12.3% and secondary 11.0% vs. 8.3%, both non-significant	Generic manufacturer matched directly to the round-three list; first 8 months precede announcement
(70)	Yang 2024 [224]	Retrospective cohort	Single centre, Huizhou	2020-01 to 2022-12	Moxifloxacin sodium chloride injection (round 3)	I	54 vs. 21	Response rate, ADR, cost	Response 88.9% vs. 85.7% and ADR 7.4% vs. 4.8%, both non-significant	Unmatched, markedly unequal arms
(71)	Yang 2025 [84]	Before-and-after, random sampling	Single centre, Xi an	Pre 2021-03 to 05; post 2022-03 to 05	Ceftriaxone injection (round 5)	II	162 vs. 207	Clinical response, treatment duration, 7-day discharge, inflammatory markers, failure rate, ADR	No significant difference in any outcome; costs lower after policy	Non-overlapping periods one year apart
(72)	Yang 2024 [103]	Active sentinel surveillance	Three tertiary centres, Guangzhou	2022-10-01 to 2023-09-30	Oxaliplatin injection (round 5)	I	8,400 vs. 1,760 drug administrations (142 vs. 30 ADR reports)	ADR incidence (CTCAE 5.0)	ADR reporting rate 1.69% vs. 1.70%, *p* = 0.967; no significant difference in any organ system (P 0.12 to 1.00); grade 3–4 events mainly leucopenia, neutropenia, thrombocytopenia, hypotension and diarrhoea	Denominator is drug administrations and the numerator spontaneous reports, so the figure is a reporting rate rather than a cohort incidence; arms unbalanced (4.8:1), unadjusted, and no baseline characteristics of the treated populations are given; source states only “oxaliplatin injection” without the physical form but describes the comparator as non-selected throughout; safety outcomes only; disease-category table gives 143 vs. 29 against 142 vs. 30 elsewhere
(73)	Yang 2025 [26]	Retrospective cohort, 1:1 PSM	Single centre, Hangzhou	Originator arm 2019; generic arm 2023	Atorvastatin (round 1)	I	92 vs. 92	LDL-C reduction, target attainment, recurrence, ADR	No significant difference in any outcome	Groups drawn from calendar years four years apart
(74)	Ye 2024 [182]	Multicentre retrospective cohort	Four centres	2019-01-01 to 2022-04-01	Apixaban (round 3)	I	641 and 50 vs. 295	PT, INR, APTT, VTE, hepatic/renal indices, ADR	No significant difference across the three arms	Both generic arms matched directly to round-three winners (Qilu Hainan; Jiangsu Hansoh); first 19 months precede announcement
(75)	Yi 2025 [611]	Retrospective cohort	Single centre, Hezhou	2020-07 to 2022-07	Atorvastatin (round 1)	I	55 vs. 33	Lipid profile, ALT, AST, FPG, HbA1c, overall response	No significant difference in any outcome; cost-effectiveness favoured generic	Unmatched, unequal arms
(76)	Yi 2024 [603]	Multicentre retrospective cohort, 1:1 PSM	Five centres, Shanghai	2017-01 to 2024-06	Bortezomib for injection (round 4)	I	215 vs. 215 after PSM	Four-cycle staged response; M protein, plasma cell ratio, beta-2 microglobulin, creatinine, calcium, sFLC-lambda; somnolence, pain, peripheral neuropathy, rash, allergy, fatigue; affordability	Objective response 184/215 vs. 193/215, *p* = 0.053; fatigue less frequent with the selected product (29 vs. 58, *p* < 0.001); lactate dehydrogenase and free light chain kappa favoured the selected product and blood urea nitrogen the originator	Baseline urea nitrogen and free light chain kappa remained imbalanced after matching (both P < 0.001) and are the two indices whose change scores later differed
(77)	Yuan 2024 [X2]	Retrospective cohort	Single centre, Beijing	2021-07 to 2022-05	Cefuroxime sodium for injection (round 5)	I	280 total	Blood loss, antibiotic duration, stay, 24-h WBC and neutrophils, fever, postoperative infection, ADR	No significant difference in any outcome	—
(78)	Zhang 2020 [248]	Prospective randomised allocation	Single centre	2016-01 to 2019-01	Clopidogrel bisulfate tablets (round 1)	I	53 vs. 53	Overall response, angina episodes, platelet aggregation, ADR, cost	Overall response 98.11% in both arms (*p* = 1.000); ADR comparable; cost lower with generic	35 of 37 months precede announcement
(79)	Zhang 2023 [115]	Retrospective cohort, four parallel groups	Single centre, Qujing	2021-01 to 2021-11	Amlodipine besylate; irbesartan (round 1)	III	264 vs. 229; 291 vs. 263	Response rate, cost-effectiveness	No significant difference in response for either molecule	No safety outcome; two comparisons; Manufacturer not named in the source, so the Yunnan expansion list could not be matched
(80)	Zhang 2022 [197]	Genotype-stratified randomised allocation	Single centre, Kunming	2019-12 to 2020-12	Clopidogrel bisulfate (round 1)	I	40 vs. 40	ADP inhibition and MA-ADP at day 5 of loading and maintenance dosing, overall and by CYP2C19 metaboliser subgroup; 12-month exercise test, stent thrombosis, restenosis, adverse reactions; cost-effectiveness	Overall ADP inhibition and response grading comparable; within the slow-metaboliser subgroup, of FIVE patients per arm, ADP inhibition was lower with the selected product at both time points	Randomisation within genotype strata. Six subgroup comparisons without adjustment for multiplicity
(81)	Zhang 2022 [208]	Retrospective cohort, five-dimension evaluation	Single centre, Kunming	2017-03 to 2021-10	Amlodipine besylate; irbesartan (round 1)	I	404 vs. 419 for safety; efficacy reported separately for each molecule	Mean blood pressure reduction and response rate for each molecule and for combination regimens; in-hospital adverse reactions; decision-tree cost-effectiveness	No comparison of the selected and originator products under the same regimen differed. The two significant four-group comparisons resolve to pairs that either lie within the originator arm or cross regimens	Master’s thesis. Subgroup denominators are not reported alongside the response rates
(82)	Zhang 2022 [211]	Retrospective three-arm cohort with decision-tree analysis	Single centre, Hefei	2018-01 to 2021-06	Moxifloxacin injection (round 3)	I	74 vs. 72 (vs 75 non-selected)	Clinical response, cost-effectiveness	No significant difference in response across arms	No safety outcome; first 2.5 years precede announcement
(83)	Zhang 2024 [607]	Retrospective cohort	Single centre, Jiaxing	2021-01 to 2021-12	Cefuroxime sodium for injection (round 5)	I	148 vs. 148 after PSM for efficacy; 4,512 vs. 6,319 for safety	Prophylactic efficacy, WBC, CRP, peak temperature, stay, ADR	Prophylactic response 144/148 vs. 142/148, *p* = 0.750; neutrophil percentage higher with the selected product (*p* = 0.021); adverse reactions 14/4,512 vs. 12/6,319, *p* = 0.207	The source states the matched sample was too small for a safety comparison and used all patients treated at the hospital between 2020-01-01 and 2023-06-30 instead
(84)	Zhang 2023 [279]	Single-arm before-and-after switch	Single centre, Ningbo	2019-12 to 2022-04	Olanzapine tablets (round 1)	III (see note)	30	Dose-adjusted serum concentration, CGI-S, CGI-I, adverse events	CGI ratings unchanged after substitution; dose-adjusted serum concentration significantly lower with the bid-winning product	Both arms are selected products, one from the pilot stage and one from the expansion stage; this is not a comparison with an originator
(85)	Zhang 2025 [207]	Real-world retrospective cohort	Single centre, Urumqi	2021-01 to 2023-12	Empagliflozin tablets (round 4)	I	Not stated	Fasting plasma glucose, glycated haemoglobin, NT-proBNP	Both arms improved significantly from baseline (*p* < 0.05) with no significant between-group difference; adverse events not separately tabulated	Methodological section of the paper not extracted
(86)	Zhao 2023 [112]	Multicentre retrospective cohort	Four centres, Zhejiang	2021-01-01 to 2021-07-30	Pantoprazole (round 4)	III	96 vs. 104	Stress ulcer incidence	No significant difference	Comparator not product-identified
(87)	Zhao 2024 [281]	Population-based matched cohort (claims)	Tianjin	2018 to 2020; follow-up to 2020-12-30	Amlodipine (round 1)	I	2,949 vs. 5,943; matched 4,360 vs. 2,180	MACE, heart failure, atrial fibrillation, adherence	No significant difference in MACE; back-switchers showed higher stroke risk	—
(88)	Zhu 2024 [749]	Thesis; retrospective cohort, 1:2 PSM with Cox regression	Single centre, Yunnan	2019-01-01 to 2023-06-30	Rivaroxaban (round 5)	I	28 vs. 72 (10 mg); 67 vs. 34 (15 mg)	Ischaemic and haemorrhagic stroke, TIA, embolism, myocardial infarction, all-cause death; eight bleeding endpoints; minimum-cost analysis	No significant difference in any efficacy or safety endpoint before or after matching in either the 10 mg or the 15 mg stratum (all log-rank *p* > 0.05); Cox regression showed no increased risk; generic arms incurred lower total drug and Western medicine costs (*p* < 0.05)	Network meta-analysis chapter not extracted; first 2.5 years precede announcement

The first column gives the reference number used in the text and reference list; studies are additionally identified by first author and year with the screening record number in square brackets, which is the key to Supplementary Tables S2, S3, S6. Where several studies share an author and year, the record number distinguishes them. Comparator strata: I, originator product, including imported originator preparations; II, the same medicine at the same institution before the policy took effect; III, a non-selected generic of the same ingredient and dosage form; IV, within-patient comparison before and after substitution. Studies contributing to more than one stratum are shown with both. Sample sizes are the analysed sets; where a source applied a separate propensity model to each outcome, or reported different analysis sets for efficacy and safety, both are given.

Because several studies compared more than two arms or more than one medicine, the 81 studies yielded more eligible comparisons than studies; 69 of these fall in the originator-comparator stratum and constitute the main analysis. Seven studies contribute more than one comparison: three injectable cephalosporins in one multicentre study; four medicines in one before-and-after thesis; two antihypertensive molecules in each of two studies; two nucleos(t)ide pairs in one six-arm study; and two parallel arms against a shared comparator in each of two further studies. Where a multi-arm study contained arms that did not meet the eligibility criteria, only the eligible pair or pairs were extracted and the remaining arms were disregarded. Study-level and comparison-level counts are reported separately throughout. Three included studies have a companion report drawn from the same data source; each pair is counted as one study and both reports are cited (89–91), giving 84 reports for 81 studies.

### 3.3 Comparator strata

Comparator type determines what a null result can support, and studies were therefore analysed in four strata (Table 1, column 7).

Sixty-eight studies compared a procurement-selected generic with the originator product, including eight in which the originator was an imported preparation. A 69th comparison is contributed by a six-arm study that also contributes a comparison to the non-selected-generic stratum, so the originator-comparator stratum comprises 69 comparisons from 69 studies and constitutes the main analysis. Twelve further studies fall wholly outside it. The 81 studies therefore divide into 68 in the originator stratum alone, one contributing to both the originator and the non-selected-generic strata, and 12 in the remaining strata. Three studies compared the same medicine before and after the policy took effect at the same institution. Seven studies compared a selected generic with a non-selected generic of the same molecule and dosage form; one further study combined the pre-policy and non-selected features, and one used a within-patient design in which each patient served as his or her own control before and after substitution. One of the seven non-selected-generic studies also used a before-and-after switch within the same patients and is counted in that stratum rather than separately. Results from these 12 studies are reported in Section 3.7 and were not pooled with the originator comparison, because equivalence to another generic does not license inference of equivalence to the reference product.

One feature of the originator stratum requires explicit statement. The official supply lists show that for three products the originator itself was among the winning suppliers of the round concerned: linezolid tablets, levetiracetam concentrate for injection and moxifloxacin hydrochloride tablets. In those three studies the comparison is therefore between a selected domestic generic and a selected originator, not between a selected product and a product outside the programme. These studies are identified in Table 1 and the distinction is preserved wherever their results are cited. The dosage form matters here: linezolid tablets were procured with the originator among the winners in round three, whereas linezolid glucose injection was procured in round five without the originator. The three linezolid studies therefore differ in this respect: the tablet study (54) compares two selected products, the injection study (20) does not, and one study (59) covers both dosage forms, so its comparison is in part between two selected products. For first-round medicines the same question is province-specific rather than national, because the expansion awarded each medicine to at most three suppliers that then chose the provinces they would serve; no included study concerns a medicine for which this arose, but the comparator description for first-round products cannot be settled from the national list alone.

Table 2 summarises the direction of effect for the 69 comparisons in the originator-comparator stratum. The denominators differ between domains because not every study reports every domain: six comparisons report no efficacy outcome and four report no tested safety outcome. One further efficacy comparison, that of the six-arm nucleos(t)ide study, is reported in Section 3.6 but is not counted in the efficacy tally because the source did not test it. Each study is counted once in each domain to which it contributes. Figure 2 shows the same comparisons individually, each bar shaded by overall risk of bias.

**Table 2:** Direction of effect by outcome domain in the originator-comparator stratum.

Outcome domain	Comparisons	Favours originator	No significant difference	Favours selected product	No direction assigned
Therapeutic efficacy	62	0	60	0	2
Comparative safety	65	4	55	3	3
Pharmacokinetics	2	1	1	0	0

Each study is counted once in each domain to which it contributes; the denominators differ between domains because not every study reports every domain.

**Figure 2:**
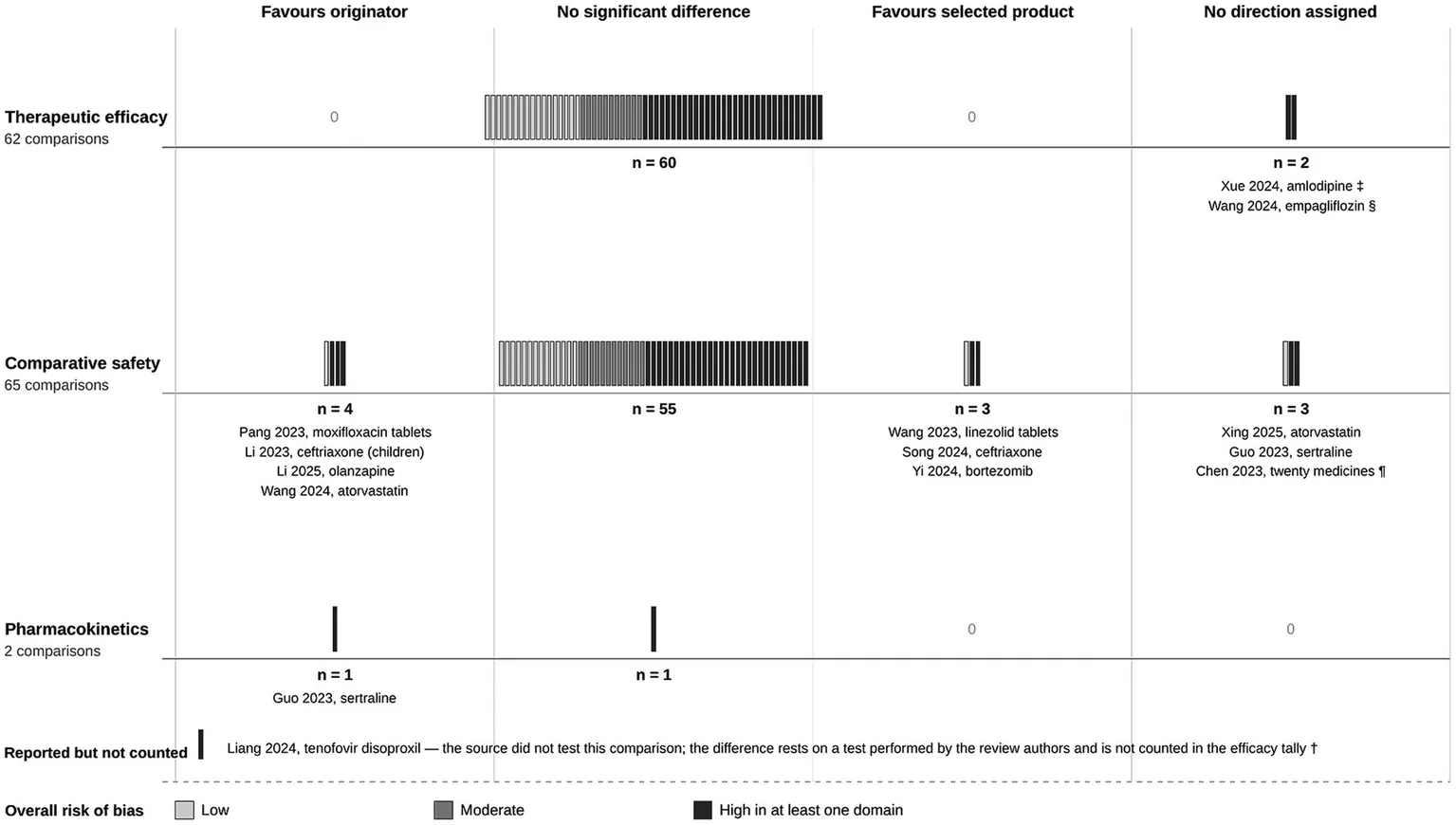
Direction of effect for each comparison in the originator-comparator stratum, by outcome domain. Each bar is one comparison; bars are shaded by overall risk of bias and ordered within each cell from lower to higher risk. Bar height is uniform and does not encode sample size, because no weighting was applied. Comparisons in the pre-policy, non-selected-generic and within-patient strata are not shown and are reported in Section 3.7.

No comparison in the efficacy domain has an assigned direction. The tenofovir disoproxil fumarate pair of a six-arm study, in which the reported rates differ, is not counted: the source did not test that comparison and the difference rests on a chi-square test performed by the review authors on reconstructed frequencies, which would not survive correction for the multiple comparisons available in that study. It is reported in Section 3.6 and examined in the sensitivity analyses. The two comparisons to which no direction is assigned in that domain are an amlodipine study in which the selected-product arm additionally received a remote pharmaceutical care programme, and an empagliflozin study in which the source defines the one index that differed in two mutually contradictory ways. In the safety domain the four comparisons favouring the originator are moxifloxacin tablets, olanzapine, paediatric ceftriaxone and atorvastatin; the three favouring the selected product are linezolid tablets, ceftriaxone and bortezomib; and the three to which no direction is assigned are sertraline and atorvastatin, in each of which significant findings within one cohort point in opposite directions, and a study reporting adverse-reaction rates in aggregate across 20 medicines. Studies in the pre-policy, non-selected-generic and within-patient strata are reported in Section 3.7 and do not appear in this table.

### 3.4 Efficacy in the originator-comparator stratum

Sixty-three of the 69 comparisons in this stratum reported an efficacy outcome. Six did not: two measured serum concentration without a clinical endpoint, two reported safety outcomes only, one reported adverse reactions across 20 medicines without an efficacy endpoint, and one compared the selected product only against itself before and after treatment.

Sixty of the 63 found no significant difference in the outcome on which the source based its conclusion. In one, the six-arm nucleos(t)ide comparison described in Section 3.6, the reported rates favour the originator, but the source did not test that comparison and it is not counted in Table 2; in two no direction was assigned. Findings by therapeutic class follow.

Cardiovascular and antithrombotic medicines (23 comparisons). Atorvastatin accounted for eight, amlodipine for four (one of them together with irbesartan), clopidogrel for four, ticagrelor for three, rivaroxaban for two, and apixaban and valsartan–amlodipine for one each. Lipid endpoints — absolute and percentage fall in low-density lipoprotein cholesterol, the proportion achieving a fall of at least 50%, and target attainment — showed no significant between-group difference in any study, including a 16-hospital matched cohort in which 3,610 pairs (5) were analysed for efficacy. Studies reporting clinical events found no difference in major adverse cardiac and cerebrovascular events or in bleeding at 12 months. Platelet-function endpoints were comparable overall in both clopidogrel studies that measured them.

Anti-infective medicines (16 comparisons). Moxifloxacin accounted for four, cefuroxime for three, ceftriaxone, meropenem and linezolid for two each, and levofloxacin, caspofungin and voriconazole for one each. A further linezolid study and a cefdinir study made no between-group efficacy comparison and contribute to the safety domain only. Clinical response, 72-h response, bacteriological clearance and normalisation of temperature, white cell count, neutrophil percentage, C-reactive protein and procalcitonin were comparable between arms in every study; in the largest, a 17-centre ceftriaxone cohort with 510 matched pairs (48), clinical response was 487 of 510 against 485 of 510.

Psychiatric and neurological medicines (5 comparisons). Three studies of levetiracetam and one each of pramipexole and olanzapine found no significant difference in seizure control, levodopa-equivalent dose trajectory or symptom rating scales. Studies of sertraline and paroxetine compared serum concentration or prescribed dose rather than a clinical endpoint and are reported in the pharmacokinetic domain.

Endocrine and metabolic medicines (8 comparisons). Reduction in glycated haemoglobin and fasting plasma glucose with empagliflozin, vildagliptin, saxagliptin, metformin and glimepiride, and serum urate target attainment with febuxostat, did not differ significantly between arms. In one empagliflozin study the source reports (55) a significant difference in a renal index but defines that index inconsistently between its text and its table, so no direction can be assigned; the same source reports glycated haemoglobin *p* values that its own test statistics do not support, and the arms differed in that measure at baseline.

Oncology and haematology medicines (5 comparisons). Objective response and disease control with capecitabine, pemetrexed and tegafur, staged response with bortezomib (184 of 215 against 193 of 215) (76) and two-cycle remission with azacitidine were comparable between arms. In the pemetrexed study the matched analysis (67), which is the source’s principal comparison, found no difference in response, disease control or adverse events.

Gastrointestinal and respiratory medicines (3 comparisons). *Helicobacter pylori* eradication with an omeprazole-containing regimen was 140 of 158 against 147 of 158 (31). In an esomeprazole study treatment response was 39 of 43 (24) against 41 of 43. In a budesonide trial the clinical control grading (13) did not differ.

Antiviral medicines (3 comparisons). In a single-centre cohort of 173 patients the selected tenofovir alafenamide (38) formulation and the imported originator did not differ in HBV-DNA negative conversion (64.9% versus 63.8%, *p* = 0.900), HBeAg seroclearance (9 of 24 versus 14 of 34, *p* = 0.778) or alanine aminotransferase normalisation (11 of 20 versus 11 of 21, *p* = 0.867). An entecavir study found no difference in any virological or biochemical endpoint. The third comparison is described in Section 3.6.

### 3.5 Safety in the originator-comparator stratum

Sixty-five of the 69 comparisons reported a tested safety outcome. Four did not: three reported no safety data at all, and one reported laboratory indices and event counts without any between-group test. Fifty-five of the 65 found no significant difference, four favoured the originator, three favoured the selected product, and in three no direction was assigned: in two the significant findings within one cohort pointed in opposite directions, and in one the source reported adverse-reaction rates in aggregate across 20 medicines without identifying which differed or giving a denominator. The 10 comparisons in which a difference was reported are set out individually in Section 3.6.

Three features limit the strength of the concordant majority. Event counts were small in many studies: several compared arms of fewer than 60 patients, and in two no adverse event occurred in either arm. Denominators are not uniform: five studies report adverse events against pre-matching group sizes while reporting laboratory abnormalities against matched pairs, one uses a hospital-wide population for safety and a matched cohort for efficacy, and one reports three laboratory indices against three different denominators because patients of indeterminate status were excluded from each. Finally, six studies reported safety outcomes only and six reported efficacy outcomes only, so the two domains do not rest on the same set of studies.

### 3.6 Studies reporting between-group differences

Every comparison in this stratum that reported at least one statistically significant between-group difference surviving verification against the source article is set out individually below, because such differences are not visible in an aggregate statement of concordance, and because in several cases the difference does not determine the direction assigned to the study.

Differences favouring the originator (four studies, all in the safety domain). In an outpatient cohort of 2,179 patients with community-acquired pneumonia (43), total adverse drug reactions were more frequent with the selected moxifloxacin tablet (76 of 1,121 against 25 of 1,058, *p* < 0.001), as were allergic reactions (18 against 3, *p* = 0.002), gastrointestinal reactions (30 against 11, *p* = 0.009) and abnormal blood glucose (17 against 5, *p* = 0.015); clinical response, treatment course and re-attendance did not differ, and a modified Poisson model found no increased risk of treatment failure (RR 1.132, 95% CI 0.883 to 1.542). In an olanzapine outpatient cohort (28), glucose abnormality was more frequent with the selected product (29 of 288 against 8 of 174, *p* = 0.036) while lipid abnormality and total adverse reactions did not differ. In a paediatric ceftriaxone study (25), adverse reactions were more frequent with the selected product (8 of 140 against 2 of 152) and defervescence was slower; this comparison is at the threshold of significance, giving *p* = 0.039 by chi-square and *p* = 0.052 by Fisher’s exact test, and clinical response did not differ. In an atorvastatin cohort, creatine kinase (57) was higher with the domestic product at 12 weeks; lipid endpoints, transaminases and the adverse reaction rate (37 of 263 against 25 of 255, *p* = 0.135) did not differ, and the arms differed at baseline in body mass index and hypertension prevalence without matching or adjustment.

Differences favouring the selected product (three studies, all in the safety domain). In a multicentre linezolid tablet study (54), hepatic injury occurred in none of 60 patients receiving the selected product against 3 of 35 receiving the originator (Fisher *p* = 0.046); pancytopenia, gastrointestinal events and clinical response did not differ, and the originator in this comparison was itself a winning supplier of the round. In a 17-centre ceftriaxone study (48), neutrophil reduction was less frequent with the selected product (10 of 510 against 28 of 510); recalculation from the reported counts gives *p* = 0.003. In a five-hospital bortezomib cohort (76), fatigue was less frequent with the selected product (29 of 215 against 58 of 215, *p* < 0.001), while peripheral neuropathy was more frequent without reaching significance (44 against 29, *p* = 0.053).

Opposing directions within one cohort (two studies). In a sertraline cohort (18), the proportion with high-density lipoprotein cholesterol below the reference range was higher with the selected product (41.8% against 38.3%, *p* = 0.039) while the proportion with low-density lipoprotein cholesterol above the reference range was lower (41.8% against 45.4%, *p* = 0.032). In the 16-hospital atorvastatin cohort (5), aspartate aminotransferase at least three times the upper limit was more frequent with the generic (96 of 6,335 against 64 of 6,335, *p* = 0.014) while hepatoprotective prescribing (908 of 52,765 against 1,054 of 52,765, *p* = 0.001), the rise in fasting plasma glucose (*p* = 0.009) and new-onset diabetes (664 of 28,640 against 1,244 of 28,640, *p* < 0.001) were all less frequent. No direction is assigned in either.

A difference in the efficacy domain, identified by the present authors (one study). In a six-arm study of nucleos(t)ide analogues (35), sustained virological response at 48 weeks was 48.5% with selected tenofovir disoproxil fumarate against 66.7% with the originator. The source compared every arm against tenofovir amibufenamide only and states so explicitly; it did not test the selected products against their own comparators. Using the arm sizes and percentages given in the source we reconstructed the frequencies and applied chi-square tests: 52 of 107 against 54 of 81, chi-square 6.12, *p* = 0.013, and *p* = 0.020 with continuity correction. This analysis was performed by the present authors and is not reported in the source; the reconstruction carries a rounding uncertainty of one patient, the difference would not survive Bonferroni correction across the 15 pairwise comparisons available in that study, and the arm sizes themselves, while consistent with every reported percentage, were not read directly from the source. The source reports that renal function did not differ between the same two arms. This comparison is reported here but is not counted in the efficacy tally of Table 2, because a direction that rests on a test the review authors performed rather than one the source reported should not contribute to the tally. The efficacy domain of the main analysis therefore contains no comparison with an assigned direction.

A difference not attributable to the medicine (one study). In an amlodipine trial the proportion achieving blood-pressure control (65) was higher with the selected product (78 of 100 against 62 of 100, *p* < 0.05), but the selected-product arm additionally received a remote pharmaceutical care programme that the comparator arm did not, so the difference cannot be attributed to the medicine and no direction is assigned.

Significant findings in secondary or surrogate measures that do not determine direction (five studies). In an esomeprazole study, episodes of haematemesis (24) and of melaena were fewer with the selected product at all 10 time points measured over 5 days, with every test statistic reproducing from the printed summary statistics; treatment response, the primary outcome, did not differ, and the arms differed in mean age by 13 years although the source describes them as comparable. In a budesonide trial, dyspnoea and rhonchi (13) resolved faster and the forced expiratory volume in 1 s was higher with the selected product, all three favouring it; clinical control grading did not differ. In a cefuroxime prophylaxis study (83), the postoperative neutrophil percentage was higher with the selected product (*p* = 0.021) while prophylactic response, white cell count, C-reactive protein, peak temperature and length of stay did not differ. In a genotype-stratified clopidogrel trial (80), ADP inhibition was lower with the selected product within the slow-metaboliser subgroup at both time points, but that subgroup contains five patients per arm and six subgroup comparisons were made without adjustment for multiplicity; overall inhibition and the other two genotype subgroups showed no difference. In a comprehensive evaluation of amlodipine and irbesartan (81), two four-group comparisons reached significance, but the source reports that the pairwise differences lay between the originator dual-therapy group and the originator dual-plus-metoprolol group, and between the selected dual-therapy group and the originator dual-plus-metoprolol group; neither pair compares the selected and originator products under the same regimen.

Findings whose direction could not be determined (one study). In an empagliflozin cohort a renal index (55) differed significantly after treatment (*p* = 0.002), but the source calls it the creatinine clearance rate in its text, for which a higher value is better, and defines it as creatinine in the legend of the same table, for which a higher value is worse, and gives no unit; the two readings imply opposite directions. In the same source the reported *p* values for glycated haemoglobin are not consistent with its own test statistics, which indicate a difference both before and after treatment, so the post-treatment difference cannot in any case be attributed to the medicine.

Differences confined to a pre-matching comparison (one study). In a pemetrexed cohort, total adverse events (67), myelotoxicity and hepatotoxicity were all more frequent with the selected product before matching. The arms at that stage differed in disease stage (III or IV in 92% against 79%), in the proportion receiving palliative or maintenance chemotherapy, in median dose and in median number of cycles. After matching none of the comparisons differed, and the matched analysis is the source’s principal comparison.

Differences in treatment-pattern measures (two studies). In a metformin cohort of 4,077 matched pairs (40), the originator arm had higher defined daily doses and higher three- and six-month persistence while the selected arm had a lower daily cost and higher single and repeat switching rates (all *p* < 0.001); the clinical and laboratory comparisons in the same study showed no difference. In a linezolid study the daily dose (54) was higher with the selected product in respiratory and other-site infection. These are measures of use rather than of clinical outcome and are reported in Supplementary Table S3 without a direction.

One further study in this stratum reports adverse drug reaction rates in aggregate across 20 medicines (11) and states that the rate was lower with the selected product for an unspecified minority of them. Because the source identifies neither the medicines concerned nor any denominator, no direction is assigned to it.

A note on the reliability of the sources is required here rather than in the discussion, because it bears directly on the figures above. Of the 24 studies whose full texts were re-examined, six report test statistics that cannot be reproduced from their own summary statistics or counts. In one, every reported pairing of t and P is arithmetically impossible and the abstract contradicts the Results section. In another, the reported *p* values for glycated haemoglobin are inconsistent with its own test statistics. In a third, two of three safety comparisons presented as significant are not significant on recalculation, and those two have been removed from the account above. In a fourth, a 13-year difference in mean age is described as no difference. In a fifth, the Discussion section states the opposite of the table for one outcome and transposes the values of another; the tables have been followed throughout. In a sixth, a reported test statistic differs from its recalculated value although both indicate significance. Wherever a source’s text, abstract or discussion conflicts with its tables, the tables have been used, and each such conflict is recorded in Supplementary Table S3.

### 3.7 Non-originator comparator strata

Seven studies set a selected product against a non-selected generic of the same molecule, and one further study contributed a comparison of this kind. In a multicentre randomised trial in which the regimens differed only in whether the amoxicillin component had been procured centrally, eradication was 90.7% against 92.1% in per-protocol analysis and 84.7% against 85.3% in intention-to-treat analysis; that study was extracted from the publisher’s abstract, its arm sizes are not reported and its adverse events are given only as ranges pooled across two arms. In a cohort of patients already in complete virological response on domestic entecavir who did or did not switch to the selected product, complete response was 151 of 156 against 101 of 104 at 24 weeks and 100% in both arms at 48 weeks, with no adverse event in either arm; the ceiling effect in this population limits the sensitivity of the comparison. In a 19-institution study of three injectable cephalosporins, clinical response was comparable for all three, while normalisation of C-reactive protein was more frequent with selected ceftazidime and platelet reduction and eosinophil elevation were more frequent with selected cefazolin and ceftazidime respectively; the denominators for those laboratory comparisons differ by index and are not reported. In the six-arm study, the selected tenofovir alafenamide arm had higher LDL cholesterol, triglycerides and triglyceride to HDL cholesterol ratio than the non-selected arm at 48 weeks. The remaining comparisons in this stratum found no significant difference.

Three studies compared the same medicine before and after the policy at the same institution, and one combined that design with a non-selected comparator. Clinical response and adverse reaction rates were comparable in all four. In each, the two groups were drawn from non-overlapping calendar periods, so secular change in clinical practice cannot be separated from any product effect.

One study used a within-patient design in children switched from the imported originator to a domestic generic levetiracetam; seizure control rose from 77.3 to 83.8% after substitution and three mild adverse reactions were recorded against one before. A further study, also within-patient, switched adults between two generic olanzapine products, one of which was the bid-winning product. The log-transformed dose-adjusted serum concentration was significantly lower with the bid-winning product, while Clinical Global Impressions ratings did not change. Both products in that study were winning suppliers, one of the pilot stage and one of the expansion stage, so it compares two selected products rather than a selected product with an originator, and it does not enter the originator-comparator synthesis. Its authors conclude that one generic psychotropic medicine may not be therapeutically equivalent to another.

### 3.8 Economic outcomes

Economic findings are reported for completeness and are treated as context rather than as an outcome of the review. Acquisition costs of selected products were between 44% and more than 90% lower than those of their comparators in every study reporting them. That difference is the mechanism of the policy rather than a finding about it: prices were bid down by design, and a review of clinical studies adds nothing to what the procurement notices already state. The findings that do bear on the policy are those in which total treatment cost was measured: in one 17-centre study total hospitalisation cost was CNY 4,211.66 against 5,748.74 and antimicrobial cost CNY 37.94 against 605.40 (both *p* < 0.001), while other in-hospital costs did not differ, and cost-effectiveness ratios favoured the selected product wherever they were calculated. Three qualifications apply even to these: most analyses counted direct acquisition costs only, few applied discounting, and time horizons rarely extended beyond the treatment episode, so downstream costs of any difference in efficacy or safety were not captured. In one study the printed cost table is internally inconsistent and its figures are not cited.

### 3.9 Risk of bias

Risk of bias was assessed in eight domains, three of which are specific to the policy setting (Supplementary Table S2).

Exposure misclassification was the dominant limitation. Twenty-one studies began recruitment more than 12 months before the announcement of the relevant round, or overlapped the post-announcement period by less than 3 months; a further 16 began recruitment within 12 months before the announcement. In the most extreme case recruitment opened almost 9 years before the medicine entered a procurement round, at a hospital that did not stock the selected product until December 2019, and in another 35 of 37 months of recruitment preceded the announcement. In these studies, patients recorded in the selected-product arm during the pre-announcement period cannot have received a product holding selected status, and the exposure contrast is correspondingly diluted. Forty-two studies were free of this problem. In one study the recruitment window was not reported and in one the medicines evaluated belonged to several different rounds, so no single announcement date applies.

Secular confounding arose in 10 studies whose arms were drawn from non-overlapping calendar periods, in one instance separated by 4 years. Comparator identifiability was moderate in seven studies, in which the comparator is stated to be the originator but the product is not named, and high in two, in which it is a non-selected generic that is not named.

Confounding control was graded high in 12 studies that neither matched nor adequately adjusted and whose arms differed at baseline. In one cohort of 1,620 patients the arms differed significantly in infarction type and in concomitant medication and only regression adjustment was applied. In an active surveillance study the two arms differed almost fivefold in the volume of drug administered and no characteristics of the treated populations were reported at all. In one study allocated by admission order the arms differ in mean age by 13 years while the source describes them as comparable. One further study was graded moderate: it applied propensity score matching, but two baseline indices remained imbalanced after matching and those are precisely the two indices whose change scores later differed.

Co-intervention and centre effect were graded high in two studies: in one the selected-product arm also received a remote pharmaceutical care programme that the comparator arm did not, and in the other treatment arm and study centre were completely collinear.

Outcome reporting was graded high in 15 studies and moderate in seven. The high gradings arise where one outcome domain is wholly unreported, where only a within-arm comparison is made, where no event occurred in either arm, where adverse reaction rates are given in aggregate across 20 medicines with no denominator, or where the reported result cannot be reconciled with the study’s own data. The moderate gradings arise where denominators or subgroup sizes are not reported, where safety was assessed in a different population from efficacy, or where the significant findings are confined to a pre-matching comparison.

This last point requires a plain statement. Twenty-four included studies were re-examined against the source article during this revision. Six report test statistics that cannot be reproduced from their own summary statistics or counts, and in one the Discussion section states the opposite of the tables for one outcome and transposes the values of another. In one of the six, every reported pairing of t and P is arithmetically impossible and the abstract contradicts the Results section. In another, two of three safety comparisons presented as significant are not significant on recalculation. Wherever a source’s text, abstract or discussion conflicted with its tables, the tables were used, and each conflict is recorded in Supplementary Table S3.

Independence of data sources: 23 of the 81 studies originate from five research groups or multicentre platforms. These studies address different medicines and do not duplicate patients, but they are not independent sources of evidence. One study was extracted from the publisher’s abstract because the full text could not be obtained; all others were extracted from the full text.

Verification of selected status is reported in Section 2.2 and its limits are stated there. The manufacturer of the selected product matched the official award list directly in 21 studies. In one study the manufacturer named for the selected arm does not appear on the corresponding list and the approval numbers of the two arms appear to have been transposed in the source; that comparison is treated as unverified. The study found no significant difference in any endpoint, so its removal would not alter the direction of any tally. Three first-round studies are unverified at manufacturer level, two because the source does not name the manufacturer or names it only by approval number, and one because neither arm is named and its province published its awards only within its procurement platform.

### 3.10 Sensitivity analyses

Eleven sensitivity analyses were conducted (Supplementary Table S7). The first two are those specified in the original submission: exclusion of any study rated high in any risk-of-bias domain, which implements the original restriction to studies at low or moderate overall risk now that risk of bias is assessed by domain rather than overall (32 studies remain); and restriction to multicentre studies (19 remain). The remaining nine were added during this revision: restriction to the originator-comparator stratum (69 comparisons); exclusion of studies at high risk of exposure misclassification (58); exclusion of studies whose arms are drawn from non-overlapping calendar windows (69); exclusion of studies without matching or adequate adjustment (67); exclusion of the study extracted from an abstract only (78); exclusion of studies with incomplete or irreconcilable outcome reporting (64); retention of a single study per research group or platform (61); exclusion of comparisons tested only by the review authors (68 comparisons); and assignment of a direction wherever any outcome of a study’s principal analysis differed significantly (79).

When every study rated high in any risk-of-bias domain is excluded, 32 of the 81 studies remain. Within that subset no comparison in the efficacy domain has an assigned direction, and the safety domain retains one comparison in each direction. The pattern is unchanged and becomes more symmetrical.

When the synthesis is restricted to the 19 multicentre studies, the balance of the safety signals reverses: of the four comparisons that retain a direction, three favour the selected product and one favours the originator. Three of the four comparisons favouring the originator in the full set are single-centre studies, so the signals favouring the originator arise disproportionately from smaller single-centre work. No comparison in the efficacy domain retains a direction.

An eleventh analysis tested the rule by which direction was assigned, assigning a direction wherever any outcome of a study’s principal analysis differed significantly rather than only where the primary outcome did. Under that rule the efficacy domain contains three comparisons favouring the originator, three favouring the selected product, three to which no direction can be assigned and 54 showing no difference, a total of 63; the denominator here is the 63 comparisons that report an efficacy outcome (Section 3.4) rather than the 62 counted in Table 2. The safety domain is unchanged. The two directions remain in balance. The pattern of findings was otherwise unchanged. Two further analyses are consequential for interpretation. When each group or platform contributes once, the evidence base falls from 81 studies to 63, and the medicines represented fall disproportionately in the cardiovascular and anti-infective classes. The analysis that excludes comparisons tested only by the review authors leaves the efficacy tally unchanged, because the one comparison of that kind is already excluded from the tally in the main analysis (Section 3.6); the analysis is retained because it also removes that comparison from the narrative account.

### 3.11 Certainty of evidence

Certainty was rated for the originator-comparator stratum using GRADE (Supplementary Table S4). All included studies except one randomised trial are non-randomised, so each domain began at low certainty. Certainty was rated down for risk of bias in every domain, for imprecision in every domain, and for indirectness in the three clinical domains; it was rated down for inconsistency in comparative safety, where 10 of 65 comparisons show a difference in both directions without any explanation by medicine, drug class or design. It was rated down for publication and reporting bias in the three clinical domains: publication bias could not be assessed by funnel plot because no outcome was reported as a commensurable estimate by enough studies, and qualitative assessment found that no included study could be linked to a prospective registration, that 23 of 81 arise from five groups or platforms, and that one study reported the underlying rates of a comparison without testing it. No domain was rated up.

Certainty is therefore very low for therapeutic efficacy, for comparative safety and for pharmacokinetics, and low for economic outcomes. A rating of very low means that the true effect is likely to be substantially different from the estimate. The finding that most comparisons show no significant difference should not be read as evidence of equivalence: an effect estimate was unavailable for 42 of the 81 studies, many comparisons were made in arms of fewer than 100 patients, and the one reported difference that rests on a subgroup of five patients per arm was not allowed to determine direction.

## 4 Discussion

This systematic review synthesised evidence from 81 studies evaluating the clinical efficacy, safety and pharmacokinetic characteristics of medicines procured through China’s NVBP policy. Sixty-eight of those studies, together with one comparison contributed by a six-arm study, compared a procured generic with the originator product and form the main analysis; the remaining 12 used a pre-policy baseline, a non-selected generic or a within-patient design and are reported separately.

The central finding is that, across more than 40 active ingredients, procured generics showed efficacy and comparative safety similar to their comparators in the large majority of comparisons. Sixty of the 62 counted efficacy comparisons showed no significant difference, and no comparison in that tally carries an assigned direction. In one further comparison the reported rates differ, but the source did not test it: that difference rests on a test we performed ourselves on reconstructed frequencies, and it is reported without being counted. In the two comparisons to which no direction is assigned, the selected-product arm in one also received a remote pharmaceutical care programme, and in the other the source defines the single index that differed in two mutually contradictory ways. Among the 65 comparative safety comparisons, 55 showed no difference, four favoured the originator (total adverse reactions with moxifloxacin tablets, glucose abnormality with olanzapine, adverse reactions with paediatric ceftriaxone, and creatine kinase with atorvastatin), three favoured the procured product (hepatic injury with linezolid tablets, neutrophil reduction with ceftriaxone, and fatigue with bortezomib), and in three no direction was assigned: in two the significant findings within one cohort pointed in opposite directions, and in one the source reported adverse-reaction rates in aggregate across 20 medicines without identifying which differed or giving a denominator.

This pattern was consistent regardless of study design. It is more consistent with expected between-study variation than with systematic inferiority of generics, but the consistency should not be overstated: an effect estimate is available for only 39 of the 81 studies, and the aggregate is dominated by small single-centre retrospective cohorts.

These findings are broadly consistent with the international evidence base on generic substitution. A landmark systematic review by Kesselheim et al. (92) examined 47 studies comparing cardiovascular generics with brand-name drugs and concluded that clinical equivalence was the norm. The NVBP context nonetheless differs fundamentally from the settings in which most international evidence has been generated. In the United States, the European Union and Australia, generic substitution typically occurs at the pharmacy level, patients generally retain the right to request the originator, and physicians can override substitution when clinically indicated; this model presupposes a market in which both products remain available. The NVBP policy operates through policy-driven mandatory conversion at the institutional level: winning manufacturers receive guaranteed procurement volumes, and non-selected products, including originator brands, may be effectively unavailable within the hospital formulary. Patients and prescribers thus face a near-complete loss of choice rather than a voluntary substitution decision. If reassuring evidence emerges from a system where patients cannot opt out, that is a stronger real-world test of generic equivalence than evidence from settings where dissatisfied patients can switch back. Equally, the mandatory nature of conversion means that any quality or safety signal, even a rare one, reaches a much larger share of the patient population, which is an argument for surveillance designed specifically for this environment rather than for transferring international substitution evidence directly.

The strength of these conclusions must be tempered by the state of the evidence base. Of the 81 included studies, one was a multicentre randomised controlled trial, seven allocated patients prospectively, one was a prospective observational cohort and the remaining 72 were retrospective or before-and-after in design; 62 were single-centre. The contrast with the evidence base for biosimilars is instructive: 12 head-to-head randomised trials have been documented in inflammatory diseases (93), whereas this review identified one randomised trial of NVBP chemical generics, and that trial could be extracted only from its published abstract.

The composition of the included evidence follows the timeline of the policy. The policy was launched in late 2018 and the clinical research cycle introduces a lag of several years; the majority of included studies appeared between 2023 and 2025, indicating a field in early exponential growth. This review therefore provides a snapshot at a critical juncture and a structured methodological baseline against which rapidly accumulating future evidence can be evaluated.

One pharmacokinetic observation warrants particular attention. In a within-patient study, one procured olanzapine formulation produced a significantly lower dose-corrected steady-state concentration than another procured formulation although each had demonstrated bioequivalence to the reference product; both products in that comparison were winning suppliers, one of the pilot stage and one of the expansion stage. China currently lacks a unified, dynamically updated national narrow therapeutic index catalogue and a tiered substitution framework. Given that several procured medicines in this review are classified as narrow therapeutic index agents in other jurisdictions, differentiated management protocols for their procurement should be considered a policy priority.

Generalisability is further constrained. Most included studies were conducted in major urban centres, and applicability to resource-limited settings, rural areas and ethnically diverse populations remains uncertain. Distinct patterns of medication adherence have been documented in a Tibetan autonomous region (94), where geographic isolation, altitude-related physiological differences and cultural barriers create confounding factors not captured in studies from eastern Chinese cities.

### 4.1 Strengths and limitations

This review has several strengths. It is, to our knowledge, the first systematic review focused specifically on the clinical outcomes of NVBP-procured medicines. The search covered Chinese and English sources in two arms, one anchored on procurement terminology and one on generic-versus-originator terminology. Eligibility was determined by the identity of the product evaluated rather than by the vocabulary of the report, and was applied to all 862 full-text reports through a fixed eight-question sequence, screened in duplicate, with the reason for every exclusion recorded and a random sample of 400 of the 781 exclusions independently re-assessed. Risk of bias was assessed in eight domains, three of which were developed for this policy setting because no existing instrument captures them. Twenty-four included studies were re-examined against the source article, and every figure reported here was verified against either that source or the screening record.

The limitations are correspondingly specific. First, the clinical and methodological heterogeneity across drug classes, designs and comparator types precluded quantitative meta-analysis. Second, the synthesis classifies each comparison by the outcome of a significance test. That metric ignores magnitude and clinical relevance, is prone to false-negative findings in small studies, and means that no significant difference cannot be equated with demonstrated equivalence; it was retained only because most included studies report nothing else. An effect estimate is available for 39 of the 81 studies — four reported by the source with a confidence interval and 37 calculated by us from counts or summary statistics, two studies by both routes — while for 42 the source reported only a significance test. Supplementary Table S3 gives every comparison as reported, with an estimate wherever the source data allowed one. Corresponding authors were contacted for the missing values and none were supplied.

Third, exposure misclassification is the dominant threat to validity in this literature. Twenty-one of the 81 studies began recruitment more than 12 months before the medicine entered the relevant procurement round, or overlapped the post-announcement period by less than 3 months; in one the window opened almost 9 years before, and in another 35 of 37 months of recruitment preceded the announcement. Patients recorded in the selected-product arm during those periods cannot have received a product holding selected status, so the exposure contrast is diluted and the observed concordance is, to an unquantifiable degree, a comparison of like with like.

Fourth, the included evidence is less independent than the number of reports suggests: 23 of the 81 studies originate from five research groups or multicentre platforms working within shared hospital information systems. When each group contributes a single study, the evidence base falls to 63.

Fifth, and most consequentially for how the individual results should be read, six of the 24 studies re-examined against their source articles report test statistics that cannot be reproduced from their own summary statistics or counts. In one, every reported pairing of t and P is arithmetically impossible and the abstract contradicts the Results section. In another, two of three safety comparisons presented as significant are not significant on recalculation, and those two have been removed from our account. In a third, the Discussion states the opposite of the tables for one outcome and transposes the values of another. In a fourth, a 13-year difference in mean age between arms is described as no difference. Wherever a source’s text conflicted with its tables we used the tables, and each conflict is recorded in Supplementary Table S3; but the frequency of such discrepancies is itself a finding about the reliability of this literature.

Sixth, title and abstract screening was conducted by one reviewer with a second checking, as specified at registration, rather than independently in duplicate. A *post hoc* reliability check in a random sample of 500 records gave a Cohen’s kappa of 0.78 (95% CI 0.73 to 0.84), which is substantial but not complete agreement; individual records may therefore have been missed at that stage. The corresponding check at the stage that determines inclusion is reassuring: re-assessment of a random half of the excluded reports agreed with the recorded decision in 393 of 400 cases, and every discrepancy was resolved in favour of the recorded decision. The kappa reported here is an estimate made after the event and not a record of the original screening process.

Seventh, publication and reporting bias cannot be excluded and could not be assessed by funnel plot, because no outcome was reported as a commensurable estimate by enough studies. No included study could be linked to a prospective registration, and one study reported the underlying rates of a comparison without testing it.

Eighth, the predominance of retrospective observational designs introduces selection bias and residual confounding; 12 studies neither matched nor adequately adjusted and had arms that differed at baseline. Ninth, conference abstracts and unpublished regulatory data were not eligible; seven master’s theses that met the criteria and were retrieved by the search are included, so coverage of unpublished academic work is partial rather than absent. Tenth, three included studies each have a companion report drawn from the same data source; each pair is counted once and both reports are cited. Eleventh, because the included studies span heterogeneous drug classes, the consistency in direction should be read as a cross-class pattern rather than a class-specific or pooled estimate; no medicine is supported by more than nine studies. Twelfth, Embase was listed at registration but could not be searched because institutional access was unavailable, and bibliographic records could not be exported from Cochrane CENTRAL. Thirteenth, none of the observational studies stated how many participants were lost to follow-up, so completeness of follow-up could not be judged for any of them. Fourteenth, the evidence assembled here predates the nationwide renewal of procurement contracts for the first eight rounds announced in February 2026, so it characterises the initial procurement cycles rather than the programme in its present form.

A further limitation concerns what these studies were able to detect. No included study reports a sample-size calculation. Using the event counts and group sizes reported in Supplementary Table S3, we calculated for each comparison in the main analysis the smallest difference it could have detected with 80% power at a two-sided alpha of 0.05. Among the 22 efficacy comparisons for which this could be calculated, the median smallest detectable fall in the response rate was 16.5 percentage points (interquartile range 10.2 to 20.3); 17 of the 22 could not have detected a fall of less than 10 percentage points. Among the 27 safety comparisons for which it could be calculated, the median smallest detectable relative risk was 2.67 (interquartile range 1.87 to 3.92); 19 of the 27 could not have detected a doubling of risk and 11 could not have detected a tripling. The concordance of null findings reported here is therefore in large part a property of the sample sizes available, and not a measurement of equivalence. A difference of the size that would concern a prescriber would have gone undetected in most of these studies.

A further limitation concerns the rule by which direction was assigned. Most included studies do not designate a primary outcome, so for those studies the outcome on which direction turns was chosen by us rather than by the source. That choice converted a statistically significant finding into no assigned direction in six studies. To test whether the rule shapes the result, a sensitivity analysis assigned a direction wherever any outcome of a study’s principal analysis differed significantly (Section 3.10). Under that rule the efficacy domain contains three comparisons favouring the originator, three favouring the selected product, three to which no direction can be assigned and 54 with no difference; the safety domain is unchanged. The balance between the two directions is therefore not an artefact of the rule, but the number of comparisons classified as showing no difference is sensitive to it.

A further limitation concerns the completeness of the rebuilt search itself. Two studies of glimepiride that meet every eligibility criterion, and that had been included in the version of this review submitted for peer review, were not retrieved by the rebuilt search of 2,903 records and were not recovered by citation searching of the included studies. They were found only when the reference list of that earlier version was compared with the screening record, and they are included here as two of the 81 studies. We report this because it bears on the argument of Section 4.3: a search rebuilt across six sources in two arms still failed to retrieve two eligible studies that were known to exist, and the reason it failed is not apparent from the search records. The deficit described in Section 4.3 should therefore be read as a lower bound on what terminology-based retrieval misses in this field, not as a measure of it.

The importance of confounding control in this literature is illustrated by one included study in which the generic arm showed significantly higher rates of adverse events, myelosuppression and hepatotoxicity before matching, but also contained more advanced-stage patients receiving higher doses over more cycles; after propensity score matching none of these differences remained.

### 4.2 Implications for policy and practice

Before turning to clinical implications, these findings should be situated within China’s regulatory quality-assurance framework, which governs the chemical and manufacturing dimensions of drug quality that lie outside the scope of the clinical evidence synthesized here. Eligibility for NVBP participation is conditional on passing the national generic consistency-evaluation programme, which requires demonstrated pharmaceutical equivalence and bioequivalence to the reference product (1). Beyond market entry, NVBP-selected products and their manufacturers remain subject to ongoing post-market regulatory surveillance, including routine sampling and inspection. As regulatory context rather than as data from the included clinical studies, official communications report that, among the more than 1,600 winning products across the first nine procurement rounds covered by that communication, nine were withdrawn from selection for identified quality risks, three of them originator products, indicating that the surveillance system detects and acts on quality signals across both generic and originator products (95). Those figures count winning products at manufacturer level and are therefore not comparable with the 491 procurement items against which eligibility was judged in this review (Section 2.2). The reassuring clinical findings of this review should therefore be understood as complementary to, rather than a substitute for, this regulatory quality-assurance system. These findings support the continued implementation of the procurement policy from a clinical safety perspective, while indicating where refinement could strengthen quality assurance. Drawing on the tiered risk-management framework proposed in the comparative policy literature (96), procurement administrators could establish differentiated procurement and monitoring protocols for narrow therapeutic index medicines, with post-substitution therapeutic drug monitoring for the highest-risk agents, prescriber-directed substitution for intermediate-risk agents, and standard substitution for the remainder. Intensive post-marketing surveillance is best directed at the medicines in which signals have been detected and at narrow therapeutic index medicines newly entering procurement, rather than applied uniformly to every procured product. Procurement status under this policy is defined at the level of the active ingredient and dosage form rather than the generic name, and different dosage forms of the same ingredient have entered procurement in different rounds, in some cases several years apart: levofloxacin eye drops, tablets and injection entered in the third, fourth and fifth rounds respectively, and voriconazole tablets and injection in the fourth and eighth. Studies that identify procured products by generic name alone may therefore misclassify exposure, and provincial or inter-provincial alliance procurement of the same ingredient can precede or follow the national programme.

### 4.3 Terminological heterogeneity and its consequences for evidence synthesis

The consequences of terminology-based retrieval in this field can be quantified from this review’s own history. The search reported in the original submission was described in two arms but, as executed, retrieved 110 records across four sources; of the 35 studies included in that version, the databases contributed nine and the remaining 26 were identified by citation searching of a review and of the included studies. Three quarters of the evidence base had therefore not been retrieved by the search at all. Rebuilt across six sources, with a second arm anchored on generic-versus-originator terminology and containing no procurement terms, the search retrieved 2,903 records, from which 72 of the 81 studies now included were identified; citation searching contributed nine. The proportion of included studies that the database search failed to retrieve fell from 74% (26 of 35) to 11% (9 of 81).

The reason is that investigators comparing a procurement-selected generic with its originator frequently frame the work as a conventional pharmacological comparison and never use procurement vocabulary. A search built on policy terminology alone therefore misses eligible studies systematically rather than at random, and the studies it misses are not a random subset: they are disproportionately those framed around the medicine rather than around the policy. Any future review in this field that relies on procurement terminology alone should expect the same deficit.

### 4.4 Variability among generic products of the same medicine

What bioequivalence establishes bears directly on how these findings should be read. Admission to the consistency-evaluation programme, and hence to procurement, requires demonstrated pharmaceutical equivalence together with bioequivalence to the reference product: ordinarily a crossover study in healthy volunteers under fasting conditions, conducted on a single batch of each product, in which the 90% confidence intervals for the test-to-comparator ratios of AUC and Cmax lie within 80 to 125% (3, 4). That design is deliberately narrow. It does not test whether differences in excipients, in manufacturing process, or in batch-to-batch variability during routine commercial production give rise to differences in clinical outcome. Such differences are permitted between a generic and its reference product provided the pharmacokinetic criteria are met, and they are controlled after approval through manufacturing standards and post-market sampling rather than through the bioequivalence study itself. The extrapolation from that study to the patients treated in the studies synthesised here is least secure for narrow therapeutic index medicines, for products whose release characteristics depend on formulation, and for patients whose absorption differs from that of the healthy volunteers in whom equivalence was demonstrated.

This review cannot adjudicate any of these mechanisms. *In vitro* pharmaceutical quality data were not sought as review outcomes (Section 2.2), and neither the batch of the product dispensed nor its excipient composition or manufacturing process was among the variables extracted from the included studies (Section 2.5). A between-group difference observed in these studies could therefore not have been attributed to a specific pharmaceutical cause, and the concordance of null findings cannot be read as evidence that no such cause exists.

Each procurement round admits more than one manufacturer for most medicines, and bioequivalence is established between each generic and the originator rather than among the generics themselves. Two products each bioequivalent to a common reference are not thereby bioequivalent to one another, and the intervals permitted by bioequivalence testing allow a wider separation between two generics than between either and the originator. Almost every study included here compares one named generic product with its comparator, so its findings characterise that product rather than the class of procured generics of that medicine.

Where a study included more than one manufacturer, the results were not uniform. One study of azacitidine analysed the products of two manufacturers separately; their identities are anonymised in the source and could not be established. One multicentre study of three injectable cephalosporins found a difference for two of the three and not for the third. Most directly, the within-patient olanzapine study (84) compared two products that had both won procurement, one in the pilot stage and one in the expansion, and found a significant difference in dose-adjusted serum concentration between them. Reports that pool several manufacturers under a single generic label may therefore average across genuinely different products and mask a difference confined to one of them. Systematic comparison of bioequivalence and real-world performance among the generics admitted to the same round is not currently possible from the published literature and is a distinct research need.

### 4.5 Recommendations supported by the evidence and recommendations arising from its gaps

Recommendations that follow from the evidence assembled here are distinguished below from those that follow from what the evidence does not contain.

Three recommendations rest on the included evidence. For the medicines studied here, the available evidence gives no indication that procured products perform less well than their comparators on efficacy: 60 of 62 efficacy comparisons in the main analysis showed no difference and none carries an assigned direction, while the one comparison in which the reported rates differ rests on a test we performed rather than one the source reported. Given that the certainty of this evidence is very low, this supports continued use; it is not a demonstration of therapeutic equivalence. Second, post-procurement monitoring should be organised around tolerability rather than around therapeutic failure, since every difference identified in the main analysis fell in an adverse-event or laboratory outcome. Third, monitoring should be product-specific rather than class-wide, since the differences that were found attached to individual products of individual medicines and, in one case, distinguished two products that had both won procurement.

Four further recommendations follow from gaps rather than from findings. Targeted real-world safety studies are required for the medicines in which signals were detected — procured moxifloxacin tablets for allergic and gastrointestinal reactions, procured olanzapine for glucose metabolism, procured ceftriaxone in children, and procured atorvastatin for creatine kinase — designed with matched comparison groups and predefined outcomes rather than assembled from routine data after the fact. Patient-reported outcomes are absent from the included evidence and cannot be reconstructed from it, so recommending them is a statement about future research rather than an inference from present findings. Narrow therapeutic index medicines are represented by few studies and require dedicated concentration-controlled evaluation. Comparative evaluation among the several generics admitted to the same procurement round has not been reported and would require a design the current literature does not provide.

### 4.6 Candidate indicators for monitoring the quality performance of generic substitution policies

Four measurable dimensions emerged from the patterns observed across the included studies and could be operationalised by policymakers and regulators using routinely available healthcare data. They are offered as evidence-derived starting points rather than as validated instruments, and each would require expert consultation and pilot testing before use.

The difference between intensive and routine adverse-event detection is the first. Two included studies used active surveillance rather than spontaneous reporting alone, and both detected adverse reactions at rates that passive systems would be unlikely to capture. A provincial active-surveillance study of 13 procured medicines across 39 hospitals, which was excluded from this synthesis for lack of an eligible comparator but is informative as context, reported adverse-reaction rates of 0 to 4.58% under intensive monitoring against substantially fewer signals under routine passive surveillance of the same medicines. The ratio between the two detection rates would indicate whether routine surveillance is capturing clinically important safety information, and a large ratio would argue for expanding active monitoring.

The proportion of procured medicines evaluated by a controlled design is the second. Of the 81 included studies, one used a randomised controlled design and seven allocated participants prospectively; the remainder relied on observational designs. The procurement catalogue now covers several hundred products, whereas the evidence assembled here covers just over 40 active ingredients, so this proportion is presently very low and would serve as an indicator of how far the evidence base has matured.

Coverage of therapeutic drug monitoring for narrow therapeutic index medicines is the third. The concentration difference observed for sertraline, and the difference reported between two procured olanzapine products, both arose in medicines for which concentration measurement is available in routine practice. The proportion of procured narrow therapeutic index medicines for which post-market concentration data exist would indicate whether that capacity is being used.

Coverage of patient-reported outcomes is the fourth. None of the 81 included studies assessed perceived efficacy, treatment satisfaction or quality of life after substitution. Given the documented nocebo effect in generic switching, the proportion of post-market evaluations that include a patient-reported measure would indicate whether the patient perspective is represented in the evidence at all.

### 4.7 Implications for future research

Three research needs are not covered by the recommendations above. Follow-up in the included studies rarely extended beyond 12 months, so the durability of equivalence across the three to 5 years over which a chronic medicine is actually taken remains unexamined. The observational designs that dominate this literature would support stronger causal inference if the analytic methods matched the design, with instrumental-variable and difference-in-differences approaches applied to the policy-period comparisons that currently rest on before-and-after contrasts alone.

The third need follows from the reporting practices of this literature and is the most immediately actionable. Once a sufficient number of studies of the same medicine report effect estimates with confidence intervals, drug-specific meta-analyses should be undertaken. The present synthesis could not be quantitative because 42 of 81 studies report a significance test alone; that constraint, rather than heterogeneity by itself, is what a future synthesis would need to overcome. A related and more basic requirement is that reported statistics should be reconcilable with the data from which they are derived. Six of the 24 studies we re-examined against their source articles failed that test, which is a problem of reporting standards in the field and not of synthesis method.

## 5 Conclusion

This systematic review of 81 studies found that the available evidence, while predominantly observational, does not indicate that medicines procured through China’s NVBP policy perform less well than originator or non-selected alternatives. Across more than 40 active ingredients, 60 of 62 efficacy comparisons and 55 of 65 comparative safety comparisons in the originator-comparator stratum showed no significant difference. Seven safety comparisons showed a difference, four favouring the originator and three favouring the procured product; no direction could be assigned in three further comparisons, in two because significant findings within one cohort pointed in opposite directions and in one because adverse-reaction rates were reported in aggregate across 20 medicines. No efficacy comparison in the main analysis carries an assigned direction. One comparison in which the reported rates differ is excluded from the tally because the source did not test it; the difference rests on a test performed by the review authors and would not survive correction for multiplicity.

The GRADE certainty of this evidence is very low for efficacy, comparative safety and pharmacokinetics. That rating is not a formality: an effect estimate was unavailable for 42 of the 81 studies, 62 were single-centre, 21 began recruitment more than a year before the medicine entered procurement, 23 arise from five research groups, and six report statistics irreconcilable with their own data. The concordance of null findings therefore provides cautious reassurance that the price reductions achieved by this policy have not come at an evident cost to clinical quality; it does not demonstrate equivalence, and it should not be cited as though it did.

Three actions follow. Procured moxifloxacin tablets, procured olanzapine, procured ceftriaxone in children and procured atorvastatin should be the subject of prospective real-world safety studies with matched comparison groups and predefined adverse-event outcomes. Post-procurement surveillance should record the manufacturer and, where feasible, the batch of the dispensed product, since the evidence characterises individual products rather than classes, and one included study found a significant difference between two products that had both won procurement. Future evaluations should report effect estimates with confidence intervals rather than significance tests alone, and should report statistics that can be reconciled with their own data; these are the two changes that would allow the next synthesis in this field to be quantitative.

## Data Availability

The original contributions presented in the study are included in the article/Supplementary material, further inquiries can be directed to the corresponding author.

## Supplementary material

Supplementary material 1 PROSPERO registration record CRD420261303351, reproduced in full.

Supplementary Table S1 Complete search strategy for each source and search arm, the record flow, and the reports identified by citation searching.

Supplementary Table S2 Risk-of-bias assessment of all 81 included studies across eight domains.

Supplementary Table S3 Outcomes and effect estimates of every included study.

Supplementary Tables S4,S7 GRADE evidence profile (S4) and the eleven sensitivity analyses (S7).

Supplementary Table S5 Completed PRISMA 2020 checklist; item 24c lists every amendment made after registration.

Supplementary Table S6 Full-text eligibility decision for all 862 reports assessed.
