# Supplementary material for: Clinical efficacy and safety of medicines procured through China’s national volume-based procurement policy: a systematic review of 81 studies
Source: Front Public Health. 2026 Oct 15;14:1820505. doi: 10.3389/fpubh.2026.1820505 (PMC13716852; doi:10.3389/fpubh.2026.1820505)
Supplement: Supplementary file 1 [file Data_Sheet_1.pdf]

NIHR | National Institute for Health and Care Research

PROSPERO
International prospective register of systematic reviews

TRAINING

Quality, Efficacy, and Safety of Medicines Procured Through China's National Volume-Based Procurement Policy: A Systematic Review

Lele Zhang, Youjie Zhang

REVIEW TITLE AND BASIC DETAILS

Review title

Quality, Efficacy, and Safety of Medicines Procured Through China's National Volume-Based Procurement Policy: A Systematic Review

Condition or domain being studied

Pharmaceutical policy; Quality and safety ; Healthcare policy

This review focuses on China's National Volume-Based Procurement (NVBP) policy, a centralized drug procurement reform initiated in 2018 that mandates large-scale substitution of originator drugs with competitively priced generic alternatives. We aim to systematically evaluate the impact of this policy-driven generic substitution on therapeutic equivalence, clinical effectiveness, and safety outcomes in real-world clinical settings.

Rationale for the review

China's National Volume-Based Procurement (NVBP) policy, launched in 2018, represents one of the world's largest centralized drug procurement reforms, covering 491 generic drug products across the first eleven procurement rounds. While the policy has achieved substantial price reductions (averaging 50-60%), concerns have been raised by clinicians, patients, and regulators regarding whether the quality and therapeutic equivalence of procured generic drugs are maintained after large-scale substitution. Several individual studies have examined post-NVBP changes in clinical outcomes, adverse drug reactions and bioequivalence data, but findings are inconsistent and fragmented across different therapeutic areas and study designs. To date, no systematic review has comprehensively synthesized the available evidence on the quality and efficacy implications of NVBP-driven generic substitution. This review will fill this gap by systematically identifying, appraising, and synthesizing all relevant studies, providing an evidence-based assessment of whether current quality assurance mechanisms under NVBP are adequate. The findings will inform ongoing policy refinements and contribute to the international discourse on balancing drug affordability with quality assurance in centralized procurement systems.

Review objectives

This systematic review aims to: (1) Systematically identify and synthesize all available evidence on the impact of China's National Volume-Based Procurement (NVBP) policy on the quality and therapeutic equivalence of procured generic drugs; (2) Assess whether NVBP-driven generic substitution is associated with changes in clinical effectiveness, adverse drug reaction profiles, pharmacokinetic measures, or patient-reported outcomes compared with pre-procurement periods or originator drugs; (3) Evaluate the methodological quality and risk of bias of included studies; (4) Identify gaps in the current evidence base and areas requiring further investigation. The overarching question is: Does the large-scale generic substitution mandated by China's NVBP policy maintain equivalent drug quality and therapeutic outcomes in real-world clinical practice?

Keywords

volume-based procurement; Generic substitution; Drug quality; Therapeutic equivalence; Generic drugs

Country

China

ELIGIBILITY CRITERIA

Population

Included
Studies involving patients who received generic drugs procured through China's National Volume-Based Procurement (NVBP) policy, across any therapeutic area or disease condition. Eligible study populations include patients of any age who were prescribed or administered NVBP-procured generic medications in Chinese healthcare settings (hospitals, community health centers, or pharmacies).

Excluded
Studies exclusively focused on drug pricing, cost savings, or procurement logistics without any assessment of therapeutic outcomes. Studies conducted outside of mainland China or examining generic substitution unrelated to the NVBP policy. Animal studies, in vitro studies, editorials, commentaries, conference abstracts, and non-peer-reviewed publications.

Intervention(s) or exposure(s)

Included
Pharmaceutical policy; Healthcare policy

The intervention or exposure of interest is generic drug substitution driven by China's National Volume-Based Procurement (NVBP) policy, implemented from 2018 onwards. Eligibility is determined by the substance of the medicine evaluated rather than by whether the source article uses procurement terminology. A study is eligible where the active ingredient and dosage form under investigation appear in the official list of products procured in a completed NVBP round; that list covers the first eleven rounds and 491 products and is taken from the results notice of each round. Dosage-form equivalence is judged against the procurement unit used in the national list, such as "solid oral immediate-release form", rather than against the pharmacopoeial dosage form. A study is eligible on timing where its recruitment window overlaps, by at least one month, the period following the announcement of the results of the relevant round.

Excluded
Studies examining voluntary generic substitution unrelated to the NVBP policy mandate. Studies focused solely on traditional Chinese medicine or on biological products not covered by the NVBP catalogue. Studies reporting only in vitro pharmaceutical quality data, such as dissolution, impurity profiles or content uniformity, without a clinical, safety or pharmacokinetic outcome.

Comparator(s) or control(s)

Included
PICO tags selected: Usual Care

Three comparator types are eligible: the originator product, including imported originator preparations; the same medicine at the same institution before the policy took effect; and a non-selected generic of the same active ingredient and dosage form. These are analysed as separate strata. The originator-comparator stratum constitutes the main analysis; comparisons against a pre-policy baseline or a non-selected generic are reported separately and are not pooled with it, because equivalence to another generic does not support inference of equivalence to the reference product. A study without an eligible comparator is excluded. Where the official supply list shows that the originator was itself among the winning suppliers of that round, the comparison is identified as being between a selected generic and a selected originator.

Excluded
Studies with no comparator group of any of the three eligible types. Where both arms are procurement-selected products, the comparison is reported separately as a within-policy substitution and does not enter the originator-comparator synthesis; it is not treated as evidence of equivalence to the reference product.

Study design
Both randomized and nonrandomized study types will be included.

Included
This review will include all empirical study designs: randomised controlled trials, non-randomised controlled studies, cohort studies (prospective and retrospective), before-and-after studies, and cross-sectional studies. No restrictions will be placed on study design to ensure comprehensive capture of all available evidence, given the emerging nature of this research area.

Excluded
Editorials, commentaries, opinion pieces, narrative reviews, conference abstracts, case reports involving a single patient, letters to the editor without original data, and non-peer-reviewed publications such as news articles and government reports without primary data. Bioequivalence studies conducted in healthy volunteers are excluded, as their population and outcomes do not meet the review's PICO.

Context
Studies conducted in mainland China healthcare settings, including tertiary hospitals, secondary hospitals, primary care

institutions, and community pharmacies. The review is restricted to the period from 2018 onwards, corresponding to the initiation of China's National Volume-Based Procurement (NVBP) policy. Studies must be set within the context of NVBP policy implementation, examining drugs included in any of the eleven completed procurement rounds. No restrictions on patient age, sex, or disease condition will be applied.

TIMELINE OF THE REVIEW

Date of first submission to PROSPERO
09 February 2026

Review timeline 1 change
Review start date: 7 February 2026. Review end date: 8 September 2026.

Date of registration in PROSPERO
09 February 2026

AVAILABILITY OF FULL PROTOCOL

Availability of full protocol
A full protocol has not been written.

SEARCHING AND SCREENING

Search for unpublished studies
Only published studies will be sought.

Main sources that will be searched
The main sources to be searched are *CENTRAL - Cochrane Central Register of Controlled Trials*, *CNKI - China National Knowledge Infrastructure database* and *PubMed*.

Other sources that will be searched
Web of Science Core Collection; Wanfang Data; VIP (CQVIP)

Search language restrictions
There are no language restrictions.

Search date restrictions
Databases will be searched for articles published from 1 January 2018 and before by 31 January 2026.

Other methods of identifying studies
Other studies will be identified by: *reference list checking (backward citation searching)*.

Additional information about identifying studies
Reference list checking identified seven reports that the database searches did not independently retrieve; all seven met the eligibility criteria and are included. They enter the review through the other-methods route of the PRISMA flow diagram.

Link to search strategy
A full search strategy has been uploaded to PROSPERO. The PDF may be accessed through this link https://www.crd.york.ac.uk/PROSPEROFILES/b95dfd58d4898ba1fd91303d3caf348e.pdf.

Selection process
Titles and abstracts were screened by one reviewer and checked by a second, as specified at registration. All reports reaching full-text assessment were screened independently and in duplicate by two reviewers, with disagreements resolved by discussion.

Other relevant information about searching and screening
Records from all sources are deduplicated by matching DOI and standardised title. Titles and abstracts are screened by one reviewer and checked by a second, as specified at registration, with disagreements resolved by discussion; records judged potentially eligible or uncertain by either reviewer proceed to full-text assessment. The reliability of that stage was assessed post hoc in a random sample of 500 of the 1,876 deduplicated records, re-screened independently and in duplicate blind to the original decision: raw agreement 89.2%, Cohen kappa 0.78 (95% CI 0.73 to 0.84). All 860 reports reaching full-text assessment are screened independently and in duplicate against a fixed sequence of eight questions, applied in a set order

so that each excluded report carries the reason encountered first: whether the medicine or dosage form belongs to a completed procurement round; whether the study period precedes the announcement of that round; whether an eligible comparator is present; whether a clinical, safety or pharmacokinetic outcome is reported; whether the report is original research; whether it duplicates an included study; whether the full text is obtainable; and whether the publication date falls within the search window. Disagreements arose for 18 reports and were resolved by discussion. Of the excluded records, 400 of 781 were re-assessed independently against the full text; judgements agreed in 393 cases and the seven discrepancies were resolved without change. Records could not be exported from Cochrane CENTRAL and did not enter deduplication. Embase could not be searched because institutional access was unavailable.

DATA COLLECTION PROCESS

Data extraction from published articles and reports

Data will be extracted by one person (or a machine) and checked by at least one other person.

Authors will be asked to provide any required data not available in published reports.

Study risk of bias or quality assessment

Risk of bias will be assessed using: *Cochrane RoB-2*, *Newcastle-Ottawa* and *ROBINS-I*

Institute of Health Economics (IHE) checklist for case series. Risk of bias is assessed in eight domains: five correspond to ROBINS-I domains, and three are specific to this review - exposure misclassification, comparator identifiability, and independence of data sources.

Data will be assessed by one person (or a machine) and checked by at least one other person.

Additional information will be sought from study investigators if required information is unclear or unavailable in the study publications/reports.

Reporting bias assessment

Risk of bias due to missing results is assessed qualitatively within GRADE. Funnel plots are not used because no outcome is reported as a commensurable estimate by enough studies. Prospective registration of included studies, concentration of studies within single research platforms, and comparisons reported but not tested are all considered.

Certainty assessment

Certainty of evidence is assessed using the GRADE approach for each outcome domain: therapeutic efficacy, comparative safety, pharmacokinetics and economic outcomes. Randomised evidence starts at high certainty and non-randomised evidence at low certainty. Domains are rated down for risk of bias, inconsistency, indirectness, imprecision and publication bias, with the rationale for each judgement recorded. Certainty is expressed as high, moderate, low or very low, and the full evidence profile is presented in a supplementary GRADE table.

OUTCOMES TO BE ANALYSED

Main outcomes

The main outcomes are: (1) Therapeutic efficacy - disease control, treatment response, symptom improvement or other clinical efficacy indicators as reported by the source, at the time points it reports; (2) Comparative safety - incidence of adverse drug reactions, adverse events and laboratory abnormality, compared between arms; (3) Pharmacokinetics - steady-state serum or plasma concentration of the medicine, where measured; (4) Patient-reported outcomes - satisfaction, adherence and quality of life, where reported. The standardised effect measure for synthesis is the direction of effect for each study and outcome domain, recorded as favouring the procurement-selected generic, favouring the comparator, or no statistically significant difference, because the effect measures reported across studies are not commensurable. Where a source reports event counts or summary statistics, a risk ratio or a mean difference with its confidence interval is calculated and reported alongside. No meta-analysis is planned. In vitro pharmaceutical quality data, such as dissolution, impurity profiles and content uniformity, are not sought as review outcomes; results of national regulatory sampling are summarised as contextual background only.

Additional outcomes

Additional outcomes include: (1) Economic outcomes - acquisition cost, total treatment cost, and cost-effectiveness or cost-minimisation results reported in the included studies; (2) Healthcare resource utilisation - hospitalisation, outpatient attendance, length of stay and readmission; (3) Medication use measures - prescribed daily dose, defined daily doses, treatment persistence, switching and re-switching rates. Measures of use are recorded for each study but do not determine the direction of effect in any outcome domain, because they reflect prescribing and procurement practice rather than the clinical performance of the medicine.

PLANNED DATA SYNTHESIS

Strategy for data synthesis

Results are synthesised without meta-analysis, following the SWiM reporting guideline. Studies are first allocated to comparator strata: the originator product, a pre-policy baseline at the same institution, a non-selected generic of the same ingredient and dosage form, and within-patient substitution. The originator-comparator stratum constitutes the main analysis; the other strata are reported separately and are not pooled with it, because equivalence to another generic does not support inference of equivalence to the reference product.

Within the main stratum, studies are allocated to outcome domains and, within each domain, stratified by therapeutic area and drug class. The direction of effect for each study and domain is recorded as favouring the procurement-selected generic, favouring the comparator, or no statistically significant difference, and summarised in effect-direction tables and a harvest plot. In the harvest plot each study is one bar of uniform height, shaded by risk of bias; bar height does not encode sample size, because no weighting is applied. Where significant differences within one cohort point in opposite directions, no direction is assigned. An outcome reported without an identifiable denominator does not determine direction.

Where a source reports group sizes and outcome percentages for an eligible comparison but does not test it, frequencies are reconstructed and chi-square tests applied by the review authors; such analyses are identified as post hoc.

Ten sensitivity analyses are conducted. Two were specified in the original submission: exclusion of studies at high risk of bias, implemented as exclusion of any study rated high in any of the eight domains; and restriction to multicentre studies. Eight were added during the revision: restriction to the originator-comparator stratum; exclusion of studies at high risk of exposure misclassification; exclusion of studies whose arms are drawn from non-overlapping calendar windows; exclusion of studies without matching or adequate adjustment; exclusion of the study extracted from an abstract only; exclusion of studies with incomplete or irreconcilable outcome reporting; retention of a single study per research platform; and exclusion of comparisons tested only by the review authors.

CURRENT REVIEW STAGE

Stage of the review at this submission 1 change

Review stage	Started	Completed
Pilot work	✓	✓
Formal searching/study identification	✓	✓
Screening search results against inclusion criteria	✓	✓
Data extraction or receipt of IPD	✓	✓
Risk of bias/quality assessment	✓	✓
Data synthesis	✓	✓

Review status

The review is completed.

Publication of review results

Results of the review will be published in English.

REVIEW AFFILIATION, FUNDING AND PEER REVIEW

Review team members

Dr Lele Zhang (review guarantor and contact) China Pharmaceutical University. China.

No conflict of interest declared.

Mr Youjie Zhang. China Pharmaceutical University. China.

No conflict of interest declared.

Named contact

Dr Lele Zhang (cpuzll@126.com). China Pharmaceutical University. China.

Review affiliation
School of International Pharmaceutical Business, China Pharmaceutical University, Nanjing, Jiangsu, China

Funding source
Review has no specific/external funding but is supported by guarantor/review team (non-commercial) institutions.

Peer review
There has been no peer review of this planned review.

ADDITIONAL INFORMATION

Additional information
This review will be reported following the Preferred Reporting Items for Systematic Reviews and Meta-Analyses (PRISMA) 2020 guidelines. The completed PRISMA checklist will be submitted as supplementary material with the final manuscript.

Review conflict of interest
Declared individual interests are recorded under team member details.. No additional interests are recorded for this review.

Medical Subject Headings
China; Drug Substitution; Drug-Related Side Effects and Adverse Reactions; Drugs, Generic; Humans; Patient Reported Outcome Measures; Policy; Therapeutic Equivalency; Treatment Outcome

Revision note 1 change
This amendment corrects four figures and one statement recorded in Version 3.0; it changes no eligibility criterion, outcome, synthesis method or screening procedure. The number of reports reaching full-text assessment is 862, not 860. The number of reports identified by reference list checking is nine, not seven; all nine met the eligibility criteria and are included. The number of sensitivity analyses is eleven, not ten: two were specified in the original submission and nine were added during the revision. The ninth addition, assignment of a direction wherever any outcome of a study's principal analysis differed significantly rather than by the primary outcome alone, was specified in the manuscript but omitted from the list in Version 3.0. The statement in Version 3.0 that the review title had been aligned with the title of the manuscript is corrected. The record title retains the word "Quality" from the original registration, whereas the manuscript is titled "Clinical Efficacy and Safety of Medicines Procured Through China's National Volume-Based Procurement Policy: A Systematic Review of 81 Studies". In vitro pharmaceutical quality data were removed from the review outcomes during the revision, as recorded in Version 3.0; the record title will be aligned at the next major revision. The review status and end date are updated to reflect completion of all review stages.

SIMILAR REVIEWS

Check for similar records already in PROSPERO
PROSPERO identified a number of existing PROSPERO records that were similar to this one (last check made on 7 February 2026). These are shown below along with the reasons given by that the review team for the reviews being different and/or proceeding.

- Generic vs. Brand Medications: Are Patient Health Outcomes Affected? Evidence from Clinical Trials and Pharmacoepidemiologic Studies: A Systematic Review [published 10 October 2021] [CRD42021277689]. The review was judged **not to be similar**
- The Impact of Centralized Procurement on Medical Consumables: A Systematic Review [published 21 March 2025] [CRD420251015908]. The review was judged **not to be similar**
- Clinical Pharmacogenomic Variants Among the Saudi Population and Their Impact on Drug Response: A Review of Saudi-Based Evidence [published 17 November 2025] [CRD420251232518]. The review was judged **not to be similar**

PROSPERO version history 1 change
- Version 3.1, published 15 Sep 2026
- Version 3.0, published 14 Sep 2026
- Version 2.0, published 11 Sep 2026
- Version 1.0, published 09 Feb 2026

Disclaimer
The content of this record displays the information provided by the review team. PROSPERO does not peer review

registration records or endorse their content.

PROSPERO accepts and posts the information provided in good faith; responsibility for record content rests with the review team. The guarantor for this record has affirmed that the information provided is truthful and that they understand that deliberate provision of inaccurate information may be construed as scientific misconduct.

PROSPERO does not accept any liability for the content provided in this record or for its use. Readers use the information provided in this record at their own risk.

Any enquiries about the record should be referred to the named review contact
