# Supplementary material for: Clinical efficacy and safety of medicines procured through China’s national volume-based procurement policy: a systematic review of 81 studies
Source: Front Public Health. 2026 Oct 15;14:1820505. doi: 10.3389/fpubh.2026.1820505 (PMC13716852; doi:10.3389/fpubh.2026.1820505)
Supplement: Supplementary file 2 [file Table_1.docx]

**Supplementary Table S1. Complete search strategy by source and search arm, record flow, and reports identified by citation searching.**

**Part A. Search strings as executed**

| **Source** | **Arm** | **Date searched** | **Records** | **Search string as executed** |
| --- | --- | --- | --- | --- |
| CNKI | 1 | 2026-08-09 | 283 | 主题=("带量采购" OR "集中采购" OR "集采" OR "4+7" OR "国家组织药品集中采购" OR "中选") AND 主题=("疗效" OR "有效性" OR "安全性" OR "不良反应" OR "临床" OR "血药浓度") |
| CNKI | 2 | 2026-08-09 | 116 | 主题=("仿制药" OR "原研" OR "一致性评价" OR "通用名药") AND 主题=("疗效" OR "有效性" OR "安全性" OR "不良反应" OR "血药浓度") |
| Wanfang Data | 1 | 2026-08-09 | 793 | 主题:("带量采购" OR "集中采购" OR "集采" OR "4+7" OR "国家组织药品集中采购" OR "中选") AND 主题:("疗效" OR "有效性" OR "安全性" OR "不良反应" OR "临床" OR "血药浓度") |
| Wanfang Data | 2 | 2026-08-09 | 152 | 主题:("仿制药" OR "原研" OR "一致性评价") AND 主题:("疗效" OR "安全性" OR "不良反应" OR "血药浓度") |
| VIP | 1 | 2026-08-09 | 131 | 题名或关键词=("带量采购" OR "集中采购" OR "集采" OR "中选") AND 题名或关键词=("疗效" OR "安全性" OR "不良反应") |
| VIP | 2 | 2026-08-09 | 58 | 题名或关键词=("仿制药" OR "原研") AND 题名或关键词=("疗效" OR "安全性" OR "不良反应") |
| PubMed | 1 | 2026-09-08 | 64 | ("volume-based procurement"[tiab] OR "volume based procurement"[tiab] OR "centralized drug procurement"[tiab] OR "centralised drug procurement"[tiab] OR "centralized procurement"[tiab] OR "national centralized drug procurement"[tiab] OR "national centralized drug purchasing"[tiab] OR "national organized drug procurement"[tiab] OR "4+7 cities"[tiab] OR "4+7 pilot"[tiab] OR NVBP[tiab] OR NCDP[tiab]) AND (efficacy[tiab] OR effectiveness[tiab] OR safety[tiab] OR adverse[tiab] OR "clinical outcome"[tiab] OR "clinical outcomes"[tiab] OR "therapeutic equivalence"[tiab] OR "generic substitution"[tiab] OR bioequivalence[tiab] OR "drug quality"[tiab]) AND ("2018/01/01"[dp] : "2026/01/31"[dp]) |
| PubMed | 2 | 2026-09-08 | 496 | (("generic drug"[tiab] OR "generic drugs"[tiab] OR "generic substitution"[tiab] OR "generic medicine"[tiab] OR "generic medicines"[tiab] OR "generic version"[tiab] OR originator[tiab] OR "brand-name"[tiab] OR "branded drug"[tiab] OR "branded drugs"[tiab] OR bioequivalence[tiab] OR "consistency evaluation"[tiab]) AND (efficacy[tiab] OR effectiveness[tiab] OR safety[tiab] OR "adverse drug reaction"[tiab] OR "adverse drug reactions"[tiab] OR "adverse event"[tiab] OR "adverse events"[tiab] OR "clinical outcome"[tiab] OR "clinical outcomes"[tiab] OR "therapeutic equivalence"[tiab] OR "plasma concentration"[tiab] OR "plasma concentrations"[tiab]) AND (China[tiab] OR Chinese[tiab] OR "China"[MeSH Terms])) AND ("2018/01/01"[dp] : "2026/01/31"[dp]) |
| Web of Science | 1 | 2026-09-08 | 79 | TS=("volume-based procurement" OR "volume based procurement" OR "centralized drug procurement" OR "centralised drug procurement" OR "centralized procurement" OR "national centralized drug procurement" OR "national centralized drug purchasing" OR "national organized drug procurement" OR "4+7 cities" OR "4+7 pilot" OR NVBP OR NCDP) AND TS=(efficacy OR effectiveness OR safety OR adverse OR "clinical outcome*" OR "therapeutic equivalence" OR "generic substitution" OR bioequivalence OR "drug quality") |
| Web of Science | 2 | 2026-09-08 | 731 | TS=("generic drug*" OR "generic substitution" OR "generic medicine*" OR "generic version*" OR originator OR "brand-name" OR "branded drug*" OR bioequivalence OR "consistency evaluation") AND TS=(efficacy OR effectiveness OR safety OR "adverse drug reaction*" OR "adverse event*" OR "clinical outcome*" OR "therapeutic equivalence" OR "plasma concentration*") AND TS=(China OR Chinese) |
| Cochrane CENTRAL | 1 | 2026-09-08 | 6 | "volume-based procurement" OR "centralized drug procurement" OR "national centralized drug procurement" OR "4+7 cities" OR NVBP in Title Abstract Keyword; Publication Year 2018–2026; Trials |
| Cochrane CENTRAL | 2 | 2026-09-08 | 1,281 | ("generic drug" OR "generic substitution" OR "generic medicine" OR "generic version" OR originator OR "brand-name" OR "branded drug" OR bioequivalence OR "consistency evaluation") AND (efficacy OR effectiveness OR safety OR "adverse drug reaction" OR "adverse event" OR "clinical outcome" OR "therapeutic equivalence" OR "plasma concentration") AND (China OR Chinese) in Title Abstract Keyword; Publication Year 2018–2026; Trials |

Two search arms were used at every source. Arm 1 combined procurement terminology with clinical outcome terms. Arm 2 was anchored on generic-versus-originator terminology, without procurement terms, in order to retrieve eligible studies that evaluate NVBP-selected products without using procurement wording. No language restriction was applied. Records published outside 1 January 2018 to 31 January 2026 were excluded at screening on the basis of the publication year recorded in the bibliographic record. For PubMed, the phrases "national organized drug procurement" and "4+7 cities" are not present in the PubMed phrase index and were processed as individual terms; this is reported here because it affects reproducibility. For Cochrane CENTRAL, hit counts were taken from the results page; bibliographic records could not be exported because full CENTRAL content requires a subscription, and CENTRAL records therefore did not enter deduplication or screening. Embase was listed as an information source at registration but could not be searched because institutional access was unavailable (Supplementary Table S5, item 24c).

**Part B. Records by source and arm**

| **Source** | **Arm 1** | **Arm 2** | **Total** |
| --- | --- | --- | --- |
| CNKI | 283 | 116 | 399 |
| Wanfang Data | 793 | 152 | 945 |
| VIP | 131 | 58 | 189 |
| PubMed | 64 | 496 | 560 |
| Web of Science | 79 | 731 | 810 |
| Total entering deduplication | 1,350 | 1,553 | 2,903 |
| Cochrane CENTRAL (hit counts only; not deduplicated or screened) | 6 | 1,281 | 1,287 |

**Part C. Record flow (these figures correspond exactly to Figure 1)**

| **Stage** | **n** |
| --- | --- |
| Records identified from the five exportable sources, both arms | 2,903 |
| Duplicate records removed before screening | 1,027 |
| Records screened at title and abstract | 1,876 |
| Records excluded at title and abstract | 1,023 |
| Reports sought for retrieval, database route | 853 |
| Reports not retrieved | 0 |
| Reports identified by citation searching, other-methods route | 9 |
| Reports assessed for eligibility in full text, both routes | 862 |
| Reports excluded at full text | 781 |
| Studies awaiting classification | 0 |
| Studies included in the review | 81 |

**Part D. Identification via other methods**

Reference lists of the included studies, of one broad review of the field, and of the version of this review submitted for peer review were searched. Nine reports were identified in this way that the database searches did not independently retrieve; all nine met the eligibility criteria and are among the 81 included studies. They enter the review through the other-methods route shown in Figure 1. For comparison, the search reported in the original submission retrieved 110 records across four sources and citation searching contributed 26 of the 35 studies included in that version; under the rebuilt strategy the database route contributes 72 of the 81 included studies and citation searching nine.

| **ID** | **Authors** | **Title** | **Source** | **DOI** |
| --- | --- | --- | --- | --- |
| X1 | 彭倩雯, 徐斌, 张伟霞 | 急性冠脉综合征患者原研与"带量采购"阿托伐他汀钙片疗效和安全性的回顾性比较 | 药物流行病学杂志 2023;32(4):361-366 | 10.19960/j.issn.1005-0698.202304002 |
| X2 | 袁倩倩, 马月静, 刘莹, 张相林, 覃旺军 | 基于真实世界的集中带量采购品种注射用头孢呑辛钠预防剖孫产术后感染的临床再评价 | 中国医院用药评价与分析 2024;24(3):275-277 | 10.14009/j.issn.1672-2124.2024.03.005 |
| X3 | 王琲 等 | 利奈唑胺片仿制药与原研药有效性和安全性比较的真实世界研究 | 中国临床药学杂志 2023;32(1):18-22 | 10.19577/j.1007-4406.2023.01.004 |
| X4 | 吴义来, 何银梅, 余建国, 栏家杰 | 基于真实世界数据的阿托伐他汀钙片集采品种短期降脂效果、安全性及经济性评价 | 齐齐哈尔医学院学报 2024;45(18):1707-1712 | 10.3969/j.issn.1002-1256.2024.18.002 |
| X5 | 李一辰, 伊曼曼, 邵腾飞 等 | 集中带量采购替格瑞洛仿制药与原研药用于行支架辅助弹簧圈栓塞术颅内动脉瘤患者的有效性及安全性比较 | 中国临床药学杂志 2024;33(9):661-666 | 10.19577/j.1007-4406.2024.09.004 |
| X6 | Si X, Huang L, Ding Q, et al. | Comparison of clinical and economic evaluation between selected generic and original febuxostat tablets in Chinese gout patients with hyperuricemia | Medicine 2024;103(4):e37081 | 10.1097/MD.0000000000037081 |
| X7 | Xing X, Wang Z, Wang K, et al. | Comparison of treatment patterns, efficacy and safety between generic and branded atorvastatin users in China | Lipids in Health and Disease 2025;24:248 | 10.1186/s12944-025-02673-9 |
| X8 | 罗巧, 王之舟, 刘文娜, 崔晓辉, 董宪喆, 张兰 | 格列美脲集采中选仿制药与原研药临床效果的真实世界研究 | 中国药业 2023;32(24):50-54 | 10.3969/j.issn.1006-4931.2023.24.012 |
| X9 | 李玲, 管瀛芸, 于平, 齐艳霞, 马培志, 张文, 栾家杰, 卞晓岚 | 基于倾向性评分匹配法评价格列美脲仿制药和原研药的疗效与安全性 | 中国临床药学杂志 2023;32(1):8-12 | 10.19577/j.1007-4406.2023.01.002 |
