# Supplementary material for: Clinical efficacy and safety of medicines procured through China’s national volume-based procurement policy: a systematic review of 81 studies
Source: Front Public Health. 2026 Oct 15;14:1820505. doi: 10.3389/fpubh.2026.1820505 (PMC13716852; doi:10.3389/fpubh.2026.1820505)
Supplement: Supplementary file 3 [file Table_2.docx]

**Supplementary Table S2. Risk of bias in the 81 included studies, assessed in eight domains.**

Five domains correspond to those of ROBINS-I and were applied through the instrument appropriate to each design. Three are specific to this policy setting and are not covered by any existing instrument: D1 exposure misclassification, D3 comparator identifiability and D7 independence of data sources. D8 records the source of the extracted data.

D1 exposure misclassification: low, the recruitment window lies wholly after the announcement of the relevant round; moderate, it opens within 12 months before the announcement; high, it opens more than 12 months before the announcement or overlaps the post-announcement period by less than three months; indeterminate, the window is not reported or the medicines span several rounds. D2 secular bias: high where the arms are drawn from non-overlapping calendar periods. D3 comparator identifiability: low where the comparator is an originator identified by manufacturer or brand; moderate where it is an originator but the product is not named; high where it is a non-selected generic that is not named. D4 confounding control: low for randomisation, propensity score matching or weighting; moderate for multivariable adjustment; high where neither was applied and the arms differed at baseline. D5 co-intervention and centre effect: high where a systematic difference other than the medicine exists. D6 outcome reporting: high where one outcome domain is wholly unreported, where only within-arm comparison is made, where no events occurred, or where the reported result cannot be reconciled with the study’s own data; moderate where denominators or subgroup sizes are not reported. D7 records whether the study belongs to a research group or platform that contributed other included studies. D8 records whether extraction was from the full text or from an abstract only.

| **No.** | **Study and medicine** | **D1** | **Basis for D1** | **D2** | **D3** | **D4** | **D5** | **D6** | **D7** | **D8** | **Notes from full-text verification** |
| --- | --- | --- | --- | --- | --- | --- | --- | --- | --- | --- | --- |
| 26 | Yang 2024 [26] — Atorvastatin | Low | Study period lies wholly after the announcement of the round | High | Low | Low | Low | Low | — | Full text | — |
| 38 | Guo 2023 [38] — Sertraline | Low | Study period lies wholly after the announcement of the round | Low | Low | Low | Low | High | Yes | Full text | — |
| 72 | Sun 2024 [72] — Cefuroxime injection | Moderate | Exposure misclassification window 8 months (study period 2020-11 to 2022-11; round five announced 2021-06-28) | Low | Low | Low | Low | Low | Yes | Full text | — |
| 73 | Luo 2021 [73] — Six psychiatric medicines | Low | Study period lies wholly after the announcement of the round | High | Low | Low | Low | Low | — | Full text | — |
| 75 | Cai 2024 [75] — Levofloxacin sodium chloride injection | Moderate | Exposure misclassification window 8 months | Low | Low | Low | Low | Low | Yes | Full text | — |
| 78 | Xue 2024 [78] — Amlodipine besylate | Low | Study period lies wholly after the announcement of the round | Low | Low | Low | High | Low | — | Full text | — |
| 82 | Li 2026 [82] — Voriconazole tablets | High | Exposure misclassification window 2 years | Low | Low | Low | Low | Low | — | Full text | — |
| 83 | Li 2025 [83] — Saxagliptin | Low | Study period lies wholly after the announcement of the round | Low | Low | Low | Low | Low | — | Full text | — |
| 84 | Yang 2023 [84] — Ceftriaxone injection | Low | Study period lies wholly after the announcement of the round | High | Low | Low | Low | Low | — | Full text | — |
| 89 | Song 2024 [89] — Ceftriaxone injection | High | Exposure misclassification window 17 months (study period 2020-01 to 2022-09; round five announced 2021-06-28) | Low | Low | Low | Low | Moderate | Yes | Full text | The comparator is the named originator (Rocephin, Shanghai Roche) P values throughout the safety table are not reproducible from the printed counts; two of three comparisons reported as significant are not significant on recalculation |
| 96 | Li 2025 [96] — Caspofungin acetate injection | High | Exposure misclassification window nearly 4 years | Low | Low | Low | Low | Low | — | Full text | — |
| 97 | Gao 2026 [97] — Meropenem injection | Low | Study period lies wholly after the announcement of the round | High | Low | Low | Low | Low | — | Full text | — |
| 102 | Li 2025 [102] — Olanzapine tablets | Low | Study period lies wholly after the announcement of the round | Low | Low | High | Low | High | — | Full text | A between-group efficacy endpoint is reported. Graded high for selection of the reported result: every reported pairing of t and P is arithmetically impossible and the abstract contradicts the Results section |
| 103 | Yang 2024 [103] — Oxaliplatin injection | Low | Study period lies wholly after the announcement of the round | Low | Moderate | High | Low | High | — | Full text | — |
| 104 | Qian 2024 [104] — Omeprazole | Low | Study period lies wholly after the announcement of the round | Low | Low | Low | Low | Low | — | Full text | — |
| 107 | Jia 2022 [107] — Paroxetine tablets | Low | Study period lies wholly after the announcement of the round | High | Low | Low | Low | High | Yes | Full text | — |
| 112 | Zhao 2022 [112] — Pantoprazole | Moderate | Exposure misclassification window 1 month | Low | Moderate | Low | Low | Low | — | Full text | — |
| 115 | Zhang 2022 [115] — Amlodipine besylate; irbesartan | Low | Study period lies wholly after the announcement of the round | Low | Moderate | Low | Low | High | — | Full text | — |
| 119 | Wang 2024 [119] — Empagliflozin | Low | Study period lies wholly after the announcement of the round | Low | Low | High | Low | High | — | Full text | No matching or adjustment; comorbidity and concomitant heart-failure therapy differed between arms No tested comparative safety outcome; reported HbA1c P values are inconsistent with the study’s own test statistics; the significant renal index is defined inconsistently between text and table |
| 142 | Wang 2023 [142] — Tegafur | Moderate | Exposure misclassification window about 1 year | Low | Low | Low | Low | Low | Yes | Full text | — |
| 143 | Miao 2024 [143] — Ticagrelor tablets | Low | Study period lies wholly after the announcement of the round | Low | Low | Low | Low | Low | — | Full text | — |
| 144 | Chen 2023 [144] — 20 medicines in 10 groups (multiple rounds) | Indeterminate | Aggregated across rounds (20 representative medicines belonging to different rounds), so no single announcement date is available for comparison; study period recorded as 2020 to 2022 | Low | Low | Low | Low | High | — | Full text | Adverse reaction rates reported in aggregate across twenty medicines; neither the medicines that differed nor any denominator is given |
| 145 | Hong 2025 [145] — Atorvastatin; capecitabine; pregabalin; budesonide | Low | Study period lies wholly after the announcement of the round | Low | Low | Low | Low | Low | — | Full text | — |
| 154 | Wang 2025 [154] — Pramipexole tablets | Moderate | Exposure misclassification window: identification periods reported separately | Low | Low | Low | Low | Low | Yes | Full text | — |
| 164 | Li 2025 [164] — Meropenem injection | High | Exposure misclassification window 18 months | Low | Low | Low | Low | Low | — | Full text | — |
| 182 | Ye 2024 [182] — Apixaban | High | Exposure misclassification window 19 months | Low | Low | Low | Low | Low | Yes | Full text | — |
| 183 | Wang 2023 [183] — Azacitidine injection | High | Exposure misclassification window 20 months | Low | Low | Low | Low | Low | Yes | Full text | — |
| 185 | Yang 2023 [185] — Entecavir | High | Exposure misclassification window: the study period opens 2010-01, nearly nine years before the round one announcement of 2018-12-07 (actual exposure in the generic arm began after 2019-12) | High | Low | High | Low | Low | — | Full text | — |
| 186 | Wang 2023 [186] — Cefdinir | Moderate | Exposure misclassification window 8 months | Low | Low | Low | Low | High | Yes | Full text | — |
| 194 | Xiao 2022 [194] — Amlodipine besylate | Low | Study period lies wholly after the announcement of the round | High | Moderate | Low | Low | High | — | Full text | — |
| 197 | Zhang 2022 [197] — Clopidogrel bisulfate | Low | Study period lies wholly after the announcement of the round | Low | Low | Low | Low | Low | Yes | Full text | The subgroup in which a difference was found contains five patients per arm, and six subgroup comparisons were made without adjustment for multiplicity |
| 198 | Chen 2021 [198] — Amlodipine besylate | Low | Study period lies wholly after the announcement of the round | Low | Low | Low | Low | Low | Yes | Full text | — |
| 200 | Wang 2024 [200] — Rivaroxaban | High | Exposure misclassification window 2 years | Low | Low | Low | Low | Low | Yes | Full text | — |
| 207 | Zhang 2025 [207] — Empagliflozin tablets | Moderate | Exposure misclassification window 1 month | Low | Low | Low | Low | High | — | Full text | No comparative safety outcome reported; safety represented only by a composite evaluation score |
| 208 | Zhang 2022 [208] — Amlodipine besylate; irbesartan | Moderate | Exposure misclassification window spans the date of selection | Low | Low | Low | Low | Moderate | Yes | Full text | Subgroup denominators are not reported alongside the response rates, and the pairwise comparisons underlying the two significant four-group tests do not compare the products under the same regimen |
| 211 | Zhang 2022 [211] — Moxifloxacin injection | High | Exposure misclassification window 2 years 6 months | Low | Low | Low | Low | High | — | Full text | — |
| 215 | Pang 2023 [215] — Moxifloxacin hydrochloride tablets | Low | Study period lies wholly after the announcement of the round | Low | Low | Low | Low | Low | — | Full text | — |
| 218 | Feng 2024 [218] — Levetiracetam concentrate for injection | Low | Study period lies wholly after the announcement of the round | Low | Low | Low | High | Low | Yes | Full text | — |
| 219 | Yang 2023 [219] — Ticagrelor tablets | Moderate | Exposure misclassification window 8 months | Low | Low | Low | Low | Low | — | Full text | — |
| 220 | Wen 2023 [220] — Vildagliptin tablets | Moderate | Exposure misclassification window 8 months | Low | Low | Low | Low | Low | — | Full text | — |
| 223 | Wang 2024 [223] — Atorvastatin | Low | Study period lies wholly after the announcement of the round | Low | Low | High | Low | Low | — | Full text | No matching or adjustment; baseline body mass index (P=0.010) and hypertension prevalence (P=0.009) differed |
| 224 | Yang 2023 [224] — Moxifloxacin sodium chloride injection | Moderate | Exposure misclassification window 8 months | Low | Low | High | Low | Low | — | Full text | — |
| 228 | He 2023 [228] — Linezolid glucose injection | High | Exposure misclassification window 18 months | Low | Low | Low | Low | Low | — | Full text | — |
| 236 | Liang 2022 [236] — Atorvastatin | Moderate | Exposure misclassification window 11 months | Low | Low | Low | Low | Low | — | Full text | — |
| 240 | Song 2021 [240] — Levetiracetam | Low | Study period lies wholly after the announcement of the round | Low | Low | High | Low | Low | — | Full text | — |
| 243 | Yan 2023 [243] — Levetiracetam tablets | Low | Study period lies wholly after the announcement of the round | Low | Low | Low | Low | Low | — | Full text | — |
| 244 | Liang 2022 [244] — Levetiracetam | Low | Study period lies wholly after the announcement of the round | Low | Low | Low | Low | Low | — | Full text | — |
| 248 | Zhang 2020 [248] — Clopidogrel bisulfate tablets | High | Exposure misclassification window 35 months (study period 2016-01 to 2019-01; results announced 2018-12-07; overlap about 55 days) | Low | Low | Low | Low | Low | — | Full text | — |
| 251 | Jiang 2022 [251] — Esomeprazole sodium for injection | High | Exposure misclassification window 18 months | Low | Low | High | Low | Low | — | Full text | Allocated by admission order without matching; the source describes the arms as comparable but reports mean ages of 60.50 and 47.34 years (t=14.0) |
| 257 | Xie 2021 [257] — Atorvastatin calcium tablets | High | Exposure misclassification window about 2 years, two thirds of the study period | Low | Low | Low | Low | Low | — | Full text | — |
| 264 | Liu 2025 [264] — Tenofovir alafenamide | High | Exposure misclassification window 2 years 10 months | Low | Low | Low | Low | High | — | Full text | — |
| 279 | Zhang 2023 [279] — Olanzapine tablets | Low | Study period lies wholly after the announcement of the round | Low | Moderate | High | Low | Low | — | Full text | — |
| 281 | Zhao 2024 [281] — Amlodipine | Low | Study period lies wholly after the announcement of the round | Low | Low | Low | Low | Low | — | Full text | — |
| 423 | Wang 2025 [423] — Linezolid | Moderate | Exposure misclassification window partially overlapping | Low | Low | Low | Low | Low | Yes | Full text | — |
| 603 | Yi 2024 [603] — Bortezomib for injection | High | Exposure misclassification window 4 years | Low | Low | Moderate | Low | Low | — | Full text | Propensity score matched, but baseline urea nitrogen and free light chain kappa remained imbalanced (both P<0.001) and are the two indices whose change scores later differed |
| 606 | Huang 2022 [606] — Entecavir | Low | Study period lies wholly after the announcement of the round | Low | Moderate | Low | Low | High | — | Full text | — |
| 607 | Zhang 2022 [607] — Cefuroxime sodium for injection | Moderate | Recruitment opens about six months before the announcement of round five and overlaps the post-announcement period by six months | Low | Low | Low | Low | Moderate | — | Full text | Safety was assessed in a hospital-wide population of 4,512 and 6,319 patients rather than in the 148 matched pairs used for efficacy D1 moderate: cefuroxime sodium for injection belongs to round five, announced 28 June 2021, and recruitment ran from January to December 2021. |
| 611 | Yi 2023 [611] — Atorvastatin | Low | Study period lies wholly after the announcement of the round | Low | Low | High | Low | Low | — | Full text | — |
| 618 | Liu 2024 [618] — Cefazolin, cefuroxime and ceftazidime injections | High | Exposure misclassification window 17 months (study period 2020-01 to 2022-09; round five announced 2021-06-28) | Low | High | Low | Low | Moderate | Yes | Full text | Laboratory denominators differ by index and none is reported; one entry is incompatible with its group size |
| 718 | Liang 2024 [718] — Tenofovir disoproxil fumarate; tenofovir alafenamide | Moderate | Exposure misclassification window: the first 6 months of the TAF arm precede the round seven announcement | Low | Low | Low | Low | High | — | Full text | Selective reporting: every arm was tested against tenofovir amibufenamide only and the selected-versus-comparator pairs were not tested. Arm sizes used in the review’s reconstruction were not read directly from the source |
| 720 | Wang 2023 [720] — Clopidogrel | High | Exposure misclassification window 1 year | Low | Low | High | Low | Low | — | Full text | — |
| 730 | Han 2021 [730] — Clopidogrel | Low | Study period lies wholly after the announcement of the round | Low | Low | Low | Low | Low | — | Full text | — |
| 739 | Luo 2023 [739] — Metformin | Low | Study period lies wholly after the announcement of the round | Low | Low | Low | Low | Low | Yes | Full text | The three safety indices use three different denominators (188/192, 159/162, 199/198) |
| 749 | Zhu 2024 [749] — Rivaroxaban | High | Exposure misclassification window 2 years 6 months | Low | Low | Low | Low | Low | — | Full text | — |
| 751 | Chen 2023 [751] — Capecitabine | Low | Study period lies wholly after the announcement of the round | Low | Low | Low | Low | Low | Yes | Full text | — |
| 752 | Yang 2021 [752] — Pemetrexed | Low | Study period lies wholly after the announcement of the round | Low | Low | Low | Low | Moderate | Yes | Full text | Significant differences are confined to the pre-matching comparison, in arms differing in stage, treatment intent, dose and cycle number; the matched analysis is the principal comparison |
| 753 | Li 2023 [753] — Ceftriaxone sodium for injection | High | Exposure misclassification window 18 months | Low | Low | Low | Low | Moderate | — | Full text | The reported test statistic for defervescence is not reproducible from the printed summary statistics, and the cost table prints identical values in two columns with different test statistics and components that do not sum to the stated total |
| 760 | Xu 2023 [760] — Valsartan/amlodipine | Low | Study period lies wholly after the announcement of the round | Low | Low | High | Low | Low | — | Full text | — |
| 823 | Cheng 2022 [823] — Budesonide suspension for inhalation | High | Exposure misclassification window: overlap of only 33 days | Low | Low | Low | Low | High | — | Full text | No safety outcome reported. In addition the Discussion section states the opposite of Table 3 for FEV1 and transposes the rhonchi values; the tables were followed |
| 840 | Ge 2024 [840] — Tigecycline for injection | Low | Study period lies wholly after the announcement of the round | High | Low | Low | Low | Low | — | Full text | — |
| 853 | Liu 2023 [853] — Moxifloxacin sodium chloride injection | Low | Study period lies wholly after the announcement of the round | Low | Low | Low | Low | Low | — | Full text | — |
| 1415 | Cheng 2025 [1415] — Amoxicillin as regimen component | Indeterminate | Study period could not be obtained, so this domain cannot be judged | Low | High | Low | Low | Low | — | Abstract only | — |
| X1 | Peng 2022 [X1] — Atorvastatin calcium tablets | Low | Study period lies wholly after the announcement of the round | Low | Low | Low | Low | Low | — | Full text | — |
| X2 | Yuan 2024 [X2] — Cefuroxime sodium for injection | Low | Study period lies wholly after the announcement of the round | Low | Low | Low | Low | Low | — | Full text | — |
| X3 | Wang 2023 [X3] — Linezolid tablets | Low | Study period lies wholly after the announcement of the round | Low | Low | Low | Low | Low | — | Full text | — |
| X4 | Wu 2024 [X4] — Atorvastatin calcium tablets | Low | Study period lies wholly after the announcement of the round | Low | Low | Low | Low | Low | — | Full text | — |
| X5 | Li 2024 [X5] — Ticagrelor tablets | Low | Study period lies wholly after the announcement of the round | High | Low | Low | Low | Low | — | Full text | — |
| X6 | Si 2024 [X6] — Febuxostat tablets | High | Exposure misclassification window 6 years 8 months | Low | Low | Low | Low | Low | — | Full text | — |
| X7 | Xing 2025 [X7] — Atorvastatin | Low | Study period lies wholly after the announcement of the round | Low | Low | Low | Low | Low | Yes | Full text | — |
| X8 | Luo 2023b [X8] — Glimepiride tablets | Low | Recruitment for the selected arm lies wholly after the announcement of round two | High | Moderate | Low | Low | Moderate | Yes | Full text | The two arms are drawn from non-overlapping calendar windows (originator 2019, selected product October 2020 to September 2021). The abstract and discussion state that persistence at six and nine months was lower with the selected product while the Results section states that it was higher; the corresponding table could not be read, so no direction is assigned to that outcome. The three safety comparisons use different denominators (78/78, 70/67, 80/81). Entered through citation searching. |
| X9 | Li 2023b [X9] — Glimepiride tablets | Moderate | Recruitment opens 21 days before the announcement of round two and overlaps the post-announcement period by 23 months | Low | Low | Low | Low | Low | Yes | Full text | Originator identified by brand (Amaryl). The selected arm pools three manufacturers (Shengping, Liyiping, Dibei), so the findings characterise those products in aggregate. Entered through citation searching. |

Distribution: D1 low 42, moderate 16, high 21, indeterminate 2. D2 low 71, high 10. D3 low 72, moderate 7, high 2. D4 low 68, moderate 1, high 12. D5 low 79, high 2. D6 low 59, moderate 7, high 15. D7 records a shared group or platform for 23 studies, arising from five groups. D8 full text 80, abstract only 1.

Twenty-four included studies were re-examined against the source article during this revision. Six report test statistics that cannot be reproduced from their own summary statistics or counts, and one states in its Discussion the opposite of what its tables show; these are graded in D6 and identified in the notes column. Wherever a source’s text, abstract or discussion conflicts with its tables, the tables were used.
