# Supplementary material for: Clinical efficacy and safety of medicines procured through China’s national volume-based procurement policy: a systematic review of 81 studies
Source: Front Public Health. 2026 Oct 15;14:1820505. doi: 10.3389/fpubh.2026.1820505 (PMC13716852; doi:10.3389/fpubh.2026.1820505)
Supplement: Supplementary file 4 [file Table_3.docx]

**Supplementary Table S3. Outcomes as reported by each included study, with effect estimates calculated by the review team where the source data allowed.**

For each included study the principal efficacy outcome and the principal comparative safety outcome are given as reported by the source. Where the source reported event counts, or counts recoverable exactly from reported percentages and group sizes, or means with standard deviations and group sizes, a risk ratio or a mean difference with its 95% confidence interval was calculated by the review team from those data. A ratio above 1, or a positive mean difference, indicates a higher value with the procurement-selected product. Where one arm had no events, 0.5 was added to every cell of the two-by-two table before the ratio was calculated and the entry is marked with a dagger; where neither arm had events, no ratio is estimable.

No value has been imputed, derived by back-calculation, or reconstructed from pooled ranges. Where a source reports only a statement of significance, or reports percentages without the corresponding group sizes, the effect estimate is recorded as not calculable rather than estimated. One study was extracted from the publisher’s abstract because the full text could not be obtained; its arm sizes are not reported and no counts have been derived for it. Post hoc analyses performed by the review authors are identified as such in the Notes and are also declared in the Methods.

Each study is entered once for each outcome domain to which it contributes. Studies are identified by first author and year with the screening record number in square brackets; the corresponding reference number is given in the first column of Table 1. Comparator strata are those defined in Section 2.2: I originator, II pre-policy baseline at the same institution, III non-selected generic of the same ingredient and dosage form, IV within-patient before and after substitution. Results from strata II to IV are reported separately and are not combined with stratum I.

| **Study** | **Medicine, design and analysed sample** | **Comparator stratum** | **Domain and outcome** | **Result as reported by the source** | **Effect estimate (95% CI)** | **Direction** | **Notes** |
| --- | --- | --- | --- | --- | --- | --- | --- |
| **Guo 2023 [38]** | Sertraline (round 3). Retrospective mirror-image cohort, PSM; Single centre, Beijing; First year after policy; 5,973 vs 7,686; 1,758 vs 1,758 after PSM. | I Originator | Therapeutic efficacy — Steady-state serum sertraline concentration; prescribed daily dose | Serum concentration 50 +/- 39 vs 54 +/- 40 ug/L (P=0.009); prescribed daily dose 161 +/- 47 vs 166 +/- 44 mg (P<0.001). No clinical efficacy endpoint was reported. | **MD -4.00 (-6.61 to -1.39)** | Significant difference; direction not assigned | Pharmacokinetic domain. The concentration difference is statistically significant (P=0.009) and the calculated interval excludes the null. Both group means lie within the 10-150 ug/L reference range and the source regards the difference as without clinical significance. No clinical efficacy endpoint is available, so the efficacy domain is recorded as not reported. |
|  |  |  | Comparative safety — Prolactin elevation; hepatic and renal abnormality; lipid abnormality | Prolactin elevation and hepatic and renal abnormality did not differ. HDL-C below the lower reference limit 41.8% vs 38.3% (P=0.039); LDL-C above the upper reference limit 41.8% vs 45.4% (P=0.032). | Not calculable from report | Opposing directions within cohort | Two lipid indices differ in opposite directions within the same cohort, so no direction is assigned under Section 2.7. Denominators for the lipid percentages are not reported. |
| **Song 2024 [89]** | Ceftriaxone injection (round 5). Multicentre retrospective cohort, PSM; 17 centres, Kunming; 2020-01 to 2022-09; 546 vs 1,275 before matching; 510 vs 510 after matching. | I Originator | Therapeutic efficacy — Clinical response, 72-h response, bacteriological efficacy, recovery of infection markers | Clinical response 487/510 (95.5%) vs 485/510 (95.2%), P=0.767; 72-h response 451/510 (88.4%) vs 433/510 (84.9%), P=0.244; bacteriological efficacy 41/510 (8.0%) vs 46/510 (9.0%), P=0.850. Recovery of temperature 138 vs 158, white cell count 88 vs 94, neutrophil count 85 vs 60, neutrophil percentage 94 vs 114, C-reactive protein 48 vs 72 and procalcitonin 21 vs 36 (P=0.332 to 0.054). | **RR 1.00 (0.98 to 1.03)** | No significant difference | — |
|  |  |  | Comparative safety — Adverse events; abnormality of WBC, NEUT, Hb, EOS, AST, ALT, Cr, Urea and platelets | Adverse events, all rash and all grade 1: 1/546 vs 3/1,275, P=1.000. Neutrophil reduction 10/510 (2.0%) vs 28/510 (5.5%), reported P=0.012. White cell reduction 13 vs 28 (P=0.057), haemoglobin reduction 17 vs 16 (P=0.094), eosinophil elevation 5 vs 1 (P=0.219), AST elevation 1 vs 7 (P=0.004), ALT elevation 5 vs 6 (P=0.108), creatinine elevation 3 vs 6 (P=0.009), urea elevation 5 vs 4 (P=0.09), platelet reduction 4 vs 10 and platelet elevation 24 vs 26 (P=0.406). No patient in either arm had a raised total bilirubin. | **RR 0.36 (0.18 to 0.73)** | Favours procured product | The estimate shown is for neutrophil reduction, the one significant comparison that reproduces from the reported counts (chi-square 8.9, P=0.003). The P values reported by this source are not reproducible from its own counts: the elevations of AST (1 vs 7) and creatinine (3 vs 6), which the source reports as significant at P=0.004 and P=0.009, give Fisher P=0.07 and chi-square P=0.32 respectively and are therefore not treated as significant here; the non-significant comparisons of haemoglobin, ALT and urea are likewise printed with P values that their counts do not support. Adverse-event denominators are the pre-matching group sizes (546 and 1,275); the laboratory denominators are the 510 matched pairs. |
| **Li 2025 [102]** | Olanzapine tablets (round 1). Retrospective cohort; Xiamen Third Hospital (reported as an unnamed tertiary hospital); 2022-01 to 2023-12; 288 vs 174. | I Originator | Therapeutic efficacy — Depression rating scale score after three months of treatment | Scale score after treatment 20.57 +/- 2.52 vs 19.73 +/- 2.25 points; the source reports t=0.369, P=0.952. Both arms improved from baseline (26.03 +/- 3.68 and 26.17 +/- 3.42); baseline scores did not differ (t=0.451, P=0.968). Prescribed daily dose 5.92 +/- 3.11 vs 5.54 +/- 2.40 mg, reported as t=6.592, P=0.015. | **MD 0.84 (0.40 to 1.28)** | No significant difference | This study reports a between-group efficacy endpoint and therefore contributes to the efficacy domain. Verified against the full text: the test statistics reported by this source are not reproducible from its own summary statistics and are mutually inconsistent throughout (t=8.535 with P=0.027, t=7.127 with P=0.034, t=0.369 with P=0.952, t=6.592 with P=0.015 are each impossible pairings), and its abstract states that daily dose did not differ while its Results section states that it did. The interval shown is calculated from the reported means, standard deviations and group sizes; the direction follows the source’s own conclusion of no difference. A lower scale score indicates better control. Group order verified: 288 selected, 174 originator. |
|  |  |  | Comparative safety — Glucose abnormality; lipid abnormality; total adverse reactions | Glucose abnormality 29/288 (10.07%) vs 8/174 (4.59%), chi-square 4.408, P=0.036. Total cholesterol 12/288 vs 7/174 (P=0.940), LDL-C 18/288 vs 11/174 (P=0.975), HDL-C 17/288 vs 10/174 (P=0.985), triglycerides 21/288 vs 20/174 (P=0.124), all abnormalities combined 97/288 (33.68%) vs 56/174 (32.18%), P=0.741. Total adverse reactions 108/288 (37.50%) vs 68/174 (39.08%), P=0.096. | **RR 2.19 (1.02 to 4.68)** | Favours originator | The direction rests on the glucose abnormality comparison, the only significant result. The chi-square values reported by this source are not reproducible from its own counts. |
| **Yang 2024 [103]** | Oxaliplatin injection (round 5). Active sentinel surveillance; Three tertiary centres, Guangzhou; 2022-10-01 to 2023-09-30; 8,400 vs 1,760 drug administrations (142 vs 30 ADR reports). | I Originator | Therapeutic efficacy — None reported | No efficacy endpoint reported. | Not calculable from report | Not applicable | Safety-only study. |
|  |  |  | Comparative safety — Adverse drug reaction reporting rate (CTCAE 5.0) | ADR reporting rate 1.69% (142/8,400) vs 1.70% (30/1,760), P=0.967; no organ-system category differed (P=0.12 to 1.00). Severity was predominantly grade 1-2. | **RR 0.99 (0.67 to 1.47)** | No significant difference | The denominator is drug administrations, not patients, and the numerator is spontaneous reports, so the figure is a reporting rate and not an incidence. The disease-category table of the source gives 143 and 29 where its other two tables give 142 and 30; the subgroup counts are therefore not cited. |
| **Qian 2024 [104]** | Omeprazole (round 3). Retrospective cohort; Single centre; 2022-01 to 2023-06; 158 vs 158. | I Originator | Therapeutic efficacy — Helicobacter pylori eradication | Eradication 88.61% (140/158) vs 93.04% (147/158); no significant difference. | **RR 0.95 (0.89 to 1.02)** | No significant difference | Counts recovered exactly from the reported percentages and group sizes. |
|  |  |  | Comparative safety — Adverse reactions | Adverse reactions 11/158 vs 9/158; no significant difference. | **RR 1.22 (0.52 to 2.87)** | No significant difference | — |
| **Wang 2023 [142]** | Tegafur (round 2). Multicentre retrospective cohort, 1:3 PSM; Three centres; 2019-01-01 to 2021-12-31; 695 vs 63. | I Originator | Therapeutic efficacy — CA19-9, CEA, CA72-4 and haemoglobin values and attainment rates | No between-group difference in any marker or attainment rate (all P>0.05). | Not calculable from report | No significant difference | Two selected manufacturers were compared against a shared originator arm (Hengrui 310, Qilu 385, originator 63). |
|  |  |  | Comparative safety — Adverse reactions; discontinuation for adverse reaction | Adverse reactions and discontinuation did not differ. | Not calculable from report | No significant difference | — |
| **Miao 2024 [143]** | Ticagrelor tablets (round 3). Retrospective cohort; Single centre; 2021-07 to 2023-06; 156 vs 264. | I Originator | Therapeutic efficacy — 12-month NACE, MACCE, event-free survival | No between-group difference in any endpoint (all P>0.05). | Not calculable from report | No significant difference | — |
|  |  |  | Comparative safety — Bleeding events; blood count | Bleeding events did not differ. | Not calculable from report | No significant difference | — |
| **Wang 2025 [154]** | Pramipexole tablets (round 4). Retrospective cohort, 1:1 PSM; Single centre, Beijing; 2018-07-01 to 2023-06-30; 935 vs 750. | I Originator | Therapeutic efficacy — Levodopa-equivalent dose trajectory over one year | Levodopa-equivalent dose did not differ between arms at any of the five time points. Scheirer-Ray-Hare test: between arms H=1.736, P=0.188; drug-by-time interaction H=1.538, P=0.820. A sensitivity analysis restricted to patients with at least five follow-ups (108 matched pairs) gave the same result. | Not calculable from report | No significant difference | The efficacy analysis uses the subset with at least four follow-up records, 188 matched pairs, not the 738 pairs matched overall. |
|  |  |  | Comparative safety — Adverse events identified from new diagnoses and concomitant prescriptions | Adverse events over the whole follow-up 123 events in the selected arm and 159 in the originator arm. At the first follow-up 76/738 (10.3%) vs 73/738 (9.9%), P=0.795. Log-rank comparison of adverse-event-free survival P=0.384; mean adverse-event-free survival 17 months in both arms. Neuropsychiatric events 88 vs 119 (P=0.295); gastrointestinal events 32 vs 43 (P=0.551). | **RR 1.04 (0.77 to 1.41)** | No significant difference | Verified against the full text: 123 events belong to the selected arm and 159 to the originator arm, and these are event counts over the whole follow-up rather than patients. The estimate shown is for the first follow-up, the only time point for which the source gives a rate with a denominator. P=0.795 belongs to that first-follow-up comparison; the log-rank test gives P=0.384. |
| **Ye 2024 [182]** | Apixaban (round 3). Multicentre retrospective cohort; Four centres; 2019-01-01 to 2022-04-01; 641 and 50 vs 295. | I Originator | Therapeutic efficacy — PT, INR, APTT, venous thromboembolism | No difference between the three arms in any index (all P>0.05). | Not calculable from report | No significant difference | Three-arm study; two selected manufacturers (Hengrui 641, Qilu Hainan 50) against a shared originator arm (295). Only the selected-versus-originator pairs are used. |
|  |  |  | Comparative safety — AST, ALT, urea, uric acid, creatinine; adverse drug reactions | No difference between arms in any safety index. | Not calculable from report | No significant difference | — |
| **Zhang 2025 [207]** | Empagliflozin tablets (round 4). Real-world retrospective cohort; Single centre, Urumqi; 2021-01 to 2023-12; Not stated. | I Originator | Therapeutic efficacy — Fasting plasma glucose, HbA1c, NT-proBNP | Fasting plasma glucose fell from 9.56 +/- 3.64 to 7.02 +/- 1.59 mmol/L in the selected arm and from 9.39 +/- 3.64 to 7.70 +/- 2.18 mmol/L in the originator arm; HbA1c from 8.50 +/- 1.87% to 7.47 +/- 1.13% and from 8.10 +/- 1.37% to 7.41 +/- 1.19%; NT-proBNP from 818.55 to 340.15 and from 754.00 to 332.00 pg/mL. All within-arm changes were significant (P<0.05); no between-group difference. | Not calculable from report | No significant difference | Group sizes are not reported by the source, so no estimate is calculable. Any figure stating arm sizes for this study is not supported by the record. |
|  |  |  | Comparative safety — None reported | The source reports no separate adverse-event rate; safety is represented only by a composite rapid-evaluation score (originator 74.6, selected 71.6). | Not calculable from report | Not applicable | Comparative safety recorded as not reported. |
| **Pang 2023 [215]** | Moxifloxacin hydrochloride tablets (round 2). Retrospective cohort; Single centre outpatient, Lianyungang; 2021-01 to 2021-12; 1,121 vs 1,058. | I Originator | Therapeutic efficacy — Clinical response; time to remission; treatment course; re-attendance | Clinical response 932/1,121 (83.14%) vs 906/1,058 (85.63%), chi-square 2.563, P=0.109; re-attendance 59/1,121 (5.26%) vs 66/1,058 (6.24%), P=0.261; treatment course did not differ. Time to remission 2.12 +/- 0.84 vs 1.86 +/- 0.73 days, P=0.016, longer with the selected product. Modified Poisson model for treatment failure: RR 1.132 (95% CI 0.883 to 1.542), P=0.327. | **RR 0.97 (0.94 to 1.01)** | No significant difference | Verified against the full text. The estimate shown is for clinical response, which did not differ; the significant efficacy finding is the longer time to remission. The arms differed at baseline in chronic obstructive pulmonary disease (14.63% vs 24.39%, P<0.001) and fever (23.19% vs 27.50%, P=0.021). |
|  |  |  | Comparative safety — Total adverse drug reactions; allergic, neurological, gastrointestinal, QT, tendon and glycaemic events | Total adverse drug reactions 76/1,121 (6.78%) vs 25/1,058 (2.36%), P<0.001. Allergic reactions 18 vs 3 (P=0.002); gastrointestinal reactions 30 vs 11 (P=0.009); abnormal blood glucose 17 vs 5 (P=0.015); neurological reactions (P=0.290) and QT prolongation (P=0.702) did not differ. | **RR 2.87 (1.84 to 4.47)** | Favours originator | Verified against the full text: the counts are those reported by the source. All four significant safety comparisons favour the originator. |
| **Feng 2024 [218]** | Levetiracetam concentrate for injection (round 3). Two-centre retrospective cohort, PSM; Two centres; 2021-11 to 2022-03; 18 vs 17 (treatment); 76 vs 76 (prophylaxis). | I Originator | Therapeutic efficacy — Treatment response (treatment subgroup); prophylactic response (prophylaxis subgroup) | Treatment subgroup response 50.00% (9/18) vs 58.82% (10/17); prophylaxis subgroup 97.37% (74/76) vs 100% (76/76); both P>0.05. | **RR 0.85 (0.46 to 1.56)** | No significant difference | The estimate shown is for the treatment subgroup. In the prophylaxis subgroup the originator arm had no failures, so a ratio is not informative. The originator product was itself a winning supplier of round three. |
|  |  |  | Comparative safety — Adverse drug reactions | No adverse drug reaction occurred in either subgroup in either arm. | Not calculable from report | No significant difference | Not estimable: no events in either arm. |
| **Wen 2023 [220]** | Vildagliptin tablets (round 3). Multicentre retrospective cohort, PSM; Four centres, Guangdong; 2020-01 to 2021-12; 3,039 vs 1,472. | I Originator | Therapeutic efficacy — One-year fall in HbA1c and fasting plasma glucose | HbA1c fall 0.50% vs 0.90% (P=0.814); fasting plasma glucose fall 0.59 vs 1.00 mmol/L (P=0.674). | Not calculable from report | No significant difference | Efficacy comparison rests on the matched subsets, whose sizes differ from the safety analysis set of 3,039 and 1,472. |
|  |  |  | Comparative safety — Adverse events; hypoglycaemia | Adverse drug reactions 1.6 vs 2.7 per 1,000 (P=0.721); hypoglycaemia 0.7 vs 0.7 per 1,000. | Not calculable from report | No significant difference | — |
| **Liu 2025 [264]** | Tenofovir alafenamide (round 7). Retrospective cohort, three parallel arms; Single centre, Tianjin; 2019-09 to 2024-09; 57 vs 58. | I Originator | Therapeutic efficacy — 48-week HBV-DNA negative conversion, HBeAg seroclearance, ALT normalisation, HBsAg clearance | HBV-DNA negative conversion 64.91% vs 63.79% (P=0.900); HBeAg seroclearance 37.50% (9/24) vs 41.18% (14/34) (P=0.778); ALT normalisation 55.00% (11/20) vs 52.38% (11/21) (P=0.867); HBsAg clearance nil in both arms. | **RR 0.91 (0.47 to 1.75)** | No significant difference | Three-arm study; the tenofovir amibufenamide arm is a different active ingredient and is not used. The estimate shown is for HBeAg seroclearance, the only efficacy outcome for which counts are reported. The manufacturer named for the selected arm does not appear on the round-seven award list and the two approval numbers appear transposed in the source; selected status is unverified and the study is removed in one sensitivity analysis. |
|  |  |  | Comparative safety — Hepatotoxicity; nephrotoxicity; lipid change | Hepatotoxicity 12.28% (7/57) vs 17.24% (10/58); nephrotoxicity 10.53% (6/57) vs 15.52% (9/58); three-group P=0.376 and 0.329. Triglycerides rose from baseline in the selected arm while falling in the imported originator arm. | **RR 0.71 (0.29 to 1.74)** | No significant difference | — |
| **Zhao 2024 [281]** | Amlodipine (round 1). Population-based matched cohort (claims); Tianjin; 2018 to 2020; follow-up to 2020-12-30; 2,949 vs 5,943; matched 4,360 vs 2,180. | I Originator | Therapeutic efficacy — MACE (all-cause death, stroke, myocardial infarction), heart failure, atrial fibrillation | Pure switchers vs non-switchers 2.86 vs 2.95 events per 100 person-years; HR 0.97 (95% CI 0.70 to 1.33). Back-switchers showed a higher stroke rate (HR 1.55, 95% CI 1.03 to 2.34). | Not calculable from report | No significant difference | Estimate with confidence interval reported by the source. Back-switching is not a generic-versus-originator comparison and is not counted. A companion report of the same Tianjin claims cohort exists and is listed as a second report of one study. |
|  |  |  | Comparative safety — Same endpoints as efficacy | The outcome events are themselves the safety events. | Not calculable from report | No significant difference | — |
| **Yi 2024 [603]** | Bortezomib for injection (round 4). Multicentre retrospective cohort, 1:1 PSM; Five centres, Shanghai; 2017-01 to 2024-06; 215 vs 215 after PSM. | I Originator | Therapeutic efficacy — Staged response after four cycles; change in M protein, plasma-cell fraction, beta-2 microglobulin, lactate dehydrogenase, creatinine, calcium, urea nitrogen and serum free light chains | Objective response 184/215 (85.58%) vs 193/215 (89.77%); four-category comparison P=0.053. Change in lactate dehydrogenase +11.33 +/- 114.45 vs +64.91 +/- 261.26 (P<0.001), in urea nitrogen −1.82 +/- 3.77 vs −2.62 +/- 5.07 (P=0.043) and in serum free light chain kappa −19.78 +/- 86.97 vs −11.50 +/- 62.02 (P=0.033). Change in M protein (P=0.546), plasma-cell fraction (P=0.657), beta-2 microglobulin (P=0.866), creatinine (P=0.207), calcium (P=0.514) and free light chain lambda (P=0.569) did not differ. | **RR 0.95 (0.89 to 1.02)** | No significant difference | The renal finding concerns blood urea nitrogen, not creatinine, which did not differ. Free light chain kappa also differed. Baseline values remained imbalanced after matching for exactly the two indices that later differed: urea nitrogen 7.22 +/- 4.79 vs 9.01 +/- 6.00 (P<0.001) and free light chain kappa 23.50 +/- 87.31 vs 14.3 +/- 61.36 (P<0.001), so regression to the mean is sufficient to account for both change scores. The source states that the arms were comparable after matching; its own baseline table does not support that statement. |
|  |  |  | Comparative safety — Gastrointestinal toxicity, fatigue, somnolence, peripheral neuropathy, pain, rash, allergy | Fatigue 29/215 vs 58/215, P<0.001. Gastrointestinal toxicity 31 vs 23 (P=0.193), somnolence 5 vs 0 (P=0.061), peripheral neuropathy 44 vs 29 (P=0.053), pain 5 vs 0 (P=0.061), rash 20 vs 10 (P=0.087) and allergy 3 vs 8 (P=0.221) did not differ. | **RR 0.50 (0.33 to 0.75)** | Favours procured product | Fatigue is the only significant safety comparison; peripheral neuropathy, more frequent with the selected product, approached significance at P=0.053. |
| **Han 2021 [730]** | Clopidogrel (round 1). Registry cohort (NCT04184583); Single centre, Beijing; 12-month follow-up; 1,657 vs 1,753. | I Originator | Therapeutic efficacy — 12-month MACCE | MACCE 4.91% vs 5.07% (P=0.83). | Not calculable from report | No significant difference | Group sizes 1,657 selected and 1,753 originator. Single endpoint figures in the source are internally inconsistent, so only MACCE and BARC>=2 major bleeding are cited. |
|  |  |  | Comparative safety — BARC >=2 major bleeding | Major bleeding, log-rank P=0.60. | Not calculable from report | No significant difference | — |
| **Chen 2023 [751]** | Capecitabine (round 3). Retrospective cohort, PSM; Single centre, Beijing; 2020-11 to 2021-11; 118 vs 118 after PSM. | I Originator | Therapeutic efficacy — Objective response and disease control in first-line patients | ORR and DCR did not differ (P=0.05 and P=0.196). | Not calculable from report | No significant difference | The source states explicitly that P=0.05 did not meet its threshold. The efficacy analysis set is confined to first-line patients and is smaller than the 118 matched pairs used for safety. |
|  |  |  | Comparative safety — Adverse reactions | Adverse reactions did not differ (P>=0.05). | Not calculable from report | No significant difference | — |
| **Ge 2024 [840]** | Tigecycline for injection (round 7). Before-and-after retrospective analysis; Single centre, Shanghai; Pre 2021-12 to 2022-05; post 2022-12 to 2023-05; 76 vs 86. | II and III | Therapeutic efficacy — Clinical response | Clinical response 89.47% (post-policy) vs 88.37% (pre-policy), P>0.05. | Not calculable from report | No significant difference | Before-and-after design; 76 post-policy and 86 pre-policy. |
|  |  |  | Comparative safety — Spontaneously reported ADRs | ADR reports 7 (9.21%) vs 6 (7.41%); not tested. | Not calculable from report | No significant difference | The pre-period percentage does not correspond to 6/86, so no estimate is derived. |
| **Liu 2023 [853]** | Moxifloxacin sodium chloride injection (round 3). Retrospective cohort, PSM; Single centre, Shijiazhuang; 2020-10 to 2021-10; 61 vs 111 after PSM. | I Originator | Therapeutic efficacy — 72-h and discharge response grading; temperature, CRP, PCT | No between-group difference in any outcome (all P>0.05). | Not calculable from report | No significant difference | 61 selected and 111 originator after matching. |
|  |  |  | Comparative safety — Total adverse reactions | Total adverse reaction rate did not differ. | Not calculable from report | No significant difference | — |
| **Cheng 2025 [1415]** | Amoxicillin as regimen component (rounds 2 and 3). Multicentre randomised controlled trial, three arms; Multicentre; Not stated; 450 randomised. | III Non-selected generic | Therapeutic efficacy — Helicobacter pylori eradication (urea breath test; per-protocol and intention-to-treat) | Per-protocol eradication 90.7% (procured amoxicillin quadruple) vs 92.1% (non-procured amoxicillin quadruple) vs 91.7% (vonoprazan dual); three-group P=0.889. Intention-to-treat 84.7% vs 85.3% vs 88.0%; three-group P=0.947. Cost per course fell by 44% (CNY 239 vs 315). | Not calculable from report | No significant difference | Extracted from the publisher’s abstract; the full text could not be obtained and the corresponding author did not reply. The source gives percentages only; arm sizes are not reported and counts have NOT been derived from the percentages. The eligible comparison is the two quadruple arms, identical apart from the source of the amoxicillin component; the vonoprazan dual arm is a different regimen and is not used. The comparator is a non-selected product, so this study sits in the non-selected-generic stratum. |
|  |  |  | Comparative safety — Not reported by arm | The abstract gives adverse events and adherence only as ranges pooled across the two quadruple arms (any adverse event 6.8% for vonoprazan dual vs 17.9%-19% for the two quadruple arms, P=0.003; dysgeusia 1.4% vs 15.2%-15.6%, P<0.001; adherence 99% vs 95.2%-95.9%, P<0.04). | Not calculable from report | Not applicable | Comparative safety recorded as not reported because the abstract does not separate the two quadruple arms. The adherence figure printed as "95.9.7%" in the abstract is a typesetting error and is not cited. |
| **Yang 2023 [219]** | Ticagrelor tablets (round 3). Retrospective cohort, PSM; Single centre, Dalian; 2020-01 to 2021-07; 690 vs 690 after PSM. | I Originator | Therapeutic efficacy — 12-month primary composite (cardiac death, stroke, target-vessel revascularisation, reinfarction); secondary composite | After matching, primary composite 12.6% (87/690) vs 12.3% (85/690) and secondary composite 11.0% vs 8.3%; both P>0.05. | **RR 1.02 (0.77 to 1.35)** | No significant difference | Counts recovered exactly from the reported percentages and matched group size. |
|  |  |  | Comparative safety — Bleeding | Bleeding 0.3% vs 0.4% (P>0.05); no deaths. | Not calculable from report | No significant difference | — |
| **Wang 2024 [223]** | Atorvastatin (round 1). Retrospective cohort; Single centre, Xuyi; 2022-01 to 2023-12; 263 vs 255. | I Originator | Therapeutic efficacy — Total cholesterol, triglycerides, LDL-C and HDL-C at 12 weeks; same indices within comorbidity subgroups | At 12 weeks TC 4.93 +/- 0.69 vs 4.88 +/- 0.73 (P=0.345), TG 1.35 +/- 0.56 vs 1.28 +/- 0.44 (P=0.094), LDL-C 2.52 +/- 0.52 vs 2.60 +/- 0.50 (P=0.064) and HDL-C 1.21 +/- 0.25 vs 1.23 +/- 0.22 (P=0.581). Within the coronary disease, stroke and diabetes subgroups neither TC nor LDL-C differed at either time point. | Not calculable from report | No significant difference | No matching or adjustment was applied and the arms differed at baseline in body mass index (25.59 +/- 3.07 vs 26.33 +/- 3.46, P=0.010) and in the proportion with hypertension (43.4% vs 54.9%, P=0.009). |
|  |  |  | Comparative safety — AST, ALT and creatine kinase; adverse drug reactions | AST and ALT did not differ. Creatine kinase was higher with the domestic product at 12 weeks (P<0.05). Adverse drug reactions 37/263 (14.1%) vs 25/255 (9.8%), P=0.135; univariable regression found comparable risk between the two products (P=0.347) with a higher risk in men (P=0.032), and multivariable regression found no factor with a significant effect. | **RR 1.43 (0.89 to 2.31)** (estimate is for the outcome named above, which did not differ; the direction rests on a further comparison for which counts are not reported) | Favours originator | The direction rests on the creatine kinase comparison, which the source lists among its pre-specified safety indicators; the estimate shown is for adverse drug reactions, which did not differ. |
| **He 2023 [228]** | Linezolid glucose injection (round 5). Retrospective cohort, PSM; Single centre, Nanchong; 2020-01 to 2022-04; 30 vs 30 after PSM. | I Originator | Therapeutic efficacy — 30-day all-cause mortality, in-hospital mortality; duration of bacteraemia, length of stay | Mortality and duration of bacteraemia did not differ (P>0.05); length of stay was significantly longer in the exposed arm (P<0.05). | Not calculable from report | No significant difference | 30 per arm after matching. Length of stay is a utilisation measure and does not determine direction. |
|  |  |  | Comparative safety — Adverse drug reactions | Adverse drug reaction rate did not differ (P>0.05). | Not calculable from report | No significant difference | — |
| **Liang 2022 [236]** | Atorvastatin (round 1). Retrospective cohort, 1:1 PSM; Two centres, Guangzhou; 2018-01-01 to 2021-03-31; 363 vs 302. | I Originator | Therapeutic efficacy — Recurrent ischaemic stroke or TIA, NIHSS and mRS change, incident coronary disease | No between-group difference in any efficacy index before or after matching. | Not calculable from report | No significant difference | 363 generic and 302 originator. |
|  |  |  | Comparative safety — AST/ALT elevation; composite adverse events | No between-group difference in any safety index. | Not calculable from report | No significant difference | — |
| **Song 2021 [240]** | Levetiracetam (round 1). Retrospective real-world study; Single centre, Xi an; 2019-04 to 2020-05; 125 vs 475. | I Originator | Therapeutic efficacy — Serum concentration; response rate under monotherapy and each co-medication pattern | Monotherapy response 78.9% vs 74.1%; with enzyme inducers 64.3% vs 62.7%; with enzyme inhibitors 59.3% vs 78.2%; with both 69.2% vs 71.4%. No between-group difference. | Not calculable from report | No significant difference | Subgroup denominators are not reported alongside the percentages (125 generic and 475 originator overall). |
|  |  |  | Comparative safety — Adverse reactions | Adverse reaction rates were similar; the commonest were behavioural change and difficulty falling asleep. | Not calculable from report | No significant difference | — |
| **Yan 2023 [243]** | Levetiracetam tablets (round 1). Retrospective cohort with outpatient follow-up; Single centre, Suzhou; 2020-01 to 2022-06; 53 vs 72. | I Originator | Therapeutic efficacy — Seizure control; reduction in seizure frequency | Seizure control 83.02% (44/53) with the selected product vs 87.50% (63/72) with the originator, chi-square 0.497, P=0.481. Median seizure frequency after treatment 0 (IQR 0-0) in both arms, P=0.335. An increasing number of concomitant antiseizure medicines lowered the probability of control (OR 0.333, 95% CI 0.114 to 0.969, P=0.044); drug brand was not associated with control. | **RR 0.95 (0.82 to 1.10)** | No significant difference | Arm order verified against the full text: the originator arm is 72 patients with 63 controlled and the selected arm 53 patients with 44 controlled. |
|  |  |  | Comparative safety — Adverse reactions | Four adverse reactions were reported, all in originator-treated patients on monotherapy (emotional lability 2, rash 2); no patient stopped or reduced treatment. The source performs no statistical test of this comparison and states that the number of reports is too small to support a conclusion about safety. | **RR 0.15 (0.01 to 2.73) †** | No significant difference | Verified against the full text: the source neither tests this comparison nor draws a safety conclusion from it, and the calculated ratio, with 0.5 continuity correction, has an interval that includes the null. The study is therefore recorded as showing no significant difference. |
| **Liang 2022 [244]** | Levetiracetam (round 1). Single-arm before-and-after switch; Single centre, Guangzhou; 2019-05 to 2020-12; 154. | IV Within-patient | Therapeutic efficacy — Seizure control; seizure frequency | Seizure control 77.3% before substitution and 83.8% after (P<0.05); seizure frequency did not differ. | Not calculable from report | Favours procured product | Within-patient before-and-after design in 154 children; the two observations are paired, so a between-arm ratio is not calculable. The direction follows the source. |
|  |  |  | Comparative safety — Adverse drug reactions | One mild event (0.6%) before substitution and three (1.9%) after (P>0.05). | **RR 3.00 (0.32 to 28.52)** | No significant difference | Paired design; the ratio is shown for completeness only. |
| **Jiang 2022 [251]** | Esomeprazole sodium for injection (round 5). Prospective allocation by admission sequence; Single centre, Changzhou; 2020-01 to 2022-01; 43 vs 43. | I Originator | Therapeutic efficacy — Treatment response; episodes of haematemesis and melaena on days 1 to 5 | Treatment response 39/43 (90.69%) vs 41/43 (95.35%), chi-square 0.717, P=0.397. Haematemesis episodes on days 1 to 5 were fewer with the selected product at every time point (2.78 +/- 0.73 vs 3.20 +/- 0.81; 1.80 +/- 0.55 vs 2.13 +/- 0.51; 0.92 +/- 0.30 vs 1.13 +/- 0.42; 0.69 +/- 0.19 vs 0.83 +/- 0.23; 0.33 +/- 0.13 vs 0.52 +/- 0.24; t=2.526 to 4.565, P=0.013 to <0.001), as were melaena episodes (3.18 +/- 0.79 vs 3.56 +/- 0.87; 1.86 +/- 0.66 vs 2.22 +/- 0.61; 0.71 +/- 0.58 vs 0.99 +/- 0.67; 0.54 +/- 0.24 vs 0.66 +/- 0.26; 0.19 +/- 0.14 vs 0.34 +/- 0.22; t=2.072 to 3.772, P=0.037 to <0.001). | **RR 0.95 (0.85 to 1.07)** | No significant difference | All ten symptom comparisons favour the selected product and all ten test statistics reproduce from the printed means, standard deviations and group sizes. They are secondary symptom measures; the primary outcome, treatment response, did not differ, so no direction is assigned. The source states that the arms were comparable at baseline, but the mean ages it reports are 60.50 +/- 5.10 years in the selected arm and 47.34 +/- 3.43 in the originator arm, which gives t=14.0; a thirteen-year difference in an upper gastrointestinal bleeding population is a major confounder and the study neither matched nor adjusted. |
|  |  |  | Comparative safety — Adverse reactions | Adverse reactions 4/43 (9.30%) vs 6/43 (13.95%), chi-square 0.453, P=0.502. By type: abdominal pain 1 vs 1, diarrhoea 1 vs 2, nausea or vomiting 1 vs 1, hepatic abnormality 1 vs 2. | **RR 0.67 (0.20 to 2.20)** | No significant difference | — |
| **Xie 2021 [257]** | Atorvastatin calcium tablets (round 1). Prospective randomised allocation; Single centre, Qianjiang; 2017-01 to 2019-12; 75 vs 75. | I Originator | Therapeutic efficacy — Overall lipid-lowering response; TG, TC, LDL-C, HDL-C over 24 weeks | Overall response 92.00% (69/75) vs 94.67% (71/75) (P>0.05); no between-group difference in lipid indices. | **RR 0.97 (0.89 to 1.06)** | No significant difference | — |
|  |  |  | Comparative safety — Adverse reactions | Adverse reactions 10.67% (8/75) vs 12.00% (9/75) (P>0.05). | **RR 0.89 (0.36 to 2.18)** | No significant difference | — |
| **Wang 2025 [423]** | Linezolid (rounds 3 and 5). Retrospective cohort, PSM; Single centre, Beijing; 2019-01 to 2023-06; 137 vs 137 after PSM. | I Originator | Therapeutic efficacy — None reported | No efficacy endpoint reported. | Not calculable from report | Not applicable | Safety-only study. |
|  |  |  | Comparative safety — Thrombocytopenia; severe thrombocytopenia; anaemia reaching transfusion threshold | Thrombocytopenia 28.44% (39/137) vs 21.17% (29/137); severe thrombocytopenia 6.42% vs 4.95%; anaemia reaching transfusion threshold 2.75% vs 3.15%; all P>0.05. Falls in platelet count and haemoglobin during follow-up also did not differ. | **RR 1.34 (0.89 to 2.04)** | No significant difference | — |
| **Yi 2023 [611]** | Atorvastatin (round 1). Retrospective cohort; Single centre, Hezhou; 2020-07 to 2022-07; 55 vs 33. | I Originator | Therapeutic efficacy — TC, TG, HDL-C, LDL-C; fasting glucose and HbA1c; overall response | TC, LDL-C and TG fell within both arms (P<0.05) with no between-group difference; HDL-C, fasting glucose, HbA1c and overall response also did not differ. | Not calculable from report | No significant difference | 55 observation and 33 control. |
|  |  |  | Comparative safety — ALT and AST abnormality | ALT elevation 2 vs 2, AST elevation 1 vs 1, AST reduction 1 vs 3; other ALT and AST values did not differ. | **RR 0.60 (0.09 to 4.06)** | No significant difference | — |
| **Yang 2024 [26]** | Atorvastatin (round 1). Retrospective cohort, 1:1 PSM; Single centre, Hangzhou; Originator arm 2019; generic arm 2023; 92 vs 92. | I Originator | Therapeutic efficacy — LDL-C target attainment at three months; percentage fall in LDL-C; readmission for recurrence | Median percentage fall in LDL-C 40.94% (IQR 29.10-48.57) vs 39.63% (IQR 32.11-47.30), P=0.992; target attainment 53.26% vs 50.91%, P=0.658; readmission for recurrence 4/92 (4.35%) vs 4/92 (4.35%), P=1.000. | **RR 1.00 (0.26 to 3.88)** | No significant difference | Verified against the full text: the source reports the attainment rates as percentages only, with no counts. 53.26% resolves exactly to 49/92, but 50.91% has no integer solution at n=92 (46/92 = 50.00%, 47/92 = 51.09%); the inconsistency is in the published table, not in extraction, and no count is derived for that arm. The estimate shown is for readmission. The two arms were drawn four years apart (selected 2023, originator 2019). |
|  |  |  | Comparative safety — ALT, AST or CK elevation; other adverse drug reactions | Patients with any adverse drug reaction 5/92 (5.43%) vs 4/92 (4.35%), P=1.000. By type, selected arm ALT 4, AST 4, CK 1; originator arm ALT 3, AST 1, CK 0; no other adverse reaction in either arm. One patient in the selected arm reached ALT 1,040 U/L and AST 1,057 U/L, assessed as probably related and resolved after treatment. | **RR 1.25 (0.35 to 4.51)** | No significant difference | The type counts are events and exceed the patient counts; the denominators for the ratio are patients. |
| **Sun 2024 [72]** | Cefuroxime injection (round 5). Retrospective cohort, PSM; Single centre, Fuzhou; 2020-11 to 2022-11; 1,042 vs 1,520 before matching; 1:1 matched within each department. | I Originator | Therapeutic efficacy — Efficacy indices (four surgical departments, matched separately) | No between-group difference. | Not calculable from report | No significant difference | Matched pairs are reported department by department in the source and no pooled figure is given. A companion report covering gynaecology and obstetrics is listed as a second report of one study and must not be counted twice. |
|  |  |  | Comparative safety — Adverse reactions | No between-group difference. | Not calculable from report | No significant difference | — |
| **Cai 2024 [75]** | Levofloxacin sodium chloride injection (round 5). Retrospective cohort, PSM; Single centre, Fuzhou; 2020-10-20 to 2022-10-20; 117 vs 117. | I Originator | Therapeutic efficacy — Day-7 clinical response; WBC, NEUT%, CRP, PCT, temperature, treatment course | No between-group difference in any outcome. | Not calculable from report | No significant difference | 117 per arm after matching. |
|  |  |  | Comparative safety — Hepatic and renal abnormality | No between-group difference. | Not calculable from report | No significant difference | — |
| **Xue 2024 [78]** | Amlodipine besylate (round 1). Prospective randomised allocation; Single centre, Shenzhen; 2021-01 to 2021-03; 100 vs 100. | I Originator | Therapeutic efficacy — Overall clinical response; blood-pressure control | Overall response did not differ (P>0.05); blood-pressure control 78.00% (78/100) vs 62.00% (62/100) (P<0.05). | **RR 1.26 (1.05 to 1.51)** | Direction not assigned | No direction is assigned: the selected-product arm additionally received a remote pharmaceutical care programme that the comparator arm did not, so the difference cannot be attributed to the medicine. |
|  |  |  | Comparative safety — Total adverse reactions | Adverse reactions 2.00% (2/100) vs 1.00% (1/100) (P>0.05). | **RR 2.00 (0.18 to 21.71)** | No significant difference | — |
| **Li 2026 [82]** | Voriconazole tablets (round 4). Retrospective cohort (DRG group ES13); Single centre, Linyi; 2019-01 to 2023-12; 90 vs 78. | I Originator | Therapeutic efficacy — Clinical response; length of stay; unplanned readmission | Clinical response 96.67% (87/90) with the selected product vs 89.74% (70/78) with the originator; length of stay 6.64 +/- 2.43 vs 7.26 +/- 3.30 days; unplanned readmission 5.56% vs 6.41%; all P>0.05. | **RR 1.08 (0.99 to 1.17)** | No significant difference | Arm order established by recovery of counts against the group sizes (70/78=89.74%, 87/90=96.67%). |
|  |  |  | Comparative safety — Voriconazole-related ALT elevation | ALT elevation 3.33% vs 2.56% (P>0.05). | Not calculable from report | No significant difference | Counts resolve to 3/90 and 2/78 but are not stated as counts by the source; no estimate is entered. |
| **Li 2025 [83]** | Saxagliptin (round 5). Multicentre retrospective cohort, PSM; Four centres; 2021-09 to 2023-08; 178 vs 164; 141 vs 141 after PSM. | I Originator | Therapeutic efficacy — Six-month change in HbA1c and fasting plasma glucose; HbA1c attainment | No between-group difference in any outcome (all P>0.05). | Not calculable from report | No significant difference | 141 per arm after matching. |
|  |  |  | Comparative safety — Hypoglycaemia; hepatic and renal abnormality | No between-group difference. | Not calculable from report | No significant difference | — |
| **Li 2025 [96]** | Caspofungin acetate injection (round 9). Retrospective cohort, PSM; Single centre, Huangshi; 2020-01 to 2024-06; 117 vs 117 after PSM. | I Originator | Therapeutic efficacy — Composite cure (clinical response, imaging and microbiological clearance all met) | Composite cure 80/117 vs 85/117, P=0.283. Clinical cure or improvement 87/117 (74.36%) vs 93/117 (79.49%), P=0.883; microbiological clearance or presumed clearance 86/117 (73.50%) vs 89/117 (76.06%), P=0.382. | **RR 0.94 (0.80 to 1.11)** | No significant difference | Verified against the full text. The source prints the composite cure rate as 69.37% for the selected arm, but 80/117 is 68.38%, and the source’s own failure count for that arm (37/117, 31.63%) implies 68.37%; the printed 69.37% is a typographical error for 68.37%. The counts, not the printed percentage, are used here. The comparator arm resolves exactly (85/117 = 72.65%). |
|  |  |  | Comparative safety — Adverse reactions (hepatic and renal abnormality, nausea or vomiting, electrolyte disturbance, rash) | Adverse reactions 17/117 (14.52%) vs 14/117 (11.96%); no significant difference. | **RR 1.21 (0.63 to 2.35)** | No significant difference | Counts taken from the text of the source; the tables report the rate only. |
| **Jia 2022 [107]** | Paroxetine tablets (round 1). Before-and-after cohort, PSM; Single centre, Beijing; 23 Mar to 23 Sep 2019, with 23 Mar to 23 Sep 2018 as the preceding-year reference; 1,168 vs 1,168 after matching. | I Originator | Therapeutic efficacy — Prescribed dose; serum concentration (no clinical efficacy endpoint) | Prescribed dose and serum concentration did not differ (P>0.05). | Not calculable from report | No significant difference | 1,168 per arm after stratified matching within the post-policy period. |
|  |  |  | Comparative safety — Hepatic and renal abnormality; lipid, glucose and prolactin abnormality | No abnormality rate differed between arms (P>0.05). | Not calculable from report | No significant difference | — |
| **Wang 2024 [119]** | Empagliflozin (round 4). Retrospective cohort; Single centre, Beijing; 2022-06 to 2023-06; 406. | I Originator | Therapeutic efficacy — Fasting plasma glucose, HbA1c, NT-proBNP, LVEF, eGFR and a creatinine index | After treatment, fasting plasma glucose 6.96 +/- 2.30 vs 6.71 +/- 2.10 (t=−1.105, P=0.270), eGFR 88.21 +/- 27.45 vs 91.12 +/- 27.78 (P=0.290), NT-proBNP 1,759.86 +/- 5,463.00 vs 915.27 +/- 3,353.22 (P=0.063) and LVEF 55.73 +/- 13.69 vs 55.53 +/- 12.47 (P=0.874) did not differ. The index printed as GREA was 85.29 +/- 49.94 vs 72.78 +/- 23.98 after treatment, t=−3.186, P=0.002. HbA1c was 7.30 +/- 1.78 vs 7.80 +/- 1.61 before treatment (reported t=2.964, P=0.381) and 7.10 +/- 1.58 vs 7.51 +/- 1.43 after treatment (reported t=2.693, P=0.340). | Not calculable from report | Direction not assigned | Direction not determinable, because of a defect in the source rather than a missing table: the Results text describes the significant index as the creatinine clearance rate, for which a higher value is better, while the legend of the same table defines it as creatinine, for which a higher value is worse; no unit is given and the two readings imply opposite directions. Group sizes are 197 with the selected product and 209 with the originator. The HbA1c P values are not consistent with the source’s own test statistics: t=2.964 and t=2.693 at these group sizes correspond to P of about 0.003 and 0.007, not 0.381 and 0.340, so HbA1c differed both before and after treatment, with the lower values in the selected arm; because it already differed at baseline the post-treatment difference cannot be attributed to the medicine. No matching or adjustment was applied and the arms differed in comorbidity and in concomitant heart-failure therapy. |
|  |  |  | Comparative safety — None reported as a tested comparison | The source reports counts without tests: eGFR fell by more than 30% in 6 patients in the selected arm and 7 in the originator arm; urinary tract infection in 2 and 4; hypoglycaemia in 1 and 3; no diabetic ketoacidosis in either arm. | Not calculable from report | Not applicable | Comparative safety recorded as not reported because no safety comparison was tested. |
| **Chen 2023 [144]** | 20 medicines in 10 groups (multiple rounds). Multi-medicine retrospective analysis with active surveillance; Single centre, Guangzhou; 2020 to 2022; Not stated. | I Originator | Therapeutic efficacy — None reported | No clinical efficacy endpoint reported. | Not calculable from report | Not applicable | — |
|  |  |  | Comparative safety — ADR incidence (spontaneous reporting and active surveillance) | For most selected generics the ADR rate did not differ from the non-selected originator; for a minority the selected product had a significantly lower rate. | Not calculable from report | Direction not assigned | No direction assigned: twenty representative medicines in ten groups are reported in aggregate, the source does not identify which medicines differed, and no denominator is given for any medicine. Under the rule adopted for this review an outcome reported without an identifiable denominator does not determine direction. The medicines span several procurement rounds, so no single announcement date applies and exposure misclassification is graded indeterminate. |
| **Li 2025 [164]** | Meropenem injection (round 7). Retrospective cohort, PSM; Single centre, Huangshi; 2021-01 to 2024-06; 193 vs 193 after PSM. | I Originator | Therapeutic efficacy — Cure rate | Cure rate 86.01% (166/193) vs 91.19% (176/193) (P>0.05). | **RR 0.94 (0.88 to 1.01)** | No significant difference | — |
|  |  |  | Comparative safety — Total adverse reactions | Adverse reactions 7.25% vs 5.70% (P>0.05). | Not calculable from report | No significant difference | Counts resolve to 14/193 and 11/193 but are not stated as counts by the source; no estimate is entered. |
| **Wang 2023 [183]** | Azacitidine injection (round 3). Multicentre retrospective cohort, PSM; Three centres; 2019-01-01 to 2022-03-31; 93 vs 53. | I Originator | Therapeutic efficacy — Remission within two cycles | Remission rate did not differ (P>0.05). | Not calculable from report | No significant difference | Three-arm study; the two generic arms are anonymised in the source and the corresponding author did not reply. Both arms are recorded as of unconfirmed selected status and the study is removed in one sensitivity analysis. |
|  |  |  | Comparative safety — Myelosuppression, secondary infection, gastrointestinal reaction, hepatic injury, fever, fatigue | No adverse reaction category differed (P>0.05). | Not calculable from report | No significant difference | — |
| **Yang 2023 [185]** | Entecavir (round 1). Retrospective cohort; Single centre, Jinan; 2010-01 to 2022-12 (the hospital first stocked the selected capsule in December 2019); 30 vs 30. | I Originator | Therapeutic efficacy — HBV-DNA, HBsAg and HBeAg negative conversion; ALT normalisation | No between-group difference in any efficacy index (P>0.05). | Not calculable from report | No significant difference | 30 per arm. |
|  |  |  | Comparative safety — Nephrotoxicity and hepatotoxicity | No between-group difference (P>0.05). | Not calculable from report | No significant difference | — |
| **Wang 2023 [186]** | Cefdinir (round 3). Retrospective cohort, PSM; Single centre, Beijing; 2020-01-01 to 2021-12-31; 1,268 vs 1,268 after PSM. | I Originator | Therapeutic efficacy — None compared between groups | The source compares only within the selected arm before and after treatment. Daily dose, treatment course and use intensity were all lower in the selected arm (P<0.001). | Not calculable from report | Not applicable | No between-group efficacy comparison is available, so the study enters the safety domain only. The selected product is a dispersible tablet and the comparator an originator capsule of the same ingredient and strength, which are within one procurement item. |
|  |  |  | Comparative safety — ALT, AST, creatinine, urea nitrogen; ADR reporting | Hepatic and renal indices after treatment did not differ; no ADR was reported in either arm. | Not calculable from report | No significant difference | 1,268 per arm after matching. |
| **Zhang 2022 [197]** | Clopidogrel bisulfate (round 1). Genotype-stratified randomised allocation; Single centre, Kunming; 2019-12 to 2020-12; 40 vs 40. | I Originator | Therapeutic efficacy — ADP inhibition and MA-ADP; one-year exercise test, stent thrombosis, restenosis, ischaemia, MACE | Overall ADP inhibition 62.61 +/- 10.165 vs 64.80 +/- 6.694 on day 5 of loading dosing (t=−1.137, P=0.259) and 71.69 +/- 11.237 vs 75.93 +/- 8.509 on day 5 of maintenance dosing (t=−1.905, P=0.060); response grading did not differ at either time point (Fisher 1.723, P=0.588; Fisher 1.708, P=0.572). MA-ADP 40.87 +/- 4.960 vs 39.78 +/- 3.722 and 38.33 +/- 4.930 vs 38.70 +/- 3.444, both P>0.05. Within the slow-metaboliser subgroup ADP inhibition was lower with the selected product at loading (43.02 +/- 8.025 vs 56.52 +/- 5.206) and at maintenance dosing (51.92 +/- 12.83 vs 68.02 +/- 6.375), both P<0.05. | **MD -13.50 (CI not calculable)** | No significant difference | The slow-metaboliser subgroup contains FIVE patients per arm. Recalculation from the reported means and standard deviations gives t=3.16 (P=0.014) and t=2.51 (P=0.036); no confidence interval is entered because the comparison rests on five patients per arm. Three genotype subgroups were each compared at two time points, six comparisons in all, without adjustment for multiplicity. The fast- and intermediate-metaboliser subgroups showed no difference in any of four indices. Randomisation was performed within each genotype stratum. The primary comparison, overall ADP inhibition, did not differ, so no direction is assigned. |
|  |  |  | Comparative safety — Stent thrombosis, restenosis, adverse drug reactions within one year | No between-group difference (Fisher=0.157, P>0.05). | Not calculable from report | No significant difference | — |
| **Wang 2024 [200]** | Rivaroxaban (round 5). Retrospective cohort, IPTW; Single centre, Kunming; 2019-07 to 2022-06; 529. | I Originator | Therapeutic efficacy — Stroke, systemic embolism, readmission | After inverse probability weighting: systemic embolism HR 0.280 (95% CI 0.029 to 2.723, P=0.273); stroke HR 1.830 (0.552 to 6.070, P=0.323); embolism or stroke HR 1.413 (0.476 to 4.198, P=0.533). Stratified composite endpoints also did not differ. | Not calculable from report | No significant difference | Estimates with confidence intervals reported by the source. 529 patients. |
|  |  |  | Comparative safety — Bleeding; all-cause death | All-cause death HR 1.183 (0.491 to 2.855, P=0.708); any bleeding HR 0.921 (0.531 to 1.596, P=0.768); major bleeding HR 0.183 (0.022 to 1.515, P=0.115); minor bleeding HR 0.728 (0.298 to 1.778, P=0.486); trivial bleeding HR 1.491 (0.690 to 3.222, P=0.309). | Not calculable from report | No significant difference | Estimates with confidence intervals reported by the source. |
| **Zhang 2022 [208]** | Amlodipine besylate; irbesartan (round 1). Retrospective cohort, five-dimension evaluation; Single centre, Kunming; 2017-03 to 2021-10; 404 vs 419 for safety; efficacy reported separately for each molecule. | I Originator | Therapeutic efficacy — Mean fall in blood pressure; response rate (fall of at least 10 mmHg or return to normal) | Amlodipine: mean fall 10.24 vs 11.46 mmHg alone and 9.20 vs 16.46 with metoprolol (F=1.069, P=0.363); response 81.9%, 86.2%, 82.1% and 90.2% (chi-square 1.754, P=0.625). Irbesartan: mean fall 11.10 vs 12.02 and 7.99 vs 11.06 (F=1.62, P=0.185); response 84.3%, 83.1%, 90.4% and 90.0% (chi-square 2.182, P=0.536). Both medicines combined: mean fall 12.69 (selected dual), 10.92 (originator dual), 13.17 (selected dual plus metoprolol) and 21.85 (originator dual plus metoprolol), F=3.077, P=0.028; response 77.8%, 85.4%, 86.1% and 97.6%, chi-square 10.043, P=0.018. | Not calculable from report | No significant difference | Verified against the full text. For both significant four-group comparisons the source reports that the pairwise differences lay between the originator dual-therapy group and the originator dual-plus-metoprolol group, and between the selected dual-therapy group and the originator dual-plus-metoprolol group; all other pairwise comparisons were non-significant. Neither significant pair is a comparison of the selected and originator products under the same regimen: one lies within the originator arm and the other crosses regimens. The four-group comparison of mean blood pressure gives F=3.077, P=0.028. Subgroup denominators are not reported alongside the response rates, so no estimate is calculable. |
|  |  |  | Comparative safety — Adverse reactions during admission | Adverse reactions 2/404 vs 3/419, all mild; no between-group difference (P>0.05). | **RR 0.69 (0.12 to 4.12)** | No significant difference | — |
| **Zhang 2023 [279]** | Olanzapine tablets (round 1). Single-arm before-and-after switch; Single centre, Ningbo; 2019-12 to 2022-04; 30. | III (see note) | Therapeutic efficacy — Clinical Global Impressions severity and improvement | CGI-S and CGI-I did not change significantly after substitution and no symptom worsening was observed over at least three months of follow-up. | Not calculable from report | No significant difference | Within-patient before-and-after substitution in 30 patients stable for at least six months. |
|  |  |  | Comparative safety — Dose-adjusted steady-state serum olanzapine concentration; self-reported events and laboratory abnormality | The log-transformed, dose-adjusted serum concentration of the bid-winning product was significantly lower than that of the preceding generic (P<0.05). Some adverse events and mild laboratory abnormalities were recorded after substitution, but no patient withdrew or switched back. | Not calculable from report | Favours originator | Both arms are generics: the product used before substitution won the pilot stage of round one and the product used after won the expansion stage in the province concerned, so this study compares two selected products and not a selected product with an originator. The source concludes that one generic psychotropic medicine may not be therapeutically equivalent to another. This study does not enter the originator-comparator synthesis. |
| **Liang 2024 [718]** | Tenofovir disoproxil fumarate; tenofovir alafenamide (rounds 1 and 7). Prospective cohort, six parallel arms; thesis; Single centre, Zhanjiang; 2022-01 to 2024-01; 107 vs 81; 101 vs 71. | I and III | Therapeutic efficacy — 48-week sustained virological response; HBeAg negative conversion; ALT normalisation; HBsAg clearance | Sustained virological response at 48 weeks: selected tenofovir disoproxil fumarate 48.5% vs originator 66.7%; selected tenofovir alafenamide 52.4% vs non-selected 71.8%. HBeAg negative conversion 19.4% vs 20.8% and 20.3% vs 25.0%; ALT normalisation 86.9% vs 91.4% and 91.2% vs 94.4%. No arm achieved HBsAg clearance. | **RR 0.73 (0.57 to 0.93)** | Favours originator | Post hoc. The source tested every arm against tenofovir amibufenamide only and states so explicitly; it did not test the selected products against their own comparators. Frequencies were reconstructed by the review authors from the reported arm sizes and percentages and chi-square tests applied: chi-square 6.12, P=0.013 (P=0.020 with continuity correction) for the disoproxil pair and chi-square 6.53, P=0.011 (P=0.017 with correction) for the alafenamide pair. The reconstruction carries a rounding uncertainty of one patient and neither difference would survive Bonferroni correction across the fifteen pairwise comparisons available in that study. The arm sizes used (107, 81, 101 and 71) are consistent with every reported percentage but were not read directly from the source and form part of the reconstruction. The alafenamide pair belongs to the non-selected-generic stratum. |
|  |  |  | Comparative safety — Renal function; lipids at 48 weeks | The source states that renal function did not differ between the selected and originator disoproxil arms at 48 weeks (P>0.05). LDL-C, triglycerides and the triglyceride to HDL-C ratio were all higher in the selected alafenamide arm than in the non-selected alafenamide arm (P<0.05). | Not calculable from report | No significant difference | The renal comparison within the originator-comparator pair was tested by the source and showed no difference. The lipid findings concern the alafenamide pair, which belongs to the non-selected-generic stratum. |
| **Luo 2023 [739]** | Metformin (round 3). Retrospective cohort, PSM; Single centre, Beijing; 2021-01-01 to 2021-09-30; 200 vs 200 (direct); 4,077 vs 4,077 (indirect). | I Originator | Therapeutic efficacy — Change in HbA1c and fasting plasma glucose; HbA1c below 7.0% | HbA1c change −0.50 (IQR −1.50 to −0.02) with the selected product vs −0.40 (IQR −1.50 to 0.00) with the originator, Z=−1.157, P=0.247; fasting plasma glucose change −0.63 vs −0.82 mmol/L, Z=−0.778, P=0.473; HbA1c below 7.0% 163/200 (81.50%) vs 155/200 (77.50%), chi-square 0.982, P=0.322. | **RR 1.05 (0.95 to 1.16)** | No significant difference | Arm order verified against the full text: the two arms are of equal size (200 each), so arithmetic recovery cannot establish the order, and the source states the originator attainment as 77.50% and the selected as 81.50%. Direct comparison uses 200 matched pairs; an indirect comparison of 4,077 pairs is reported separately. |
|  |  |  | Comparative safety — ALT, AST and creatinine abnormality | AST abnormality 6/188 (3.19%) vs 8/192 (4.17%), chi-square 0.255, P=0.614; ALT abnormality 10/159 (6.29%) vs 5/162 (3.09%), chi-square 1.848, P=0.174; creatinine abnormality 2/199 (1.00%) vs 2/198 (1.01%), chi-square 0.000, P=0.996. | Not calculable from report | No significant difference | The three safety comparisons use different denominators (188/192, 159/162, 199/198) because patients whose status could not be determined were excluded from each. In the 4,077-pair indirect analysis the originator arm had higher defined daily doses and higher 3- and 6-month persistence, and the selected arm a lower daily cost and higher single and repeat switching rates (all P<0.001); these are utilisation measures and do not determine direction. |
| **Zhu 2024 [749]** | Rivaroxaban (round 5). Thesis; retrospective cohort, 1:2 PSM with Cox regression; Single centre, Yunnan; 2019-01-01 to 2023-06-30; 28 vs 72 (10 mg); 67 vs 34 (15 mg). | I Originator | Therapeutic efficacy — Ischaemic and haemorrhagic stroke, TIA, systemic embolism, myocardial infarction, all-cause death | In both the 10 mg and 15 mg dose groups no efficacy endpoint differed before or after matching; cumulative incidence log-rank P>0.05 throughout and Cox regression showed no increased risk with the generic. | Not calculable from report | No significant difference | 10 mg group 28 generic and 72 originator; 15 mg group 67 generic and 34 originator. |
|  |  |  | Comparative safety — Eight bleeding endpoints | No bleeding endpoint differed before or after matching. | Not calculable from report | No significant difference | — |
| **Yang 2021 [752]** | Pemetrexed (round 1). Retrospective cohort, PSM; Single centre, Beijing; 2019-03 to 2019-12; 38 vs 38 after PSM. | I Originator | Therapeutic efficacy — Objective response and disease control after two cycles | After matching, objective response 10/38 (26%) vs 12/38 (32%), P=0.723, and disease control 34/38 (89%) vs 36/38 (96%), P=0.674. | **RR 0.83 (0.41 to 1.69)** | No significant difference | The matched analysis is the study’s principal comparison. Before matching the arms differed in stage (III or IV in 92% vs 79%, P=0.032), in the proportion receiving palliative or maintenance chemotherapy (P<0.001), in median dose (900 vs 800 mg/m2, P=0.019) and in median number of cycles (5 vs 4, P<0.001). |
|  |  |  | Comparative safety — Adverse events | After matching, adverse events 18/38 (47%) vs 9/38 (24%), P=0.055. Before matching, total adverse events 37/85 (44%) vs 25/97 (26%) (P=0.018), myelotoxicity (P=0.037) and hepatotoxicity (P=0.018) were all more frequent with the selected product. | **RR 2.00 (1.03 to 3.88)** | No significant difference | Note: the calculated interval marginally excludes the null because it is uncorrected, whereas the source reports P=0.055; an uncorrected chi-square on the same counts gives P=0.031 and a continuity-corrected one gives P=0.054. The comparison sits on the threshold and is recorded as not significant, following the source. The pre-matching differences arose in arms that differed in stage, treatment intent, dose and number of cycles, and are not attributed to the medicine. |
| **Li 2023 [753]** | Ceftriaxone sodium for injection (round 5). Retrospective pharmacoeconomic comparison; Single centre paediatrics, Urumqi; 2020-01 to 2022-08; 140 vs 152. | I Originator | Therapeutic efficacy — Clinical response; time to defervescence; treatment course; time to recovery of inflammatory markers | Clinical response (cure plus marked response) 122/140 (87.14%) vs 136/152 (89.47%), chi-square 0.566, P=0.753. Time to defervescence 2.83 +/- 0.86 vs 2.39 +/- 0.62 days, reported t=3.09, P<0.05, longer with the selected product. Treatment course (P=0.183), time to resolution of cough and sputum (P=0.280) and of rales (P=0.400), and time to recovery of WBC, CRP, neutrophil percentage, procalcitonin and interleukin-6 (P=0.286 to 0.724) did not differ. | **RR 0.97 (0.90 to 1.06)** | No significant difference | The primary outcome did not differ. Recalculation of the defervescence comparison from the reported means, standard deviations and group sizes gives t=4.98, not the 3.09 printed by the source; both indicate a significant difference. The cost table of the source prints identical values for length-of-stay cost and investigation cost while giving different test statistics, and its component costs do not sum to the reported total, so its economic figures are not cited. |
|  |  |  | Comparative safety — Adverse reactions | Adverse reactions 8/140 (5.71%): diarrhoea 1, upper eyelid oedema 1, rash or papules 6; versus 2/152 (1.32%): rash 1, granulocytopenia 1. The source reports P<0.05. | **RR 4.34 (0.94 to 20.10)** | Favours originator | Borderline: recalculation gives chi-square 4.28, P=0.039, but the expected counts are below five and Fisher’s exact test gives P=0.052. The calculated risk ratio has an interval that marginally includes the null. The direction as recorded rests on a comparison at the threshold of significance. |
| **Xu 2023 [760]** | Valsartan/amlodipine (round 4). Retrospective cohort; Single centre, Tengzhou; 2021-05 to 2023-05; 100 vs 100. | I Originator | Therapeutic efficacy — Overall response; systolic and diastolic blood pressure | Overall response 87.00% (87/100) vs 92.00% (92/100) (chi-square 1.330, P=0.249); blood pressure fell in both arms (P<0.01) with no between-group difference. | **RR 0.95 (0.86 to 1.04)** | No significant difference | — |
|  |  |  | Comparative safety — Adverse reactions | One case of headache, in the originator arm. | **RR 0.33 (0.01 to 8.09) †** | No significant difference | 0.5 continuity correction applied. |
| **Cheng 2022 [823]** | Budesonide suspension for inhalation (round 5). Prospective randomised allocation; Single centre, Chongqing; 2020-08 to 2021-07; 74 total. | I Originator | Therapeutic efficacy — Time to resolution of dyspnoea, cough, wheeze and rhonchi; FEV1 and PEF; clinical control | Clinical control grading did not differ (Mann–Whitney P=0.326; control/marked/partial/no response 9/18/16/3 with the selected product vs 7/18/16/7 with the originator). Dyspnoea resolved in 1.81 +/- 0.78 vs 2.49 +/- 0.71 days (t=4.386, P<0.001) and rhonchi in 3.64 +/- 0.83 vs 4.38 +/- 0.82 days (t=4.336, P<0.001), both faster with the selected product. FEV1 99.46 +/- 7.54 vs 94.97 +/- 9.51 (t=−2.540, P=0.013), higher with the selected product. Cough (P=0.510), wheeze (P=0.894) and PEF (P=0.085) did not differ. | Not calculable from report | No significant difference | Verified against the full text: Table 3 of the source and its Results section both show the originator value as the lower of the two, and all four reported t values reproduce from the printed means, standard deviations and group sizes. Only the Discussion section states the opposite, and it also transposes the rhonchi values; under the rule adopted for this review the tables govern. All three significant findings therefore favour the selected product, and this study is not an instance of opposing directions. The primary outcome, clinical control grading, did not differ, so no direction is assigned. Analysed sample is 46 and 48, a total of 94. |
|  |  |  | Comparative safety — None reported | The source reports no separate adverse-reaction data. | Not calculable from report | Not applicable | — |
| **Luo 2021 [73]** | Six psychiatric medicines (round 1). Before-and-after cohort; Single centre psychiatric department; Control 2018-03 to 2019-02; intervention 2019-03 to 2020-02; 1,000 vs 1,000. | II Pre-policy baseline | Therapeutic efficacy — Efficacy score; composite effectiveness score | Efficacy and composite effectiveness scores were similar before and after the policy. | Not calculable from report | No significant difference | Before-and-after design, 1,000 per period; six psychiatric medicines pooled. |
|  |  |  | Comparative safety — Adverse reaction score | Adverse reaction scores were similar. | Not calculable from report | No significant difference | — |
| **Yang 2023 [84]** | Ceftriaxone injection (round 5). Before-and-after, random sampling; Single centre, Xi an; Pre 2021-03 to 05; post 2022-03 to 05; 162 vs 207. | II Pre-policy baseline | Therapeutic efficacy — Clinical response, treatment duration, 7-day discharge, inflammatory markers, pathogen clearance, treatment failure | No efficacy index differed between the pre- and post-policy periods (all P>0.05). | Not calculable from report | No significant difference | Before-and-after design; 207 pre-policy and 162 post-policy. |
|  |  |  | Comparative safety — Overall adverse reactions | Overall adverse reaction rate did not differ (P=0.257). | Not calculable from report | No significant difference | — |
| **Gao 2026 [97]** | Meropenem injection (round 7). Retrospective cohort, PSM; Single centre, Wuhan; Selected arm 2023-01 to 2024-12; comparator 2020-01 to 2022-12; 116 vs 116 after PSM. | I Originator | Therapeutic efficacy — Clinical response; 72-h response | Clinical response 73.3% (85/116) vs 70.7% (82/116) (P=0.661); 72-h response 68.1% vs 66.4% (P=0.780). | **RR 1.04 (0.88 to 1.22)** | No significant difference | The two arms are drawn from non-overlapping calendar periods (selected 2023-01 to 2024-12, comparator 2020-01 to 2022-12). |
|  |  |  | Comparative safety — Adverse events | Adverse events 1.8% vs 2.6% (P>0.05). | Not calculable from report | No significant difference | — |
| **Zhao 2022 [112]** | Pantoprazole (round 4). Multicentre retrospective cohort; Four centres, Zhejiang; 2021-01-01 to 2021-07-30; 96 vs 104. | III Non-selected generic | Therapeutic efficacy — Incidence of stress ulcer | Univariable and multivariable analyses found no difference in the prevention of stress ulcer; subgroup analysis suggested possible variation by age. | Not calculable from report | No significant difference | 96 selected and 104 non-selected. Non-selected-generic stratum. |
|  |  |  | Comparative safety — Same endpoint as efficacy | The outcome event is itself the safety event. | Not calculable from report | No significant difference | — |
| **Zhang 2022 [115]** | Amlodipine besylate; irbesartan (round 1). Retrospective cohort, four parallel groups; Single centre, Qujing; 2021-01 to 2021-11; 264 vs 229; 291 vs 263. | III Non-selected generic | Therapeutic efficacy — Treatment response | Response did not differ between the non-selected and selected arms for either molecule (both P>0.05). | Not calculable from report | No significant difference | Four parallel arms: amlodipine 229 non-selected vs 264 selected; irbesartan 263 non-selected vs 291 selected. Non-selected-generic stratum. |
|  |  |  | Comparative safety — None reported | No safety outcome reported. | Not calculable from report | Not applicable | — |
| **Hong 2025 [145]** | Atorvastatin; capecitabine; pregabalin; budesonide. Thesis; before-and-after, four medicines; Single centre, Mudanjiang; 2019-12-10 to 2022-12-31; 133/150; 50/76; 56/75; 241/139. | II Pre-policy baseline | Therapeutic efficacy — LDL-C attainment and fall; ORR/DCR; VAS and time to pain relief; FEV1, FEV1/FVC and response grading | No efficacy index differed for any of the four medicines (all P>0.05). | Not calculable from report | No significant difference | Before-and-after thesis covering four medicines: atorvastatin 150 post vs 133 pre; capecitabine 76 vs 50; pregabalin 75 vs 56; budesonide 139 vs 241. |
|  |  |  | Comparative safety — Adverse reactions for each medicine | Total adverse reaction rates did not differ for any medicine. | Not calculable from report | No significant difference | — |
| **Xiao 2022 [194]** | Amlodipine besylate (round 1). Before-and-after retrospective cohort; Single centre, Leshan; Comparator 2017-03 to 2019-08; selected 2019-12 to 2022-03; 387 vs 395. | III Non-selected generic | Therapeutic efficacy — Treatment response | Response 96.90% (375/387) with the selected product vs 97.97% (387/395) with the non-selected product. | **RR 0.99 (0.97 to 1.01)** | No significant difference | Non-selected-generic stratum. Neither arm is identified by manufacturer and the province concerned published its awards only within its procurement platform, so manufacturer-level verification is not obtainable; the study is removed in one sensitivity analysis. |
|  |  |  | Comparative safety — Adverse reaction reporting | No adverse reaction was reported in either arm. | Not calculable from report | No significant difference | Not estimable: no events in either arm. |
| **Chen 2021 [198]** | Amlodipine besylate (round 1). Prospective allocation by visit sequence; Single centre, Kunming; 2020-01 to 2021-01; 50 vs 50. | I Originator | Therapeutic efficacy — Blood pressure, overall response, blood-pressure variability, heart rate | Overall response 44/50 (88%) vs 46/50 (92%), chi-square 0.444, P>0.05; fall in whole-day systolic pressure 15.02 +/- 7.93 vs 14.97 +/- 6.00 and diastolic 10.92 +/- 5.65 vs 10.10 +/- 4.67, neither differing between arms. Control of morning systolic blood-pressure variability was better with the imported originator (P<0.05); morning diastolic variability and whole-day and night-time variability did not differ. Heart rate 74.02 +/- 7.87 vs 71.66 +/- 7.54 (P=0.155) and its variability (P=0.639) did not differ. Daily dose 5 mg in 42 vs 46 and 10 mg in 8 vs 4 (chi-square 1.515, P=0.218). | **RR 0.96 (0.84 to 1.09)** | No significant difference | The primary outcome did not differ. Morning systolic blood-pressure variability is a secondary measure of blood-pressure control, not a safety outcome, and does not determine direction. Imported originator comparator. The source does not name the manufacturer of the selected arm; the same hospital, period and approval number appear in another included study whose full text does name the company, so the manufacturer is recorded as probable rather than verified. |
|  |  |  | Comparative safety — Adverse reactions | Adverse reactions 3/50 (6.00%) vs 5/50 (10.00%), chi-square 0.543, P=0.461. By type: fatigue 0 vs 1, oedema 2 vs 2, dizziness 1 vs 1, palpitation 0 vs 2; no flushing or rash in either arm. No patient stopped treatment and liver and renal function did not change. | **RR 0.60 (0.15 to 2.38)** | No significant difference | — |
| **Zhang 2022 [211]** | Moxifloxacin injection (round 3). Retrospective three-arm cohort with decision-tree analysis; Single centre, Hefei; 2018-01 to 2021-06; 74 vs 72 (vs 75 non-selected). | I Originator | Therapeutic efficacy — Clinical response | Response did not differ across the three arms (P>0.05). | Not calculable from report | No significant difference | Three arms: selected 74, non-selected 75, imported originator 72. Only the selected-versus-imported-originator pair is used. |
|  |  |  | Comparative safety — None reported | No safety outcome reported. | Not calculable from report | Not applicable | — |
| **Yang 2023 [224]** | Moxifloxacin sodium chloride injection (round 3). Retrospective cohort; Single centre, Huizhou; 2020-01 to 2022-12; 54 vs 21. | I Originator | Therapeutic efficacy — Treatment response | Response 88.89% (48/54) vs 85.71% (18/21) (P>0.05). | **RR 1.04 (0.85 to 1.26)** | No significant difference | Imported originator comparator. |
|  |  |  | Comparative safety — Adverse reactions | Adverse reactions 7.41% (4/54) vs 4.76% (1/21) (P>0.05). | **RR 1.56 (0.18 to 13.12)** | No significant difference | — |
| **Huang 2022 [606]** | Entecavir (round 1). Registry-based cohort (switch vs no switch); Single centre, Zhengzhou; 2019-01-01 to 2021-05-18; 156 vs 104. | III Non-selected generic | Therapeutic efficacy — Complete virological response at 24 and 48 weeks; ALT normalisation; HBeAg negative conversion and seroconversion | Complete virological response at 24 weeks 96.79% (151/156) vs 97.12% (101/104), P=1.000; at 48 weeks both arms reached 100%. ALT normalisation chi-square 0.157, P=0.692 at 24 weeks and 2.316, P=0.128 at 48 weeks. HBeAg negative conversion 3 vs 2 at 24 weeks and 9 vs 4 at 48 weeks; seroconversion 0 vs 2 and 3 vs 1; none significant. | **RR 1.00 (0.95 to 1.04)** | No significant difference | Non-selected-generic stratum: both arms were already in complete virological response on a domestic product, and the ceiling effect limits the sensitivity of the comparison. |
|  |  |  | Comparative safety — Adverse drug reactions | No adverse reaction occurred in either arm. | Not calculable from report | No significant difference | Not estimable: no events in either arm. |
| **Zhang 2022 [607]** | Cefuroxime sodium for injection (round 5). Retrospective cohort; Single centre, Jiaxing; 2021-01 to 2021-12; 148 vs 148. | I Originator | Therapeutic efficacy — Prophylactic response; postoperative white cell count, neutrophil percentage, CRP, peak temperature, length of stay | Prophylactic response 144/148 (97.3%) vs 142/148 (96.0%), chi-square 0.414, P=0.750. Neutrophil percentage 69.65 (IQR 59.83 to 75.73) vs 63.75 (IQR 54.98 to 74.85), Z=−2.314, P=0.021, higher with the selected product. White cell count (P=0.075), CRP (P=0.118), peak temperature (P=0.760) and length of stay (P=0.648) did not differ. | **RR 1.01 (0.97 to 1.06)** | No significant difference | The direction of the neutrophil comparison is now established from the full text; the values are medians with interquartile ranges and no estimate is calculable for it. Neutrophil percentage is a secondary laboratory marker and does not determine direction; the primary outcome, prophylactic response, did not differ. |
|  |  |  | Comparative safety — Adverse reactions | Adverse reactions 14/4,512 (0.31%) vs 12/6,319 (0.19%), chi-square 1.593, P=0.207. | **RR 1.63 (0.76 to 3.53)** | No significant difference | Different denominator: the source states that the matched sample was too small for a safety comparison and therefore used all patients who received either product at the same hospital between 1 January 2020 and 30 June 2023. The safety denominators (4,512 and 6,319) are therefore a different population from the 148 matched pairs used for efficacy. One event in the selected arm was assessed as serious; all others were ordinary. |
| **Liu 2024 [618]** | Cefazolin, cefuroxime and ceftazidime injections (rounds 3 and 5). Multicentre retrospective cohort, PSM; 19 centres, Kunming; 2020-01 to 2022-09; 394/394; 472/472; 504/590. | III Non-selected generic | Therapeutic efficacy — Clinical and 72-h response, bacterial clearance, recovery of infection markers, for three medicines | Cefazolin clinical response 377/394 (95.7%) vs 378/394 (95.9%), chi-square 0.032, P=0.859; cefuroxime 461/472 (97.7%) vs 458/472 (97.0%), P=0.543; ceftazidime 487/504 (96.6%) vs 568/590 (96.3%), P=0.752. For ceftazidime, recovery of C-reactive protein to the reference range was more frequent with the selected product, 46 (46.9%) vs 31 (27.9%), chi-square 8.084, P=0.004. No other efficacy index differed for any of the three medicines. | **RR 1.00 (0.97 to 1.03)** | No significant difference | Three medicines in one report; the estimate shown is for cefazolin clinical response. Non-selected-generic stratum, so this study does not enter the main synthesis. The primary outcome did not differ for any medicine; the CRP recovery finding is a secondary marker. |
|  |  |  | Comparative safety — Adverse drug reactions; laboratory abnormality, for three medicines | Adverse drug reaction rates did not differ for any medicine (cefazolin 4/394 vs 1/394, P=0.370). Platelet reduction was more frequent with selected cefazolin, 35 (20.7%) vs 8 (7.1%), chi-square 9.566, P=0.002, and eosinophil elevation more frequent with selected ceftazidime, 12 (5.3%) vs 5 (1.9%), chi-square 4.209, P=0.040. No other laboratory index differed. | **RR 4.00 (0.45 to 35.63)** (estimate is for the outcome named above, which did not differ; the direction rests on a further comparison for which counts are not reported) | Favours originator | Denominators not reported: the source states that patients whose status could not be determined were excluded from each laboratory index, so the denominator differs by index and none is given. Deriving them from the printed percentages is not permitted under the rules adopted for this review, and no estimate is entered for the laboratory comparisons; the estimate shown is for the adverse drug reaction comparison, which did not differ. The cefazolin AST entry is printed as 45 (6.3%), which is not compatible with a group of 394, and is not cited. Both significant safety findings favour the comparator, so this is not an instance of opposing directions. |
| **Wang 2023 [720]** | Clopidogrel (round 1). Retrospective cohort with regression adjustment; Single centre, Linyi; 2018-01-01 to 2021-12-31; 705 vs 915. | I Originator | Therapeutic efficacy — All-cause death, cardiac death, MACCE, stroke | All-cause death (P=0.405), cardiac death (P=0.309), MACCE (P=0.296) and stroke (P=0.466) did not differ; regression found no association between clopidogrel type and MACCE risk. | Not calculable from report | No significant difference | 705 domestic and 915 imported originator. The arms differed significantly in infarction type and concomitant medication and only regression adjustment was applied. |
|  |  |  | Comparative safety — Same endpoints as efficacy | The outcome events are themselves the safety events. | Not calculable from report | No significant difference | — |
| **Peng 2022 [X1]** | Atorvastatin calcium tablets (round 1). Retrospective comparison; Single centre, Shanghai; 2020-04 to 2021-03; 36 vs 37. | I Originator | Therapeutic efficacy — LDL-C target attainment at 4-6 weeks; change in lipid indices | Attainment 50.0% vs 43.2% (P>0.05); no difference in the magnitude of lipid change. | **RR 1.16 (0.71 to 1.89)** | No significant difference | Counts recovered exactly from the reported percentages and group sizes (36 selected, 37 originator). Entered through citation searching. |
|  |  |  | Comparative safety — Adverse events | Adverse events did not differ (P>0.05). | Not calculable from report | No significant difference | — |
| **Yuan 2024 [X2]** | Cefuroxime sodium for injection (round 5). Retrospective cohort; Single centre, Beijing; 2021-07 to 2022-05; 280 total. | I Originator | Therapeutic efficacy — Operative blood loss, antibiotic course, length of stay, 24-h WBC and neutrophil ratio, fever and postoperative infection | No index differed (all P>0.05). | Not calculable from report | No significant difference | 280 patients; imported originator comparator. Entered through citation searching. |
|  |  |  | Comparative safety — Adverse reactions | Adverse reaction rates were low in both arms and did not differ. | Not calculable from report | No significant difference | — |
| **Wang 2023 [X3]** | Linezolid tablets (round 3). Multicentre retrospective cohort, PSM; Multicentre; 2020-05-01 to 2021-12-31; 95 vs 59. | I Originator | Therapeutic efficacy — Total dose, treatment course, clinical response at 72 hours; daily dose by infection site | Clinical response 48/60 (80.00%) vs 32/35 (91.43%), treatment course 9.13 +/- 4.87 vs 8.86 +/- 5.10 days and total dose 10.9 +/- 5.91 vs 9.57 +/- 6.57 g did not differ (all P>0.05). Daily dose was higher with the selected product in respiratory infection (1.18 +/- 0.10 vs 1.06 +/- 0.26 g) and at other sites (1.20 +/- 0 vs 0.94 +/- 0.32 g), both P<0.05; in skin and soft-tissue infection both arms received 1.20 g. | **RR 0.88 (0.74 to 1.03)** | No significant difference | Analysed sample is 60 and 35 after 1:3 propensity score matching, a total of 95; the pre-matching total was 154 (95 and 59). Daily dose is a utilisation measure and does not determine direction. Before matching the arms differed significantly in sex, insurance type, number of comorbidities and treating department (infectious diseases 6/95 vs 30/59). |
|  |  |  | Comparative safety — Hepatic injury, pancytopenia, nausea or vomiting; concomitant medicines used for adverse reactions | Hepatic injury 0/60 (0%) vs 3/35 (8.57%), P<0.05. Pancytopenia 3/60 (5.00%) vs 6/35 (17.14%), nausea or vomiting 1/60 vs 1/35, hepatoprotective drugs 18/60 (30.00%) vs 16/35 (45.71%), antihistamines 2/60 vs 2/35 and gastric protectants 32/60 vs 21/35 all did not differ. | **RR 0.08 (0.00 to 1.59) †** | Favours procured product | Note: with no events in the selected arm the 0.5-corrected interval is conservative and includes the null, whereas Fisher’s exact test on the raw counts gives P=0.046, consistent with the significance reported by the source. The direction is retained on that basis. The originator product was itself a winning supplier of round three, so this comparison is between a selected generic and a selected originator. Entered through citation searching. |
| **Wu 2024 [X4]** | Atorvastatin calcium tablets (round 1). Retrospective cohort; Single centre; 2023-05 to 2023-12; 44 vs 29. | I Originator | Therapeutic efficacy — Percentage fall in TC, TG, LDL-C and non-HDL-C after one month | Lipids fell significantly in both arms with no between-group difference in the magnitude of the fall (P>0.05). | Not calculable from report | No significant difference | 44 selected and 29 originator. Entered through citation searching. |
|  |  |  | Comparative safety — Hepatic enzyme abnormality | Hepatic enzyme abnormality did not differ significantly. | Not calculable from report | No significant difference | — |
| **Li 2024 [X5]** | Ticagrelor tablets (round 3). Retrospective cohort; Single centre, Nanjing; Pre 2020-01 to 2020-11; post 2020-12 to 2021-11; 88 vs 93. | I Originator | Therapeutic efficacy — ADP inhibition and attainment during admission; GOS and mRS at six months | ADP inhibition 62.35 +/- 27.62 vs 59.01 +/- 29.05% and attainment 64.86% vs 60.00% (P>0.05); GOS and mRS did not differ. | Not calculable from report | No significant difference | Before-and-after design across the procurement boundary (88 post-policy and 93 pre-policy). Event analysis in the source uses an unruptured-aneurysm subgroup rather than the full arms, so no estimate is entered. Entered through citation searching. |
|  |  |  | Comparative safety — Ischaemic events; bleeding events | Ischaemic events 5.88% vs 4.71%; bleeding 18.82% vs 15.29% (P>0.05). | Not calculable from report | No significant difference | — |
| **Si 2024 [X6]** | Febuxostat tablets (round 3). Multicentre retrospective cohort, 1:1 PSM; Three centres, Beijing; 2014-01 to 2022-02; 57 vs 57 after PSM. | I Originator | Therapeutic efficacy — Serum urate below 300 umol/L at 12 weeks; percentage fall from baseline | Neither attainment nor the percentage fall differed (both P>0.05). | Not calculable from report | No significant difference | 57 matched pairs. Entered through citation searching. |
|  |  |  | Comparative safety — Adverse drug reactions | Adverse reaction rates did not differ; no serious adverse reaction was reported in either arm. | Not calculable from report | No significant difference | — |
| **Xing 2025 [X7]** | Atorvastatin (round 1). Multicentre retrospective cohort, 1:1 PSM; 16 centres; 2020-04-01 to 2022-03-31; 39,742 vs 39,742 (treatment patterns); 3,610 vs 3,610 (efficacy). | I Originator | Therapeutic efficacy — Absolute and percentage fall in LDL-C, non-HDL-C, TC and TG; LDL-C fall of at least 50% | No efficacy outcome differed. LDL-C absolute fall 0.10 (IQR 0-0.73) vs 0.06 (IQR 0-0.85) mmol/L, P=0.414; percentage fall 4.86% vs 3.00%, P=0.263; LDL-C fall of at least 50% 350/3,610 (9.7%) vs 329/3,610 (9.1%), P=0.397. | Not calculable from report | No significant difference | Verified against the full text: the counts are reported by the source. Arm order uncertain for the efficacy table: the Results text of the source states that the absolute fall was 0.06 mmol/L with the generic and 0.10 mmol/L with the branded product, whereas its Table 2 prints these values in the opposite column order from the safety table. The two arms cannot be assigned with certainty for this outcome and no estimate is entered; the comparison is not significant either way. The figure of 359,159 is the number of patients reviewed before matching; a separate propensity model was fitted for each outcome, so matched sizes differ by outcome (efficacy 3,610 pairs). |
|  |  |  | Comparative safety — Hepatotoxicity; creatine phosphokinase; statin-associated muscle symptoms; fasting glucose; new-onset diabetes | AST at least three times the upper limit 96/6,335 (1.5%) with the generic vs 64/6,335 (1.0%) with the branded product, P=0.014. ALT at least three times the upper limit 104 vs 90 (P=0.335) and both enzymes together 49 vs 42 (P=0.529) did not differ; drug-induced liver injury 17/52,765 in each arm (P=1.000); hepatoprotective drugs prescribed 908/52,765 (1.7%) vs 1,054/52,765 (2.0%), P=0.001. CPK four to ten times the upper limit 32/4,220 vs 29/4,220 (P=0.787) and above ten times 18 vs 15 (P=0.728) did not differ. Fasting glucose rose 0.01 vs 0.16 mmol/L (P=0.009); fasting glucose at least 7.0 mmol/L 387/2,291 (16.9%) vs 415/2,291 (18.1%), P=0.286; new-onset diabetes 664/28,640 (2.3%) vs 1,244/28,640 (4.3%), P<0.001. | **RR 1.50 (1.10 to 2.05)** | Opposing directions within cohort | Verified against the full text: all safety counts are reported by the source and the arm order in its safety table is generic first, confirmed by the accompanying text. Significant results point in opposite directions within one cohort (AST elevation favours the branded product; hepatoprotective prescribing, fasting glucose and new-onset diabetes favour the generic), so no direction is assigned. The estimate shown is for AST elevation. The Results text of the source prints P<0.001 for the fasting-glucose threshold comparison while its Table 3 gives P=0.286; the table value is used. The Chinese companion report of the same data source uses a different matched set and is listed rather than excluded, with the English article as the main report. |
| **Zhang 2020 [248]** | Clopidogrel bisulfate tablets (round 1). Prospective randomised allocation; Single centre; 2016-01 to 2019-01; 53 vs 53. | I Originator | Therapeutic efficacy — Overall response; frequency of angina; platelet aggregation | Overall response 98.11% (52/53) in both arms (chi-square 0.000, P=1.000); frequency of angina and platelet aggregation did not differ (P>0.05). | **RR 1.00 (0.95 to 1.05)** | No significant difference | Imported originator comparator. The recruitment window is stated only to the month; read as running to the end of January 2019, the overlap with the post-announcement period is about eight weeks, and the study is graded at high risk of exposure misclassification and removed first in the corresponding sensitivity analysis. |
|  |  |  | Comparative safety — Total adverse reactions | Adverse reaction rate did not differ (chi-square 0.059, P=0.807). | Not calculable from report | No significant difference | Counts are not entered for this comparison. |
| **Luo 2023b [X8]** | Glimepiride tablets (round 2). Retrospective cohort, PSM; Single centre, Beijing; Originator 2019-01-01 to 2019-12-30; selected 2020-10-01 to 2021-09-30; 81 vs 81 after PSM for direct indices; 1,350 vs 1,350 for indirect indices. | I Originator | Therapeutic efficacy — Change in HbA1c and fasting plasma glucose; HbA1c below 7.0% | Change in HbA1c −0.40 (IQR −1.05 to 0.05) with the selected product vs −0.30 (IQR −0.80 to 0.20) with the originator, Z=−1.470, P=0.142; change in fasting plasma glucose −0.59 (IQR −2.11 to 0.26) vs −0.43 (IQR −1.70 to 0.64), Z=−0.502, P=0.615. HbA1c below 7.0% was reached by 50 of 81 (61.73%) with the selected product against 34 of 81 (41.98%) with the originator, chi-square 6.330, P=0.018. | **RR 1.47 (1.08 to 2.00)** | No significant difference | Note: the interval shown is for attainment of the HbA1c target, which is significant, whereas the direction recorded for the domain follows the primary measures, which are not. The primary efficacy measures, the changes in HbA1c and fasting plasma glucose, did not differ; attainment of the HbA1c target is a secondary measure and the source concludes that the direct indices did not differ. Direct comparison uses 81 matched pairs; an indirect comparison of 1,350 pairs is reported separately for utilisation and cost. The two arms are drawn from non-overlapping calendar windows: the originator arm from January to December 2019 and the selected arm from October 2020 to September 2021. |
|  |  |  | Comparative safety — AST, ALT and creatinine abnormality after treatment | AST abnormality 6/78 (7.69%) vs 5/78 (6.41%), chi-square 0.098, P=0.754; ALT abnormality 5/67 (7.46%) vs 4/70 (5.71%), chi-square 0.170, P=0.680; creatinine abnormality 3/81 (3.70%) vs 2/80 (2.50%), chi-square 0.194, P=0.660. The three indices did not differ before treatment (P=0.509, 0.234, 0.992). | **RR 1.20 (0.38 to 3.77)** | No significant difference | The three safety comparisons use different denominators (78/78, 70/67, 80/81). In the 1,350-pair indirect analysis the selected arm had a higher volume and defined daily dose (both 20.34% higher) and a daily cost 98.20% lower; the rate of dose escalation was higher with the selected product (4 mg to 6 mg 6.44% vs 4.44%, chi-square 5.245, P=0.022; any escalation 9.04% vs 6.96%, chi-square 3.945, P=0.047). CONFLICT: the abstract and the discussion state that persistence at six and nine months was significantly lower with the selected product, while the Results section states that it was significantly higher; the corresponding table could not be read and no direction is assigned to that outcome. Utilisation measures do not determine direction. |
| **Li 2023b [X9]** | Glimepiride tablets (round 2). Retrospective cohort, PSM; Three centres, Shanghai, Zhengzhou and Wuhu; 2020-01-01 to 2021-12-31; 103 vs 103 after PSM (229 vs 113 before). | I Originator | Therapeutic efficacy — Change in HbA1c (primary); change in fasting plasma glucose and HbA1c below 7.0% (secondary) | After propensity score matching (103 pairs), the reduction in HbA1c, the reduction in fasting plasma glucose and the proportion reaching HbA1c below 7.0% did not differ between arms (all P>0.05). Treatment lasted at least six months in both arms and the daily dose was at least 2 mg. | Not calculable from report | No significant difference | The source reports these comparisons as not significant without giving the underlying counts or summary statistics, so no estimate is calculable. Baseline sex, age, body mass index, glucose level and diabetes duration did not differ after matching. |
|  |  |  | Comparative safety — Hypoglycaemic events; raised ALT or AST; eGFR below 60 mL/min/1.73 m2 | The rate of hypoglycaemic events, the proportion with raised ALT or AST and the proportion with eGFR below 60 mL/min/1.73 m2 did not differ between arms (all P>0.05). Persistence and switching rates also did not differ. | Not calculable from report | No significant difference | Counts are not reported for the safety outcomes. Originator identified by brand (Amaryl, 2 mg tablets); the selected arm pools three manufacturers (Shengping, Liyiping, Dibei), so the findings characterise those three products in aggregate. |

**Notes**

† 0.5 continuity correction applied because one arm had no events.

Denominators are not uniform within a study: where a source applied a separate propensity model to each outcome, or reported adverse events against pre-matching group sizes and laboratory abnormalities against matched pairs, the denominator used for each estimate is stated in the Notes column.

Three studies do not use the patient as the unit of analysis: one reports drug administrations, one reports treatment courses, and one reports serum concentration determinations. These are identified in the Notes column and their rates are reporting rates, not incidences.

Where the values reported by a source are internally inconsistent, the Notes column states the inconsistency and how it was handled. No estimate is entered where the inconsistency cannot be resolved from the source itself.
