# Supplementary material for: Clinical efficacy and safety of medicines procured through China’s national volume-based procurement policy: a systematic review of 81 studies
Source: Front Public Health. 2026 Oct 15;14:1820505. doi: 10.3389/fpubh.2026.1820505 (PMC13716852; doi:10.3389/fpubh.2026.1820505)
Supplement: Supplementary file 5 [file Table_4.docx]

**Supplementary Table S4. GRADE assessment of certainty for each outcome domain.**

Certainty was rated for the originator-comparator stratum, which constitutes the main analysis. All included studies except one randomised trial are non-randomised, so each domain begins at low certainty and is rated further down where the considerations below apply. No domain was rated up: no dose-response relationship was demonstrable, effect sizes were small where they were estimable at all, and plausible confounding would not have acted to reduce an observed effect.

| **Outcome domain** | **Summary of findings** | **Risk of bias** | **Inconsistency** | **Indirectness** | **Imprecision** | **Publication bias** | **Certainty** |
| --- | --- | --- | --- | --- | --- | --- | --- |
| Therapeutic efficacy | 62 counted comparisons, 60 showing no difference and two to which no direction was assigned; none has an assigned direction. One further comparison is reported but not counted because its source did not test it | Very serious: 21 studies at high risk of exposure misclassification, 12 without matching or adjustment, 15 with incomplete or irreconcilable outcome reporting | Not serious: the direction is concordant in 60 of 62 | Serious: more than forty medicines across eight therapeutic classes are combined; no single medicine is supported by more than nine studies | Very serious: an effect estimate is unavailable for 42 of the 81 studies; two studies compared arms of fewer than 40 patients; one reported difference rests on a subgroup of five patients per arm and was not allowed to determine direction | Serious: 23 studies arise from five groups or platforms; no protocol or registration could be located for any included study | Very low |
| Comparative safety | 65 comparisons, 55 showing no difference, four favouring the originator, three favouring the selected product and three to which no direction was assigned | Very serious: as above, and six studies report test statistics that cannot be reproduced from their own data | Serious: ten of 65 comparisons show a difference, in both directions, and no explanation by medicine, class or design accounts for them | Serious: as above | Very serious: event counts are small in most studies; denominators are not uniform within or across studies | Serious: as above | Very low |
| Pharmacokinetics | 2 comparisons, one favouring the originator and one showing no difference | Serious: both are single-centre retrospective cohorts | Not assessable with two studies | Serious: two different medicines in one therapeutic class | Very serious: the evidence base is two studies | Serious: one of the two belongs to a platform contributing other included studies | Very low |
| Economic outcomes | Acquisition costs were 44% to more than 90% lower in every study reporting them; total treatment cost and cost-effectiveness ratios also favoured the selected product where measured | Serious: costing was restricted to direct acquisition costs in most studies | Not serious: the direction is concordant in every study | Serious: no study discounted or extended the horizon beyond the treatment episode | Not serious | Not serious | Low |

Publication bias could not be assessed by funnel plot because no outcome was reported as a commensurable estimate by enough studies. It was assessed qualitatively within GRADE: no included study could be linked to a prospective registration, 23 of 81 arise from five groups or platforms, and one study did not test the comparison of interest although it reported the underlying rates. These features are consistent with selective reporting and the domain was rated down accordingly.

The certainty of evidence for efficacy, comparative safety and pharmacokinetics is very low. This means that the true effect is likely to be substantially different from the estimate; the finding that most comparisons show no difference should not be read as evidence of equivalence.

**Supplementary Table S7. Sensitivity analyses.**

Eleven sensitivity analyses were conducted on the 81 included studies. The first two are those specified in the original submission and are reported first; the original specified restriction to studies at low or moderate overall risk of bias, implemented here as the exclusion of any study rated high in any domain, because risk of bias is now assessed by domain rather than overall. The remaining nine were added during this revision and are identified as not pre-specified in Section 2.7. Direction of findings refers to the pattern of the effect-direction tally within each outcome domain.

| **No.** | **Analysis** | **Studies removed** | **Studies remaining** | **Direction of findings** |
| --- | --- | --- | --- | --- |
| S7.1 | Exclusion of any study rated high in any risk-of-bias domain | 49 studies rated high in at least one of the eight domains | 32 | No efficacy comparison retains a direction; the safety domain retains one in each direction. Pattern unchanged and more symmetrical |
| S7.2 | Restriction to multicentre studies | 62 single-centre studies | 19 | The balance of safety signals reverses: of four comparisons retaining a direction, three favour the selected product and one the originator. No efficacy comparison retains a direction |
| S7.3 | Restriction to the originator-comparator stratum | 12 studies in the pre-policy, non-selected-generic or within-patient strata | 69 | Unchanged |
| S7.4 | Exclusion of studies at high risk of exposure misclassification | 21 studies graded high in D1 | 60 | Unchanged |
| S7.5 | Exclusion of studies whose arms are drawn from non-overlapping calendar windows | 10 studies graded high in D2 | 71 | Unchanged |
| S7.6 | Exclusion of studies without matching or adequate adjustment | 12 studies graded high in D4 | 69 | Unchanged |
| S7.7 | Exclusion of the study extracted from an abstract only | 1 study graded abstract-only in D8 | 80 | Unchanged |
| S7.8 | Exclusion of studies with incomplete or irreconcilable outcome reporting | 15 studies graded high in D6 | 66 | Unchanged |
| S7.9 | Retention of a single study per research group or platform | 23 studies from five groups reduced to five | 63 | Unchanged; medicines represented fall disproportionately in the cardiovascular and anti-infective classes |
| S7.10 | Exclusion of comparisons tested only by the review authors | 1 comparison reconstructed post hoc (six-arm nucleos(t)ide study) | 68 | Efficacy tally unchanged: that comparison is already excluded from the tally in the main analysis |
| S7.11 | Direction assigned wherever any outcome of a study’s principal analysis differs significantly, rather than by the primary outcome alone | None; the rule is changed rather than studies removed | 81 | Efficacy domain becomes three favouring the originator, three favouring the selected product, three with no direction assigned and 54 with no difference, a total of 63 — the comparisons that report an efficacy outcome, rather than the 62 counted in Table 2; safety domain unchanged. The two directions remain in balance |

S7.10 is the most consequential for interpretation. The efficacy domain contains exactly one comparison with an assigned direction, and that direction rests on a chi-square test performed by the review authors on frequencies reconstructed from the arm sizes and percentages published by the source, which did not test the comparison itself. When that comparison is excluded, no study in the efficacy domain of the main stratum has an assigned direction.
