# Supplementary material for: Clinical efficacy and safety of medicines procured through China’s national volume-based procurement policy: a systematic review of 81 studies
Source: Front Public Health. 2026 Oct 15;14:1820505. doi: 10.3389/fpubh.2026.1820505 (PMC13716852; doi:10.3389/fpubh.2026.1820505)
Supplement: Supplementary file 6 [file Table_5.docx]

**Supplementary Table S5. PRISMA 2020 checklist.**

Item 24c reports all amendments made after registration and runs to sixteen entries. PROSPERO Version 3.0 was published on 14 September 2026 and Version 3.1, a minor amendment correcting four figures and one statement recorded in Version 3.0, on 15 September 2026.

| **Section and topic** | **Item** | **Checklist item** | **Location where item is reported** |
| --- | --- | --- | --- |
| TITLE | 1 | Identify the report as a systematic review. | Title, p. 1 |
| ABSTRACT | 2 | See the PRISMA 2020 for Abstracts checklist. | Abstract, p. 1 |
| Rationale | 3 | Describe the rationale for the review in the context of existing knowledge. | Section 1 |
| Objectives | 4 | Provide an explicit statement of the objective(s) the review addresses. | Section 1, final paragraph |
| Eligibility criteria | 5 | Specify the inclusion and exclusion criteria and how studies were grouped for the syntheses. | Section 2.2; grouping for synthesis in Sections 2.7 and 3.3 |
| Information sources | 6 | Specify all databases, registers, websites, organisations, reference lists and other sources searched, with the date each was last searched. | Section 2.3; Supplementary Table S1 |
| Search strategy | 7 | Present the full search strategies for all databases, registers and websites, including any filters and limits used. | Supplementary Table S1 |
| Selection process | 8 | Specify the methods used to decide whether a study met the inclusion criteria, including how many reviewers screened each record and each report retrieved, whether they worked independently, and details of any automation tools. | Section 2.4; Supplementary Table S6. Titles and abstracts were screened by one reviewer and checked by a second, as specified at registration; the reliability of that stage was assessed post hoc in a random sample of 500 records (raw agreement 89.2%, Cohen’s kappa 0.78, 95% CI 0.73 to 0.84). All 862 reports reaching full-text assessment were screened in duplicate by two reviewers working independently against a fixed eight-question sequence, with 18 disagreements resolved by discussion. A random sample of 400 of the 781 excluded records was independently re-assessed against the full text; judgements agreed with the recorded decision in 393 cases, and the seven discrepancies were resolved by discussion without change. |
| Data collection process | 9 | Specify the methods used to collect data from reports, including how many reviewers collected data, whether they worked independently, and any processes for confirming data. | Section 2.5 |
| Data items | 10a | List and define all outcomes for which data were sought. | Sections 2.2, 2.5 and 2.7 |
|  | 10b | List and define all other variables for which data were sought; describe assumptions about missing or unclear information. | Section 2.5 |
| Study risk of bias assessment | 11 | Specify the methods used to assess risk of bias, including the tools used, how many reviewers assessed each study and whether they worked independently. | Section 2.6 (eight domains, three developed for this review); Supplementary Table S2 |
| Effect measures | 12 | Specify for each outcome the effect measure(s) used in the synthesis or presentation of results. | Section 2.7 (direction of effect as the standardised metric); Supplementary Table S3 |
| Synthesis methods | 13a | Describe the processes used to decide which studies were eligible for each synthesis. | Sections 2.7 and 3.3 (comparator strata and outcome domains) |
|  | 13b | Describe any methods required to prepare the data for presentation or synthesis. | Section 2.5 (post hoc reconstruction of frequencies, declared as such); Section 2.7 |
|  | 13c | Describe any methods used to tabulate or visually display results of individual studies and syntheses. | Section 2.7 (effect-direction tables and harvest plot); Table 2; Figure 2 |
|  | 13d | Describe any methods used to synthesize results and the rationale for the choice(s). | Section 2.7 (synthesis without meta-analysis, following SWiM; rationale given) |
|  | 13e | Describe any methods used to explore possible causes of heterogeneity among study results. | Section 2.7 (stratification by comparator type, therapeutic area and drug class) |
|  | 13f | Describe any sensitivity analyses conducted to assess robustness of the synthesized results. | Section 2.7 (eleven analyses; the two specified in the original submission are reported first and the remaining nine are identified as not pre-specified); Supplementary Table S7 |
| Reporting bias assessment | 14 | Describe any methods used to assess risk of bias due to missing results in a synthesis. | Section 2.7; Section 3.11 (assessed qualitatively within GRADE; funnel plots not possible); Supplementary Table S4 |
| Certainty assessment | 15 | Describe any methods used to assess certainty in the body of evidence for an outcome. | Section 2.7; Supplementary Table S4 |
| Study selection | 16a | Describe the results of the search and selection process, ideally using a flow diagram. | Section 3.1; Figure 1 |
|  | 16b | Cite studies that might appear to meet the inclusion criteria but were excluded, and explain why. | Supplementary Table S6 (full-text eligibility decision for all 862 reports, with the reason for each exclusion). No study awaited classification. |
| Study characteristics | 17 | Cite each included study and present its characteristics. | Sections 3.2 and 3.3; Table 1 |
| Risk of bias in studies | 18 | Present assessments of risk of bias for each included study. | Section 3.9; Supplementary Table S2 |
| Results of individual studies | 19 | For all outcomes, present for each study the summary statistics for each group and an effect estimate with its precision. | Supplementary Table S3; Figure 2 |
| Results of syntheses | 20a | For each synthesis, briefly summarise the characteristics and risk of bias among contributing studies. | Sections 3.4, 3.5 and 3.9 |
|  | 20b | Present results of all statistical syntheses; if comparing groups, describe the direction of the effect. | Sections 3.4 to 3.7; Table 2; Figure 2 |
|  | 20c | Present results of all investigations of possible causes of heterogeneity. | Sections 3.6 and 4.4 |
|  | 20d | Present results of all sensitivity analyses. | Section 3.10; Supplementary Table S7 |
| Reporting biases | 21 | Present assessments of risk of bias due to missing results for each synthesis assessed. | Section 3.11; Supplementary Table S4 |
| Certainty of evidence | 22 | Present assessments of certainty in the body of evidence for each outcome assessed. | Section 3.11; Supplementary Table S4 |
| Discussion | 23a | Provide a general interpretation of the results in the context of other evidence. | Section 4 |
|  | 23b | Discuss any limitations of the evidence included in the review. | Section 4.1 |
|  | 23c | Discuss any limitations of the review processes used. | Section 4.1 (fourteen limitations) |
|  | 23d | Discuss implications of the results for practice, policy and future research. | Sections 4.2, 4.5 to 4.7; Section 5 |
| Registration and protocol | 24a | Provide registration information for the review, including register name and registration number, or state that the review was not registered. | PROSPERO, CRD420261303351, registered 9 February 2026; the record was amended during this revision and the current version is Version 3.1, published on 15 September 2026. The record was created through the register’s training route. PROSPERO designates training records as not for publication, so the record does not enter the register’s published series, is not returned by its search interface, and carries a TRAINING watermark. It can be viewed directly at https://www.crd.york.ac.uk/PROSPERO/view/CRD420261303351, where the version history and the text of each version are visible, and it is reproduced in full as Supplementary File 1. Section 2.1. |
|  | 24b | Indicate where the review protocol can be accessed, or state that a protocol was not prepared. | A separate protocol document was not prepared; the registration record serves as the protocol and is reproduced as Supplementary File 1. The search strategy filed with the registration is available at https://www.crd.york.ac.uk/PROSPEROFILES/b95dfd58d4898ba1fd91303d3caf348e.pdf. Section 2.1. |
|  | 24c | Describe and explain any amendments to information provided at registration or in the protocol. | **(1) Eligibility.** In response to first-round peer review, the eligibility criterion was changed from a terminology-based definition, which required the source article to identify the medicine explicitly as NVBP-selected, to a substance-based definition keyed to the active ingredient, dosage form and procurement round (Section 2.2), to avoid terminology-based selection bias. |
|  |  |  | **(2) Search strategy and information sources.** The strategy filed with the registration combines, for each database, a policy concept with the generic-drug concept or the quality-and-outcomes concept. The search reported in the original submission was described in two arms but, as executed, retrieved 110 records in total across four sources. In this revision the search was rebuilt across six sources to implement the registered structure, the second arm being anchored on generic-versus-originator terminology without procurement terms so that eligible studies not using procurement wording could be retrieved. Wanfang Data and VIP, which were not listed at registration, were added. The complete strategy for every source and arm, with the date on which each was searched, is reported in Supplementary Table S1. |
|  |  |  | **(3) Embase and Cochrane CENTRAL.** Embase was listed among the registered information sources but could not be searched because institutional access was unavailable. Cochrane CENTRAL was searched in both arms, but its bibliographic records could not be exported and did not enter deduplication; hit counts are reported in Supplementary Table S1. Both are acknowledged as limitations in Section 4.1. |
|  |  |  | **(4) Scope.** The review was refined to real-world clinical outcomes. In vitro pharmaceutical quality data, which the registration listed among the outcomes of interest, were not synthesised; results of national regulatory sampling are summarised as contextual background in Section 4.2. |
|  |  |  | **(5) Comparator.** One study comparing two procured generic formulations of olanzapine was presented in a sensitivity analysis in the previous version. The registered record excludes comparisons between two NVBP-procured generics without reference to an originator or a pre-policy baseline; the study is retained but reported in the within-patient stratum in Section 3.7 and does not enter the originator-comparator synthesis. |
|  |  |  | **(6) Certainty, reporting bias and synthesis.** The registration stated that certainty would not be assessed, that risk of bias due to missing results would not be assessed, and that no formal synthesis was planned. In response to peer review, GRADE was applied to each outcome domain (Supplementary Table S4), publication and reporting bias was assessed qualitatively within GRADE and discussed in Sections 3.11 and 4.1, and results were synthesised without meta-analysis following the SWiM guideline, using structured effect-direction tabulation (Section 2.7). |
|  |  |  | **(7) Risk-of-bias instruments.** The registration listed Cochrane RoB 2, the Newcastle–Ottawa Scale and the JBI checklist for cross-sectional studies. Where the registered tools did not apply, a modified ROBINS-I was used for before-and-after studies and the IHE checklist for pharmacovigilance case series. Assessment was extended to eight domains, three of which were developed for this policy setting because no existing instrument captures them (Section 2.6). |
|  |  |  | **(8) Selection process.** The registration specified that records would be screened by one reviewer and checked by at least one other. Titles and abstracts were screened in that way; the original submission described that stage as independent duplicate screening, which did not match the registered process and has been corrected. All reports reaching full-text assessment were assessed independently and in duplicate against a fixed sequence of eligibility questions, with disagreements resolved by discussion, and a random sample of excluded records was independently re-assessed (Section 2.4). Data extraction followed the registered process, with one reviewer extracting and a second checking (Section 2.5). |
|  |  |  | **(9) Other.** The registration restricted inclusion to English- and Chinese-language publications and placed no end date on the search; no language restriction was applied, the publication window was closed at 31 January 2026, and the searches were re-executed during this revision (Supplementary Table S1). The registration excluded non-peer-reviewed publications; seven master’s theses indexed in CNKI that met the other eligibility criteria were included and are identified in Table 1 (Section 4.1). |
|  |  |  | **(10) Sequence of eligibility questions and reasons for exclusion.** Eligibility was applied through a fixed eight-question sequence, applied in a set order, so that each excluded report carries the reason encountered first. An eighth reason, publication date outside the search window, was added to the seven implied by the registered criteria. |
|  |  |  | **(11) Timing of the study period.** A report was excluded on timing grounds only when its recruitment window closed before the announcement of the relevant round, or when the overlap with the post-announcement period was shorter than one month. The registration did not specify a minimum overlap. |
|  |  |  | **(12) Dosage-form equivalence.** Dosage-form equivalence was judged against the procurement unit used in the national list rather than against the pharmacopoeial dosage form. The registration did not specify how dosage forms would be matched. |
|  |  |  | **(13) Multi-arm studies.** Where a study compared more than two arms or more than one medicine, only the eligible arms or medicines were extracted and each eligible pair was entered as a separate comparison. The registration did not address multi-arm designs. |
|  |  |  | **(14) Comparator strata.** The three comparator types listed at registration were analysed as separate strata, with the originator-comparator stratum constituting the main analysis and the other strata reported separately rather than pooled. The registration did not specify stratification by comparator type. |
|  |  |  | **(15) Post hoc statistical testing.** Where a source reported group sizes and outcome percentages for an eligible comparison but did not test that comparison, frequencies were reconstructed and chi-square tests applied by the review authors. Such analyses are identified as post hoc and are excluded in a sensitivity analysis. No such analysis was planned at registration. |
|  |  |  | **(16) Direction of effect where no denominator is reported.** An outcome reported without an identifiable denominator does not determine direction. One included study reports adverse drug reaction rates in aggregate across twenty medicines without identifying the medicines concerned or any denominator; no direction is assigned to it. |
| Support | 25 | Describe sources of financial or non-financial support and the role of the funders. | Funding statement |
| Competing interests | 26 | Declare any competing interests of review authors. | Conflict of Interest statement |
| Availability of data, code and other materials | 27 | Report which of the following are publicly available and where. | Data Availability Statement; Supplementary Tables S1 to S7. The re-screening records underlying Section 2.4 are available on request. |

Checklist items are from Page MJ et al. The PRISMA 2020 statement: an updated guideline for reporting systematic reviews. BMJ 2021;372:n71.
