# Supplementary material for: Clinical efficacy and safety of medicines procured through China’s national volume-based procurement policy: a systematic review of 81 studies
Source: Front Public Health. 2026 Oct 15;14:1820505. doi: 10.3389/fpubh.2026.1820505 (PMC13716852; doi:10.3389/fpubh.2026.1820505)
Supplement: Supplementary file 7 [file Table_6.docx]

**Supplementary Table S6. Full-text eligibility decision for all 862 reports assessed, with the reason for each exclusion.**

All 862 reports that reached full-text assessment were screened in duplicate against a fixed eight-question sequence applied in a set order, so that each excluded report carries the reason encountered first. Disagreements arose for 18 reports and were resolved by discussion. For the excluded records, 400 of the 781 were drawn at random and independently re-assessed against the full text by two reviewers who were blind to the recorded decision; their judgements agreed with the recorded decision in 393 cases, and the seven discrepant reports were discussed and the recorded decision was upheld in every case.

Records are ordered by decision and then by record number. Record numbers are those of the screening workbook and are used throughout Supplementary Tables S2, S3 and S7 and in Table 1.

| **No.** | **Authors** | **Title** | **Source** | **DOI** | **Decision and reason** |
| --- | --- | --- | --- | --- | --- |
| 26 | 杨惠卿; 许志连; 张定芳; 洪顺福 | 一项真实世界的集采与原研阿托伐他汀钙片对急性缺血性脑血管病的疗效与安全性研究 | 实用药物与临床 | 10.14053/j.cnki.ppcr.202510003 | Included |
| 38 | 果伟; 贾菲; 刘珊珊; 庄红艳 | 仿制与原研舍曲林片疗效和安全性比较的真实世界研究 | 药物不良反应杂志 | 10.3760/cma.j.cn114015-20221031-01011 | Included |
| 72 | 孙静雯; 蔡鸿福; 林碧娟; 刘茂柏; 曾晓芳 | 国家集中带量采购药品头孢呋辛钠疗效与安全性的真实世界研究 | 中国现代应用药学 | 10.13748/j.cnki.issn1007-7693.20243281 | Included |
| 73 | 罗晓云; 廖万威; 罗丹玲 | 国家集中采购药品在精神疾病治疗中的效用与经济性评价 | 中国处方药 | 10.3969/j.issn.1671-945X.2022.02.026 | Included |
| 75 | 蔡鸿福; 陈如月; 林靖雯; 曾晓芳; 王之舟; 董宪喆 | 国家集采左氧氟沙星氯化钠注射液仿制药疗效和安全性评价 | 中国医疗保险 | 10.19546/j.issn.1674-3830.2024.7.001 | Included |
| 78 | 薛建文; 卓力天; 黄兴振 | 国家集采苯磺酸氨氯地平片治疗高血压的临床效果与安全性远程药学随访研究 | 中国医药科学 | 10.20116/j.issn2095-0616.2024.20.26 | Included |
| 82 | 李春芝; 刘东; 彭善鑫; 王玉玺; 曹美琪; 刘文启 | 基于DRG的集采伏立康唑片治疗真菌感染的临床研究 | 实用药物与临床 | 10.14053/j.cnki.ppcr.202601003 | Included |
| 83 | 李玲; 程涛; 管滢芸; 单媛媛; 姚鸿萍; 李晓曦 | 基于倾向性评分匹配法评价沙格列汀仿制药与原研药的有效性与安全性 | 中国临床药学杂志 | 10.19577/j.1007-4406.2025.11.002 | Included |
| 84 | 杨田江; 赵凯; 魏泽洋; 刘振国 | 基于儿科临床真实世界对注射用头孢曲松钠集中带量采购前后的综合评价 | 儿科药学杂志 | 10.13407/j.cnki.jpp.1672-108X.2025.09.009 | Included |
| 89 | 宋沧桑; 邓雨琴; 刘璐; 毛盼盼; 张函舒; 董艳珍 | 基于多中心真实世界数据的集采注射用头孢曲松治疗细菌性感染的临床综合评价 | 中国医院药学杂志 | 10.13286/j.1001-5213.2024.05.15 | Included |
| 96 | 李红桥; 夏雯; 李兰; 柯敏; 周倩; 饶志威 | 基于真实世界数据的集采注射用醋酸卡泊芬净治疗真菌感染的有效性、安全性和经济性评价 | 中南药学 | 10.7539/j.issn.1672-2981.2025.04.042 | Included |
| 97 | 高顺华; 朱琳; 张春霞 | 基于真实世界的集采与非集采注射用美罗培南治疗细菌性感染有效性、安全性和经济性评价 | 实用药物与临床 | 10.14053/j.cnki.ppcr.202601004 | Included |
| X1 | 彭倩雯; 徐斌; 张伟霞 | 急性冠脉综合征患者原研与“带量采购”阿托伐他汀钙片疗效和安全性的回顾性比较 | 药物流行病学杂志 2023;32(4):361-366 | 10.19960/j.issn.1005-0698.202304002 | Included |
| X2 | 袁倩倩; 马月静; 刘莹; 张相林; 覃旺军 | 基于真实世界的集中带量采购品种注射用头孢呋辛钠预防剖宫产术后感染的临床再评价 | 中国医院用药评价与分析 2024;24(3):275-277 | 10.14009/j.issn.1672-2124.2024.03.005 | Included |
| X3 | 王琲 等 | 利奈唑胺片仿制药与原研药有效性和安全性比较的真实世界研究 | 中国临床药学杂志 2023;32(1):18-22 | 10.19577/j.1007-4406.2023.01.004 | Included |
| X4 | 吴义来; 何银梅; 余建国; 栾家杰 | 基于真实世界数据的阿托伐他汀钙片集采品种短期降脂效果、安全性及经济性评价 | 齐齐哈尔医学院学报 2024;45(18):1707-1712 | 10.3969/j.issn.1002-1256.2024.18.002 | Included |
| X5 | 李一辰; 伊曼曼; 邵腾飞 等 | 集中带量采购替格瑞洛仿制药与原研药用于行支架辅助弹簧圈栓塞术颅内动脉瘤患者的有效性及安全性比较 | 中国临床药学杂志 2024;33(9):661-666 | 10.19577/j.1007-4406.2024.09.004 | Included |
| X6 | Si X; Huang L; Ding Q; et al | Comparison of clinical and economic evaluation between selected generic and original febuxostat tablets in Chinese gout patients with hyperuricemia | Medicine 2024;103(4):e37081 | 10.1097/MD.0000000000037081 | Included |
| X7 | Xing X; Wang Z; Wang K; et al | Comparison of treatment patterns, efficacy and safety between generic and branded atorvastatin users in China: a multicenter, retrospective propensity | Lipids in Health and Disease 2025;24:248 | 10.1186/s12944-025-02673-9 | Included |
| X8 | 罗巧; 王之舟; 刘文娜; 崔晓辉; 董宪喆; 张兰 | 格列美脲集采中选仿制药与原研药临床效果的真实世界研究 | 中国药业 2023;32(24):50-54 | 10.3969/j.issn.1006-4931.2023.24.012 | Included |
| X9 | 李玲; 管滢芸; 于平; 齐艳霞; 马培志; 张文; 栾家杰; 卞晓岚 | 基于倾向性评分匹配法评价格列美脲仿制药和原研药的疗效与安全性 | 中国临床药学杂志 2023;32(1):8-12 | 10.19577/j.1007-4406.2023.01.002 | Included |
| 102 | 李巍; 林明琼 | 奥氮平片中标仿制药与原研药治疗效果与安全性的比较研究 | 中国现代医药杂志 | 10.3969/j.issn.1672-9463.2025.01.012 | Included |
| 103 | 杨燕妮; 黎曙霞; 张晓娟; 金伟军; 陈明浩 | 奥沙利铂集采药品安全性的真实世界研究 | 今日药学 | 10.12048/j.issn.1674-229X.2024.06.010 | Included |
| 104 | 钱巧; 张宁; 朱恒波; 方丹君 | 奥美拉唑原研组和带量采购组在幽门螺杆菌感染根治中疗效、安全性及经济性分析 | 中南药学 | 10.7539/j.issn.1672-2981.2024.11.042 | Included |
| 107 | 贾菲; 臧彦楠; 牛梦溪; 刘珊珊; 庄红艳; 果伟 | 帕罗西汀片药品集中采购中标药疗效与安全性的真实世界研究 | 药物流行病学杂志 | 10.19960/j.issn.1005-0698.202301003 | Included |
| 112 | 赵琴琴; 周小娟; 赵艳花; 冯秀亚; 郑波; 朱珺 | 带量采购背景下的泮托拉唑预防脑梗死患者应激性溃疡的队列研究 | 中国现代应用药学 | 10.13748/j.cnki.issn1007-7693.20222283 | Included |
| 115 | 张丽琼; 田源; 耿兴星 | 带量集中采购前后氨氯地平和厄贝沙坦治疗原发性高血压的经济性对比 | 中国药物经济学 | 10.12010/j.issn.1673-5846.2023.05.007 | Included |
| 119 | 王莹; 于欢; 闫婷婷; 潘文斐 | 恩格列净在2型糖尿病合并心衰患者中的疗效及安全性研究（英文） | Journal of Chinese Pharmaceutical Sciences |  | Included |
| 142 | 王可; 董宪喆; 王之舟; 冯英楠; 庄伟; 栾家杰 | 替吉奥仿制药与原研药疗效与安全性的真实世界研究 | 实用药物与临床 | 10.14053/j.cnki.ppcr.202304011 | Included |
| 143 | 缪阳; 孟中如; 祁旺; 张丹; 许康世; 王红霞 | 替格瑞洛集采中标药与原研药用于ACS患者PCI后的疗效与安全性比较 | 中国药房 | 10.6039/j.issn.1001-0408.2024.20.14 | Included |
| 144 | 陈永; 邓子祺; 顾航烨; 陈吉生 | 某三甲医院国家集采相关药品使用特点及安全性分析 | 今日药学 | 10.12048/j.issn.1674-229X.2023.07.013 | Included |
| 145 | 洪亮 | 某地区集中带量采购药品疗效与安全性真实世界评价分析 |  | 10.27877/d.cnki.gmdjm.2025.000007 | Included |
| 154 | 王可; 王之舟; 邢晓璇; 花一鸣; 董宪喆; 张兰 | 盐酸普拉克索片集采中选仿制药与原研药疗效与安全性的真实世界比较研究 | 中国医院药学杂志 | 10.13286/j.1001-5213.2025.05.07 | Included |
| 164 | 李红桥; 夏雯; 李兰; 柯敏; 饶志威 | 美罗培南仿制药与原研药治疗重症肺炎的有效性、安全性与经济性比较 | 中国临床药学杂志 | 10.19577/j.1007-4406.2025.07.002 | Included |
| 182 | 叶伍飞; 刘俊; 赖伟华; 马培志; 封卫毅; 栾家杰 | 阿哌沙班集中带量采购中选仿制药与原研药的临床疗效和安全性研究 | 中国医院用药评价与分析 | 10.14009/j.issn.1672-2124.2024.08.006 | Included |
| 183 | 王可; 董宪喆; 冯英楠; 王之舟; 庄伟; 栾家杰 | 阿扎胞苷集采中选仿制药与原研药的疗效及安全性比较 | 临床药物治疗杂志 | 10.3969/j.issn.1672-3384.2023.03.006 | Included |
| 185 | 杨依磊; 袁玉霞; 任洪耀; 衣巧艳; 李妍; 李羚 | 集中带量采购中标仿制药恩替卡韦与原研药治疗慢性乙型肝炎的有效性、安全性与经济性比较 | 中国临床药学杂志 | 10.19577/j.1007-4406.2023.11.001 | Included |
| 186 | 王之舟; 董宪喆; 王可; 冯英楠; 罗巧; 岳小林 | 集中带量采购中选与原研头孢地尼口服常释制剂疗效和安全性比较 | 药物不良反应杂志 | 10.3760/cma.j.cn114015-20221101-01015 | Included |
| 194 | 肖卉; 周筠 | 集中采购药品苯磺酸氨氯地平片的有效性、安全性、经济性研究 | 内江科技 |  | Included |
| 197 | 张晓东 | 集采与原研氯吡格雷对冠心病患者PCI术后的疗效及成本对比研究 |  | 10.27202/d.cnki.gkmyc.2022.000065 | Included |
| 198 | 陈维铖 | 集采与进口苯磺酸氨氯地平治疗原发性高血压效果对比研究 |  | 10.27202/d.cnki.gkmyc.2021.000711 | Included |
| 200 | 王睿 | 集采仿制利伐沙班在NVAF患者中的安全性、有效性研究及DOACs的综合研究 |  | 10.27202/d.cnki.gkmyc.2024.001682 | Included |
| 207 | 张歆; 周婧; 译丽则热·司马义; 沈静; 张裴 | 集采背景下恩格列净完整临床综合评价实践与思考 | 中国处方药 | 10.3969/j.issn.1671-945X.2025.02.001 | Included |
| 208 | 张雪莹 | 集采苯磺酸氨氯地平和厄贝沙坦的临床综合评价 |  | 10.27202/d.cnki.gkmyc.2022.000571 | Included |
| 211 | 张旭东; 苏丹; 方苹苹; 苏广全; 桂双英; 沈爱宗 | 集采非集采和进口莫西沙星注射液治疗肺部感染的药物经济学评价 | 中国药物经济学 |  | Included |
| 215 | 庞婕; 吴晓雯; 李春华; 金泰宇; 游维丽 | 中标与原研盐酸莫西沙星片治疗门诊社区获得性肺炎的有效性、安全性与经济性比较 | 中国药房 | 10.6039/j.issn.1001-0408.2023.02.14 | Included |
| 218 | 冯英楠; 董宪喆; 彭静; 王可; 王之舟; 栾家杰 | 仿制与原研左乙拉西坦注射用浓溶液疗效、安全性与经济性比较的真实世界研究 | 药物流行病学杂志 | 10.12173/j.issn.1005-0698.202312037 | Included |
| 219 | 杨世磊; 李璐; 朱艳娜; 翟晓涵; 吕林林; 陈艳伟 | 仿制与原研替格瑞洛抗血小板治疗有效性和安全性的比较研究 | 药物不良反应杂志 | 10.3760/cma.j.cn114015-20221107-01034 | Included |
| 220 | 文志勇; 郭嘉豪; 杨雪明; 李雪芹; 贺瑜; 季波 | 仿制与原研维格列汀片治疗2型糖尿病有效性和安全性比较的真实世界研究 | 药物不良反应杂志 | 10.3760/cma.j.cn114015-20221109-01041 | Included |
| 223 | 王雷; 何孝银; 陈开通; 何涛 | 国产与原研阿托伐他汀治疗高脂血症有效性和安全性的比较研究 | 实用药物与临床 | 10.14053/j.cnki.ppcr.202508006 | Included |
| 224 | 杨伟杰; 徐杏华; 赖荣芬 | 国产与进口盐酸莫西沙星氯化钠注射液治疗肺部感染的疗效和安全性 | 中国药物经济学 |  | Included |
| 228 | 何梅; 曾月涵; 侯雅琴; 黎风; 杨明 | 国产和原研利奈唑胺葡萄糖注射液治疗葡萄球菌和肠球菌轻中度血流感染疗效及安全性比较 | 中国药业 | 10.3969/j.issn.1006-4931.2023.08.023 | Included |
| 236 | 梁美芳; 陈庆状; 杨沛群; 王勇 | 基于真实世界的阿托伐他汀仿制药和原研药防治缺血性脑卒中/短暂性脑缺血发作的有效性和安全性比较 | 中国临床药理学与治疗学 | 10.12092/j.issn.1009-2501.2022.07.009 | Included |
| 240 | 宋婷婷; 黄绍平; 王雪莹; 余静洁; 李丹 | 左乙拉西坦仿制药与原研药临床疗效及安全性的真实世界研究 | 儿科药学杂志 | 10.13407/j.cnki.jpp.1672-108X.2023.08.002 | Included |
| 243 | 颜颖慧; 王凤娇; 王文娟; 朱增燕 | 左乙拉西坦片仿制药与原研药治疗儿童癫痫的疗效、安全性比较 | 中国医院用药评价与分析 | 10.14009/j.issn.1672-2124.2024.01.014 | Included |
| 244 | 梁明娟; 邱炜凤; 张静雯; 黎雪萍; 石港安; 翟琼香 | 左乙拉西坦的国产仿制药替换治疗儿童癫痫的疗效及安全性研究 | 中国当代儿科杂志 | 10.7499/j.issn.1008-8830.2111033 | Included |
| 248 | 张永强 | 硫酸氢氯吡格雷片仿制药与原研药治疗冠心病的效果比较 | 临床合理用药杂志 | 10.15887/j.cnki.13-1389/r.2020.31.016 | Included |
| 251 | 蒋凌; 张丽亚 | 艾司奥美拉唑钠原研药及仿制药治疗上消化道出血的效果 | 中国药物经济学 | 10.12010/j.issn.1673-5846.2022.12.026 | Included |
| 257 | 谢欠; 李娜; 龚敏; 易吉秀; 陈正萍 | 阿托伐他汀钙片仿制药与原研药治疗高脂血症的疗效与经济学评价 | 中国医院用药评价与分析 | 10.14009/j.issn.1672-2124.2021.01.009 | Included |
| 264 | Liu LL; Feng Y; Ren CY; Liu Y; Zhu MH; Hao GX | A Real-World Pharmacological Assessment of Tenofovir Amibufenamide and Tenofovir Alafenamide in 48-Week Chronic Hepatitis B Treatment: Efficacy, Safet | Current medical science | 10.1007/s11596-025-00120-5 | Included |
| 279 | Zhang C; Ding Y; Wu Z; Wang J; Wu X; Xie W | Does China's competitive generic substitution policy deliver equivalent clinical outcomes? A pilot study with two generic formulations of olanzepine. | Frontiers in pharmacology | 10.3389/fphar.2023.1097600 | Included |
| 281 | Zhao B; Wu J; Lu C; Feng XL | Generic substitution of amlodipine is not associated with increased risk of mortality or adverse cardiovascular events: An observational cohort study. | Clinical and translational science | 10.1111/cts.13779 | Included |
| 423 | Wang Z; Wang K; Hua Y; Hu X; Zhang X; Li X | Comparative analysis of thrombocytopenia incidence in patients treated with generic vs. brand-name linezolid: a cohort study utilizing hospital electr | Frontiers in pharmacology | 10.3389/fphar.2025.1528633 | Included |
| 603 | 伊佳; 钱石静; 赵新才; 李慧; 苏颖杰; 王可 | 硼替佐米集采中选仿制药与原研药临床效果对比的真实世界研究 | 中国医疗保险 | 10.19546/j.issn.1674-3830.2024.8.002 | Included |
| 606 | 黄炎清; 张红宇; 李莎莎; 李淼晴; 贾乔迪; 梁红霞 | 评估慢性乙型病毒性肝炎患者换用集采恩替卡韦的疗效与安全性 | 胃肠病学和肝病学杂志 | 10.3969/j.issn.1006-5709.2022.04.016 | Included |
| 607 | 张艳; 张寅英; 李晔; 杨蕊 | 集采与进口头孢呋辛钠对妇产科围术期预防用药的疗效和安全性评价 | 浙江临床医学 |  | Included |
| 611 | 易文燕; 罗青锚; 蒋周倩; 欧荣清; 李乐; 严汝庆 | 阿托伐他汀仿制药与原研药用于2型糖尿病合并血脂异常患者的临床疗效比较 | 当代医学 |  | Included |
| 618 | 刘璐; 宋沧桑; 李兴德; 邓雨琴; 毛盼盼; 张函舒 | 3种集采与非集采注射用头孢菌素有效性和安全性的多中心研究 | 中国药房 | 10.6039/j.issn.1001-0408.2024.07.10 | Included |
| 718 | 梁殷 | 不同核苷类似物治疗慢性乙型肝炎病毒感染者的疗效与安全性研究 |  |  | Included |
| 720 | 王友芳; 李兴超; 朱晓松; 周良健; 杨淑红; 相然 | 不同类型氯吡格雷治疗老年急性冠脉综合征经皮冠状动脉介入术患者对比观察 | 社区医学杂志 | 10.19790/j.cnki.JCM.2023.08.05 | Included |
| 730 | 韩嘉伦; 张翼; 林佰弟; 石秀锦; 赵强; 王依繁 | 中标对比原研氯吡格雷用于急性冠脉综合征患者经皮冠状动脉介入术后治疗的临床有效性及安全性研究 | 中国医院药学杂志 | 10.13286/j.1001-5213.2021.06.08 | Included |
| 739 | 罗巧; 董宪喆; 王之舟; 刘文娜; 崔晓辉; 张兰 | 二甲双胍带量采购中选仿制药疗效与安全性评价的真实世界研究 | 实用药物与临床 | 10.14053/j.cnki.ppcr.202305008 | Included |
| 749 | 朱虹 | 仿制与原研利伐沙班用于非瓣膜性心房颤动抗凝治疗的有效性安全性和经济性评价 |  |  | Included |
| 751 | 陈喆; 石亚飞; 杨珺; 刘敏; 戚姝娅; 朱志翔 | 仿制与原研卡培他滨的真实世界研究 | 中国医院用药评价与分析 | 10.14009/j.issn.1672-2124.2023.05.005 | Included |
| 752 | 杨珺; 石亚飞; 戚姝娅; 陈伟; 李国辉 | 仿制与原研培美曲塞治疗非小细胞肺癌疗效与安全性的比较研究 | 药物不良反应杂志 | 10.3760/cma.j.cn114015-20210209-00170 | Included |
| 753 | 李雨萱; 尹东锋; 李倩 | 仿制与原研注射用头孢曲松钠治疗社区获得性肺炎患儿药物经济学比较 | 中国药业 | 10.3969/j.issn.1006-4931.2024.02.028 | Included |
| 760 | 许金栋; 杨颖; 单玲玲 | 仿制药缬沙坦氨氯地平治疗高血压的临床效果 | 临床合理用药 | 10.15887/j.cnki.13-1389/r.2024.20.009 | Included |
| 823 | 程模; 彭杨; 胡容; 张露; 皮桃英 | 原研和国产吸入用布地奈德混悬剂治疗支气管哮喘临床疗效一致性研究 | 中药与临床 | 10.3969/j.issn.1674-926X.2024.02.014 | Included |
| 840 | 葛丽烨; 张静寅; 马维娜; 孟拥军; 蔡曼曼 | 国家组织药品集中带量采购政策下某院替加环素临床使用分析 | 中国药业 | 10.3969/j.issn.1006-4931.2024.10.006 | Included |
| 853 | 刘琰; 于静; 刘欢; 张彦景; 赵明沂 | 国家集中带量采购盐酸莫西沙星氯化钠注射液治疗社区获得性肺炎应用效果的真实世界研究 | 中国医药导报 | 10.20047/j.issn1673-7210.2023.26.29 | Included |
| 1415 | Cheng, YY; Ye, Q; He, MJ; Ma, WJ; Luo, PP | Randomized Comparison of Vonoprazan-Amoxicillin Dual Therapy Versus Bismuth Quadruple Therapies for Helicobacter pylori Eradication: A Multicenter Tri | JOURNAL OF GASTROENTEROLOGY AND HEPATOLOGY | 10.1111/jgh.70179 | Included |
| 4 | 梁澜 | A机场专业化公司固定资产内部控制案例研究 |  | 10.27015/d.cnki.gdwju.2019.000363 | E1 Medicine or dosage form not in a completed round |
| 6 | 崔满 | J人民银行固定资产内部控制研究 |  | 10.27122/d.cnki.ghlnu.2021.000215 | E1 Medicine or dosage form not in a completed round |
| 11 | 张琬鑫 | W公交集团采购业务内部控制研究 |  | 10.27786/d.cnki.gzjlg.2022.000785 | E1 Medicine or dosage form not in a completed round |
| 18 | 徐桃秀 | “药品集采”政策下我院近年降糖药的临床应用情况分析 | 北方药学 | 10.3969/j.issn.1672-8351.2025.01.038 | E1 Medicine or dosage form not in a completed round |
| 31 | 牛楠楠 | 中车集团集中采购的供应商评价研究 |  | 10.26944/d.cnki.gbfju.2019.001702 | E1 Medicine or dosage form not in a completed round |
| 37 | 彭韵佳; 黄凯莹; 林苗苗 | 从心脏支架“打折到脚踝”看集采降价不降质 | 新华每日电讯 | 10.28870/n.cnki.nxhmr.2024.001929 | E1 Medicine or dosage form not in a completed round |
| 48 | 薛允莲; 梁会营; 李丹; 刘贵浩; 张冠荣; 杨小红 | 冠脉支架集中带量采购对患者住院负担及医疗安全性的影响研究 | 中国卫生质量管理 | 10.13912/j.cnki.chqm.2025.32.12.06 | E1 Medicine or dosage form not in a completed round |
| 81 | 于文萍 | 基于COSO的集中采购内部控制管理 | 中国金融 |  | E1 Medicine or dosage form not in a completed round |
| 91 | 赵楷; 闫俊桥; 胡煜环; 毕雪华; 张琳琳 | 基于多链协同的医疗集采数据监管方案 | 计算机工程 | 10.19678/j.issn.1000-3428.0069156 | E1 Medicine or dosage form not in a completed round |
| 98 | 刘伟; 耿姣姣; 杨文勇; 邓刚 | 基于真实世界证据的聚氨酯材质PICC导管带量采购后临床应用评价研究 | 中国医疗设备 | 10.3969/j.issn.1674-1633.20240987 | E1 Medicine or dosage form not in a completed round |
| 99 | 刘士敏; 魏开心; 李晓芳 | 基于真实世界证据的药物涂层球囊集采前后临床使用评价 | 中国医疗设备 | 10.3969/j.issn.1674-1633.20241662 | E1 Medicine or dosage form not in a completed round |
| 100 | 乔媛慧; 白晓冬 | 基于真实世界证据的骨科内植物耗材集中带量采购对手术的影响 | 中国医疗设备 | 10.3969/j.issn.1674-1633.20250518 | E1 Medicine or dosage form not in a completed round |
| 139 | 张黎; 魏园; 岑峰 | 数据安全实践 |  |  | E1 Medicine or dosage form not in a completed round |
| 149 | 刘保龙 | 浅谈物资集中采购管理 | 财经界 | 10.16266/j.cnki.cn11-4098/f.2019.04.015 | E1 Medicine or dosage form not in a completed round |
| 165 | 黄鹤妹; 段文厚; 裴曌 | 耗材集采政策对膝关节置换术患者住院费用的影响——基于某三甲医院真实世界数据研究 | 中国医疗保险 | 10.19546/j.issn.1674-3830.2024.9.013 | E1 Medicine or dosage form not in a completed round |
| 187 | 丘经纬; 庞慧诗; 姚晖 | 集中带量采购前后亚胺培南西司他丁钠的临床应用评价 | 今日药学 | 10.12048/j.issn.1674-229X.2025.07.006 | E1 Medicine or dosage form not in a completed round |
| 192 | 兰艳; 舒成仁; 张松; 鲁憬莉 | 集中带量采购政策背景下某院万古霉素临床应用分析 | 中国药业 | 10.3969/j.issn.1006-4931.2023.09.026 | E1 Medicine or dosage form not in a completed round |
| 216 | 杨博雅; 魏丁玲 | 京新药业:以创新推动发展实现转型升级 | 今日科技 |  | E1 Medicine or dosage form not in a completed round |
| 230 | 何效平; 荣秋玲; 何梅; 刘福 | 国产和进口万古霉素治疗神经外科术后肺部感染MRSA的回顾性配对研究 | 中国药房 |  | E1 Medicine or dosage form not in a completed round |
| 233 | 梁虹艺; 万宁; 叶嘉盛; 季波; 袁进; 陈吉生 | 基于PSM法的真实世界中国产与原研注射用盐酸万古霉素疗效和安全性一致性评价的队列研究 | 中国医院药学杂志 | 10.13286/j.cnki.chinhosppharmacyj.2019.17.08 | E1 Medicine or dosage form not in a completed round |
| 270 | Du X; Luo X; Guo Q; Jiang X; Su Z; Zhou W | Assessment of clinical benefit, cost and uptake of biosimilars versus reference biologics in immune-mediated inflammatory diseases in China. | Frontiers in public health | 10.3389/fpubh.2024.1476213 | E1 Medicine or dosage form not in a completed round |
| 274 | Sun X; Ma C; Zhan M; Zhang Z; Zhan R | Comparative Study of Computer-Aided Designed PEEK Implants Versus Autologous Bone Flap in Cranioplasty: Clinical Efficacy and Medical Economics Analys | The Journal of craniofacial surgery | 10.1097/SCS.0000000000011775 | E1 Medicine or dosage form not in a completed round |
| 275 | Hosseini M | Competition Law and Ethics of Innovation as Catalysts for Fairness: Reimagining the EU's COVID-19 Vaccine Strategy. | The Journal of law, medicine & ethics : a journal of the Ame | 10.1017/jme.2025.10141 | E1 Medicine or dosage form not in a completed round |
| 292 | Yang Z; Ni L; Zhao W; Li Y; Wu Y; Zhu Q | Impact of volume-based procurement policy on coronary stent use in tianjin: interrupted time series analysis. | Journal of health, population, and nutrition | 10.1186/s41043-025-01128-2 | E1 Medicine or dosage form not in a completed round |
| 293 | Wu X; Yao X; Zhao Q; Qi X; Zhang Y; Cong X | Impact of zero-markup consumable policy and national procurement of orthopedic spinal devices in China: a mixed-methods study. | Frontiers in public health | 10.3389/fpubh.2025.1715856 | E1 Medicine or dosage form not in a completed round |
| 315 | Ma X; Chen Y; Jiang S; Zhao X | A Bioassay-Based Approach for the Batch-To-Batch Consistency Evaluation of Xuesaitong Injection on a Zebrafish Thrombosis Model. | Frontiers in pharmacology | 10.3389/fphar.2021.623533 | E1 Medicine or dosage form not in a completed round |
| 316 | Liu X; Dai X; Yu X; Zhou H; Xie J | A Bioequivalence Study of Azilsartan in Healthy Chinese Subjects. | Clinical pharmacology in drug development | 10.1002/cpdd.1479 | E1 Medicine or dosage form not in a completed round |
| 317 | Zhang J; Zhang M; Xue W; Zha Y; Jin S; Luo T | A Bioequivalence Study of Recombinant Human Follicle-Stimulating Hormone Injection Versus Recombinant Human Follitropin For Injection in Healthy Chine | Drugs in R&D | 10.1007/s40268-025-00513-w | E1 Medicine or dosage form not in a completed round |
| 321 | Zhang H; Wang H; Wei H; Chen H; Liu J; Li C | A Phase I Clinical Study Comparing the Tolerance, Immunogenicity, and Pharmacokinetics of Proposed Biosimilar BAT1806 and Reference Tocilizumab in Hea | Frontiers in pharmacology | 10.3389/fphar.2020.609522 | E1 Medicine or dosage form not in a completed round |
| 322 | Hu S; Liu Y; Yang L; Yang Y; Long F; Bao H | A Phase I Clinical Trial to Evaluate the Bioequivalence of an Adalimumab Biosimilar Adalimumab-WIBP and Humira(®). | Clinical drug investigation | 10.1007/s40261-024-01409-4 | E1 Medicine or dosage form not in a completed round |
| 324 | Zhang T; Chen G; Liu C; Zu L; Wang Q; Wang Y | A Phase I Study Comparing the Pharmacokinetics, Safety, and Immunogenicity of Proposed Biosimilar GB242 and Reference Infliximab in Healthy Subjects. | BioDrugs : clinical immunotherapeutics, biopharmaceuticals a | 10.1007/s40259-018-0326-x | E1 Medicine or dosage form not in a completed round |
| 325 | Zhang H; Wu M; Zhu X; Li C; Li X; Sun J | A Phase I, Randomized, Single-Dose Study to Evaluate the Biosimilarity of QL1206 to Denosumab Among Chinese Healthy Subjects. | Frontiers in pharmacology | 10.3389/fphar.2020.01329 | E1 Medicine or dosage form not in a completed round |
| 326 | Xu J; Ge J; Li Y; Liu S; Li S; Si J | A Phase I Study Comparing the Pharmacokinetics, Safety, and Immunogenicity of A140 Injection and Cetuximab (Erbitux(®)) in Healthy Chinese Male Subjec | Advances in therapy | 10.1007/s12325-025-03193-9 | E1 Medicine or dosage form not in a completed round |
| 327 | Zhou R; Chen Q; Hou S; Guo H; Li J; Qian W | A Randomized, Double-Blind, Parallel Controlled, Single-Dose Phase I Study Comparing the Pharmacokinetics, Safety, and Immunogenicity of the Inflixima | Clinical pharmacology in drug development | 10.1002/cpdd.1135 | E1 Medicine or dosage form not in a completed round |
| 328 | Yao F; Wang C; Ding J; Zhang Q; Zheng L; Zhang Q | A Randomized, Double-Blind, Parallel-Group Phase I Study Comparing the Pharmacokinetics, Safety, and Immunogenicity of CMAB015, a Candidate Secukinuma | Drug design, development and therapy | 10.2147/DDDT.S470619 | E1 Medicine or dosage form not in a completed round |
| 329 | Huang K; Que L; Ding Y; Chu N; Qian Z; Shi Y | A Randomized, Double-Blind, Single-Dose Study Comparing the Biosimilarity of HOT-1010 With Bevacizumab (Avastin®) in Chinese Healthy Male Subjects. | Frontiers in pharmacology | 10.3389/fphar.2021.694375 | E1 Medicine or dosage form not in a completed round |
| 330 | Gong F; Hu S; Yuan J; Song J | A Randomized, Open-Label, Single-Dose, Parallel-Group Bioequivalence Study of Ferric Carboxymaltose Injection Under Fasting Conditions in Chinese Adul | Pharmacology research & perspectives | 10.1002/prp2.70137 | E1 Medicine or dosage form not in a completed round |
| 332 | Cheng J; Wang C; Xu J; Zhao C; Song R; Wang Y | A Randomized, Single-Dose, Parallel-Controlled Phase 1 Clinical Comparison of an Omalizumab Biosimilar Candidate with Reference Omalizumab in Healthy | Clinical pharmacology in drug development | 10.1002/cpdd.1350 | E1 Medicine or dosage form not in a completed round |
| 333 | Pu H; Jia J; Zhao C; Hou S; Guo H; Li J | A Randomized, Single-dose, Phase I Clinical Comparison of a Trastuzumab Biosimilar With a Reference Trastuzumab Formulation in Healthy Chinese Male Vo | Clinical pharmacology in drug development | 10.1002/cpdd.1189 | E1 Medicine or dosage form not in a completed round |
| 336 | Feng S; Cai L; Wang X; Yu Q; Cai J; Hao W | A Single-dose, Two-Period Crossover Bioequivalence Study Comparing Two Liraglutide Formulations in Healthy Chinese Subjects. | Clinical pharmacology in drug development | 10.1002/cpdd.1187 | E1 Medicine or dosage form not in a completed round |
| 337 | Hu C; Chen X; Zhao Z; Gao D; Gong S; Zhang L | A Two-Sequence, Four-Period, Crossover, Replicate Study to Demonstrate Bioequivalence of Warfarin Sodium Tablet in Healthy Chinese Subjects Under Fast | Clinical pharmacology in drug development | 10.1002/cpdd.783 | E1 Medicine or dosage form not in a completed round |
| 339 | Han X; Zhang M; Wang H; Zhang Q; Li W; Hao M | A multi-center, open-label, randomized, parallel-controlled phase II study comparing pharmacokinetic, pharmacodynamics and safety of ripertamab (SCT40 | Chinese journal of cancer research = Chung-kuo yen cheng yen | 10.21147/j.issn.1000-9604.2022.06.08 | E1 Medicine or dosage form not in a completed round |
| 342 | Zhu X; Qian H; Sun J; Wu M; Yu C; Ding Y | A phase 1 randomized study compare the pharmacokinetics, safety and immunogenicity of HLX04 to reference bevacizumab sourced from the United States, t | Cancer chemotherapy and pharmacology | 10.1007/s00280-021-04297-z | E1 Medicine or dosage form not in a completed round |
| 343 | Li H; Zhao X; Xie J; Zhu X; Su Y; He C | A phase I study comparing the biosimilarity of the pharmacokinetics and safety of recombinant humanized anti-vascular endothelial growth factor monocl | BMC pharmacology & toxicology | 10.1186/s40360-023-00673-y | E1 Medicine or dosage form not in a completed round |
| 344 | Qian H; Cheng J; Gui Y; Wang W; Liang L; Zhu H | A phase I study comparing the pharmacokinetics and safety of HS628 (tocilizumab biosimilar) and reference tocilizumab in healthy male subjects. | Clinical and translational science | 10.1111/cts.13584 | E1 Medicine or dosage form not in a completed round |
| 345 | Zhou R; Guo L; Gao X; Wang Y; Xu W; Zou Y | A phase I study comparing the pharmacokinetics of the biosimilar (RD12014) with liraglutide (Victoza) in healthy Chinese male subjects. | Clinical and translational science | 10.1111/cts.13374 | E1 Medicine or dosage form not in a completed round |
| 347 | Zhang H; Li X; Liu J; Li C; Wu M; Zhu X | A randomized phase-I pharmacokinetic trial comparing the potential biosimilar tocilizumab (QX003S) with the reference product (Actemra(®)) in Chinese | Annals of medicine | 10.1080/07853890.2021.1887925 | E1 Medicine or dosage form not in a completed round |
| 348 | Yang Q; Jiang Y; Liu Z; Wu J; Dong Q; Liu Y | A randomized, double-blind, single-dose, parallel two-group study comparing the pharmacokinetics, safety, and immunogenicity of BAT1806 SC with tocili | Expert opinion on biological therapy | 10.1080/14712598.2025.2574006 | E1 Medicine or dosage form not in a completed round |
| 349 | Wang Y; Gao Z; Liu Z; Liu G; Qu X; Chen J | A randomized, double-blind, single-dose, two-way, parallel phase I clinical study comparing the pharmacokinetics and safety of adalimumab injecta and | Expert opinion on biological therapy | 10.1080/14712598.2021.1944097 | E1 Medicine or dosage form not in a completed round |
| 350 | Li Y; Wang Y; Qi L; Li Y; Zhong W; Wang X | A randomized, open, two-period, cross-over, single oral dose, pharmacokinetics and bioequivalence study of vitamin K(1) in healthy volunteers under fa | Frontiers in pharmacology | 10.3389/fphar.2026.1802709 | E1 Medicine or dosage form not in a completed round |
| 354 | Wang Y; Liu Z; Deng Q; Su Z; Xue J; Zhao Y | A randomized, single-blind, single-dose, parallel-group study in healthy subjects to demonstrate the pharmacokinetic equivalence of trastuzumab and it | Expert opinion on drug metabolism & toxicology | 10.1080/17425255.2023.2243823 | E1 Medicine or dosage form not in a completed round |
| 358 | Lin S; Lou Y; Hao R; Shao Y; Yu J; Fang L | A single-dose, randomized, open-label, four-period, crossover equivalence trial comparing the clinical similarity of the proposed biosimilar rupatadin | Frontiers in pharmacology | 10.3389/fphar.2024.1328142 | E1 Medicine or dosage form not in a completed round |
| 359 | Hu X; Zhang Q; Zheng Y; Zhai Y; Xu N; Zhao Q | A single-dose, randomized, open-labeled, parallel-group study comparing the pharmacokinetics, pharmacodynamics and safety of leuprolide acetate micros | Frontiers in pharmacology | 10.3389/fphar.2022.946505 | E1 Medicine or dosage form not in a completed round |
| 360 | Liu X; Yang T; Chen L; Lan L; Sun G; Guo P | A strategy takes "Yiqing" tablets as an example to carry out simpler multi-component quantification and use fingerprint technology for comprehensive q | Journal of pharmaceutical and biomedical analysis | 10.1016/j.jpba.2023.115809 | E1 Medicine or dosage form not in a completed round |
| 363 | Lan L; Yang T; Fan J; Sun G; Zhang H | Anti-inflammation activity of Zhizi Jinhua Pills and overall quality consistency evaluation based on integrated HPLC, DSC and electrochemistry fingerp | Journal of ethnopharmacology | 10.1016/j.jep.2023.116442 | E1 Medicine or dosage form not in a completed round |
| 364 | Fu Q; Huang C; Yuan Y; Wang Y; Zhu B; Liu Y | Assessment of Pharmacokinetics and Safety with Bioequivalence of the Nitroglycerin Sublingual Tablets of Two Formulations in Chinese Healthy Subjects: | Drugs in R&D | 10.1007/s40268-025-00519-4 | E1 Medicine or dosage form not in a completed round |
| 368 | Jia C; Zhao N; Song H; Hu Y; Xu Y; Guo C | Bioequivalence Analysis of Ondansetron Hydrochloride Tablets in Healthy Chinese Subjects: A Randomized, Open-Label, Two-Period Crossover Phase I Study | Drugs in R&D | 10.1007/s40268-024-00493-3 | E1 Medicine or dosage form not in a completed round |
| 372 | Yang M; Pei Y; Chen C; Xi J; Xia Y; Zhang S | Bioequivalence Study of Bedaquiline Fumarate Tablets in Healthy Chinese Subjects. | Clinical pharmacology in drug development | 10.1002/cpdd.1515 | E1 Medicine or dosage form not in a completed round |
| 373 | Li Y; Wu Y; Cai S; Huang X; Ye L; Wang L | Bioequivalence Study of Bedaquiline and Sirturo(®) in Healthy Chinese Subjects Under Fasting and Postprandial Conditions: A Randomized, Open-Label, Si | Drugs in R&D | 10.1007/s40268-025-00525-6 | E1 Medicine or dosage form not in a completed round |
| 376 | Hao R; Shao Y; Lin S; Wu Y; Bian L; Zhang Y | Bioequivalence Study of Tebipenem Pivoxil in Healthy Chinese Adults. | Drugs in R&D | 10.1007/s40268-024-00454-w | E1 Medicine or dosage form not in a completed round |
| 382 | Li N; Wang X; Li R; Zhao H; Gao Y; Zhang W | Bioequivalence and Food Effect Assessment of Apalutamide Tablets Relative to Erleada in Healthy Chinese Male Participants. | Clinical pharmacology in drug development | 10.1002/cpdd.1598 | E1 Medicine or dosage form not in a completed round |
| 389 | Hu C; Hu X; Wang C; Zhao Z; Gao D; Chen X | Bioequivalence and Pharmacokinetics of Bisoprolol-Amlodipine 5 mg/5 mg Combination Tablet versus Bisoprolol 5 mg Tablet and Amlodipine 5 mg Tablet: An | Clinical drug investigation | 10.1007/s40261-018-0700-y | E1 Medicine or dosage form not in a completed round |
| 395 | Sun ML; Liu C; Bai HH; Wei YL; Zhang W; Liu HJ | Bioequivalence and safety evaluation of two preparations of metformin hydrochloride sustained-release tablets (Boke(®) and Glucophage(®)-XR) in health | Annals of medicine | 10.1080/07853890.2022.2125574 | E1 Medicine or dosage form not in a completed round |
| 400 | Lei Y; Yan Y; Lu J; Li C; Wang J; Li C | Bioequivalence of Blonanserin Tablets Under Fasting and Fed Conditions in Healthy Chinese Subjects. | Clinical pharmacology in drug development | 10.1002/cpdd.1331 | E1 Medicine or dosage form not in a completed round |
| 403 | Deng Y; Wang G; Jiang G; Song D; Xu X; Zhao D | Bioequivalence of Two 6-Mercaptopurine Tablet Formulations in Healthy Fasting Chinese Volunteers. | Clinical pharmacology in drug development | 10.1002/cpdd.1298 | E1 Medicine or dosage form not in a completed round |
| 410 | Cheng F; Shen T; Zhang F; Lei C; Zhu Y; Luo G | Bioequivalence study of fluticasone propionate nebuliser suspensions in healthy Chinese subjects. | Frontiers in pharmacology | 10.3389/fphar.2024.1452596 | E1 Medicine or dosage form not in a completed round |
| 412 | Sun X; Zhu R; Lu J; Li J; Ding J; Yu Q | Bioequivalence study of two formulations of teriflunomide tablets in a healthy Chinese population under fasting and fed conditions. | International journal of clinical pharmacology and therapeut | 10.5414/CP204734 | E1 Medicine or dosage form not in a completed round |
| 413 | Lin Y; Yang H; Yang X; Guo C; Yang S; Yang G | Biosimilarity of HS-20090 to Denosumab in healthy Chinese subjects: a randomized, double-blinded, pharmacokinetics/pharmacodynamics study. | Expert opinion on investigational drugs | 10.1080/13543784.2022.2123737 | E1 Medicine or dosage form not in a completed round |
| 415 | Wang T; Shang L; He Y; Guo L; Ruan X; Han P | Brexpiprazole Oral Soluble Film: A Randomized, Open-Label, Single-Dose, Crossover Bioequivalence Study in Healthy Chinese Volunteers. | Clinical therapeutics | 10.1016/j.clinthera.2025.07.001 | E1 Medicine or dosage form not in a completed round |
| 420 | Huang Z; Yu P; Hu J; Zhang W | Comparative Pharmacokinetics and Bioequivalence of 2 Formulations of Bosentan Dispersible Tablets in Healthy Chinese Volunteers Under Fasting and Fed | Clinical pharmacology in drug development | 10.1002/cpdd.1516 | E1 Medicine or dosage form not in a completed round |
| 422 | Wang J; Yang T; Mei H; Yu X; Peng H; Wang R | Comparative Pharmacokinetics, Bioequivalence and Safety Study of Two Recombinant Human Chorionic Gonadotropin Injections in Healthy Chinese Subjects. | Drug design, development and therapy | 10.2147/DDDT.S235064 | E1 Medicine or dosage form not in a completed round |
| 425 | Cheng X; Fu Z; Liu J; Cheng B; Shi R; Xu X | Comparative real-world retention rate and safety of biosimilar IBI303 versus Humira in ankylosing spondylitis: A retrospective study. | Medicine | 10.1097/MD.0000000000043010 | E1 Medicine or dosage form not in a completed round |
| 426 | Xu Z; Liu Z; Wang Y; Xue J; Chang T; Cui Y | Comparing the bioequivalence and safety of liraglutide in healthy Chinese subjects: an open, single-dose, randomized, repeated, two-sequence, two-cycl | Expert review of clinical pharmacology | 10.1080/17512433.2023.2188192 | E1 Medicine or dosage form not in a completed round |
| 427 | Wu M; Li X; Yang D; Wang M; Zhang H; Li C | Comparison of Pharmacokinetic Similarity, Immunogenicity, and Safety of Ustekinumab and BAT2206 in Healthy Chinese Male Subjects in a Double-Blind, Ra | BioDrugs : clinical immunotherapeutics, biopharmaceuticals a | 10.1007/s40259-022-00563-5 | E1 Medicine or dosage form not in a completed round |
| 429 | Qin H; Yao C; Zhang W; Hu W; Liu Y; Yu S | Comparison of pharmacokinetics and safety of albuvirtide in healthy subjects after intravenous drip and bolus injection. | Naunyn-Schmiedeberg's archives of pharmacology | 10.1007/s00210-023-02614-2 | E1 Medicine or dosage form not in a completed round |
| 430 | Zhang CY; Li XX; Li P; Jiang Y; Li HJ | Consistency evaluation between dispensing granule and traditional decoction from Coptidis Rhizoma by using an integrated quality-based strategy. | Phytochemical analysis : PCA | 10.1002/pca.2905 | E1 Medicine or dosage form not in a completed round |
| 438 | Zhang Y; Zhou T; Wu N; Bai Z; Wei J; Cai N | Economic evaluation of three domestic bevacizumab biosimilars and the original bevacizumab for treating nonsquamous non-small cell lung cancer in chin | Cost effectiveness and resource allocation : C/E | 10.1186/s12962-025-00653-5 | E1 Medicine or dosage form not in a completed round |
| 442 | Wang J; Yue H; Dong Y; Liu T; Li J | Effective compound combination of Bufei Yishen formula ameliorates PM2.5-induced COPD by inhibiting mitochondrial oxidative stress through SIRT3-media | Phytomedicine : international journal of phytotherapy and ph | 10.1016/j.phymed.2025.156568 | E1 Medicine or dosage form not in a completed round |
| 444 | Shang DW; Wang ZZ; Hu HT; Zhang YF; Ni XJ; Lu HY | Effects of food and grapefruit juice on single-dose pharmacokinetics of blonanserin in healthy Chinese subjects. | European journal of clinical pharmacology | 10.1007/s00228-017-2340-1 | E1 Medicine or dosage form not in a completed round |
| 445 | Yu W; Luo Z; Zhao Y; Hu Z; Zhang A; Li Y | Efficacy and Safety of Combined Topical Lidocaine and Tetracaine Cream for Facial Fractional Laser Resurfacing Compared With Its Reference Product in | Journal of cosmetic dermatology | 10.1111/jocd.70358 | E1 Medicine or dosage form not in a completed round |
| 447 | Han X; Wang C; Zhang Q; Guan B; Zhang H; Yu Y | Efficacy and safety of azelastine hydrochloride and fluticasone propionate nasal spray in treating allergic rhinitis: A randomized controlled trial. | Asia Pacific allergy | 10.5415/apallergy.0000000000000161 | E1 Medicine or dosage form not in a completed round |
| 448 | Shi Y; Bi M; Li Q; Wang G; Chen J; Li M | Efficacy and safety of bevacizumab biosimilar SIBP04 compared with bevacizumab (Avastin®) as first-line treatment for locally advanced or metastatic n | Cancer pathogenesis and therapy | 10.1016/j.cpt.2025.01.001 | E1 Medicine or dosage form not in a completed round |
| 449 | Zhou H; Wang Y; Chen J; He A; Jin J; Lu Q | Efficacy and safety of generic pomalidomide plus low-dose dexamethasone in relapsed or refractory multiple myeloma: a multicenter, open-label, single- | Annals of hematology | 10.1007/s00277-023-05558-y | E1 Medicine or dosage form not in a completed round |
| 451 | Zhang S; Yin Y; Xiong H; Wang J; Liu H; Lu J | Efficacy, Safety, and Population Pharmacokinetics of MW032 Compared With Denosumab for Solid Tumor-Related Bone Metastases: A Randomized, Double-Blind | JAMA oncology | 10.1001/jamaoncol.2023.6520 | E1 Medicine or dosage form not in a completed round |
| 452 | Xi Y; Jin C; Liu W; Zhou H; Wang Z; Zhou R | Efficacy, safety and bioequivalence of the human-derived B-domain-deleted recombinant factor VIII TQG202 for prophylaxis in severe haemophilia A patie | Haemophilia : the official journal of the World Federation o | 10.1111/hae.14652 | E1 Medicine or dosage form not in a completed round |
| 454 | Lee CJ; Kapelemera AM; Tsai YZ; Lee CT; Xu MY; Wang CC | Evaluating the Therapeutic Efficacy of Si-Wu-Tang Decoction and Concentrated Extract in Follicular Maldevelopment-Related Menstrual Disorders Through | Frontiers in pharmacology | 10.3389/fphar.2020.01245 | E1 Medicine or dosage form not in a completed round |
| 455 | Liu C; Xu H; Yuan F; Chen H; Sheng L; Chen W | Evaluating the bioequivalence and safety of liraglutide injection versus Victoza(®) in healthy Chinese subjects: a randomized, open, two-cycle, self-c | Frontiers in pharmacology | 10.3389/fphar.2023.1326865 | E1 Medicine or dosage form not in a completed round |
| 456 | Qiu B; Song H; Sun X; Ding C; Du R; Bai W | Evaluating the pharmacokinetics and safety of blonanserin tablets and Lonasen(®): a randomized, open-label, two-period, two-sequence, self-crossover p | Frontiers in pharmacology | 10.3389/fphar.2024.1511214 | E1 Medicine or dosage form not in a completed round |
| 457 | Yue Z; Wang Y; Li Z; Jin T; Sheng Y | Evaluation of Bioequivalence for Avapritinib Tablets in Chinese Participants Under Fasting Conditions Using a Reference-Scaled Average Bioequivalence | Clinical pharmacology in drug development | 10.1002/cpdd.1398 | E1 Medicine or dosage form not in a completed round |
| 468 | Yang J; Lin L; Long Q; Zhang Q; Sun G; Zhou L | HLX11, a Proposed Pertuzumab Biosimilar: Pharmacokinetics, Immunogenicity, and Safety Profiles Compared to Three Reference Biologic Products (US-, EU- | BioDrugs : clinical immunotherapeutics, biopharmaceuticals a | 10.1007/s40259-022-00534-w | E1 Medicine or dosage form not in a completed round |
| 476 | Cao G; Wang J; He J; Hu Y; Yang H; Que L | LZM008, a proposed tocilizumab biosimilar: Pharmacokinetics, safety, and immunogenicity profiles compared with ACTEMRA(®) in Chinese healthy male subj | Frontiers in pharmacology | 10.3389/fphar.2023.1111893 | E1 Medicine or dosage form not in a completed round |
| 477 | Yan B; Sun G | Monitoring quality consistency of Liuwei Dihuang Pill by integrating the ultraviolet spectroscopic fingerprint, a multi-wavelength fusion fingerprint | Journal of separation science | 10.1002/jssc.201700620 | E1 Medicine or dosage form not in a completed round |
| 479 | Huang J; Ren J; Xie W; Pan R; Xu N; Liu H | Personalised functional imaging-guided multitarget continuous theta burst stimulation for post-stroke aphasia: study protocol for a randomised control | BMJ open | 10.1136/bmjopen-2023-081847 | E1 Medicine or dosage form not in a completed round |
| 482 | Wang F; Luan Z; Maltesen R; Reenberg AT; Westergaard L; Liu | Pharmacokinetic Characteristics of a Once-Weekly Combination Therapy of Insulin Icodec and Semaglutide Versus Its Separate Components in Chinese Indiv | Diabetes therapy : research, treatment and education of diab | 10.1007/s13300-025-01803-x | E1 Medicine or dosage form not in a completed round |
| 484 | Yao F; He Y; Lu P; Wang J; Xie Y; Li X | Pharmacokinetic and Bioequivalence Evaluation of Dihydroxyaluminum Aminoacetate, Heavy Magnesium Carbonate, and Aspirin Tablets in Healthy Chinese Sub | Clinical pharmacology in drug development | 10.1002/cpdd.1455 | E1 Medicine or dosage form not in a completed round |
| 486 | He L; Li W; Lv Y | Pharmacokinetic and Bioequivalence Evaluation of Ulipristal Acetate in Healthy Chinese Subjects in the Fasting and Postprandial Conditions. | Clinical pharmacology in drug development | 10.1002/cpdd.1567 | E1 Medicine or dosage form not in a completed round |
| 487 | Huang X; Huang J; Li X; Chen L | Pharmacokinetic and Bioequivalence Studies of 2 Metformin Glibenclamide Tablets in Healthy Chinese Subjects Under Fasting and Fed Conditions. | Clinical pharmacology in drug development | 10.1002/cpdd.1219 | E1 Medicine or dosage form not in a completed round |
| 488 | Yang Z; Mi X; Li Q; Chen L; Zeng Y; Du P | Pharmacokinetic and Bioequivalence Study of Lisinopril/Hydrochlorothiazide Tablet Under Fasting and Postprandial Conditions in Healthy Chinese Subject | Clinical pharmacology in drug development | 10.1002/cpdd.1329 | E1 Medicine or dosage form not in a completed round |
| 491 | Ding Y; Chu NN; Wang R; Qin W; Shi YF; Qian ZZ | Pharmacokinetic and pharmacodynamic evaluation study of etomidate: a randomized, open-label, 2-period crossover study in healthy Chinese subjects. | Scientific reports | 10.1038/s41598-024-57581-2 | E1 Medicine or dosage form not in a completed round |
| 492 | Dong W; Chen M; Niu S; Wang B; Xia L; Wang J | Pharmacokinetic bioequivalence, safety, and immunogenicity of GB222, a bevacizumab biosimilar candidate, and bevacizumab in Chinese healthy males: a r | Expert opinion on biological therapy | 10.1080/14712598.2021.1954157 | E1 Medicine or dosage form not in a completed round |
| 493 | Zhang Q; Sun C; Wu J; Wu J; Zhang X; Liu Y | Pharmacokinetic similarity study comparing the biosimilar candidate, LY05008, with its reference product dulaglutide in healthy Chinese male subjects. | Expert opinion on biological therapy | 10.1080/14712598.2023.2189009 | E1 Medicine or dosage form not in a completed round |
| 496 | Wang J; Xu Y; Zhao Z; Meng T; Zou Y; Lan Y | Pharmacokinetics and Bioequivalence Evaluation of Trazodone Hydrochloride Sustained-Release Tablets in Healthy Chinese Volunteers. | Clinical pharmacology in drug development | 10.1002/cpdd.1490 | E1 Medicine or dosage form not in a completed round |
| 505 | Pei T; Yang J; Hu C; Chen X; Gong S; Hu X | Pharmacokinetics and Bioequivalence of Clopidogrel Hydrogen Sulfate Tablets in Fed and Fasted Conditions: An Open-Label, Randomized, Semireplicated Cr | Clinical pharmacology in drug development | 10.1002/cpdd.804 | E1 Medicine or dosage form not in a completed round |
| 508 | Zhang P; Li M; Jiang H; Liu F; Huang Q; Han Y | Pharmacokinetics and Bioequivalence of Mycophenolate Sodium Enteric-Coated Tablets Under Fasting and Fed Conditions: A Single-Dose, Open-Label, Four-P | Drug design, development and therapy | 10.2147/DDDT.S529915 | E1 Medicine or dosage form not in a completed round |
| 517 | He Y; Yang L; Mou Q; Cao X; Zhu X; Wang Y | Pharmacokinetics and Safety of Oxycodone/Naloxone Prolonged-Release Tablets in Chinese Patients with Chronic Pain. | Drug design, development and therapy | 10.2147/DDDT.S486714 | E1 Medicine or dosage form not in a completed round |
| 520 | Liu Y; Wang Y; Wang M; Wang Y; Wu P; Xia X | Pharmacokinetics and bioequivalence evaluation of two oral formulations of topiramate tablets in healthy Chinese male volunteers under fasting and fed | Naunyn-Schmiedeberg's archives of pharmacology | 10.1007/s00210-024-03782-5 | E1 Medicine or dosage form not in a completed round |
| 524 | Yan Y; Zhu X; Dong P; Liu C; Lu L; Zeng L | Pharmacokinetics and bioequivalence of two formulations of mifepristone tablets in healthy Chinese subjects under fasting conditions: a single-center, | Frontiers in pharmacology | 10.3389/fphar.2024.1479205 | E1 Medicine or dosage form not in a completed round |
| 529 | Wang Y; Xue J; Su Z; Cui Y; Liu G; Yang W | Pharmacokinetics and safety of dasatinib and its generic: a phase I bioequivalence study in healthy Chinese subjects. | Expert opinion on investigational drugs | 10.1080/13543784.2023.2179481 | E1 Medicine or dosage form not in a completed round |
| 531 | Chen X; Yi X; Liu Y; Wang X; Peng Y; Wang Q | Pharmacokinetics and safety of the antitumor drug everolimus in healthy Chinese subjects: a single-dose, open-label, randomized, two-sequence, two-per | Frontiers in pharmacology | 10.3389/fphar.2025.1718032 | E1 Medicine or dosage form not in a completed round |
| 537 | Wang Y; Zhao X; Wang T; Xiong L; Liu X; Pan J | Pharmacokinetics, Bioequivalence, and Safety Studies of Crisaborole Ointment in Healthy Chinese Subjects. | Clinical pharmacology in drug development | 10.1002/cpdd.1552 | E1 Medicine or dosage form not in a completed round |
| 542 | Jin J; Huang C; Gan F; Li T; Wang L; Wang X | Pharmacokinetics, Bioequivalence, and Safety of Levothyroxine Sodium Tablets in Healthy Chinese Subjects. | Clinical pharmacology in drug development | 10.1002/cpdd.1564 | E1 Medicine or dosage form not in a completed round |
| 543 | Cui Y; Cui D; Ren X; Chen X; Liu G; Liu Z | Pharmacokinetics, Immunogenicity and Safety Study for SHR-1309 Injection and Perjeta® in Healthy Chinese Male Volunteers. | Frontiers in pharmacology | 10.3389/fphar.2021.660541 | E1 Medicine or dosage form not in a completed round |
| 544 | Chen H; Chen W; Yuan F; Guo Q; Zhang X; Wang C | Pharmacokinetics, Pharmacodynamics, Safety and Immunogenicity of CMAB807, a New Denosumab Biosimilar, in Healthy Chinese Subjects. | Frontiers in pharmacology | 10.3389/fphar.2022.821944 | E1 Medicine or dosage form not in a completed round |
| 545 | Cong D; Song J; Liu Y; Tan Y; Xue W; Liu X | Pharmacokinetics, Pharmacodynamics, and Tolerability of Opicapone in Healthy Chinese and Caucasian Subjects: An Open-Label, Single-Center, Phase 1 Stu | Neurology and therapy | 10.1007/s40120-021-00314-8 | E1 Medicine or dosage form not in a completed round |
| 546 | Gao X; Shen K; Tang D; Bai W; Wang J; Wang T | Pharmacokinetics, Relative Bioavailability, and Safety of the SHR0302 Oral Solution and Tablets: A Single-Center, Randomized, Open-Label, Crossover (T | Drug design, development and therapy | 10.2147/DDDT.S513537 | E1 Medicine or dosage form not in a completed round |
| 547 | Zhang W; Lin L; Fu W; Bao D; Zhang Q; Liu Y | Pharmacokinetics, Safety, and Bioequivalence of 2 Lopinavir/Ritonavir (200/50 mg) Tablets in Healthy Chinese Volunteers: Effect of Food on Absorption. | Clinical pharmacology in drug development | 10.1002/cpdd.1229 | E1 Medicine or dosage form not in a completed round |
| 548 | Wang W; Zhang S; Dou C; Hu B; Song H; Qi F | Pharmacokinetics, Safety, and Immunogenicity of a Biosimilar of Nivolumab (LY01015): A Randomized, Double-Blind, Parallel-Controlled Phase I Clinical | BioDrugs : clinical immunotherapeutics, biopharmaceuticals a | 10.1007/s40259-024-00679-w | E1 Medicine or dosage form not in a completed round |
| 549 | Liu Y; Tomlinson B; Guo J; Asgharnejad M; Bauer L; Surmann E | Pharmacokinetics, Tolerability, and Bioequivalence of Two Formulations of Rotigotine in Healthy Chinese Subjects. | Clinical therapeutics | 10.1016/j.clinthera.2018.05.009 | E1 Medicine or dosage form not in a completed round |
| 550 | Guo Y; Guo T; Di Y; Xu W; Hu Z; Xiao Y | Pharmacokinetics, pharmacodynamics, safety and immunogenicity of recombinant, fully human anti-RANKL monoclonal antibody (MW031) versus denosumab in C | Expert opinion on biological therapy | 10.1080/14712598.2023.2178298 | E1 Medicine or dosage form not in a completed round |
| 551 | Wang S; Yang X; Huang J; Yang S; Wu Q; Chen H | Pharmacokinetics, pharmacodynamics, safety, and immunogenicity of a biosimilar of denosumab (LY06006): a randomized, double-blind, single-dose, parall | Expert opinion on investigational drugs | 10.1080/13543784.2022.2120389 | E1 Medicine or dosage form not in a completed round |
| 552 | Qin H; Zheng L; Fang Y; Liu Y; Liu A; Wu J | Pharmacokinetics, safety and bioequivalence of two formulations of progesterone soft capsule in healthy Chinese postmenopausal females: Impacts of a h | Basic & clinical pharmacology & toxicology | 10.1111/bcpt.13687 | E1 Medicine or dosage form not in a completed round |
| 553 | Zhang H; Wu M; Sun J; Zhu X; Li C; Ding Y | Pharmacokinetics, safety, and immunogenicity of HLX03, an adalimumab biosimilar, compared with reference biologic in healthy Chinese male volunteers: | Pharmacology research & perspectives | 10.1002/prp2.733 | E1 Medicine or dosage form not in a completed round |
| 559 | Singh A; Kalaivani M; Srivastava S; Goyal RK; Gupta SK | Postmarketing Safety of Biosimilars: Current Status, Challenges, and Opportunities in the Spontaneous Reporting System. | Therapeutic innovation & regulatory science | 10.1177/2168479019872144 | E1 Medicine or dosage form not in a completed round |
| 560 | Yu Y; Gong D; Zhu Y; Wei W; Sun G | Quality consistency evaluation of Isatidis Folium combined with equal weight quantified ratio fingerprint method and determination of antioxidant acti | Journal of chromatography. B, Analytical technologies in the | 10.1016/j.jchromb.2018.07.031 | E1 Medicine or dosage form not in a completed round |
| 561 | Sun Q; Wang B; Xu L; Xiong L; Guo D; Cui W | Quality consistency evaluation on three species of North Patrininae herba by high-performance liquid chromatography coupled with electrospray ion trap | Journal of separation science | 10.1002/jssc.202200097 | E1 Medicine or dosage form not in a completed round |
| 562 | Li WJ; Cai YF; Ouyang Y; Li XY; Shi XL; Cao SX | Quality evaluation of Angelica Sinensis Radix dispensing granules by integrating microvascular activity and chemical analysis. | Journal of ethnopharmacology | 10.1016/j.jep.2023.117236 | E1 Medicine or dosage form not in a completed round |
| 563 | Liang S; Rong S | Quantitative evaluation of the central and local community-based home care policy in China based on the PMC Index model. | Frontiers in public health | 10.3389/fpubh.2025.1693612 | E1 Medicine or dosage form not in a completed round |
| 564 | Ju Y; Yin C; Zhang Y; Meng X; Zhao L; Hu L | Rapid detection and quality evaluation of Shuang-Huang-Lian injection by ATR-IR and NIR spectroscopy combined with chemometrics. | Spectrochimica acta. Part A, Molecular and biomolecular spec | 10.1016/j.saa.2022.122008 | E1 Medicine or dosage form not in a completed round |
| 565 | Zhao J; Tian G; Qu H | Research on comprehensive quality consistency evaluation strategy for TCM Granules: A case study with sugar-free Yangwei Granules produced by fluid-be | Chinese herbal medicines | 10.1016/j.chmed.2024.11.001 | E1 Medicine or dosage form not in a completed round |
| 567 | Zhang H; Li C; Liu J; Wu M; Li X; Zhu X | Safety and pharmacokinetics of a biosimilar of denosumab (KN012): Phase 1 and bioequivalence study in healthy Chinese subjects. | Expert opinion on investigational drugs | 10.1080/13543784.2021.1863371 | E1 Medicine or dosage form not in a completed round |
| 568 | El Baghdady A; Lledó-García R; Gayfieva M; Lowcock R; Watana | Safety, Tolerability, Pharmacokinetics, and Pharmacodynamics of the Neonatal Fc Receptor Inhibitor Rozanolixizumab: An Ethnic Sensitivity Study in Hea | Clinical pharmacology in drug development | 10.1002/cpdd.1484 | E1 Medicine or dosage form not in a completed round |
| 574 | Zhang J; Zhang Z; Wang Z; Zhang T; Zhou Y; Chen M | Targeted trace ingredients coupled with chemometric analysis for consistency evaluation of Panax notoginseng saponins injectable formulations. | Chinese journal of natural medicines | 10.1016/S1875-5364(23)60396-6 | E1 Medicine or dosage form not in a completed round |
| 577 | Wu J; Dong L; Sun Y; Zhao X; Gan J; Wang Z | The Effectiveness of Artificial Intelligence in Assisting Mothers with Assessing Infant Stool Consistency in a Breastfeeding Cohort Study in China. | Nutrients | 10.3390/nu16060855 | E1 Medicine or dosage form not in a completed round |
| 578 | Wang M; Yang Y; Hu W; Wang Y; Chen Y; Zhang X | The Pharmacokinetics and Bioequivalence of Desvenlafaxine Succinate in Chinese Healthy Subjects Under Fasting and Fed States. | Clinical pharmacology in drug development | 10.1002/cpdd.1272 | E1 Medicine or dosage form not in a completed round |
| 581 | Qiu B; Liu G; Wang C; Chen X; Liu R; Huang Y | Three-Period Bioequivalence Study of Sodium Levofolinate Injection With Calcium Levofolinate for Injection and Sodium Folinate for Injection in Health | Clinical pharmacology in drug development | 10.1002/cpdd.1223 | E1 Medicine or dosage form not in a completed round |
| 582 | Zhang H; Li Q; Zhu X; Li C; Li X; Liu C | Tolerance, variability, and pharmacokinetics of bevacizumab biosimilars in Chinese healthy male subjects. | Cancer chemotherapy and pharmacology | 10.1007/s00280-018-3645-1 | E1 Medicine or dosage form not in a completed round |
| 584 | Zhang Y; Wang C; Yang F; Yang Z; Wang F; Sun G | UHPLC-ESI-Q-TOF-MS/MS analysis, antioxidant activity combined fingerprints for quality consistency evaluation of compound liquorice tablets. | RSC advances | 10.1039/c8ra02431f | E1 Medicine or dosage form not in a completed round |
| 587 | Lei ZX; Sang D; Lan B; Fan Y; Cai RG; Luo Y | [Comparison of the efficacy, safety, and cost-effectiveness of trastuzumab biosimilar HLX02 and the originator combined with pertuzumab and chemothera | Zhonghua zhong liu za zhi [Chinese journal of oncology] | 10.3760/cma.j.cn112152-20241115-00503 | E1 Medicine or dosage form not in a completed round |
| 591 | 唐爱琼; 罗军 | 7-二氟亚甲基-5,4’-二甲氧基金雀异黄素对大鼠压力性尿失禁模型的疗效及其机制 | 中南大学学报（医学版） |  | E1 Medicine or dosage form not in a completed round |
| 602 | 杨照; 刘扬; 马俊; 江滨 | 武汉:“战略性购买”先行一步 | 中国卫生 |  | E1 Medicine or dosage form not in a completed round |
| 615 | 孟宇成; 张倩倩; 陆雯婷; 赵亮 | 2020-2023年全国医药信息网样本医院利妥昔单抗使用情况分析 | 科技与健康 | 10.3969/j.issn.2097-2199.2025.15.012 | E1 Medicine or dosage form not in a completed round |
| 616 | 叶万玲; 苏晓绚; 郑金丹; 卢伟锋 | 2021-2023年海南省某三甲医院乳腺癌患者住院费用构成及影响因素分析 | 中国病案 | 10.3969/j.issn.1672-2566.2024.12.026 | E1 Medicine or dosage form not in a completed round |
| 623 | 杨亚文 | A公司人工晶状体产品市场营销策略研究 |  |  | E1 Medicine or dosage form not in a completed round |
| 625 | 薛笑怡 | A医疗器械公司内部控制活动改进研究 |  |  | E1 Medicine or dosage form not in a completed round |
| 627 | 聂尧 | A煤炭集团公司集中采购内部控制优化研究 |  |  | E1 Medicine or dosage form not in a completed round |
| 628 | 罗骏 | A电力企业物资集中采购流程优化研究 |  |  | E1 Medicine or dosage form not in a completed round |
| 629 | 肖宽标 | A餐饮集团公司集中采购管理研究 |  |  | E1 Medicine or dosage form not in a completed round |
| 632 | 李佳坤; 刘艳青; 杜方; 余振华; 冯宇; 王慧 | BrainTumorLLM:面向脑肿瘤诊疗的大语言模型优化与评估 | 计算机工程 | 10.19678/j.issn.1000-3428.0252472 | E1 Medicine or dosage form not in a completed round |
| 635 | 门秋莉 | B医疗器械公司新产品研发项目管理后评价研究 |  |  | E1 Medicine or dosage form not in a completed round |
| 636 | 于夫 | B集团供应商评价改进研究 |  |  | E1 Medicine or dosage form not in a completed round |
| 638 | 朱巧露 | CM物流公司运输服务采购管理改进研究 |  |  | E1 Medicine or dosage form not in a completed round |
| 639 | Mi Qiu; Jionglan Wan; Chunxiang Hao; Zixin Zeng; Yalong Hu; | Comprehensive Transcriptomics, Hormone Metabolomics, and Physiological Analysis Reveal the Mechanism of Exogenous GA4+7 Breaking the Seed Dormancy in | Horticulturae | 10.3390/horticulturae11060627 | E1 Medicine or dosage form not in a completed round |
| 649 | 黄华毕 | EPC总承包模式下工程物资集中采购与现场精细化管控策略 | 2026中国国土经济学会会议——“智慧城市建设与创新发展论坛”(三) |  | E1 Medicine or dosage form not in a completed round |
| 651 | Marchioretto, Lucas De Ross; De Rossi, Andrea; do Amaral, Le | Efficacy and mode of action of blossom thinners on 'Fuji More' apple trees | Scientia horticulturae | 10.1016/j.scienta.2018.11.039 | E1 Medicine or dosage form not in a completed round |
| 652 | Diksha Dhiman; Neena Chauhan; Pramod Verma; Sanjana Singh; N | Exogenous Gibberellin4+7 and Benzyladenine Application Promotes Return Bloom, Vegetative Growth, Fruit Set and Yield of Apple (Malus x domestica Borkh | Applied Fruit Science | 10.1007/s10341-025-01494-5 | E1 Medicine or dosage form not in a completed round |
| 653 | 杨明晓; 常杰; 孙立; 李然 | GB/T 43711-2024标准下框架协议采购订单动态匹配模型研究 | 中国标准化 | 10.3969/j.issn.1002-5944.2025.z1.009 | E1 Medicine or dosage form not in a completed round |
| 654 | 黄岩 | GH公司军品元器件采购流程优化研究 |  |  | E1 Medicine or dosage form not in a completed round |
| 659 | 刘钦侠 | HY公司A产品生产能力改善研究 |  |  | E1 Medicine or dosage form not in a completed round |
| 660 | 马生俊; 陈旺虎; 郭宏乐; 乔保民; 李新田 | Hadoop集群中影响应用性能的因素分析 | 小型微型计算机系统 | 10.3969/j.issn.1000-1220.2018.04.018 | E1 Medicine or dosage form not in a completed round |
| 661 | 沈伟亮 | H公司眼科业务竞争战略优化研究 |  |  | E1 Medicine or dosage form not in a completed round |
| 662 | 张怡 | H公司采购管理优化研究 |  |  | E1 Medicine or dosage form not in a completed round |
| 663 | 王婧 | J公司销售人员职业生涯管理优化策略研究 |  |  | E1 Medicine or dosage form not in a completed round |
| 664 | 徐源; 康鸿淼; 宋胜利; 孙红梅 | LA百合'Eyeliner'种球高效繁育体系建立 | 上海农业学报 | 10.15955/j.issn1000-3924.2026.03.02 | E1 Medicine or dosage form not in a completed round |
| 665 | 刘世澎 | L公司药用玻璃营销策略研究 |  |  | E1 Medicine or dosage form not in a completed round |
| 666 | 伍锦良 | M企业药包材生产质量改进研究 |  |  | E1 Medicine or dosage form not in a completed round |
| 667 | 王立然 | M公司采购中台价值研究与运行机制优化 |  |  | E1 Medicine or dosage form not in a completed round |
| 668 | 马越 | M公司采购供应商选择研究 |  |  | E1 Medicine or dosage form not in a completed round |
| 669 | 许婷 | M公司锂原料的采购成本控制研究 |  |  | E1 Medicine or dosage form not in a completed round |
| 670 | 王法罡 | M公司锂电池采购成本控制研究 |  |  | E1 Medicine or dosage form not in a completed round |
| 671 | 朱旭 | M公司隐性采购成本降低策略研究 |  |  | E1 Medicine or dosage form not in a completed round |
| 673 | 陈博文 | M自动驾驶公司基于物料分类的多源采购订单分配决策优化 |  |  | E1 Medicine or dosage form not in a completed round |
| 675 | 刘婧 | N集团集中采购内部控制体系优化研究 |  |  | E1 Medicine or dosage form not in a completed round |
| 677 | Francisco José Domingues Neto; Débora Cavalcante dos Santos | Physiological Indexes in Seed Germination and Seedling Growth of Rangpur Lime (Citrus limonia L. Osbeck) under Plant Growth Regulators | Agronomy | 10.3390/agronomy14092066 | E1 Medicine or dosage form not in a completed round |
| 679 | 胡静 | SPD供应链管理模式在医用耗材管理中的运用研究 | 信息技术时代 | 10.3969/j.issn.1671-153X.2022.17.027 | E1 Medicine or dosage form not in a completed round |
| 680 | 胡红梅 | SPD在某公立医院医用耗材管理的应用实践探索 | 中国医疗管理科学 | 10.3969/j.issn.2095-7432.2025.04.011 | E1 Medicine or dosage form not in a completed round |
| 682 | 丁凯宗 | S医疗公司脑室引流产品营销策略研究 |  |  | E1 Medicine or dosage form not in a completed round |
| 685 | 梁以萍 | W公司基层销售经理培训体系优化研究 |  |  | E1 Medicine or dosage form not in a completed round |
| 686 | 张俊岿 | W公司销售与运营计划优化管理研究 |  |  | E1 Medicine or dosage form not in a completed round |
| 688 | 董锴 | X公司集中采购订单分配优化研究 |  | 10.27151/d.cnki.ghnlu.2024.003937 | E1 Medicine or dosage form not in a completed round |
| 689 | 王红梅 | Y公司体外诊断试剂库存管理优化研究 |  |  | E1 Medicine or dosage form not in a completed round |
| 708 | 王祺 | 一种基于最优传输理论的类别均衡化迁移学习方法 |  |  | E1 Medicine or dosage form not in a completed round |
| 713 | 谢菡; 陈思煌; 陈大宇; 李俐; 葛卫红 | 不同厂家氟比洛芬酯注射液有效性和安全性的真实世界研究 | 医药导报 | 10.3870/j.issn.1004-0781.2023.02.010 | E1 Medicine or dosage form not in a completed round |
| 717 | 马勉娣; 张丹; 王永旭; 马静; 胡志芳; 王兴鹏 | 不同果锈防治剂对”维纳斯黄金”苹果果锈及果实品质的影响 | 中国南方果树 | 10.13938/j.issn.1007-1431.20250088 | E1 Medicine or dosage form not in a completed round |
| 719 | 马媛媛; 王君正 | 不同植物生长调节剂组合对芒果膨大效果及品质的影响 | 现代农业科技 | 10.3969/j.issn.1007-5739.2025.20.018 | E1 Medicine or dosage form not in a completed round |
| 722 | 雷凯 | 个性化3D术前规划等智能精准辅助技术在TKA中的应用及其临床疗效对比研究 |  |  | E1 Medicine or dosage form not in a completed round |
| 723 | 宋昊; 范元清; 张成江; 谭亚辉; 徐争启; 侯明才 | 中国北方砂岩型铀矿床工艺矿物学研究若干进展 | 矿物岩石 | 10.19719/j.cnki.1001-6872.2024.01.02 | E1 Medicine or dosage form not in a completed round |
| 737 | 马艺轩 | 乐普医疗连续并购价值创造研究--基于资源编排视角 |  |  | E1 Medicine or dosage form not in a completed round |
| 738 | 陈妍 | 九州通医药供应链金融信用风险的评估与防范 |  |  | E1 Medicine or dosage form not in a completed round |
| 740 | 俞骏仁; 姜若; 罗莉; 李志建 | 人工关节类耗材集中采购对某三甲医院关节置换手术的影响 | 中国医院管理 |  | E1 Medicine or dosage form not in a completed round |
| 741 | 李秋俞; 杨转红; 马崇淇; 邓荣豪; 康周 | 人工关节集采前后相关病组住院费用结构分析 | 中国医疗保险 | 10.19546/j.issn.1674-3830.2024.10.016 | E1 Medicine or dosage form not in a completed round |
| 761 | 杨远林 | 企业财务风险管理中的成本控制策略研究 | 首席财务官 | 10.12317/j.issn.1673-3169.2025.18.016 | E1 Medicine or dosage form not in a completed round |
| 764 | 汪若晨 | 优化模糊决策树在医学数据分类中的研究与应用 |  |  | E1 Medicine or dosage form not in a completed round |
| 766 | 李蕾 | 供应链环境下心脏介入手术器械多级库存优化研究 |  |  | E1 Medicine or dosage form not in a completed round |
| 773 | 王兴慧 | 全链管理项目物资集中采购模式及其优缺点 | 价值工程 | 10.3969/j.issn.1006-4311.2025.10.020 | E1 Medicine or dosage form not in a completed round |
| 774 | 彭琳彦 | 公共图书馆带量采购模式及发展路径探索 | 图书馆研究与工作 | 10.3969/j.issn.2096-2363.2026.03.009 | E1 Medicine or dosage form not in a completed round |
| 777 | 杨春芬 | 公立医院物资管理内部控制问题及对策探讨 | 财经界 | 10.3969/j.issn.1009-2781.2026.07.050 | E1 Medicine or dosage form not in a completed round |
| 778 | 李名家 | 公立医院财务管理方法刍议——以医用耗材带量采购为例 | 华章 |  | E1 Medicine or dosage form not in a completed round |
| 779 | 杨占戈 | 关于政府采购过程中的绩效问题研究 |  |  | E1 Medicine or dosage form not in a completed round |
| 782 | 邵祯谊; 陆东哲; 沈洁; 薛迪 | 冠状动脉支架集中带量采购政策对上海市冠状动脉粥样硬化性心脏病住院患者医疗费用的影响 | 中国卫生资源 | 10.13688/j.cnki.chr.2023.230202 | E1 Medicine or dosage form not in a completed round |
| 788 | 邵佳莉; 张建科; 吴远红 | 加密二维码在海产品质量溯源系统中的应用 | 现代食品 | 10.16736/j.cnki.cn41-1434/ts.2023.19.024 | E1 Medicine or dosage form not in a completed round |
| 789 | 何锦 | 加强建筑施工企业工程项目资产管理的思考 | 经营者 |  | E1 Medicine or dosage form not in a completed round |
| 790 | 袁志远; 高杰; 杨才君 | 动态不确定性-药品物流多中心选址优化研究 | 运筹与管理 | 10.12005/orms.2023.0247 | E1 Medicine or dosage form not in a completed round |
| 801 | 陶志煜 | 医用输注器具柔性作业车间动态调度问题研究 |  |  | E1 Medicine or dosage form not in a completed round |
| 802 | 郑佳 | 医疗器械企业ERP环境下的内部控制活动风险及对策研究--以M企业为例 |  |  | E1 Medicine or dosage form not in a completed round |
| 803 | 张宇轩 | 医疗器械企业跨国并购的动因与绩效分析——以迈瑞医疗为例 |  |  | E1 Medicine or dosage form not in a completed round |
| 821 | 王倩 | 压缩感知重建算法及在图像重建中的应用研究 |  |  | E1 Medicine or dosage form not in a completed round |
| 826 | 李芳; 权婉珍; 王鹏发; 陈相兵; 樊海兵; 叶涛 | 口腔专科医院医用耗材使用分析方案与实践策略 | 空军军医大学学报 | 10.13276/j.issn.2097-1656.2025.10.010 | E1 Medicine or dosage form not in a completed round |
| 860 | 兰建平 | 在发展中调整在调整中发展 ——当前浙江工业经济运行分析 | 浙江经济 |  | E1 Medicine or dosage form not in a completed round |
| 862 | 时志雄; 朱峰; 刘舒; 符杨; 田书欣 | 基于1DCNN-DACLSTM模型的风电超短期功率预测方法 | 电子器件 | 10.3969/j.issn.1005-9490.2024.01.033 | E1 Medicine or dosage form not in a completed round |
| 867 | 丁枭 | 基于CenterNet网络模型的发电机组叶轮缺陷检测研究 |  |  | E1 Medicine or dosage form not in a completed round |
| 872 | 李沐阳 | 基于EER--PPO算法的自主水下机器人路径跟踪及自主避障研究 |  |  | E1 Medicine or dosage form not in a completed round |
| 873 | 罗冰瑶 | 基于Hadoop平台的公路路网数据管理系统的设计与实现 |  |  | E1 Medicine or dosage form not in a completed round |
| 923 | 陈咪 | 基于多传感器融合的安防机器人的导航系统研究 |  |  | E1 Medicine or dosage form not in a completed round |
| 1407 | Hoeh, B; Hohenhorst, JL; Wenzel, M; Humke, C; Preisser, F; W | Full functional-length urethral sphincter- and neurovascular bundle preservation improves long-term continence rates after robotic-assisted radical pr | JOURNAL OF ROBOTIC SURGERY | 10.1007/s11701-022-01408-7 | E1 Medicine or dosage form not in a completed round |
| 1431 | Zhu, Y; Li, C; Chen, LM; Liu, HY; Ou, L; Li, T | A Phase I Clinical Study Comparing the Pharmacokinetics, Safety, and Immunogenicity of GB221 Injection and Trastuzumab (Herceptin®) in Healthy Chinese | EUROPEAN JOURNAL OF DRUG METABOLISM AND PHARMACOKINETICS | 10.1007/s13318-024-00889-1 | E1 Medicine or dosage form not in a completed round |
| 1432 | Wang, J; Qi, L; Liu, L; Wang, ZJ; Chen, G; Wang, Y | A Phase I, Randomized, Single-Dose Study Evaluating the Biosimilarity of TAB008 to Bevacizumab in Healthy Volunteers | FRONTIERS IN PHARMACOLOGY | 10.3389/fphar.2019.00905 | E1 Medicine or dosage form not in a completed round |
| 1433 | Zhang, H; Chen, H; Li, XJ; Wu, M; Zhu, XX; Li, CY | A Phase I, Randomized, Single-Dose Study to Evaluate the Biosimilarity of HOT-3010 to Adalimumab Among Healthy Chinese Male Subjects | FRONTIERS IN PHARMACOLOGY | 10.3389/fphar.2021.646171 | E1 Medicine or dosage form not in a completed round |
| 1443 | Xing, ZM; Gao, H; Wang, D; Shang, Y; Tuliebieke, T; Jiang, J | A novel biological sources consistency evaluation method reveals high level of biodiversity within wild natural medicine: A case study of Amynthas ear | ACTA PHARMACEUTICA SINICA B | 10.1016/j.apsb.2022.10.024 | E1 Medicine or dosage form not in a completed round |
| 1447 | Ding, FF; Huang, J; Feng, ZY; Kuang, Y; Yang, S; Xiang, YX | A pharmacokinetic study to comparatively evaluate the bioequivalence and safety of a humanized recombinant monoclonal antibody targeting human epiderm | INVESTIGATIONAL NEW DRUGS | 10.1007/s10637-022-01220-y | E1 Medicine or dosage form not in a completed round |
| 1448 | Li, Y; Wang, Y; Yang, CX; Zhao, N; Wang, XH | A phase I, randomized, double-blind, parallel, single-dose pharmacokinetic study to evaluate the biosimilarity of KM118 (proposed pertuzumab biosimila | ANNALS OF MEDICINE | 10.1080/07853890.2025.2523561 | E1 Medicine or dosage form not in a completed round |
| 1449 | Zhang, H; Zhu, XX; Wei, HJ; Li, CY; Chen, H; Li, XJ | A phase I, randomized, double-blinded, single-dose study evaluating the pharmacokinetic equivalence of the biosimilar IBI305 and bevacizumab in health | INTERNATIONAL JOURNAL OF CLINICAL PHARMACOLOGY AND THERAPEUT | 10.5414/CP203349 | E1 Medicine or dosage form not in a completed round |
| 1482 | Li, YT; Huang, YY; Fu, XH; Xia, JJ; Su, JF; Gu, WZ | Bioequivalence Study of Atenolol Tablets in Healthy Chinese Subjects Under Fasting and Fed Conditions | CLINICAL PHARMACOLOGY IN DRUG DEVELOPMENT | 10.1002/cpdd.1414 | E1 Medicine or dosage form not in a completed round |
| 1487 | Li, MP; Yi, XC; Fan, LL; Yang, LX; Xie, S; Shentu, JZ | Bioequivalence Study of Two Formulations of Mifepristone Tablets in Healthy Chinese Subjects Under Fasting Conditions | CLINICAL PHARMACOLOGY IN DRUG DEVELOPMENT | 10.1002/cpdd.1249 | E1 Medicine or dosage form not in a completed round |
| 1498 | Wang, MN; Yu, HT; Li, YQ; Zeng, Y; Yang, S; Yang, GP | Bioequivalence and Pharmacokinetic Profiles of Generic and Branded Obeticholic Acid in Healthy Chinese Subjects Under Fasting and Fed Conditions | DRUG DESIGN DEVELOPMENT AND THERAPY | 10.2147/DDDT.S289016 | E1 Medicine or dosage form not in a completed round |
| 1499 | Liu, LH; Li, X; Liu, YJ; Xu, B; Li, Y; Yuan, F | Bioequivalence and Pharmacokinetic Study of 2 Edoxaban Tablets in Healthy Chinese Subjects | CLINICAL PHARMACOLOGY IN DRUG DEVELOPMENT | 10.1002/cpdd.1156 | E1 Medicine or dosage form not in a completed round |
| 1751 | Li, X; Li, Y; Xu, B; Zhang, P; Wang, YY; Wang, ZY | Pharmacokinetics and Bioequivalence of a Generic and a Branded Budesonide Nasal Spray in Healthy Chinese Subjects | CLINICAL PHARMACOLOGY IN DRUG DEVELOPMENT | 10.1002/cpdd.1036 | E1 Medicine or dosage form not in a completed round |
| 225 | 赵江林; 张晟肇; 魏琪格; 贾艳艳; 袁涛 | 国产与进口艾司奥美拉唑治疗上消化道出血的药物经济学评价 | 中国药物与临床 |  | E2 Study period preceding the relevant round |
| 226 | 廖荣琼 | 国产仿制硼替佐米治疗多发性骨髓瘤的临床观察及瘤细胞表面CD28和CD117分子表达在预后判断中的价值 |  | 10.27755/d.cnki.gcbyx.2021.000107 | E2 Study period preceding the relevant round |
| 229 | 黄芮 | 国产和原研硼替佐米治疗初诊多发性骨髓瘤的疗效与安全性观察 |  | 10.26921/d.cnki.ganyu.2021.001300 | E2 Study period preceding the relevant round |
| 238 | 顾玉兰 | 奥美拉唑肠溶胶囊质量和疗效一致性评价 |  | 10.27307/d.cnki.gsjtu.2019.004856 | E2 Study period preceding the relevant round |
| 254 | 洪明明; 龚源 | 莫西沙星仿制药与原研药用于非重症社区获得性肺炎的疗效及安全性回顾性对比研究 | 中国医院用药评价与分析 | 10.14009/j.issn.1672-2124.2019.09.017 | E2 Study period preceding the relevant round |
| 340 | Wang J; Lai X; Yao H; Yang H; Cao X; Wang X | A multicenter, randomized, open label, two formulation, crossover bioequivalence trial of doxorubicin hydrochloride liposomal injection in Chinese pat | Journal of cancer research and clinical oncology | 10.1007/s00432-025-06086-0 | E2 Study period preceding the relevant round |
| 409 | Hu C; Lin Q; Liu C; Liu J; Chen X; Li X | Bioequivalence study of 20-mg and 100-mg temozolomide capsules (TOZ309 and Temodal(®)) in glioma patients in China. | Cancer chemotherapy and pharmacology | 10.1007/s00280-020-04175-0 | E2 Study period preceding the relevant round |
| 417 | Liang Y; Zhu Y; Zhang Y; Chen Z; Li B; Liu A | Clinical Observation and Pharmacoeconomic Evaluations of Original Research Drug and Generic Drug Bortezomib in the Treatment of Multiple Myeloma. | Journal of healthcare engineering | 10.1155/2022/5201354 | E2 Study period preceding the relevant round |
| 418 | Huang T; Bai L; Wushouer H; Wang Z; Yang M; Lin H | Clinical Outcome and Medical Cost of Originator and Generic Antihypertensive Drugs: A Population-Based Study in Yinzhou, China. | Frontiers in pharmacology | 10.3389/fphar.2022.757398 | E2 Study period preceding the relevant round |
| 450 | Ji L; Gao L; Cheng Z; Ma G; Li S; Wang H | Efficacy and safety of insulin degludec biosimilar B01411 versus originator insulin degludec in Chinese patients with type 2 diabetes inadequately con | Current medical research and opinion | 10.1080/03007995.2024.2401096 | E2 Study period preceding the relevant round |
| 463 | Zhang SY; Tao LY; Yang YY; Kong T; Wu CJ; Wang Y | Evaluation of blood pressure lowering effect by generic and brand-name antihypertensive drugs treatment: a multicenter prospective study in China. | Chinese medical journal | 10.1097/CM9.0000000000001360 | E2 Study period preceding the relevant round |
| 470 | Wang W; Song X; Lou Y; Du L; Zhu D; Zhou Z | Immunogenicity of LY2963016 insulin glargine and Lantus® insulin glargine in Chinese patients with type 1 or type 2 diabetes mellitus. | Diabetes, obesity & metabolism | 10.1111/dom.14674 | E2 Study period preceding the relevant round |
| 573 | Hu X; Zhang L; Dong Y; Dong C; Jiang J; Gao W | Switching from biosimilar (Basalin) to originator (Lantus) insulin glargine is effective in Chinese patients with diabetes mellitus: a retrospective c | F1000Research | 10.12688/f1000research.13923.1 | E2 Study period preceding the relevant round |
| 575 | Chen XF; Fan YN; Si CW; Yu YY; Shang J; Yu ZJ | Tenofovir disoproxil fumarate in Chinese chronic hepatitis B patients: Results of a multicenter, double-blind, double-dummy, clinical trial at 96 week | World journal of clinical cases | 10.12998/wjcc.v9.i18.4690 | E2 Study period preceding the relevant round |
| 586 | He L; Du X; Wang WH; Ma CS | [Comparison of compliance and cost effectiveness between brand-name statins and generic statins]. | Zhonghua liu xing bing xue za zhi = Zhonghua liuxingbingxue | 10.3760/cma.j.cn112338-20191114-00809 | E2 Study period preceding the relevant round |
| 1272 | 李敏; 张曼曼 | 集采与原研头孢哌酮-舒巴坦钠治疗细菌性感染患者的成本效果分析 | 抗感染药学 | 10.13493/j.issn.1672-7878.2024.06-008 | E2 Study period preceding the relevant round |
| 2 | 张莉; 罗伟; 刘仔; 陈桂香; 李凤仪 | 5032例东莞市国家集中带量采购药品的药品不良反应报告分析 | 中国医院用药评价与分析 | 10.14009/j.issn.1672-2124.2024.12.025 | E3 No eligible comparator |
| 77 | 陈春燕; 朱青梅; 杨东亮; 单慧亭; 赵海霞; 熊代琴 | 国家集采白蛋白结合型紫杉醇仿制药的不良反应及危险因素分析 | 中国临床药学杂志 | 10.19577/j.1007-4406.2023.01.005 | E3 No eligible comparator |
| 86 | 林婷婷; 蔡立婧; 张梦颖 | 基于国家胰岛素集采下胰岛素转换替代的有效性和安全性研究 | 海峡药学 | 10.3969/j.issn.1006-3765.2025.02.014 | E3 No eligible comparator |
| 94 | 崔朴梅; 吴瑞; 林洁; 李晓东 | 基于真实世界数据的集中带量采购注射用左氧氟沙星治疗细菌性感染的临床综合评价 | 中南药学 | 10.7539/j.issn.1672-2981.2025.07.047 | E3 No eligible comparator |
| 138 | 丛美洁 | 改良二联疗法和改良二联疗法联合蒲地蓝根除幽门螺杆菌的临床疗效分析 |  | 10.27749/d.cnki.gbzyx.2022.000020 | E3 No eligible comparator |
| 172 | 周敏华; 冼冬妍; 王艳; 林江涛; 朱瑜 | 药品集中带量采购背景下智能慢病用药教育模式的构建与实践 | 北方药学 | 10.3969/j.issn.1672-8351.2023.10.026 | E3 No eligible comparator |
| 234 | 李明勇; 石雅宁; 王晖; 覃丽 | 基于VASP评估氯吡格雷抗血小板疗效方法的建立及应用 | 沈阳药科大学学报 | 10.14066/j.cnki.cn21-1349/r.2023.0593 | E3 No eligible comparator |
| 263 | Tian S; Zhong H; Yin M; Jiang P; Liu Q | A China-Based Cost-Effectiveness Analysis of Novel Oral Anticoagulants versus Warfarin in Patients with Left Ventricular Thrombosis. | Risk management and healthcare policy | 10.2147/RMHP.S454463 | E3 No eligible comparator |
| 277 | Chen M; Liu L; Zhang L; Lin Y; Lu X; Yang H | Cost-effectiveness analysis of imatinib versus dasatinib in the treatment of pediatric Philadelphia chromosome-positive acute lymphoblastic leukemia w | BMC health services research | 10.1186/s12913-023-09600-7 | E3 No eligible comparator |
| 278 | Zhang H; Chen J; Zhao Q; Zhang B | Cost-utility analysis of empagliflozin compared with dapagliflozin in patients with type 2 diabetes in China. | Frontiers in public health | 10.3389/fpubh.2025.1566101 | E3 No eligible comparator |
| 286 | Zhou L; Gu B; Wang J; Liu G; Zhang X | Human papillomavirus vaccination at the national and provincial levels in China: a cost-effectiveness analysis using the PRIME model. | BMC public health | 10.1186/s12889-022-13056-5 | E3 No eligible comparator |
| 291 | Li K; Xiang X; Zeng W; Li G; Shi J; Gao X | Impact of diagnosis-related group-based bundled payment on hospitalization costs and clinical outcomes in total hip arthroplasty: an interrupted time | Frontiers in public health | 10.3389/fpubh.2026.1886645 | E3 No eligible comparator |
| 297 | Xiao D; Weng J; Zhang L; Xing C; Wei Y; Chen Y | Long term cost-effectiveness analysis of IDegLira in the treatment of type 2 diabetes patients compared to GLP-1RA added to basal insulin after IDegLi | PloS one | 10.1371/journal.pone.0310497 | E3 No eligible comparator |
| 301 | Zhou D; Zhang D; Wang Y; Zhou K; Tang W | Optimizing strategy for cervical cancer prevention in china: a comprehensive modeling analysis. | Cost effectiveness and resource allocation : C/E | 10.1186/s12962-025-00630-y | E3 No eligible comparator |
| 305 | Du Z; Jiang Y; Shen Y; Zhou Q; Wang S; Zhu H | Reevaluation of adverse drug reactions of psychiatric drugs under the chinese drug volume-based procurement policy. | BMC health services research | 10.1186/s12913-022-07851-4 | E3 No eligible comparator |
| 307 | Ding G; Ding J | Research and practice on key drug safety monitoring under the normalization of centralized procurement: A case study of 13 selected drugs in a Provinc | Medicine | 10.1097/MD.0000000000046379 | E3 No eligible comparator |
| 310 | Lin Y; Lin X; Xia R; Chen J; Lin Z; Lu S | Tenofovir alafenamide is superior to tenofovir disoproxil fumarate and entecavir in cost-effectiveness of treatment of chronic hepatitis B in china wi | PloS one | 10.1371/journal.pone.0327298 | E3 No eligible comparator |
| 312 | Ma L; Zhu T; Duan J; Huang N; Zhang Z | The cost effectiveness of toripalimab plus bevacizumab versus sorafenib for the first-line treatment of advanced hepatocellular carcinoma in China. | Frontiers in pharmacology | 10.3389/fphar.2026.1726444 | E3 No eligible comparator |
| 323 | Wu G; Wu L; Zhou H; Lin M; Peng L; Wang Y | A Phase I Comparative Pharmacokinetic and Safety Study of Two Intravenous Formulations of Vinorelbine in Patients With Advanced Non-Small Cell Lung Ca | Frontiers in pharmacology | 10.3389/fphar.2019.00774 | E3 No eligible comparator |
| 353 | Li Y; Qi L; Yang C; Zhao N; Wang X | A randomized, open-label, two-period crossover study to evaluate the bioequivalence and food effect between two formulations of regorafenib in healthy | Frontiers in pharmacology | 10.3389/fphar.2025.1511558 | E3 No eligible comparator |
| 356 | Xie F; Vermeulen A; Colin P; Cheng Z | A semiphysiological population pharmacokinetic model of agomelatine and its metabolites in Chinese healthy volunteers. | British journal of clinical pharmacology | 10.1111/bcp.13902 | E3 No eligible comparator |
| 361 | Yu F; Wang R; Wang K; Lin B; Zhou X; Chen L | A study on the pharmacokinetic bioequivalence of oral tablet formulations of riluzole among healthy volunteers utilizing HPLC-MS/MS. | BMC pharmacology & toxicology | 10.1186/s40360-025-00931-1 | E3 No eligible comparator |
| 362 | Yu Y; Hu W; Zhang T; Peng Y; Ding J; Wang X | A study to evaluate the impact of PB-119 injection (a pegylated exenatide formulation) on the pharmacokinetic profiles of Digoxin and Warfarin sodium | SAGE open medicine | 10.1177/20503121261424728 | E3 No eligible comparator |
| 370 | Li Y; Ming JE; Kong F; Yin H; Zhang L; Bai H | Bioequivalence Study Comparing Fixed-Dose Combination of Clopidogrel and Aspirin with Coadministration of Individual Formulations in Chinese Subjects | Advances in therapy | 10.1007/s12325-020-01486-9 | E3 No eligible comparator |
| 371 | Wu L; Xu C; Wu G; Zhou H; Lv D; Zhai Y | Bioequivalence Study Of A Fixed-Dose Combination Tablet Containing Melitracen 10 mg And Flupentixol 0.5 mg In Healthy Chinese Volunteers Under Fasted | Drug design, development and therapy | 10.2147/DDDT.S207561 | E3 No eligible comparator |
| 381 | Tao Y; Jiang X; Shi P; Liu Y; Lin P; Liu S | Bioequivalence and Food Effect Assessment of 2 Rivaroxaban Formulations in Healthy Chinese Volunteers: An Open, Randomized, Single-Dose, and 4-Period | Clinical pharmacology in drug development | 10.1002/cpdd.1031 | E3 No eligible comparator |
| 383 | Wang J; Zhao Z; Tao Y; Lan Y | Bioequivalence and Food Effect Assessment of Eltrombopag Olamine Tablets in Healthy Chinese Subjects: An Open, Randomized, Single-Dose, and Two-Period | Clinical pharmacology in drug development | 10.1002/cpdd.1453 | E3 No eligible comparator |
| 384 | Hu M; Yang M; Zhang S; Li J; Pang H; Pei Y | Bioequivalence and Food Effect Assessment of Two Fixed-Dose Combination Formulations of Telmisartan-Hydrochlorothiazide Tablets in Chinese Healthy Sub | Clinical and translational science | 10.1111/cts.70228 | E3 No eligible comparator |
| 396 | Gao L; Hu Y; Wu D; Zheng W; Zhang H | Bioequivalence and safety of fixed-dose combination of rosuvastatin calcium and ezetimibe tablets versus coadministration of individual drugs in healt | Naunyn-Schmiedeberg's archives of pharmacology | 10.1007/s00210-025-04477-1 | E3 No eligible comparator |
| 398 | Li T; Liu YP; Liu SQ; Shi P; Jiang X; Tao Y | Bioequivalence evaluation and food effect assessment of Lisinopril/amlodipine tablets in healthy Chinese subjects under fasting and fed conditions. | BMC pharmacology & toxicology | 10.1186/s40360-022-00590-6 | E3 No eligible comparator |
| 428 | Fan N; Gongmin Z; Wenming C; Jiao Y; Ruijie Z; Yan C | Comparison of Pharmacokinetics and Bioavailability of Fixed-Dose Combination Tablet and Monotherapy Combination of Allisartan Isoproxil and Indapamide | Clinical pharmacology in drug development | 10.1002/cpdd.1327 | E3 No eligible comparator |
| 431 | Wu Y; Yin T; Jian G; Wan T; Zhou B | Cost-effectiveness analysis of direct oral anticoagulants versus low-molecular-weight heparin and no thromboprophylaxis in primary prevention of cance | Frontiers in pharmacology | 10.3389/fphar.2024.1373333 | E3 No eligible comparator |
| 432 | Jin X; Li Z | Cost-effectiveness analysis of first-line versus second-line use of CDK4/6 inhibitors combined with endocrine therapy in advanced HR+/HER2- breast can | Frontiers in pharmacology | 10.3389/fphar.2025.1700291 | E3 No eligible comparator |
| 435 | Zhao P; Qi Y | Development of a UPLC-ESI-MS/MS method for the determination of triamcinolone acetonide in human plasma and evaluation of its bioequivalence after a s | Frontiers in pharmacology | 10.3389/fphar.2023.1223112 | E3 No eligible comparator |
| 439 | Nie S; Chen K; Guo C; Pei Q; Zou C; Yao L | Effect of CYP4F2 Polymorphisms on Ticagrelor Pharmacokinetics in Healthy Chinese Volunteers. | Frontiers in pharmacology | 10.3389/fphar.2021.797278 | E3 No eligible comparator |
| 440 | Ding C; Song H; Qiu B; Sun X; Guo C; Bai W | Effect of High-Fat Food on the Pharmacokinetics and Safety of Amlodipine/Benazepril in Healthy Chinese Participants. | Clinical pharmacology in drug development | 10.1002/cpdd.70022 | E3 No eligible comparator |
| 459 | Chen Q; Yu C; Wu Q; Song R; Liu Y; Feng S | Evaluation of Drug-Drug Interaction Between Henagliflozin and Hydrochlorothiazide in Healthy Chinese Volunteers. | Drug design, development and therapy | 10.2147/DDDT.S433377 | E3 No eligible comparator |
| 473 | Han G; Hu S; Zhang X; Qiu Z; Huang Z | Insulin degludec/liraglutide versus its monotherapy on T2D patients: A lifetime cost-utility analysis in China. | Frontiers in pharmacology | 10.3389/fphar.2022.1011624 | E3 No eligible comparator |
| 474 | Wang Y; Lu J; Li T; Zhao S; Yang W; Liu L | Investigation of a potential drug-drug interaction between salbutamol and ambroxol and bioequivalence of a new fixed-dose combination containing these | International journal of clinical pharmacology and therapeut | 10.5414/CP203188 | E3 No eligible comparator |
| 494 | Li X; Mo E; Chen L | Pharmacokinetics and Bioequivalence Evaluation of 2 Olmesartan Medoxomil and Amlodipine Besylate Fixed-Dose Combination Tablets in Healthy Chinese Vol | Clinical pharmacology in drug development | 10.1002/cpdd.1086 | E3 No eligible comparator |
| 513 | Ye L; Li B; Ling N; Gao Y; Tao X; Cai S | Pharmacokinetics and Bioequivalence of a Generic Fixed-Dose Combination Tablet of Metformin Hydrochloride/Vildagliptin Versus a Branded Product in Hea | Clinical pharmacology in drug development | 10.1002/cpdd.992 | E3 No eligible comparator |
| 521 | Li X; Liu L; Deng Y; Li Y; Zhang P; Wang Y | Pharmacokinetics and bioequivalence of a generic empagliflozin tablet versus a brand-named product and the food effects in healthy Chinese subjects. | Drug development and industrial pharmacy | 10.1080/03639045.2020.1810263 | E3 No eligible comparator |
| 532 | Cheng Y; Chen M; Yu J; Gong H; Xu Z; Li Y | Pharmacokinetics and safety of the combination of KL130008 and methotrexate in rheumatoid arthritis patients: a multicenter, open-label phase Ib trial | Expert opinion on drug metabolism & toxicology | 10.1080/17425255.2025.2568188 | E3 No eligible comparator |
| 534 | Wu YX; Zhong XF; Li XM; Liu WL; Zhang YX; Shen QY | Pharmacokinetics of Nifedipine-Sustained Release Tablets in Healthy Subjects After a Single Oral Administration: Bioequivalence Analysis and Food Effe | Clinical pharmacology in drug development | 10.1002/cpdd.1269 | E3 No eligible comparator |
| 555 | Chen W; Jiang B; Ruan Z; Yang D; Hu Y; Lou H | Population pharmacokinetic analysis of febuxostat with high focus on absorption kinetics and food effect. | BMC pharmacology & toxicology | 10.1186/s40360-024-00783-1 | E3 No eligible comparator |
| 556 | Gu F; Tang K; Zhang C; Hu M; Sun J; Yu X | Population pharmacokinetic analysis of rivaroxaban in healthy volunteers and patients with radiofrequency ablation of non-valvular atrial fibrillation | Frontiers in pharmacology | 10.3389/fphar.2025.1562259 | E3 No eligible comparator |
| 557 | Chen W; Ruan Z; Chen J; Yang D; Shao R; Lou H | Population pharmacokinetics and enterohepatic recirculation of hyzetimibe and its main metabolite in Chinese healthy subjects. | British journal of clinical pharmacology | 10.1111/bcp.15187 | E3 No eligible comparator |
| 558 | Xu G; Yang D; He C; Zhong L; Zhu J; Shu Q | Population pharmacokinetics and toxicity correlation analysis of free and liposome-encapsulated doxorubicin in Chinese patients with advanced breast c | Cancer chemotherapy and pharmacology | 10.1007/s00280-023-04559-y | E3 No eligible comparator |
| 566 | Xie Q; Li Y; Liu Z; Mu G; Zhang H; Zhou S | SLC4A4, FRAS1, and SULT1A1 Genetic Variations Associated With Dabigatran Metabolism in a Healthy Chinese Population. | Frontiers in genetics | 10.3389/fgene.2022.873031 | E3 No eligible comparator |
| 570 | Yi L; Zhang H; Zhang JW; You XM; Ning ZQ; Yu J | Study on Drug-Drug Interactions Between Chiglitazar, a Novel PPAR Pan-Agonist, and Metformin Hydrochloride in Healthy Subjects. | Clinical pharmacology in drug development | 10.1002/cpdd.668 | E3 No eligible comparator |
| 571 | Wang J; Wei Y; Shi P; Chen J; Xu L; Kong Y | Study protocol of a multicenter, randomized, non-inferiority, open-label study investigating the efficacy of a fixed-dose combination regimen of dapag | Frontiers in endocrinology | 10.3389/fendo.2025.1690339 | E3 No eligible comparator |
| 596 | 陈盛; 曹旺烽; 司在武 | 基于SLC7A11/GPX4理论探究杜仲方治疗绝经后骨质疏松症的临床疗效 | 深圳中西医结合杂志 |  | E3 No eligible comparator |
| 604 | 陈杰彬; 胡蓉; 李成杰; 吕佩佳; 魏连波 | 肾衰宁片联合左卡尼汀对慢性肾脏病3-4期患者的疗效 | 实用医学杂志 |  | E3 No eligible comparator |
| 608 | 郑春茂; 欧玮; 宋香清 | 万古霉素新旧AUC_(0-24)/MIC计算模型的一致性评价及临床疗效预测价值比较 | 中国医院药学杂志 | 10.13286/j.1001-5213.2024.09.06 | E3 No eligible comparator |
| 610 | 张丽敏; 许惠文; 李虓; 孙越红; 王丹; 王平 | 光学比浊法与血栓弹力图法评价氯吡格雷疗效的一致性研究及氯吡格雷抵抗相关风险因素分析 | 国际检验医学杂志 |  | E3 No eligible comparator |
| 641 | 熊鑫; 程晓慧; 邹跃; 邹欣燃; 张欣媛 | DIP环境下用临床药学服务模式构建慢性心力衰竭临床药学路径的效果评价 | 中国医院用药评价与分析 | 10.14009/j.issn.1672-2124.2025.08.011 | E3 No eligible comparator |
| 697 | 杨龙华 | “互联网+”药物治疗管理在2型糖尿病患者中的应用研究 |  |  | E3 No eligible comparator |
| 716 | 胡云鹤; 付瑞瑛; 周玉焯; 于凡; 李爱东 | 不同手术方式对住院天数、住院费用的影响及GB1分组优化建议 | 中国医药导报 | 10.20047/j.issn1673-7210.2024.26.04 | E3 No eligible comparator |
| 769 | 张宇; 吕鹏; 李静; 张京莉 | 倾向性评分匹配法对集中采购注射用头孢哌酮钠舒巴坦钠用于成人肺部感染的疗效与安全性评价 | 山西医药杂志 | 10.3969/j.issn.0253-9926.2025.06.001 | E3 No eligible comparator |
| 781 | 杨胜男; 世伟; 赵玉芳; 刘治恩; 雷雯朴; 张亚男 | 内蒙古地区吉西他滨的药物不良反应及危险因素分析 | 中国药房 | 10.6039/j.issn.1001-0408.2025.04.17 | E3 No eligible comparator |
| 784 | 张敬 | 分区生存模型在缺血性脑卒中二级预防药物经济学评价中的应用 |  |  | E3 No eligible comparator |
| 857 | 顾秋君; 张颖; 王蕾; 徐蕾; 陈志高 | 国家集采白蛋白结合型紫杉醇在转移性乳腺癌治疗中的有效性、安全性和经济性评价 | 科技与健康 |  | E3 No eligible comparator |
| 1427 | Di, YJ; Wang, ZJ; Jia, CD; Xie, X; Yang, SS; Wang, WH | A Bioequivalence Study of Ezetimibe/Rosuvastatin Fixed Dose Combination (10 mg/10 mg) Versus the Individual Formulations Taken Concomitantly | ADVANCES IN THERAPY | 10.1007/s12325-023-02439-8 | E3 No eligible comparator |
| 1434 | Niu, SP; Li, Y; Dong, WL; Xia, L; Shen, TT; Wang, JX | A Randomized Study on the Bioequivalence of Desloratadine in Healthy Chinese Subjects and the Association of Different Metabolic Phenotypes with UGT2B | CURRENT DRUG METABOLISM | 10.2174/1389200221999201027143903 | E3 No eligible comparator |
| 1439 | Huang, J; Cai, HL; Li, WQ; Huang, XM; Guan, HY; Wang, X | A UPLC-MS/MS method for simultaneous quantification of sildenafil and N-desmethyl sildenafil applied in pharmacokinetic and bioequivalence studies in | INTERNATIONAL JOURNAL OF CLINICAL PHARMACOLOGY AND THERAPEUT | 10.5414/CP203838 | E3 No eligible comparator |
| 1459 | Tang, L; Wang, KL; Peng, C; Li, XW; Li, L; Wang, Y | A simplified and sensitive LC-APCI-MS/MS method for the quantification of propofol: Application to a bioequivalence study in healthy Chinese subjects | INTERNATIONAL JOURNAL OF CLINICAL PHARMACOLOGY AND THERAPEUT | 10.5414/CP203821 | E3 No eligible comparator |
| 1473 | Liu, C; Cleton, A; Sui, YB; Liu, YW; Li, JY; Yuan, F | Bioequivalence Evaluation Between Acarbose and Metformin Fixed-Dose Combination and Corresponding Individual Components in Healthy Chinese Male and Fe | CLINICAL PHARMACOLOGY IN DRUG DEVELOPMENT | 10.1002/cpdd.994 | E3 No eligible comparator |
| 1477 | Lei, YY; Yi, Q; Ji, S; Lu, JL; Yang, ZY | Bioequivalence Evaluation of Two Metformin/Empagliflozin Fixed-Dose Combination Tablets in Healthy Chinese Subjects Under Fasting and Fed Conditions: | CLINICAL PHARMACOLOGY IN DRUG DEVELOPMENT | 10.1002/cpdd.70029 | E3 No eligible comparator |
| 5 | 刘彩 | BG医药公司T产品营销策略优化研究 |  | 10.27661/d.cnki.gzhnu.2025.005191 | E4 No clinical, safety or pharmacokinetic outcome |
| 7 | 张丹 | J市L区药品安全协同治理问题研究 |  | 10.27793/d.cnki.gsdsw.2022.000019 | E4 No clinical, safety or pharmacokinetic outcome |
| 8 | 陈思蒙 | L公司GLP-1类药物中国市场营销策略研究 |  | 10.27272/d.cnki.gshdu.2025.005165 | E4 No clinical, safety or pharmacokinetic outcome |
| 9 | 罗文岚 | Q药业公司中成药营销策略优化研究 |  | 10.27175/d.cnki.gjxcu.2025.002070 | E4 No clinical, safety or pharmacokinetic outcome |
| 10 | 魏祎 | R公立医院药品采购业务内部控制案例研究 |  | 10.26975/d.cnki.gccks.2022.000046 | E4 No clinical, safety or pharmacokinetic outcome |
| 27 | 彭菲; 靳秋霞; 许西西; 刘洁琼; 郭胜才 | 三批国家集采药品121份说明书中特殊人群用药标示情况分析 | 中医药管理杂志 | 10.16690/j.cnki.1007-9203.2021.21.042 | E4 No clinical, safety or pharmacokinetic outcome |
| 28 | 李平; 谢少玲; 叶飞强; 张镇彬 | 两个集采碳青霉烯类药品治疗肺炎的真实世界疗效比较 | 中国处方药 | 10.3969/j.issn.1671-945X.2025.17.002 | E4 No clinical, safety or pharmacokinetic outcome |
| 30 | 徐勍 | 中药注射剂价格畸高与竞争政策 |  | 10.27006/d.cnki.gdbcu.2019.000095 | E4 No clinical, safety or pharmacokinetic outcome |
| 33 | 梁盟盟; 李文; 胡顺 | 临床药师在推进国家组织药品集中采购政策中的作用与意义 | 临床医学研究与实践 | 10.19347/j.cnki.2096-1413.202312052 | E4 No clinical, safety or pharmacokinetic outcome |
| 34 | 赵振基 | 丽珠医药：新模式赋能大零售 | 中国药店 |  | E4 No clinical, safety or pharmacokinetic outcome |
| 42 | 唐甘翎; 谢祥威; 罗鲜丹; 陆艳丽; 蒋慧娴; 李静怡 | 伏立康唑原研药与仿制药的有效性、安全性及经济性评价 | 中国药房 | 10.6039/j.issn.1001-0408.2024.07.11 | E4 No clinical, safety or pharmacokinetic outcome |
| 50 | 赵新喜 | 创新方式重拳出击不断提升基金监管有效性 | 中国医疗保险 |  | E4 No clinical, safety or pharmacokinetic outcome |
| 55 | 陈勇隽 | 医保药品支付价格形成机制研究 |  | 10.27307/d.cnki.gsjtu.2018.004305 | E4 No clinical, safety or pharmacokinetic outcome |
| 60 | 王之舟; 董宪喆; 白向荣; 王可; 冯英楠; 庄伟 | 口服抗菌药物集中带量采购实施情况问卷调查分析 | 中国医院药学杂志 | 10.13286/j.1001-5213.2023.13.16 | E4 No clinical, safety or pharmacokinetic outcome |
| 62 | 操德智; 丁鑫; 陈鸿; 曹雪艳; 王涵; 李燕梅 | 国产左乙拉西坦临床使用情况调研分析 | 第九届CAAE国际癫痫论坛 | 10.26914/c.cnkihy.2021.077390 | E4 No clinical, safety or pharmacokinetic outcome |
| 68 | 申芳丽; 刘璐; 宋沧桑; 李兴德; 张阳 | 国家药品集采实施效果与评价——以某三甲医院为例 | 中国医疗保险 | 10.19546/j.issn.1674-3830.2021.8.009 | E4 No clinical, safety or pharmacokinetic outcome |
| 80 | 王钰; 韩玉玲; 王忠兰; 郝丽娜 | 国家集采药品孟鲁司特钠治疗儿童哮喘的临床综合评价 | 中国医院药学杂志 | 10.13286/j.1001-5213.2024.10.01 | E4 No clinical, safety or pharmacokinetic outcome |
| 85 | 胡惠子; 文小桐; 杨莹; 毛李宁; 王兆仑; 罗佳 | 基于内分泌科医师问卷调查结果评价武汉市胰岛素类药品集中采购分组办法的合理性及可行性 | 中国药房 | 10.6039/j.issn.1001-0408.2022.06.04 | E4 No clinical, safety or pharmacokinetic outcome |
| 90 | 宋沧桑 | 基于多中心真实世界药品临床综合评价体系建设及推广应用 |  |  | E4 No clinical, safety or pharmacokinetic outcome |
| 93 | 史锦仕; 陈雯; 江滨; 易湛苗 | 基于真实世界数据的11个试点城市阿托伐他汀与瑞舒伐他汀国家集采政策效果评价——间断时间序列分析 | 中国医院药学杂志 | 10.13286/j.1001-5213.2026.02.06 | E4 No clinical, safety or pharmacokinetic outcome |
| 95 | 雷雨田 | 基于真实世界数据的集采与原研草酸艾司西酞普兰的临床综合评价 |  | 10.27202/d.cnki.gkmyc.2024.001536 | E4 No clinical, safety or pharmacokinetic outcome |
| 105 | 何红锋 | 如何让抗癌药降价落到实处 | 中国政府采购 |  | E4 No clinical, safety or pharmacokinetic outcome |
| 106 | 王焱琪; 郭朗; 孙利华 | 对“4+7”药品用量落实问题的探讨 | 中国医疗保险 | 10.19546/j.issn.1674-3830.2019.8.009 | E4 No clinical, safety or pharmacokinetic outcome |
| 108 | 杨黎; 董宪喆; 崔晓辉; 鲁松; 刘文娜; 李林 | 带量采购中标药品左乙拉西坦片的临床应用评价 | 中国医药导报 | 10.20047/j.issn1673-7210.2021.22.009 | E4 No clinical, safety or pharmacokinetic outcome |
| 109 | 李杨华 | 带量采购中选ARB类降压药在老年患者中的临床疗效观察与药物经济学评价 |  |  | E4 No clinical, safety or pharmacokinetic outcome |
| 113 | 位宁; 冯雪; 陈夏; 柴清军 | 带量采购药品阿托伐他汀钙片与原研药品的效益/风险对比分析 | 中国合理用药探索 | 10.3969/j.issn.2096-3327.2023.02.009 | E4 No clinical, safety or pharmacokinetic outcome |
| 117 | 陈举亮; 张慧; 林慧芸; 胡紫微 | 广东联盟中成药集采品种质量评价标准研究 | 中国药房 | 10.6039/j.issn.1001-0408.2023.17.02 | E4 No clinical, safety or pharmacokinetic outcome |
| 118 | 曾铮; 巫柳岑; 黄振光; 何文莉; 宓郑成; 谢祥威 | 广西医疗机构落实国家药品集中带量采购政策的效果分析 | 中国药房 | 10.6039/j.issn.1001-0408.2024.05.02 | E4 No clinical, safety or pharmacokinetic outcome |
| 120 | 杨斐頔 | 我国仿制药一致性评价的政策过程研究 |  | 10.27414/d.cnki.gxnju.2022.000295 | E4 No clinical, safety or pharmacokinetic outcome |
| 121 | 陆信 | 我国儿童抗菌药物应用的可及性与合理性现状分析 |  | 10.27253/d.cnki.gnjzu.2025.000089 | E4 No clinical, safety or pharmacokinetic outcome |
| 122 | 刁一凡 | 我国新型抗肿瘤药物公共保障政策与药物可及性评估研究 |  | 10.27648/d.cnki.gzxhu.2020.000907 | E4 No clinical, safety or pharmacokinetic outcome |
| 123 | 马枋婷; 常峰; 路云; 毛锌; 刘明珠 | 我国药品集中带量采购政策执行情况分析 | 卫生经济研究 | 10.14055/j.cnki.33-1056/f.2023.07.017 | E4 No clinical, safety or pharmacokinetic outcome |
| 124 | 赵博雅 | 我国药品集中带量采购政策的实施效果评价研究 |  | 10.27356/d.cnki.gtjdu.2023.001581 | E4 No clinical, safety or pharmacokinetic outcome |
| 127 | 曹歌; 李盼; 王玉民; 刘艳春; 郭振茹 | 我院第一批带量采购降压药品用药情况分析 | 中国社区医师 | 10.3969/j.issn.1007-614x.2021.03.006 | E4 No clinical, safety or pharmacokinetic outcome |
| 129 | 刘胜男; 冀召帅; 毛乾泰 | 抗菌药品国家集采政策实施四年效果与评价——以北京某三级医院为例 | 海峡药学 | 10.3969/j.issn.1006-3765.2024.07.028 | E4 No clinical, safety or pharmacokinetic outcome |
| 135 | 朱嘉; 翁志洁; 阮秀芳; 张清 | 探讨新修订的《药品管理法》对药品监管工作的挑战和应对策略 | 中国药事 | 10.16153/j.1002-7777.2019.12.001 | E4 No clinical, safety or pharmacokinetic outcome |
| 146 | 金晓琴; 朱星宇; 沈建飞; 马文祥 | 某院仿制与原研降压药物差异性及仿制降压药换用情况分析 | 中国处方药 |  | E4 No clinical, safety or pharmacokinetic outcome |
| 151 | 孙佳瑜; 廖昔威; 卢泽容; 林玉燕; 关水权; 陈蕾 | 深圳市福田区医疗机构药品集中采购使用现状调研分析 | 海峡药学 | 10.3969/j.issn.1006-3765.2025.10.025 | E4 No clinical, safety or pharmacokinetic outcome |
| 152 | 周浩 | 湖南省抗菌药物专项集中采购政策实施效果评估研究 |  | 10.27661/d.cnki.gzhnu.2024.003990 | E4 No clinical, safety or pharmacokinetic outcome |
| 162 | 邵靖; 蒋茜; 谢晓曦; 路云; 常峰 | 综合评分法在药品集中带量采购中的应用及优化研究 | 现代商贸工业 | 10.19311/j.cnki.1672-3198.2023.22.010 | E4 No clinical, safety or pharmacokinetic outcome |
| 171 | 宁艳阳 | 药品降价保供:国家打出一记重拳 | 中国卫生 | 10.15973/j.cnki.cn11-3708/d.2019.03.027 | E4 No clinical, safety or pharmacokinetic outcome |
| 181 | 黄勇 | 重庆市社区药学服务评价体系的研究与构建 |  | 10.27674/d.cnki.gcyku.2023.000293 | E4 No clinical, safety or pharmacokinetic outcome |
| 188 | 董慧秋 | 集中带量采购常态化背景下仿制药质量安全应急管理能力评价与仿真研究 |  | 10.27253/d.cnki.gnjzu.2024.000219 | E4 No clinical, safety or pharmacokinetic outcome |
| 193 | 刘春芳; 章孝全 | 集中带量采购瑞舒伐他汀钙片对急性脑梗死合并高脂血症患者疗效、安全性和经济性分析 | 医学理论与实践 | 10.19381/j.issn.1001-7585.2022.08.059 | E4 No clinical, safety or pharmacokinetic outcome |
| 205 | 陈路路 | 集采背景下化学仿制药研发项目立项评价研究 |  | 10.27466/d.cnki.gzzdu.2025.002154 | E4 No clinical, safety or pharmacokinetic outcome |
| 222 | 周洋; 张泽华; 彭文星; 张芸楠; 韩嘉伦; 石秀锦 | 原研和仿制替格瑞洛在经皮冠状动脉介入术后应用的相关对比研究 | 中国医药 | 10.3760/j.issn.1673-4777.2023.10.001 | E4 No clinical, safety or pharmacokinetic outcome |
| 252 | 张素珍 | 苯磺酸氨氯地平片、尼莫地平片和氯氮平片的质量和疗效一致性评价 |  |  | E4 No clinical, safety or pharmacokinetic outcome |
| 258 | 潘梅 | 阿莫西林胶囊 |  |  | E4 No clinical, safety or pharmacokinetic outcome |
| 259 | 李玉娟 | 阿莫西林胶囊0.25g |  |  | E4 No clinical, safety or pharmacokinetic outcome |
| 260 | 李玉娟 | 阿莫西林胶囊0.5g |  |  | E4 No clinical, safety or pharmacokinetic outcome |
| 261 | 赵业辉 | 阿莫西林胶囊一致性评价 |  |  | E4 No clinical, safety or pharmacokinetic outcome |
| 266 | Chen B; Chen LY; Chen P; Wang C; Yang CW; Hu Y | A retrospective analysis of outpatient use of small-molecule targeted inhibitors for lymphoma across six regions of China (2016-2022). | Frontiers in pharmacology | 10.3389/fphar.2026.1784805 | E4 No clinical, safety or pharmacokinetic outcome |
| 267 | Chen B; Wang C; Chen LY; Hu Y; Wang YZ; Li LC | A retrospective analysis of small molecule targeting inhibitor usage for lung cancer among outpatients in six major regions of China (2016-2022). | Frontiers in pharmacology | 10.3389/fphar.2025.1700389 | E4 No clinical, safety or pharmacokinetic outcome |
| 268 | Lu W; Qiao Y; Chen H; Li W; Wang M; Yuan C | A study on the availability of national centralized drug procurement in regions with different levels of economic development: an investigation and an | Frontiers in pharmacology | 10.3389/fphar.2026.1652715 | E4 No clinical, safety or pharmacokinetic outcome |
| 269 | Li S; Sun L | Analysis of the problems of drug sampling and testing programme under tripartite system reform in China. | Journal of evaluation in clinical practice | 10.1111/jep.13725 | E4 No clinical, safety or pharmacokinetic outcome |
| 271 | Chang Q; Tian Y; Gao L; Xia N | Challenges and countermeasures for China's centralised volume-based procurement policy in healthcare. | The International journal of health planning and management | 10.1002/hpm.3803 | E4 No clinical, safety or pharmacokinetic outcome |
| 272 | Wu J; Qiu L; Li J; Zhou Q; Xie W; Shen Y | Choice intention for the national volume-based procurement drug and its associated factors: a cross-sectional study on patients with late-life depress | BMC psychiatry | 10.1186/s12888-024-06026-1 | E4 No clinical, safety or pharmacokinetic outcome |
| 273 | Luo X; Du X; Lv X; Yang Y; Zhang X; Huang L | Clinical Benefit, Price, and Regulatory Approval of Cancer Drugs Granted Breakthrough Therapy Designation in China, 2020-2024. | JAMA network open | 10.1001/jamanetworkopen.2024.39080 | E4 No clinical, safety or pharmacokinetic outcome |
| 276 | Li M; Cao Y; Huang H; Qin G; Chu M; Zou M | Cost-effectiveness analysis of antiretroviral drugs for treatment-naive HIV infection in China. | BMC public health | 10.1186/s12889-023-17052-1 | E4 No clinical, safety or pharmacokinetic outcome |
| 280 | Yu X; Lin H; Li B; Fu H; Zhang Y | Effects of national volume-based procurement policy on the usage and expenditure of platinum antineoplastic drugs in China: an interrupted time series | Frontiers in pharmacology | 10.3389/fphar.2025.1593099 | E4 No clinical, safety or pharmacokinetic outcome |
| 283 | Gao L; Mao R; Xiong X; Xiong W; An X; Wen L | Governance absorption of volume-based procurement: study of dual-track implementation in a public hospital. | Frontiers in public health | 10.3389/fpubh.2026.1834133 | E4 No clinical, safety or pharmacokinetic outcome |
| 284 | Huang A; Wang X; Tao Y; Lin L; Cheng H | Healthcare professionals' knowledge, attitude and practice towards National Centralized Drug Procurement policy in central China: A cross-sectional st | Frontiers in pharmacology | 10.3389/fphar.2022.996824 | E4 No clinical, safety or pharmacokinetic outcome |
| 285 | Shen Q; Kang L; Ye D; Li Y; Zhang K | Healthcare professionals' knowledge, attitudes, and practices regarding China's National Centralized Drug Procurement policy: a cross-sectional study | Frontiers in public health | 10.3389/fpubh.2025.1616534 | E4 No clinical, safety or pharmacokinetic outcome |
| 287 | Wang Z; Wang K; Hua Y; Dong X; Zhang L | Impact and implications of national centralized drug procurement in China. | International journal of clinical pharmacy | 10.1007/s11096-024-01767-1 | E4 No clinical, safety or pharmacokinetic outcome |
| 288 | Wang M; Liu J; Bellows BK; Qi Y; Sun J; Liu J | Impact of China's Low Centralized Medicine Procurement Prices on the Cost-Effectiveness of Statins for the Primary Prevention of Atherosclerotic Cardi | Global heart | 10.5334/gh.830 | E4 No clinical, safety or pharmacokinetic outcome |
| 289 | Huang Y; Ren Y; Zhang Y; Jia Y; Li Q; Yao M | Impact of China's National Volume-Based Drug Procurement: A Multilevel Interrupted Time Series Analysis on Medical Expenditures in Hypertensive Patien | International journal of health policy and management | 10.34172/ijhpm.8540 | E4 No clinical, safety or pharmacokinetic outcome |
| 290 | Zhao B; Wu J | Impact of China's National Volume-Based Procurement on Drug Procurement Price, Volume, and Expenditure: An Interrupted Time Series Analysis in Tianjin | International journal of health policy and management | 10.34172/ijhpm.2023.7724 | E4 No clinical, safety or pharmacokinetic outcome |
| 294 | Yi H; Tan S; Cai M; Kuai L; Xu D; Han S | Impacts of national volume-based procurement policy on insulin price, volume and expenditure in China: a quasi-experimental study. | BMJ public health | 10.1136/bmjph-2025-004332 | E4 No clinical, safety or pharmacokinetic outcome |
| 298 | Lin F; Li H; Zhu K; Wu Y; Fan Q; Ding J | Mechanisms influencing generic drug substitution behavior under the national centralized drug procurement policy. | Journal of pharmaceutical policy and practice | 10.1080/20523211.2025.2547679 | E4 No clinical, safety or pharmacokinetic outcome |
| 299 | Li X; Tao R; Jin Y; Li N | National centralized drug procurement and health care expenditure of households-micro-evidence from CFPS. | Frontiers in public health | 10.3389/fpubh.2024.1405197 | E4 No clinical, safety or pharmacokinetic outcome |
| 304 | Xue A; Xue Q; Fu J; Fan K; Zhang J; Cai P | Quantifying the impacts of volume-based procurement policy on spatial accessibility of antidepressants via generic substitution: A four-city cohort st | PloS one | 10.1371/journal.pone.0318509 | E4 No clinical, safety or pharmacokinetic outcome |
| 306 | Wang Q; Ling X; Dai Z; Wang X; Guo W; Chen G | Reform progress and achievements of China's incentive policies for pediatric medicine over the last decade. | Frontiers in pharmacology | 10.3389/fphar.2025.1561095 | E4 No clinical, safety or pharmacokinetic outcome |
| 308 | Gu Y; Zhuang Q | Research on the impact of China's reform to delegate power, streamline administration, and optimize government services on the technology innovation e | Frontiers in public health | 10.3389/fpubh.2024.1325298 | E4 No clinical, safety or pharmacokinetic outcome |
| 313 | Wang Q; Liu S; Nie Z; Zhu Z; Fu Y; Zhang J | The pan-Canadian Tiered Pricing Framework and Chinese National Volume-Based Procurement: A comparative study using Donabedian's structure-process-outc | Journal of global health | 10.7189/jogh.13.04137 | E4 No clinical, safety or pharmacokinetic outcome |
| 314 | Fu H; Lai Y; Li Y; Zhu Y; Yip W | Understanding medical corruption in China: a mixed-methods study. | Health policy and planning | 10.1093/heapol/czad015 | E4 No clinical, safety or pharmacokinetic outcome |
| 341 | Qu J; Zuo W; Took RL; Schafermeyer KW; Lukas S; Wang S | A nationwide survey exploring physicians' and pharmacists' knowledge, awareness and perceptions regarding generic medicines in China. | BMC health services research | 10.1186/s12913-022-08438-9 | E4 No clinical, safety or pharmacokinetic outcome |
| 414 | Lee VW; Cheng FW; Fong FY; Ng EE; Lo LL; Ngai LY | Branded versus generic drug use in chronic disease management in Hong Kong-perspectives of health care professionals and the general public. | Hong Kong medical journal = Xianggang yi xue za zhi | 10.12809/hkmj177087 | E4 No clinical, safety or pharmacokinetic outcome |
| 433 | Gani L; Lau E; Luk A; Sobrepena L; Tran QK; Kesavadev J | Cross-sectional survey of biosimilar insulin utilization in Asia: The Joint Asia Diabetes Evaluation Program. | Journal of diabetes investigation | 10.1111/jdi.12843 | E4 No clinical, safety or pharmacokinetic outcome |
| 436 | Li X; Xu J | Does China's national volume-based drug procurement policy promote or hinder pharmaceutical innovation? | Frontiers in pharmacology | 10.3389/fphar.2024.1392239 | E4 No clinical, safety or pharmacokinetic outcome |
| 446 | Han X; Ma W; Wang B; Gu X; Wang G; Lin L | Efficacy and Safety of a Generic Clevidipine in Hypertensive Urgencies and Non-Fulminant Hypertensive Emergencies: A Phase III, Multicenter, Randomize | Journal of clinical hypertension (Greenwich, Conn.) | 10.1111/jch.70144 | E4 No clinical, safety or pharmacokinetic outcome |
| 467 | Huo BN; Ai ML; Jia YT; Liu Y; Wang Y; Yin NG | General characteristics and reasons for the discontinuation of drug clinical trials in mainland China. | BMC medical research methodology | 10.1186/s12874-021-01443-2 | E4 No clinical, safety or pharmacokinetic outcome |
| 469 | Liu Q; Huang Z; Mao Z | Has the consistency evaluation policy of generic drugs promoted the innovation quality of Chinese pharmaceutical manufacturing industry? An empirical | Frontiers in public health | 10.3389/fpubh.2023.1265756 | E4 No clinical, safety or pharmacokinetic outcome |
| 472 | Wang Z; Wang R; Li X; Bai L; Fan P; Tang Y | Influencing Factors of Generic Prescribing Behavior of Physicians: A Structural Equation Model Based on the Theory of Planned Behavior. | Risk management and healthcare policy | 10.2147/RMHP.S446743 | E4 No clinical, safety or pharmacokinetic outcome |
| 475 | Qu J; Zuo W; Wang S; Du L; Liu X; Gao Y | Knowledge, perceptions and practices of pharmacists regarding generic substitution in China: a cross-sectional study. | BMJ open | 10.1136/bmjopen-2021-051277 | E4 No clinical, safety or pharmacokinetic outcome |
| 580 | Shen M; Liang X; Wu H | The impact of bioequivalence regulation on pharmaceutical firm outcomes: Evidence from China. | Social science & medicine (1982) | 10.1016/j.socscimed.2025.118242 | E4 No clinical, safety or pharmacokinetic outcome |
| 585 | Lu S; Cai J; Xu F | Ultra-low price of generic agents in China may weaken patients' drug recognition and compliance. | Drug discoveries & therapeutics | 10.5582/ddt.2020.01011 | E4 No clinical, safety or pharmacokinetic outcome |
| 588 | Ding C; Qiu Y; Li W; Yuan F; Gong W; Chen Z | [Expert consistency evaluation on nutrition and health knowledge questionnaire items for Chinese adults]. | Wei sheng yan jiu = Journal of hygiene research | 10.19813/j.cnki.weishengyanjiu.2022.06.002 | E4 No clinical, safety or pharmacokinetic outcome |
| 589 | Huang GQ; Zhang L; Zhou JH; Xu CY; Zheng L; Wang AM | [Rationality of formula composition of ammonium glycyrrhizinate in Yanlishuang Oral Dripping Pills based on pharmacodynamics and pharmacokinetics]. | Zhongguo Zhong yao za zhi = Zhongguo zhongyao zazhi = China | 10.19540/j.cnki.cjcmm.20250822.201 | E4 No clinical, safety or pharmacokinetic outcome |
| 593 |  | 国家医保局:国家药品集采协议期满后要平稳接续 | 中国数字医学 |  | E4 No clinical, safety or pharmacokinetic outcome |
| 601 | 胡琴; 肖晓彤; 李春辉; 孟秀娟; 刘韶; 邓晟 | 抗菌药物集采前后美罗培南临床应用及处方合理性评价 | 中华医院感染学杂志 |  | E4 No clinical, safety or pharmacokinetic outcome |
| 605 | 丁妍之; 云意 | 药品集中带量采购背景下临床药师参与胸心外科围术期抗菌药物管理的效果评价 | 临床合理用药 |  | E4 No clinical, safety or pharmacokinetic outcome |
| 613 | 黄晓威; 陈婷婷; 傅新阳; 洪珊珊; 林志强 | 2017-2019年泉州市第一医院口服抗高血压药的应用情况与趋势分析 | 2020年泉州市药学会学术年会 |  | E4 No clinical, safety or pharmacokinetic outcome |
| 614 | 段自皞; 李晶; 朱枝祥; 沈炳香 | 2019—2021年六安市人民医院质子泵抑制剂使用情况分析 | 现代药物与临床 | 10.7501/j.issn.1674-5515.2022.06.033 | E4 No clinical, safety or pharmacokinetic outcome |
| 617 | 陈丽娜 | 39种国家谈判药品的使用,5个省份陆续表态 | 医师在线 | 10.3969/j.issn.2095-7165.2018.23.010 | E4 No clinical, safety or pharmacokinetic outcome |
| 619 | 刘清扬; 李建涛; 周羽威; 唐彦; 后子靖; 屈静晗 | 4种国家组织集中带量采购抗高血压药仿制品种替换对患者处方依从性的影响研究 | 中国药学杂志 | 10.11669/cpj.2023.18.014 | E4 No clinical, safety or pharmacokinetic outcome |
| 621 | 于佳; 李玲; 杨秋洁; 张石宇; 李广松 | AP联合PD-1/PD-L1抑制剂治疗非小细胞肺癌的药物经济学评价 | 中国药物经济学 | 10.12010/j.issn.1673-5846.2025.10.004 | E4 No clinical, safety or pharmacokinetic outcome |
| 622 | 赵洪鹤 | A公司T药品市场营销策略优化研究 |  |  | E4 No clinical, safety or pharmacokinetic outcome |
| 624 | 石丹丹 | A公司抗癌类药物的营销策略研究——以肺癌I和T产品为例 |  |  | E4 No clinical, safety or pharmacokinetic outcome |
| 626 | 谢正武 | A医药公司营销人员薪酬方案优化研究 |  |  | E4 No clinical, safety or pharmacokinetic outcome |
| 630 | 李光杰 | BG钢铁贸易公司营销策略优化研究 |  |  | E4 No clinical, safety or pharmacokinetic outcome |
| 633 | 顾旦淳 | B公司抗肿瘤药营销策略优化研究 |  |  | E4 No clinical, safety or pharmacokinetic outcome |
| 637 | 上官瑞燕 | CMT公司FTB仿制药研发项目风险管理研究 |  |  | E4 No clinical, safety or pharmacokinetic outcome |
| 640 | 林坤河; 上官业声; 饶娅琦; 彭静; 陈翼; 姚轶凡 | DIP支付方式下医保供给侧政策协同对费用控制的影响研究 | 中国卫生政策研究 | 10.3969/j.issn.1674-2982.2024.05.003 | E4 No clinical, safety or pharmacokinetic outcome |
| 642 | 代雪飞; 杜娟 | DRGs环境下2型糖尿病临床药学路径制定及应用成效分析 | 中国医院药学杂志 | 10.13286/j.1001-5213.2022.22.18 | E4 No clinical, safety or pharmacokinetic outcome |
| 644 | 傅杨杨; 朱怀军; 卞晓洁; 高延庆; 张庆红; 陶亮 | DRG支付下胃癌手术患者住院费用医保结余影响因素分析 | 中华医院管理杂志 | 10.3760/cma.j.cn111325-20221116-00995 | E4 No clinical, safety or pharmacokinetic outcome |
| 647 | 吴梦月 | DTP药房的冷链配送 | 中国药店 |  | E4 No clinical, safety or pharmacokinetic outcome |
| 648 | 张香香 | D公司降糖药品山东市场营销策略研究 |  |  | E4 No clinical, safety or pharmacokinetic outcome |
| 655 | 周一静 | GT生物医药项目的商业计划书 |  |  | E4 No clinical, safety or pharmacokinetic outcome |
| 656 | 庞海参 | G公司A口服溶液仿制药研发项目风险管理研究 |  |  | E4 No clinical, safety or pharmacokinetic outcome |
| 657 | 孙佳 | G公司抗癫痫L药营销策略优化研究 |  |  | E4 No clinical, safety or pharmacokinetic outcome |
| 672 | 宋国强 | M制药公司抗肿瘤仿制药竞争战略研究 |  |  | E4 No clinical, safety or pharmacokinetic outcome |
| 674 | 马振华 | M集团江苏制药分公司精益六西格玛管理改进研究 |  |  | E4 No clinical, safety or pharmacokinetic outcome |
| 676 | Rupam Nehta; N. C. Sharma; Pramod Verma; Nitin Sharma | Optimization of Yield and Fruit Quality Attributes of ‘Delicious’ Apple (Malus × domestica Borkh) Through GA4+7 + BA Sprays | Applied Fruit Science | 10.1007/s10341-025-01558-6 | E4 No clinical, safety or pharmacokinetic outcome |
| 678 | 宋树杰; 许桂媛; 马先杰; 李祥鹏; 刘建芳 | Power BI在我院药品数据可视化中的应用 | 海峡药学 | 10.3969/j.issn.1006-3765.2024.08.033 | E4 No clinical, safety or pharmacokinetic outcome |
| 681 | 张翠玲 | S制药公司质量管理持续改进研究 |  |  | E4 No clinical, safety or pharmacokinetic outcome |
| 683 | 李鑫鑫 | S生物样本检测中心发展战略研究 |  |  | E4 No clinical, safety or pharmacokinetic outcome |
| 687 | 乔世龙 | XH制药公司财务风险预警及防范研究 |  |  | E4 No clinical, safety or pharmacokinetic outcome |
| 690 | 江霞 | Y公司处方类仿制药竞争战略 |  |  | E4 No clinical, safety or pharmacokinetic outcome |
| 691 | 邢鹏英 | Y药业集团处方药促销策略研究 |  |  | E4 No clinical, safety or pharmacokinetic outcome |
| 692 | 蒋亚茹 | Z公司连续股权激励动因及经济后果研究 |  |  | E4 No clinical, safety or pharmacokinetic outcome |
| 693 | 李俊珂 | Z医院流感药品需求预测及库存优化研究 |  |  | E4 No clinical, safety or pharmacokinetic outcome |
| 694 | 李俊颖 | Z银行软硬件项目集中采购管理改进对策研究 |  |  | E4 No clinical, safety or pharmacokinetic outcome |
| 696 | 邹国盛; 赵家强; 梅清华; 叶浩婷; 周辉; 朱新昌 | “4+7城市”药品集中采购实施后广东省第二人民医院原研药与仿制药使用情况分析 | 中国医院用药评价与分析 | 10.14009/j.issn.1672-2124.2020.07.022 | E4 No clinical, safety or pharmacokinetic outcome |
| 698 | 员东智 | “带量采购”政策对医药上市公司股价波动的影响探究 |  |  | E4 No clinical, safety or pharmacokinetic outcome |
| 699 | 杜赵雨 | “带量采购”背景下，中国上市医药制造业企业的竞争战略选择及绩效影响--以普洛药业和普利制药为例 |  |  | E4 No clinical, safety or pharmacokinetic outcome |
| 700 | 刘俊 | ”4+7”前后抗乙型肝炎病毒药物应用情况分析 | 中国城乡企业卫生 | 10.16286/j.1003-5052.2021.11.029 | E4 No clinical, safety or pharmacokinetic outcome |
| 701 | 战旗; 葛继芸; 黄玉凤 | ”4+7”带量采购政策下的恩替卡韦应用情况调查 | 药学服务与研究 | 10.5428/pcar20210216 | E4 No clinical, safety or pharmacokinetic outcome |
| 702 | 郭晋芸; 李霞; 郭晋纲; 贾丽红 | ”4+7”药品对我院门诊药房退药情况的调查分析 | 中国药物与临床 | 10.11655/zgywylc2021.18.049 | E4 No clinical, safety or pharmacokinetic outcome |
| 703 | 林凤梅 | ”4+7”药品带量采购政策对某三甲医院慢性病用药的影响 | 甘肃医药 |  | E4 No clinical, safety or pharmacokinetic outcome |
| 704 | 张英楠; 王常珺; 周蜜; 邱书琳; 胡雅洁 | ”十三五”至”十四五”期间医疗费用结构变迁与政策效应分析——基于某三甲医院乳腺癌患者数据的实证研究 | 中国卫生事业管理 | 10.3969/j.issn.1004-4663.2025.12.008 | E4 No clinical, safety or pharmacokinetic outcome |
| 705 | 林莉莉; 梁淑婷; 陈翠仪 | ”集采”背景下城镇居民使用紫杉醇(白蛋白结合)的住院诊疗费用变化研究 | 养生科学 | 10.3969/j.issn.2095-6673.2025.04.079 | E4 No clinical, safety or pharmacokinetic outcome |
| 709 | 王淼; 王旭涛; 李玉莹; 苏红 | 一致性评价对中国仿制药行业的影响 | 中国医药工业杂志 | 10.16522/j.cnki.cjph.2024.11.017 | E4 No clinical, safety or pharmacokinetic outcome |
| 712 | 杨琦; 顾洪安 | 上海市社区卫生服务中心国家药品集中采购实施情况调查 | 上海医药 | 10.3969/j.issn.1006-1533.2020.04.004 | E4 No clinical, safety or pharmacokinetic outcome |
| 714 | 汪皖青; 郑爽; 侯欢; 姝丽雅; 陈蓉 | 不同地区两家三甲医院医生和患者国家药品集中采购政策认知现状及药学需求调查研究 | 医药导报 | 10.3870/j.issn.1004-0781.2022.10.034 | E4 No clinical, safety or pharmacokinetic outcome |
| 715 | 姝丽雅 | 不同地区两家三甲医院国家药品集中采购政策医、患认知现状及药学服务需求调研与分析 |  |  | E4 No clinical, safety or pharmacokinetic outcome |
| 724 | 郑亮亮 | 中国医药产品数字化营销研究——以葛兰素史克(GSK)公司舒利迭(Seretide)品牌为例 |  |  | E4 No clinical, safety or pharmacokinetic outcome |
| 725 | 冉薇 | 中国医药行业上市公司的财务风险预警研究——以T公司为例 |  |  | E4 No clinical, safety or pharmacokinetic outcome |
| 728 | 熊尧 | 中国新医改背景下药品价格规制政策效果的实证评估 |  |  | E4 No clinical, safety or pharmacokinetic outcome |
| 732 | 乔瑞娟 | 中药处方点评的标准化与临床实践中的应用 | 关爱生命大讲堂——生命关怀与智慧康养系列学术研讨会 |  | E4 No clinical, safety or pharmacokinetic outcome |
| 734 | 侯江涛; 范超; 谢晓敏; 李祥鹏 | 临床用药辅助决策系统集成”药视通”完善集中采购药品与原研药品并存的精细管理 | 医药导报 | 10.3870/j.issn.1004-0781.2026.05.026 | E4 No clinical, safety or pharmacokinetic outcome |
| 742 | 焦海东; 张恒杰; 东航; 高倩 | 从赋能到使能:集采平台全产业链管理模式研究 | 工程造价管理 | 10.19730/j.cnki.1008-2166.2024-06-023 | E4 No clinical, safety or pharmacokinetic outcome |
| 743 | 林丽敏 | 以医疗机构与个体患者视角评估国家药品集中采购政策的实施效果 |  |  | E4 No clinical, safety or pharmacokinetic outcome |
| 744 | 王敏娇; 方敏; 严运楼 | 价值医疗视域下”三医协同”创新路径研究 | 卫生经济研究 | 10.3969/j.issn.1004-7778.2025.11.003 | E4 No clinical, safety or pharmacokinetic outcome |
| 745 | 赵洁; 李巍; 王皋俊 | 价值医疗视角下国家药品集中带量采购在某公立医院的实施效果评价 | 中国药房 | 10.6039/j.issn.1001-0408.2021.19.18 | E4 No clinical, safety or pharmacokinetic outcome |
| 746 | 赵雅筠 | 价值链视角下K公司营运资金管理研究 |  |  | E4 No clinical, safety or pharmacokinetic outcome |
| 747 | 王涵 | 价值链视角下医药行业轻资产盈利模式转型研究 | 商业观察 | 10.3969/j.issn.2096-0808.2026.08.011 | E4 No clinical, safety or pharmacokinetic outcome |
| 759 | 张镓小 | 仿制药政策对我医药上市企业影响的研究 |  |  | E4 No clinical, safety or pharmacokinetic outcome |
| 767 | 刘慧敏 | 依托全流程监管 精耕集采药管 | 新晋商 |  | E4 No clinical, safety or pharmacokinetic outcome |
| 768 | 梁龚山 | 借助Power BI对国家集采药品使用监控的可行性与标准化方案设计讨论 | 中国标准化 | 10.3969/j.issn.1002-5944.2026.12.047 | E4 No clinical, safety or pharmacokinetic outcome |
| 771 | 司晓菲; 韩云川; 李武超; 陈思; 朱月; 姜典卓 | 全国药品集中采购中选口服调释制剂的药学研究分析 | 中国药事 | 10.16153/j.1002-7777.2022.03.006 | E4 No clinical, safety or pharmacokinetic outcome |
| 772 | 王馨悦; 刘锦如; 房小宇; 孙强 | 全链条视角下药品价格形成机制的国际经验及中国路径 | 中国卫生经济 |  | E4 No clinical, safety or pharmacokinetic outcome |
| 785 | 刘君阳 | 分级管理视角下山东省抗菌药物使用分析 |  |  | E4 No clinical, safety or pharmacokinetic outcome |
| 786 | 许惠溢; 马椿乔; 徐峰; 郭达群; 康惠滨; 彭康 | 利用药物经济学方法评价部分4+7集中采购药品 | 海峡药学 | 10.3969/j.issn.1006-3765.2020.10.012 | E4 No clinical, safety or pharmacokinetic outcome |
| 787 | 吕哲 | 制药企业定期报告问询函研究--以步长制药年报问询函为例 |  |  | E4 No clinical, safety or pharmacokinetic outcome |
| 791 | 张权宇; 杨玉洁; 李星; 雷海潮 | 北京医耗联动综合改革的效率与可及性评价 | 中国卫生政策研究 | 10.3969/j.issn.1674-2982.2021.01.004 | E4 No clinical, safety or pharmacokinetic outcome |
| 806 | 于婧 | 医药企业财务数字化转型的挑战与路径 | 理财 | 10.3969/j.issn.1673-1107.2025.40.003 | E4 No clinical, safety or pharmacokinetic outcome |
| 807 | 孟轶楠 | 医药企业金融资产配置的动因与绩效研究--以云南白药为例 |  |  | E4 No clinical, safety or pharmacokinetic outcome |
| 808 | 杨涛; 魏云捷 | 医药政策变动对二级市场影响的实证研究:基于集合经验模态分解 | 管理评论 |  | E4 No clinical, safety or pharmacokinetic outcome |
| 809 | 刘铭 | 医药流通行业应收账款资产证券化财务效应分析--以瑞康医药为例 |  |  | E4 No clinical, safety or pharmacokinetic outcome |
| 810 | 周李平 | 医药行业信用风险及应收账款管理方法分析 | 中国经贸 |  | E4 No clinical, safety or pharmacokinetic outcome |
| 815 | 路维琳 | 医院药品集中采购的模式分析 | 世界最新医学信息文摘（连续型电子期刊） | 10.19613/j.cnki.1671-3141.2018.29.124 | E4 No clinical, safety or pharmacokinetic outcome |
| 820 | 刁仁昌; 石斌; 卢旻; 陈晨 | 南京医保大数据平台的构建与应用 | 中国数字医学 | 10.3969/j.issn.1673-7571.2024.02.001 | E4 No clinical, safety or pharmacokinetic outcome |
| 824 | 厦门市医疗保障局 | 厦门药品集中采购和使用试点的创新与成效 | 中国医疗保险 | 10.19546/j.issn.1674-3830.2020.2.012 | E4 No clinical, safety or pharmacokinetic outcome |
| 834 | 史复; 陆阳; 刘元元; 段永刚 | 国产与原研盐酸达泊西汀片治疗早泄的临床研究 | 中国男科学杂志 | 10.3969/j.issn.1008-0848.2022.03.013 | E4 No clinical, safety or pharmacokinetic outcome |
| 842 | 曾扬 | 国家组织药品集中采购和使用在我院的应用效果分析 | 北方药学 | 10.3969/j.issn.1672-8351.2023.05.021 | E4 No clinical, safety or pharmacokinetic outcome |
| 844 | 何江江; 唐密; 丛鹂萱; 徐源; 宋捷; 陈珉惺 | 国家组织药品集中采购和使用试点对临床用药管理与使用的影响 | 中国卫生资源 |  | E4 No clinical, safety or pharmacokinetic outcome |
| 846 | 安扬; 唐婧; 毛乾泰; 艾超 | 国家组织药品集中采购对医保患者的影响及药物经济学评价 | 临床药物治疗杂志 | 10.3969/j.issn.1672-3384.2020.11.003 | E4 No clinical, safety or pharmacokinetic outcome |
| 848 | 锁驰; 罗碧燕; 唐成润; 商潘磊; 车安兵; 李璠 | 国家组织药品集采执行效果分析——以云南省为例 | 中国医疗保险 | 10.19546/j.issn.1674-3830.2023.4.002 | E4 No clinical, safety or pharmacokinetic outcome |
| 849 | 王艳杰 | 国家药品新政策对社区医疗机构药品价格的影响与思考 | 继续医学教育 | 10.3969/j.issn.1004-6763.2019.07.088 | E4 No clinical, safety or pharmacokinetic outcome |
| 850 | 王浩扬; 韩悦; 谢金平; 邵蓉 | 国家药品集中带量采购及接续政策对药品用量、价格及费用的影响研究 | 中国卫生经济 |  | E4 No clinical, safety or pharmacokinetic outcome |
| 868 | 李占武; 李瑞珂; 刘伟; 刘淡宜 | 基于CiteSpace软件分析医院药品精细化管理的现状 | 医学临床研究 | 10.3969/j.issn.1671-7171.2025.02.001 | E4 No clinical, safety or pharmacokinetic outcome |
| 878 | 朱欣叶; 钱爱兵; 时孝春; 阮智慧; 梁晓玲 | 基于PMC指数模型的我国药品集中带量采购政策量化分析 | 中国卫生事业管理 |  | E4 No clinical, safety or pharmacokinetic outcome |
| 1420 | Chen, L; Yang, Y; Luo, M; Hu, BR; Yin, SC; Mao, ZF | The Impacts of National Centralized Drug Procurement Policy on Drug Utilization and Drug Expenditures: The Case of Shenzhen, China | INTERNATIONAL JOURNAL OF ENVIRONMENTAL RESEARCH AND PUBLIC H | 10.3390/ijerph17249415 | E4 No clinical, safety or pharmacokinetic outcome |
| 1424 | Wang, JL; Zhang, SY; Wang, CH; Li, J; Wang, R; Zhu, L | The vacated space of volume/price of the drugs centralized procurement with quantity in secondary and above public hospitals of China | BMC HEALTH SERVICES RESEARCH | 10.1186/s12913-024-11217-3 | E4 No clinical, safety or pharmacokinetic outcome |
| 1 | 郭晋晖 | 14万份真实病例数据：集采仿制药疗效与原研药相当 | 第一财经日报 | 10.28207/n.cnki.ndycj.2023.004117 | E5 Not original research |
| 3 | 柴嵘 | 63种国家集采药品完成临床研究 | 北京日报 | 10.28033/n.cnki.nbjrb.2024.010184 | E5 Not original research |
| 12 | 胡睿 | “4+7”仿制药替代显效三甲医院支持率攀升 | 医药经济报 | 10.38275/n.cnki.nyyjj.2021.000283 | E5 Not original research |
| 13 | 戴小河; 傅苏颖 | “4+7”带量采购品种料扩围 | 中国证券报 | 10.28162/n.cnki.nczjb.2019.002852 | E5 Not original research |
| 14 |  | “4+7”带量采购方案发布 | 中国药店 |  | E5 Not original research |
| 15 | 朱萍; 武瑛港 | “4+7”带量采购试点即将开标医保强支付时代或开启 | 21世纪经济报道 |  | E5 Not original research |
| 16 | 徐翔英; 魏盈; 毕红玉; 刘匡一; 熊国营 | “4+7”带量采购试点扩围在某三甲医院年度实施效果 | 药品评价 | 10.19939/j.cnki.1672-2809.2021.19.03 | E5 Not original research |
| 17 | 陈聪 | “4+7”试点促我国仿制药质量提升 | 新华每日电讯 | 10.28870/n.cnki.nxhmr.2019.001150 | E5 Not original research |
| 19 | 孙秀艳 | “血压不降、麻药不睡、泻药不泻”？ | 人民日报 | 10.28655/n.cnki.nrmrb.2025.001550 | E5 Not original research |
| 20 |  | “血压不降、麻药不睡、泻药不泻”？医保和药监部门这样回应 | 新华每日电讯 | 10.28870/n.cnki.nxhmr.2025.000954 | E5 Not original research |
| 21 | 李丹青 | “血压不降、麻药不睡、泻药不泻”？官方回应来了 | 工人日报 | 10.28277/n.cnki.ngrrb.2025.000699 | E5 Not original research |
| 22 | 彭韵佳 | “降价不降质”集采中选仿制药与原研药临床疗效一致 | 经济参考报 | 10.28419/n.cnki.njjck.2021.003324 | E5 Not original research |
| 23 |  | 《关于以药品集中采购和使用为突破口进一步深化医药卫生体制改革的若干政策措施》印发 | 中国医药报 |  | E5 Not original research |
| 24 | 陆悦 | 一致性评价推进药品质量提升 | 中国医药报 | 10.38249/n.cnki.nyiya.2020.001597 | E5 Not original research |
| 25 | 刘志勇 | 一致性评价洗牌医药江湖 | 健康报 |  | E5 Not original research |
| 29 | 耿鸿武 | 中成药/生物制剂集中带量采购循序渐进 | 医药经济报 | 10.38275/n.cnki.nyyjj.2021.001328 | E5 Not original research |
| 32 | 董梅; 张建中; 刘炜 | 临床常用静脉输液调配与使用 |  |  | E5 Not original research |
| 35 | 邱英鹏; 李理然; 赵羽西; 肖月 | 人工晶体卫生技术评估方法学分析及启示 | 中国卫生质量管理 | 10.13912/j.cnki.chqm.2021.28.5.02 | E5 Not original research |
| 36 | 彭韵佳; 黄凯莹; 林苗苗 | 从“打折到脚踝”的心脏支架看集采产品降价不降质 | 经济参考报 | 10.28419/n.cnki.njjck.2024.000969 | E5 Not original research |
| 39 | 落楠 | 仿制药一致性评价为药品集采提供有力支撑 | 中国医药报 | 10.38249/n.cnki.nyiya.2021.001153 | E5 Not original research |
| 40 | 张维 | 仿制药与原研药按相同标准支付 | 法制日报 |  | E5 Not original research |
| 41 | 赵波 | 仿制药，让“救命药”降下“身段” | 青岛日报 |  | E5 Not original research |
| 43 | 韩利明 | 低价药质量与用药选择权：集采下的患者需求引热议国家医保局发函回应 | 21世纪经济报道 | 10.28723/n.cnki.nsjbd.2025.000298 | E5 Not original research |
| 44 | 董超 | 促进医保、医疗、医药协同发展 | 保健时报 | 10.28034/n.cnki.nbjsb.2024.000160 | E5 Not original research |
| 45 | 季媛媛 | 全国中成药集采陆续落地 | 21世纪经济报道 | 10.28723/n.cnki.nsjbd.2023.004295 | E5 Not original research |
| 46 | 张茜 | 全年预计节约医疗费用1.6亿元 | 济南日报 | 10.28453/n.cnki.njnrb.2020.000424 | E5 Not original research |
| 47 | 陶凤 | 关于集采药疗效，专业声音值得被听见 | 北京商报 | 10.28036/n.cnki.nbjxd.2025.000182 | E5 Not original research |
| 49 | 李丹青 | 几角钱的仿制药如何保证质量？ | 工人日报 | 10.28277/n.cnki.ngrrb.2023.005132 | E5 Not original research |
| 51 | 梁倩 | 剑指降药价政策红利频频释放 | 经济参考报 |  | E5 Not original research |
| 52 | 金观平 | 努力让新药好药“用得起买得到” | 经济日报 | 10.28425/n.cnki.njjrb.2025.006144 | E5 Not original research |
| 53 | 孟刚 | 化药注射剂一致性评价落地 | 中国消费者报 | 10.28867/n.cnki.nxfzb.2020.000880 | E5 Not original research |
| 54 | 刘可 | 化药注射液仿制药一致性评价启动行业竞争愈加激烈 | 经济观察报 | 10.28421/n.cnki.njjgc.2020.000150 | E5 Not original research |
| 56 | 童荣生; 赵杰; 王明贵; 李小丝; 崔小娇; 梁淑红 | 医疗机构抗菌药物集中带量采购管理专家共识 | 医药导报 |  | E5 Not original research |
| 57 | 刘昶荣 | 十四万病例证明集采仿制药安全有效性和原研药相当 | 中国青年报 | 10.38302/n.cnki.nzgqn.2023.004838 | E5 Not original research |
| 58 | 崔永耀; 杨静 | 原研还是仿制？不仅仅是经济账 | 健康报 | 10.28415/n.cnki.njika.2020.000107 | E5 Not original research |
| 59 | 汪斌; 刘启国 | 厦门开出“4+7”中选药品采购第一单 | 中国医药报 |  | E5 Not original research |
| 61 | 周萍 | 同发力推动集采常态化下药品重点监管 | 中国市场监管报 | 10.28075/n.cnki.ncgsb.2021.000679 | E5 Not original research |
| 63 |  | 国务院：十五项举措深化药品集中采购 | 中国招标 |  | E5 Not original research |
| 64 |  | 国务院：推动药品带量采购实行市场清退制度 | 中国招标 |  | E5 Not original research |
| 65 | 吴少杰 | 国家医保局召开医药集中带量采购座谈会 | 健康报 | 10.28415/n.cnki.njika.2025.000040 | E5 Not original research |
| 66 | 柴嵘 | 国家医保局：仿制药无疗效差异 | 北京日报 | 10.28033/n.cnki.nbjrb.2025.000800 | E5 Not original research |
| 67 | 梁倩 | 国家医保局：集采中选仿制药的临床疗效和安全性与原研药相当 | 经济参考报 | 10.28419/n.cnki.njjck.2023.003722 | E5 Not original research |
| 69 | 吴少杰 | 国家药品集采规则有哪些变化 | 健康报 | 10.28415/n.cnki.njika.2025.003609 | E5 Not original research |
| 71 | 刘琰 | 国家集中带量采购抗菌药物的临床综合评价研究 |  |  | E5 Not original research |
| 76 | 马相 | 国家集采是什么？为什么集采药品降价那么多？ | 西安日报 | 10.28843/n.cnki.nxarb.2022.001441 | E5 Not original research |
| 92 | 董名扬 | 基于层次分析模型的第三代头孢类抗菌药物治疗儿童细菌性肺炎的临床综合评价 |  | 10.27652/d.cnki.gzyku.2023.000853 | E5 Not original research |
| 101 | 李晓青 | 多措并举确保患者用得上中选药品 | 乌鲁木齐晚报(汉) | 10.28830/n.cnki.nwlwb.2020.001757 | E5 Not original research |
| 110 | 马晓华 | 带量采购如何不伤害医药行业？业界称要解决5个核心问题 | 第一财经日报 |  | E5 Not original research |
| 111 | 张兰 | 带量采购开启仿制药发展“元年” | 中国卫生 | 10.15973/j.cnki.cn11-3708/d.2019.12.021 | E5 Not original research |
| 114 | 齐欣 | 带量采购鼓励“价格+模式”联动 | 医药经济报 |  | E5 Not original research |
| 116 | 陈吉生; 陈永; 曹伟灵 | 广东省外科围手术期预防用药常用头孢菌素注射剂临床快速综合评价专家共识（2023版） | 今日药学 |  | E5 Not original research |
| 125 | 钟晨 | 我市首张“4+7”药品处方开出 | 东莞日报 | 10.28181/n.cnki.ndgrb.2020.000014 | E5 Not original research |
| 126 | 王敏娜 | 我省完善仿制药供应保障使用政策 | 辽宁日报 |  | E5 Not original research |
| 128 |  | 把较高质量风险产品排除在集采之外 | 北京商报 | 10.28036/n.cnki.nbjxd.2025.000162 | E5 Not original research |
| 130 | 吴刚; 朱琳 | 抗菌药集采需全流程科学化管理 | 健康报 | 10.28415/n.cnki.njika.2024.001835 | E5 Not original research |
| 131 |  | 持续完善集采制度消除公众用药担忧 | 第一财经日报 | 10.28207/n.cnki.ndycj.2025.000199 | E5 Not original research |
| 132 | 叶阳欢; 张雅静 | 按下发展“快进键” | 吉林日报 | 10.28447/n.cnki.njlrb.2020.005178 | E5 Not original research |
| 133 | 朱萍 | 振兴中医药破解临床评价和质量控制难题成关键 | 21世纪经济报道 | 10.28723/n.cnki.nsjbd.2021.000876 | E5 Not original research |
| 134 | 陈子仪; 何颖 | 探索良药入保新方案 | 海南日报 | 10.28356/n.cnki.nhlrb.2024.006018 | E5 Not original research |
| 136 |  | 推动医药集采扩围提质 | 经济日报 | 10.28425/n.cnki.njjrb.2025.006279 | E5 Not original research |
| 137 | 吴娜 | 支持新药好药跑出上市“加速度” | 北京日报 | 10.28033/n.cnki.nbjrb.2024.003374 | E5 Not original research |
| 140 |  | 新政 | 光彩 |  | E5 Not original research |
| 141 | 陶凤; 常蕾 | 明年底前疫苗将一物一码 | 北京商报 |  | E5 Not original research |
| 147 | 樊华; 迟敬 | 沈阳32种药品最高降幅96% | 沈阳日报 | 10.28770/n.cnki.nsyrb.2020.001125 | E5 Not original research |
| 148 | 陆悦 | 注射剂一致性评价工作步入常态化 | 中国医药报 | 10.38249/n.cnki.nyiya.2021.000273 | E5 Not original research |
| 150 | 陈海荣 | 深圳全方位落实国家“4+7”试点工作 | 中国医药报 |  | E5 Not original research |
| 153 | 沐铁城; 彭韵佳 | 疗效与安全性如何？有哪些评价指标？ | 新华每日电讯 | 10.28870/n.cnki.nxhmr.2023.006225 | E5 Not original research |
| 155 |  | 省卫生健康委等九部门关于印发贵州省关于贯彻落实国家基本药物制度的实施意见的通知 | 贵州省人民政府公报 |  | E5 Not original research |
| 156 | 张兰; 董宪喆; 王之舟 | 真实世界研究在国产仿制药临床疗效与安全性评价中的应用及展望 | 药物不良反应杂志 | 10.3760/cma.j.cn114015-20230103-01203 | E5 Not original research |
| 157 | 吴佳佳 | 确保百姓用药质优价宜 | 经济日报 | 10.28425/n.cnki.njjrb.2025.007039 | E5 Not original research |
| 158 | 张小洁 | 第五批药品国采本周开标多家上市药企“踩点”过评 | 经济参考报 | 10.28419/n.cnki.njjck.2021.003455 | E5 Not original research |
| 159 | 柴嵘 | 第十批国家组织药品集采开标 | 北京日报 | 10.28033/n.cnki.nbjrb.2024.009503 | E5 Not original research |
| 160 | 江开达 | 精神疾病的特殊性与开展带量采购的思考 | 国际精神病学杂志 | 10.13479/j.cnki.jip.2021.03.005 | E5 Not original research |
| 161 |  | 织密民生保障网增强群众幸福感 | 海南日报 | 10.28356/n.cnki.nhlrb.2024.000471 | E5 Not original research |
| 163 | 巢艾伦 | 美国仿制药上市后药物警戒活动分析研究和对我国集采药品的启示 | 中国食品药品监管 | 10.3969/j.issn.1673-5390.2023.01.006 | E5 Not original research |
| 166 | 黄晶 | 胰岛素集采突破生物药重围 | 医师报 | 10.44211/n.cnki.nysbz.2021.001226 | E5 Not original research |
| 167 | 叶龙杰 | 药价降低后的真实世界 | 健康报 | 10.28415/n.cnki.njika.2022.000270 | E5 Not original research |
| 168 | 顾莹 | 药企备战中成药集采难在如何标准化 | 第一财经日报 | 10.28207/n.cnki.ndycj.2021.003761 | E5 Not original research |
| 169 | 张雪 | 药品采购“国家第一标”来了药企或面临不同境遇 | 上海证券报 |  | E5 Not original research |
| 170 | 龙华; 胡雯洁 | 药品采购改革引发“复合效应” | 湖北日报 | 10.28310/n.cnki.nhbbr.2020.009570 | E5 Not original research |
| 173 |  | 药品集中采购:带量采购谁是最大赢家? | 中国招标 |  | E5 Not original research |
| 174 | 陈思煌; 谢菡; 魏璟璇; 胡美玲; 王鑫梅; 李俐 | 药品集中采购政策背景下仿制药与原研药有效性和安全性对比的系统综述 | 中南药学 | 10.7539/j.issn.1672-2981.2023.10.039 | E5 Not original research |
| 175 | 戴小河; 傅苏颖 | 药品集采试点全国扩围拟中选结果出炉 | 中国证券报 | 10.28162/n.cnki.nczjb.2019.001955 | E5 Not original research |
| 176 | 郑莹莹 | 落地措施引热议上海模式面面观 | 医药经济报 |  | E5 Not original research |
| 177 | 陈海波 | 让人放心的仿制药离不开一致性评价这一环 | 光明日报 | 10.28273/n.cnki.ngmrb.2021.003486 | E5 Not original research |
| 178 | 郭晋晖 | 课题组研究了11万份病例：14个集采仿制药与原研药临床等效 | 第一财经日报 | 10.28207/n.cnki.ndycj.2021.002581 | E5 Not original research |
| 179 | 阎红; 车晨菲 | 辽宁完善仿制药采购使用政策 | 健康报 |  | E5 Not original research |
| 180 |  | 辽宁省人民政府办公厅关于改革完善仿制药供应保障及使用政策的实施意见 | 辽宁省人民政府公报 |  | E5 Not original research |
| 184 | 储白珊 | 降价不降质药效不“打折” | 福建日报 | 10.28232/n.cnki.nfjrb.2020.001595 | E5 Not original research |
| 189 | Pharmacoeconomics Committee of Shandong Hospital Association | 集中带量采购政策下山东省调脂药物临床综合评价专家共识 | 中国药房 | 10.6039/j.issn.1001-0408.2022.15.01 | E5 Not original research |
| 190 | 张卫芳 | 集中带量采购政策下江西省口服二氢吡啶类钙离子通道阻滞剂药品临床综合评价专家共识 | 中国现代应用药学 | 10.13748/j.cnki.issn1007-7693.20223990 | E5 Not original research |
| 191 | 董艳珍; 宋沧桑; 李兴德; 刘璐 | 集中带量采购政策下的药品临床综合评价研究进展 | 中南药学 |  | E5 Not original research |
| 195 | 贠娟绸 | 集中采购，让群众用上质优价廉的药品 | 山西经济日报 | 10.28755/n.cnki.nsxjj.2019.000141 | E5 Not original research |
| 196 | 刘卉 | 集采“限价”“一品两规”难题何解？ | 医药经济报 |  | E5 Not original research |
| 199 | 吴佳佳 | 集采中选的仿制药疗效不如原研药吗 | 经济日报 | 10.28425/n.cnki.njjrb.2023.007166 | E5 Not original research |
| 201 | 柴嵘 | 集采仿制药未发现疗效差异 | 北京晚报 | 10.48993/n.cnki.nbjwb.2025.000016 | E5 Not original research |
| 202 | 胡睿 | 集采品种真实世界数据出炉中选仿制药品质过硬 | 医药经济报 | 10.38275/n.cnki.nyyjj.2021.000776 | E5 Not original research |
| 203 | 边界 | 集采引发一致性评价热度迫降？ | 医药经济报 | 10.38275/n.cnki.nyyjj.2022.000773 | E5 Not original research |
| 204 | 傅苏颖 | 集采推动仿制药质量水平提升 | 中国证券报 | 10.28162/n.cnki.nczjb.2023.005090 | E5 Not original research |
| 206 | 骆兵; 杨贤; 李依璇; 严思敏; 兰希; 鲍雪 | 集采背景下国产对比进口氯吡格雷治疗冠心病的疗效与安全性的Meta分析 | 中国循证心血管医学杂志 | 10.3969/j.issn.1674-4055.2023.06.02 | E5 Not original research |
| 209 | 叶龙杰 | 集采药品疗效和安全性与原研药相当 | 健康报 | 10.28415/n.cnki.njika.2023.002900 | E5 Not original research |
| 210 | 李丹青 | 集采阿司匹林一片3分钱，质量有保证吗？ | 工人日报 | 10.28277/n.cnki.ngrrb.2025.000089 | E5 Not original research |
| 212 | 郭婷 | 集采：努力寻求多方平衡点 | 中国医药报 | 10.38249/n.cnki.nyiya.2025.000075 | E5 Not original research |
| 213 |  | 青海省人民政府办公厅关于印发青海省改革完善仿制药供应保障及使用政策实施方案的通知 | 青海政报 |  | E5 Not original research |
| 214 | 徐莹波 | “桂林造”阿莫西林胶囊通过仿制药一致性评价 | 桂林日报 | 10.28272/n.cnki.nglrb.2019.000323 | E5 Not original research |
| 217 | 王天鹅 | 以患者为中心，走高质量发展之路 | 健康报 |  | E5 Not original research |
| 231 | 梁明娟; 陈志红; 翟琼香; 邱炜凤; 黎雪萍 | 国产左乙拉西坦替换治疗的疗效及安全性分析 | 第九届CAAE国际癫痫论坛 | 10.26914/c.cnkihy.2021.077391 | E5 Not original research |
| 232 | 刘志勇 | 国家团购：“4＋7”平稳落地 | 健康报 |  | E5 Not original research |
| 235 | 王鑫宇 | 基于两项仿制药BE试验并结合文献分析剂量/体重/饮食对恩格列净PK/PD的影响 |  | 10.27288/d.cnki.gsxyu.2025.001419 | E5 Not original research |
| 237 | 茅关兴; 赵丽敏; 李琦君; 徐诗强 | 奥美拉唑肠溶胶囊人体生物等效性临床研究 | 上海医药 | 10.3969/j.issn.1006-1533.2024.15.024 | E5 Not original research |
| 239 | 刘曼; 刘会臣 | 对国内奥美拉唑肠溶制剂生物等效性试验的分析 | 中国新药杂志 | 10.3969/j.issn.1003-3734.2023.06.009 | E5 Not original research |
| 241 | 曹建英; 曲秀君; 宋玉环; 臧彩红; 寻亚慧; 石仲歌 | 左乙拉西坦仿制药替换原研药治疗癫痫有效性及安全性的Meta分析 | 中国医院药学杂志 | 10.13286/j.1001-5213.2025.18.12 | E5 Not original research |
| 242 | 李丹; 石永晶; 余静洁; 杨琳; 黄绍平; 张抗怀 | 左乙拉西坦品牌药转换为仿制药对儿童癫痫患者血药浓度及临床疗效的影响一项前瞻性自身对照研究 | 第九届CAAE国际癫痫论坛 | 10.26914/c.cnkihy.2021.077447 | E5 Not original research |
| 245 | 陆璐; 吴欣桐; 肖静; 周东 | 抗癫痫药物左乙拉西坦仿制药有效性及安全性：多中心真实世界研究 | 第九届CAAE国际癫痫论坛 | 10.26914/c.cnkihy.2021.077363 | E5 Not original research |
| 246 | 王玮丽 | 推动产业链创新链价值链“三链融合” | 南通日报 | 10.28606/n.cnki.nntrb.2021.000945 | E5 Not original research |
| 247 | 贺锐锐; 杨进波 | 浅谈同一API不同成盐药物一致性评价中可能存在的问题 | 中国食品药品监管 |  | E5 Not original research |
| 249 | 梁永超; 朱莹; 赵志霞; 刘丽宏 | 硼替佐米治疗多发性骨髓瘤的研究现状 | 中国临床药理学杂志 | 10.13699/j.cnki.1001-6821.2021.15.038 | E5 Not original research |
| 253 | 刘曼; 张育玮; 郭俊荣; 潘伟东; 刘会臣 | 苯磺酸氨氯地平片生物等效性试验的现状 | 中国临床药理学杂志 | 10.13699/j.cnki.1001-6821.2020.07.043 | E5 Not original research |
| 255 | 李佳; 李嘉欣; 王钰; 闫佳佳; 陈杰 | 阿哌沙班仿制药与原研药在膝关节置换手术围术期的有效性和安全性比较 | 第十六届药源性疾病与安全用药中国论坛 | 10.26914/c.cnkihy.2024.090093 | E5 Not original research |
| 262 | 王婷婷; 洪建文 | 阿莫西林胶囊国内外现状比较分析 | 国外医药(抗生素分册) | 10.13461/j.cnki.wna.005208 | E5 Not original research |
| 282 | Jamison DT; Summers LH; Chang AY; Karlsson O; Mao W; Norheim | Global health 2050: the path to halving premature death by mid-century. | Lancet (London, England) | 10.1016/S0140-6736(24)01439-9 | E5 Not original research |
| 296 | Cao K; Kam YW | Literature Review and Policy Recommendations for Single-Dose HPV Vaccination Schedule in China: Opportunities and Challenges. | Vaccines | 10.3390/vaccines13080786 | E5 Not original research |
| 302 | Parra-Herran C; Romero Y; Milner D | Pathology and Laboratory Medicine in cancer care: A global analysis of national cancer control plans. | International journal of cancer | 10.1002/ijc.33384 | E5 Not original research |
| 318 | Zhao C; Wang Y; Shao Y | A Bioequivalence Trial of Dienogest in Healthy Chinese Participants Under Fed Conditions. | Clinical pharmacology in drug development | 10.1002/cpdd.1324 | E5 Not original research |
| 319 | Shao J; Liu X; Zhang G; Xiang A; Xie X | A Bioequivalence Trial of Lenvatinib Mesylate Capsules in Healthy Subjects Under Fasting and Postprandial Conditions. | Clinical pharmacology in drug development | 10.1002/cpdd.1470 | E5 Not original research |
| 320 | Xie Q; Chen J; Yang H; Liang J; Ma R; Guo J | A Comprehensive Review of Coptidis Rhizoma and Magnoliae Officinalis Cortex Drug Pair and Their Chemical Composition, Pharmacological Effects and Phar | Drug design, development and therapy | 10.2147/DDDT.S477381 | E5 Not original research |
| 331 | Peng X; Liu Y; Li H; Lei Y; Gan F; Wu Y | A Randomized, Open-Label, Two-Sequence, Crossover Trial Evaluating the Bioequivalence, and Pharmacokinetics of Two Sulfamethoxazole/Trimethoprim Table | Clinical pharmacology in drug development | 10.1002/cpdd.1537 | E5 Not original research |
| 334 | Ge B; Li C; Wei H; Ding Y; Wu M; Liu Y | A Reference-Scaled Average Bioequivalence Study of Azithromycin Tablets Manufactured in China and the United States: An Open-Label, Randomized, Single | Clinical pharmacology in drug development | 10.1002/cpdd.1132 | E5 Not original research |
| 335 | Huang K; Yin S; Huang W; Ding L; Qin W; Qian Z | A Single Dose, Randomized, Open-Label, Cross-Over Bioequivalence Study of Budesonide Pressurized Metered-Dose Inhaler in Healthy Chinese Subjects. | Pharmacology research & perspectives | 10.1002/prp2.70197 | E5 Not original research |
| 338 | Li X; He A; Liu B; Shan R; Zhu J; Li X | A comparative evaluation of bioequivalence of Gan & Lee glargine U300 and Toujeo(®) in Chinese healthy male participants. | Frontiers in endocrinology | 10.3389/fendo.2024.1407829 | E5 Not original research |
| 346 | Liu H; Li T; Yu H; Chen X; Li J; Tan H | A phase-I randomized euglycemic clamp study to demonstrate the pharmacokinetic and pharmacodynamic equivalence of an insulin degludec biosimilar (B014 | Expert opinion on investigational drugs | 10.1080/13543784.2023.2254690 | E5 Not original research |
| 351 | Xu Z; Wang Y; Liu G; Chen J; Wang W; Cheng Y | A randomized, open-label, single-dose, two-cycle crossover study to evaluate the bioequivalence and safety of lenvatinib and Lenvima® in Chinese healt | Expert opinion on investigational drugs | 10.1080/13543784.2022.2067528 | E5 Not original research |
| 352 | Liu G; Xue J; Wang Y; Liu Z; Li X; Qu D | A randomized, open-label, two-cycle, two-crossover phase I clinical trial comparing the bioequivalence and safety of afatinib and Giotrif(®) in health | Journal of cancer research and clinical oncology | 10.1007/s00432-022-04148-1 | E5 Not original research |
| 355 | Sun J; Hu CJ; Stuntz M; Hogerzeil H; Liu Y | A review of promoting access to medicines in China - problems and recommendations. | BMC health services research | 10.1186/s12913-018-2875-6 | E5 Not original research |
| 357 | Yang Z; Li Q; Yu D; Zhang X; Wang Y; Liu S | A single-dose, four-cycle, fully repetitive crossover bioequivalence of dabigatran etexilate in Chinese. | BMC pharmacology & toxicology | 10.1186/s40360-025-00896-1 | E5 Not original research |
| 365 | White CM; White LR | Believing FDA's Assurance of Quality Pharmaceutical Products Can Be Dangerous to Your Patients' Health. | The Annals of pharmacotherapy | 10.1177/10600280251343626 | E5 Not original research |
| 366 | Yan K; Ju G; Tan Q; Zeng L; Qiu W | Bioequivalence Analysis of 2 Dapoxetine Hydrochloride Formulations in Healthy Chinese Male Volunteers Under Fed and Fasting Conditions: A Randomized, | Clinical pharmacology in drug development | 10.1002/cpdd.866 | E5 Not original research |
| 367 | Wang Z; Wen H; Qian X; Huang M; Cheng G; Zhong G | Bioequivalence Analysis of Clindamycin Hydrochloride Capsules in Healthy Chinese Subjects Under Fasted and Fed Conditions. | Clinical pharmacology in drug development | 10.1002/cpdd.1480 | E5 Not original research |
| 369 | Li Y; Zhang Z; Xie J; Lian X; Zhang G; Wang C | Bioequivalence Assessment of Two Dapoxetine Hydrochloride Formulations in Healthy Chinese Males Under Fasted and Fed Conditions. | Clinical pharmacology in drug development | 10.1002/cpdd.1393 | E5 Not original research |
| 374 | Yang D; Wang X; Duan Y; Xu Y; Ruan Z; Jiang B | Bioequivalence Study of Epalrestat for Healthy Chinese Subjects. | Clinical pharmacology in drug development | 10.1002/cpdd.1347 | E5 Not original research |
| 375 | Zhao MX; Wu JL; Dong LC; Chen J; Zhu FJ; Fan YX | Bioequivalence Study of Miglitol Orally Disintegrating Tablets in Healthy Chinese Volunteers Under Fasting Condition Based on Pharmacodynamic and Phar | Clinical pharmacology in drug development | 10.1002/cpdd.1268 | E5 Not original research |
| 377 | Lei Y; Liu Y; Gao L; Peng X; Chen L; Li C | Bioequivalence Study of Two Empagliflozin Tablets in Healthy Chinese Subjects Under Fasting and Fed Conditions. | Clinical pharmacology in drug development | 10.1002/cpdd.1530 | E5 Not original research |
| 378 | Yang Y; Kai J; Lv D; Zhou H; Yu Y; Shentu J | Bioequivalence Study of Two Formulations of Flunarizine Hydrochloride Capsules in Healthy Chinese Subjects Under Fasting and Fed Conditions. | Drugs in R&D | 10.1007/s40268-025-00521-w | E5 Not original research |
| 380 | Bai W; Song H; Hu Y; Zhang X; Wang X; Guo C | Bioequivalence Study of Vortioxetine Hydrobromide Tablets in Healthy Chinese Subjects Under Fasting and Fed Conditions. | Drug design, development and therapy | 10.2147/DDDT.S428771 | E5 Not original research |
| 385 | Wang S; Ren J; Wang H; Zhi T; Zhu Y; Feng J | Bioequivalence and Pharmacokinetic Evaluation of 2 Pyrazinamide Formulations in Healthy Chinese Adults: A Single-Dose, Open-Label, Randomized-Sequence | Clinical pharmacology in drug development | 10.1002/cpdd.1035 | E5 Not original research |
| 386 | Wang Z; Zhang Y; Liu J; Chen X | Bioequivalence and Pharmacokinetic Evaluation of Regorafenib Tablets in Healthy Chinese Volunteers. | Clinical pharmacology in drug development | 10.1002/cpdd.1474 | E5 Not original research |
| 387 | Huang Y; Cai Q; Li M; Guo S; Dong G; Hu S | Bioequivalence and Pharmacokinetic Evaluation of Two Metformin Hydrochloride Tablets Under Fasting and Fed Conditions in Healthy Chinese Volunteers. | European journal of drug metabolism and pharmacokinetics | 10.1007/s13318-025-00961-4 | E5 Not original research |
| 388 | Zhang Q; Wang Z; Wu J; Zhou Z; Zhou R; Hu W | Bioequivalence and Pharmacokinetic Evaluation of Two Oral Formulations of Regorafenib: An Open-Label, Randomised, Single-Dose, Two-Period, Two-Way Cro | Drug design, development and therapy | 10.2147/DDDT.S323169 | E5 Not original research |
| 390 | Sun ML; Xu XW; Liu C; Tong YX; Wei YL; Liu HJ | Bioequivalence and Safety Assessment of 2 Formulations of Low-Dose Metformin Hydrochloride under Fasting Conditions in Healthy Chinese Participants: A | Clinical pharmacology in drug development | 10.1002/cpdd.1369 | E5 Not original research |
| 391 | Sun ML; Liu HJ; Luo XD; Wang Y; Zhang W; Liu C | Bioequivalence and Safety Assessment of Two Formulations of Metformin Hydrochloride Sustained-Release Tablets (Yuantang(®) SR and Glucophage(®) XR) Un | Drugs in R&D | 10.1007/s40268-021-00377-w | E5 Not original research |
| 392 | Liu Y; Zou Y; Wang Y; Jiang F; Xu W; Liu S | Bioequivalence and Safety of Levetiracetam Granules and Oral Solution: A Randomized, Single-Dose, 2-Period Crossover Study in Healthy Chinese Voluntee | Clinical pharmacology in drug development | 10.1002/cpdd.1063 | E5 Not original research |
| 393 | Wang M; Peng Y; Yan H; Pan Z; Du R; Liu G | Bioequivalence and Safety of Two Amisulpride Formulations in Healthy Chinese Subjects Under Fasting and Fed Conditions: A Randomized, Open‑Label, Sing | Drugs in R&D | 10.1007/s40268-025-00508-7 | E5 Not original research |
| 394 | Yu H; Fang Y; Qi X; Luo P; He Z; Wang K | Bioequivalence and Tolerability of 2 Lurasidone Formulations: A Randomized, Single-Dose, 2-Period, Crossover Study in Healthy Chinese Subjects Under F | Clinical pharmacology in drug development | 10.1002/cpdd.1529 | E5 Not original research |
| 399 | Cao Y; Su J | Bioequivalence of 200 mg Amisulpride Tablets in Healthy Chinese Volunteers under Fasting and Fed Conditions. | Clinical pharmacology in drug development | 10.1002/cpdd.1348 | E5 Not original research |
| 401 | Dong L; Zhao M; Huang H; Guan R; Li R; Fan Y | Bioequivalence of Celecoxib Capsules in Chinese Healthy Volunteers. | Clinical pharmacology in drug development | 10.1002/cpdd.1270 | E5 Not original research |
| 402 | Hu S; Liu X; Wan Q; Zhang X; Gong F | Bioequivalence of Meloxicam Nanocrystal Injection in Healthy Chinese Volunteers. | Clinical pharmacology in drug development | 10.1002/cpdd.1467 | E5 Not original research |
| 404 | Wang S; Guo S; Han Y; Ni S; Wu F; Dai Y | Bioequivalence of Two Linezolid Tablets Under Fed and Fasting Conditions in Healthy Chinese Individuals. | Clinical pharmacology in drug development | 10.1002/cpdd.1155 | E5 Not original research |
| 405 | Yu M; Li X; Jin H; Chen L; Wang N; Wang H | Bioequivalence of a Generic Nateglinide Formulation in Healthy Chinese Volunteers under Fasting and Fed Conditions: A Randomized, Open-Label, Double-C | Pharmacology | 10.1159/000512851 | E5 Not original research |
| 406 | Bai W; Shi S; Yin D; Guo C; Song H; Hu Y | Bioequivalence of a single dose of two palbociclib formulations in healthy Chinese subjects under fasting conditions: a two-period crossover study wit | Frontiers in pharmacology | 10.3389/fphar.2025.1722151 | E5 Not original research |
| 407 | Li X; Wang C; Li T; Liu Y; Liu S; Tao Y | Bioequivalence of levamlodipine besylate tablets in healthy Chinese subjects: a single-dose and two-period crossover randomized study. | BMC pharmacology & toxicology | 10.1186/s40360-020-00459-6 | E5 Not original research |
| 408 | Liu ZZ; Ren Q; Zhou YN; Yang HM | Bioequivalence of two esomeprazole magnesium enteric-coated formulations in healthy Chinese subjects. | World journal of clinical cases | 10.12998/wjcc.v8.i22.5518 | E5 Not original research |
| 411 | Xu J; Zhao X; Wang Z; Zhou Y; Yang X; Cui Y | Bioequivalence study of the antiepileptic drug levetiracetam in concentrated solution for injection versus oral tablets in healthy Chinese subjects un | International journal of clinical pharmacology and therapeut | 10.5414/CP203274 | E5 Not original research |
| 416 | Hu C; Gao D; Li D; Zhou D; Zhang L | Chinese- and French-Manufactured Immediate-Release Glucophage(®) Bioequivalence: A Randomized, Open-Label, Crossover Study. | Drugs in R&D | 10.1007/s40268-022-00405-3 | E5 Not original research |
| 421 | Liu Y; Peng X; Zhao M; Liu F; Wang X; Chen L | Comparative Pharmacokinetics and Bioequivalence of Two Sodium Valproate Tablets in Healthy Chinese Subjects Under Fasting and Fed Conditions. | Clinical pharmacology in drug development | 10.1002/cpdd.1563 | E5 Not original research |
| 434 | Zhang H; Liu C; Zheng Q | Development and application of anthelminthic drugs in China. | Acta tropica | 10.1016/j.actatropica.2019.105181 | E5 Not original research |
| 441 | Sun X; Song H; Liu H; Qiu B; Ding C; Hu Y | Effect of a High-Fat Diet on the Pharmacokinetics and Safety of Rivaroxaban in Healthy Chinese Subjects. | Clinical pharmacology in drug development | 10.1002/cpdd.1330 | E5 Not original research |
| 443 | Chen J; Lou H; Jiang B; Shao R; Yang D; Hu Y | Effects of Food and Gender on Pharmacokinetics of Rosuvastatin in a Chinese Population Based on 4 Bioequivalence Studies. | Clinical pharmacology in drug development | 10.1002/cpdd.706 | E5 Not original research |
| 453 | Yang R; Lin Y; Chen K; Huang J; Yang S; Yao A | Establishing Virtual Bioequivalence and Clinically Relevant Specifications for Omeprazole Enteric-Coated Capsules by Incorporating Dissolution Data in | The AAPS journal | 10.1208/s12248-024-00956-0 | E5 Not original research |
| 458 | Ju G; Yan K; Xu Y; Chen S; Zheng Z; Qiu W | Evaluation of Bioequivalency and Pharmacokinetic Parameters for Two Formulations of Glimepiride 1-mg in Chinese Subjects. | Drug design, development and therapy | 10.2147/DDDT.S249355 | E5 Not original research |
| 460 | Wang Y; He Y; Li W; Li H; Tang L; Dai X | Evaluation of Ornidazole Tablets Bioequivalence in Chinese Healthy Participants Under Fasted and Fed Conditions Using Pharmacokinetic Parameters. | Drugs in R&D | 10.1007/s40268-024-00457-7 | E5 Not original research |
| 461 | Wang Y; Wang T; Fang H; Huang H; Fang L; Zhang X | Evaluation of Pharmacokinetics and Safety With Bioequivalence of Anastrozole in Healthy Chinese Volunteers: Bioequivalence Study Findings. | Clinical pharmacology in drug development | 10.1002/cpdd.1099 | E5 Not original research |
| 462 | Huang C; Dong D; Yu F; Ren X; Wu Y; Wang Z | Evaluation of Pharmacokinetics and Safety With Bioequivalence of Voriconazole Injection of 2 Formulations in Chinese Healthy Volunteers: Bioequivalenc | Clinical pharmacology in drug development | 10.1002/cpdd.1218 | E5 Not original research |
| 464 | Liu H; Xiong Y; Chen X; Yu H; Lan L; He W | Evaluation of pharmacokinetic and pharmacodynamic similarity of an IDegAsp biosimilar versus the originator in healthy Chinese volunteers. | Expert opinion on investigational drugs | 10.1080/13543784.2025.2463085 | E5 Not original research |
| 465 | Tan D; Zhang F; Fu X; Su J; Xu Z; Yang H | Evaluation of the Pharmacokinetics, Bioequivalence, and Safety of Two Preparations of 20-mg Trimetazidine in Healthy Chinese Subjects. | Clinical pharmacology in drug development | 10.1002/cpdd.1582 | E5 Not original research |
| 466 | Sreedevi A; Raj EA; Sreedhar D; Ligade VS | Exploring the challenges faced by generic version of complex drugs: a scoping review. | Systematic reviews | 10.1186/s13643-025-02931-y | E5 Not original research |
| 471 | Li X; Liang E; Hong X; Han X; Li C; Wang Y | In Vitro and In Vivo Bioequivalence Study of 3D-Printed Instant-Dissolving Levetiracetam Tablets and Subsequent Personalized Dosing for Chinese Childr | Pharmaceutics | 10.3390/pharmaceutics14010020 | E5 Not original research |
| 478 | Du P; Li P; Liu H; Zhao R; Zhao Z; Yu W | Open-Label, Randomized, Single-Dose, 2-Period, 2-Sequence Crossover, Comparative Pharmacokinetic Study to Evaluate Bioequivalence of 2 Oral Formulatio | Clinical pharmacology in drug development | 10.1002/cpdd.743 | E5 Not original research |
| 480 | Liu YJ; Sun LN; Cheng ZP; Qian Y; Ma ZQ; Zhang XH | Pharmacogenetic and safety analysis of cinacalcet hydrochloride in healthy Chinese subjects. | Annals of translational medicine | 10.21037/atm-20-1329 | E5 Not original research |
| 481 | He Y; Wang J; Yao F; Lu P; Xie Y; Li X | Pharmacokinetic Bioequivalence and Safety Assessment of Two Venlafaxine Hydrochloride Extended-Release Capsules in Healthy Chinese Subjects Under Fed | Drugs in R&D | 10.1007/s40268-024-00470-w | E5 Not original research |
| 483 | Shao R; Yang DD; Ruan ZR; Chen JL; Hu Y; Jiang B | Pharmacokinetic and Bioequivalence Evaluation of 2 Tadalafil Tablets in Healthy Male Chinese Subjects Under Fasting and Fed Conditions. | Clinical pharmacology in drug development | 10.1002/cpdd.1007 | E5 Not original research |
| 485 | Xu L; Su J; Muhetaer H; Zhang C; Huang C; Tang Y | Pharmacokinetic and Bioequivalence Evaluation of Two Cefprozil Dispersible Tablets in Healthy Chinese Volunteers. | Clinical pharmacology in drug development | 10.1002/cpdd.1304 | E5 Not original research |
| 489 | Shi P; Jiang X; Tao Y; Li T; Li X; Wang C | Pharmacokinetic and Safety Comparison of 2 Afatinib Dimaleate Tablets in Healthy Chinese Volunteers Under Fasted Conditions: A Randomized, Open-Label, | Clinical pharmacology in drug development | 10.1002/cpdd.1134 | E5 Not original research |
| 490 | Mei Y; Zuo X; Zhang T; Bu Z; Xu Z | Pharmacokinetic and bioequivalence of two formulations of Valsartan/Amlodipine Tablet(I) in healthy Chinese volunteers: A randomized, three-period, pa | Scientific reports | 10.1038/s41598-025-32576-9 | E5 Not original research |
| 495 | Wang L; Ruan Z; Yang D; Hu Y; Liang J; Chen J | Pharmacokinetics and Bioequivalence Evaluation of Erlotinib Hydrochloride Tablets: Randomized, Open-Label, 2-Period Crossover Study in Healthy Chinese | Clinical pharmacology in drug development | 10.1002/cpdd.811 | E5 Not original research |
| 497 | Li X; Yuan F; Xu B; Xiao G; Fan X; Li Y | Pharmacokinetics and Bioequivalence Studies of Roxatidine Acetate Hydrochloride Sustained-Release Capsule in Healthy Chinese Subjects. | Clinical pharmacology in drug development | 10.1002/cpdd.1053 | E5 Not original research |
| 498 | Xin L; Chen Q; Chen D; Li Y; Li Y; Wu Q | Pharmacokinetics and Bioequivalence of 2 Nifedipine Controlled-Release Tablets: A Randomized, Single-Dose, 2-Period Crossover Study in Healthy Chinese | Clinical pharmacology in drug development | 10.1002/cpdd.1258 | E5 Not original research |
| 499 | Ye L; Shen M; Chen Y; Li X; Xu M; Yao C | Pharmacokinetics and Bioequivalence of 2 Oral Formulations of Domperidone After Single-Dose Administration in Healthy Chinese Subjects. | Clinical pharmacology in drug development | 10.1002/cpdd.1097 | E5 Not original research |
| 500 | Tian M; Ye L; Liang B; Chen Y; Mei J; Zhao Z | Pharmacokinetics and Bioequivalence of 2 Oral Formulations of Vildagliptin in Healthy Chinese Subjects. | Clinical pharmacology in drug development | 10.1002/cpdd.1482 | E5 Not original research |
| 501 | Wu ZX; Wang CJ; Shi P; Liu YP; Li T; Sun FF | Pharmacokinetics and Bioequivalence of Abiraterone Acetate Tablets in Healthy Chinese Volunteers: An Open, Randomized, Single-Dose, Three-Period, Thre | Drugs in R&D | 10.1007/s40268-023-00418-6 | E5 Not original research |
| 502 | Tang L; Wang Y; Chen R; He Y; Liu Y; Wang N | Pharmacokinetics and Bioequivalence of Amlodipine Besylate Tablet in Healthy Chinese Volunteers Under Fasting and Fed Conditions. | Clinical pharmacology in drug development | 10.1002/cpdd.1345 | E5 Not original research |
| 503 | Bai W; Sun X; Qiu B; Guo C; Song H; Hu Y | Pharmacokinetics and Bioequivalence of Apremilast Tablets in Chinese Healthy Subjects Under Fasting and Postprandial States. | Drug design, development and therapy | 10.2147/DDDT.S461771 | E5 Not original research |
| 504 | Lin PP; Wang CJ; Liu YP; Li T; Gao XM; Ma YP | Pharmacokinetics and Bioequivalence of Cefprozil for Suspension and Granule Formulation in Healthy Chinese Volunteers: Two Single-Dose Crossover Studi | Advances in therapy | 10.1007/s12325-020-01593-7 | E5 Not original research |
| 506 | Chen N; He Q; Ma Y; Liu S; Wei H; Peng A | Pharmacokinetics and Bioequivalence of Fluconazole Capsules Manufactured in France and China in Healthy Chinese Participants: Open-Label, Randomized, | Clinical pharmacology in drug development | 10.1002/cpdd.1248 | E5 Not original research |
| 507 | Wang S; Wu F; Han Y; Ni S; Guo S; Dai Y | Pharmacokinetics and Bioequivalence of Misoprostol Tablets: An Open-Label, Randomized, Single-dose, Crossover Study With Healthy Chinese Volunteers. | Clinical pharmacology in drug development | 10.1002/cpdd.1102 | E5 Not original research |
| 509 | Li Y; Qi L; Bai H; Liu Y; Fan R; Tu Y | Pharmacokinetics and Bioequivalence of Rasagiline Tablets in Chinese Healthy Subjects Under Fasting and Fed Conditions: An Open, Randomized, Single-Do | Frontiers in pharmacology | 10.3389/fphar.2020.571747 | E5 Not original research |
| 510 | Rao X; Wu X; Hu J; Huang Z | Pharmacokinetics and Bioequivalence of Two Formulations of Montelukast Sodium Tablets in Healthy Chinese Volunteers Under Fasting and Fed Conditions. | Clinical pharmacology in drug development | 10.1002/cpdd.1485 | E5 Not original research |
| 511 | Wu Q; Wang X; Chen Q; Zou Y; Xu X; Li T | Pharmacokinetics and Bioequivalence of Two Formulations of Valsartan 80 mg Capsules: A Randomized, Single Dose, 4-Period Crossover Study in Healthy Ch | Drug design, development and therapy | 10.2147/DDDT.S253078 | E5 Not original research |
| 512 | Shao J; Liu X; Lin J; Chen J; Xie X | Pharmacokinetics and Bioequivalence of Two Powders of Azithromycin for Suspension: A Nonblinded, Single-Dose, Randomized, Three-Way Crossover Study in | Drugs in R&D | 10.1007/s40268-024-00492-4 | E5 Not original research |
| 514 | Lei Y; Luo P; Chen Y; Xue L; Zhang D; Qi X | Pharmacokinetics and Safety Evaluation of a New Generic Sitafloxacin: A Phase I Bioequivalence Study in Healthy Chinese Participants. | Clinical pharmacology in drug development | 10.1002/cpdd.1519 | E5 Not original research |
| 515 | Wang M; Wang M; Zhang Q; Zong S; Lv C | Pharmacokinetics and Safety of Levetiracetam Extended-Release Tablets and Relative Bioavailability Compared with Immediate-Release Tablets in Healthy | European journal of drug metabolism and pharmacokinetics | 10.1007/s13318-018-0461-2 | E5 Not original research |
| 516 | Chen H; Xu H; Yuan F; Li H; Sheng L; Liu C | Pharmacokinetics and Safety of Linezolid Tablets of 2 Different Manufacturers in Healthy Chinese Subjects in Fasting and Fed States. | Clinical pharmacology in drug development | 10.1002/cpdd.1462 | E5 Not original research |
| 518 | Wu B; Wang W; Zhang Q; Yu G; Lin J; Zhang T | Pharmacokinetics and Safety with Bioequivalence of Isosorbide Mononitrate Sustained-Release Tablets in Chinese Healthy Volunteers: Bioequivalence Stud | Drug design, development and therapy | 10.2147/DDDT.S508202 | E5 Not original research |
| 519 | Huang X; Wang Y; Li B; Shen X; Tao X; Zheng W | Pharmacokinetics and bioequivalence assessment of two prucalopride formulations in healthy Chinese women: a randomized, open-label, two-period, two-se | Frontiers in pharmacology | 10.3389/fphar.2025.1562692 | E5 Not original research |
| 525 | Lu J; Lei Y; Mo Y; Wang X; Liu W; Yan Y | Pharmacokinetics and bioequivalence of two formulations of the S-1 (tegafur/gimeracil/oxonate) capsule in Chinese cancer patients under fasting and fe | Frontiers in pharmacology | 10.3389/fphar.2025.1494902 | E5 Not original research |
| 526 | Han M; He Y; Liang J; Yao F; Lu P; Yan H | Pharmacokinetics and bioequivalence study of candesartan cilexetil tablet in Chinese volunteers under fasting condition: an open-label, randomized-seq | Translational and clinical pharmacology | 10.12793/tcp.2024.32.e10 | E5 Not original research |
| 527 | Ding Y; Chu N; Que L; Huang K; Chen Y; Qin W | Pharmacokinetics and bioequivalence study of esomeprazole magnesium enteric-coated tablets 20 mg in healthy Chinese subjects under fasting and fed con | Frontiers in pharmacology | 10.3389/fphar.2023.1169103 | E5 Not original research |
| 528 | Zhu G; Wang L; Han S; Peng H; Tong M; Gu X | Pharmacokinetics and bioequivalence study of montelukast sodium oral soluble film and chewable tablet in healthy Chinese volunteers: randomized trials | Annals of translational medicine | 10.21037/atm-22-6485 | E5 Not original research |
| 530 | Qiu B; Song H; Ding C; Sun X; Du R; Yang H | Pharmacokinetics and safety of highly variable valsartan in single-pill combination with amlodipine versus its generic formulation: a randomized, thre | Frontiers in pharmacology | 10.3389/fphar.2023.1264321 | E5 Not original research |
| 533 | Yu J; Wang Y; Wu Y; Lin S; Hao R; Fang L | Pharmacokinetics of Meloxicam Tablets in Healthy Chinese Adults in the Fasting and Fed States: A Single-Site, Single-Dose, Randomized, Open, 2-Period, | Clinical pharmacology in drug development | 10.1002/cpdd.965 | E5 Not original research |
| 535 | Zhao D; Ni J; Zhang W; Zhou Y; Chang D; Yang Z | Pharmacokinetics, Bioequivalence, and Safety Evaluation of Dienogest in Healthy Subjects Under Fasting and Fed Conditions. | Clinical pharmacology in drug development | 10.1002/cpdd.1215 | E5 Not original research |
| 536 | Wang Y; Liu Y; Tang L; Gao J; Li H; Dai X | Pharmacokinetics, Bioequivalence, and Safety Evaluation of Two Oral Formulations of Calcium Dobesilate Capsules in Healthy Chinese Volunteers Under Fa | Clinical pharmacology in drug development | 10.1002/cpdd.1376 | E5 Not original research |
| 538 | Zhou Z; Wang C; Zheng X; Yu X; Yu C; Zhang D | Pharmacokinetics, Bioequivalence, and Safety Studies of Prucalopride in Healthy Chinese Subjects. | Clinical pharmacology in drug development | 10.1002/cpdd.754 | E5 Not original research |
| 539 | Luo HY; Long HZ; Zhou ZW; Xu SG; Li FJ; Cheng Y | Pharmacokinetics, Bioequivalence, and Safety of 2 Formulations of Hydroxychloroquine Tablets in Healthy Chinese Volunteers Under Fasting and Fed Condi | Clinical pharmacology in drug development | 10.1002/cpdd.1166 | E5 Not original research |
| 541 | Jin J; Huang C; Zhu C; Feng W; He A; Li T | Pharmacokinetics, Bioequivalence, and Safety of Esomeprazole Magnesium Enteric-Coated Capsules in Healthy Chinese Subjects. | Clinical pharmacology in drug development | 10.1002/cpdd.1273 | E5 Not original research |
| 569 | Yu J; Wu Y; Lin S; Wang Y | Safety, Tolerability, and Pharmacokinetics of Voriconazole for Injection in Two Preparations in Chinese Healthy Adult Volunteers. | Clinical pharmacology in drug development | 10.1002/cpdd.1370 | E5 Not original research |
| 572 | Zeng Z; Zhong L; Zhu L; Liao L; Zhu Z; Cao Y | Subcutaneous versus intravenous trastuzumab for HER2-positive breast cancer: a global systematic review and meta-analysis with a cost-minimization ana | Frontiers in pharmacology | 10.3389/fphar.2025.1730175 | E5 Not original research |
| 576 | Zhang W; Zhang Y; Tang J; Wang X; Meng C; Wu J | The Changing Landscape of Heart Failure Drug Clinical Trials in China, 2013-2023. | Drug design, development and therapy | 10.2147/DDDT.S511608 | E5 Not original research |
| 579 | Zhao N; Jia C; Hu Y; Sun X; Song H; Qiu B | The effect of high-fat diet on the pharmacokinetics of ondansetron hydrochloride tablets in healthy Chinese subjects. | Frontiers in pharmacology | 10.3389/fphar.2025.1512857 | E5 Not original research |
| 583 | Liu T; Liu D; Jin Y; Dong M | Trastuzumab biosimilars vs trastuzumab originator in the treatment of HER2-positive breast cancer: a systematic review and network meta-analysis. | Immunopharmacology and immunotoxicology | 10.1080/08923973.2022.2090956 | E5 Not original research |
| 590 | Yu HL; Liu H; Li JQ; Tan HW; Yu YR | [The Pharmacokinetics, Pharmacodynamics and Bioequivalence of Insulin Aspart Produced by the United Laboratories Evaluated by Euglycemic Clamp Study]. | Sichuan da xue xue bao. Yi xue ban = Journal of Sichuan Univ | 10.12182/20200560607 | E5 Not original research |
| 592 |  | 《中国医疗保险》杂志近期征稿选题 | 中国医疗保险 |  | E5 Not original research |
| 609 | 蒋力群; 黄琴 | 你能看懂卡培他滨的说明书吗 | 漫科学 |  | E5 Not original research |
| 612 | 蔡德山[] | 阿托伐他汀钙片-“阿乐”重生历程 | 中国制药信息 |  | E5 Not original research |
| 620 | 朱刚令; 王朝永 | AI如何助力医药集采高质量发展?——基于我国AI赋能医药集采的现状与思考 | 中国医疗保险 | 10.19546/j.issn.1674-3830.2025.10.005 | E5 Not original research |
| 631 | 白依鹭 | BSD医疗器械公司辽宁市场营销策略研究 |  |  | E5 Not original research |
| 634 | 张健 | B公司输液器生产现场管理改进研究 |  |  | E5 Not original research |
| 658 | 张慧 | G公司采购审核管理业务流程优化研究 |  |  | E5 Not original research |
| 684 | 文林睿 | TZ医院医药采购内部监督研究 |  |  | E5 Not original research |
| 695 | 宁艳阳 | “4+7”药品带量采购面对的三个问题 | 中国卫生 | 10.15973/j.cnki.cn11-3708/d.2019.09.008 | E5 Not original research |
| 706 | 张雪强; 潘梦超 | ”高价药”的成因及其治理策略研究 ——基于德、日药价管理经验 | 价值工程 |  | E5 Not original research |
| 707 | 王妍; 武志昂 | 《国家组织药品集中采购和使用试点工作监测方案》要点分析 | 中国药房 | 10.6039/j.issn.1001-0408.2019.17.03 | E5 Not original research |
| 710 | 史玉清; 刘长青; 冯雪莹; 徐梅; 杨建卫; 赵玉娟 | 三医协同视角下的医疗器械不良事件监测工作实践与展望 | 医疗装备 | 10.3969/j.issn.1002-2376.2025.10.007 | E5 Not original research |
| 726 | 谷伟军 | 中国基层医疗机构基础胰岛素的使用现状和选择的新思路 | 药品评价 | 10.19939/j.cnki.1672-2809.2024.01.01 | E5 Not original research |
| 727 | 郑毅 | 中国大输液的发展趋势和包装的变革 | 基层医学论坛（新加坡学术出版社） | 10.12373/jcyxlt.2024.12.16673 | E5 Not original research |
| 729 | 戴伟 | 中成药集采要以质量为重 | 中国社会保障 | 10.3969/j.issn.1008-4304.2022.01.024 | E5 Not original research |
| 731 | 庞婕 | 中标对比原研莫西沙星片治疗门诊社区获得性肺炎患者临床疗效与安全性评价 | 2022中国临床药学学术年会暨第十八届中国临床药师论坛 |  | E5 Not original research |
| 733 | 张燊 | 中药配方颗粒集中带量采购政策分析与思考 | 药学研究 | 10.13506/j.cnki.jpr.2025.02.009 | E5 Not original research |
| 735 | 代雪飞; 杜娟 | 临床药师视角下甘精胰岛素U100转换应用甘精胰岛素U300致血糖波动1例 | 实用药物与临床 | 10.14053/j.cnki.ppcr.202308012 | E5 Not original research |
| 754 |  | 仿制药一致性评价、带量采购打开新局面 | 医师在线 |  | E5 Not original research |
| 755 | 药娃 | 仿制药一致性评价问答(一) | 药物与人 |  | E5 Not original research |
| 756 | 药娃 | 仿制药一致性评价问答(三) | 药物与人 |  | E5 Not original research |
| 757 | 药娃 | 仿制药一致性评价问答(二) | 药物与人 |  | E5 Not original research |
| 758 | 药娃 | 仿制药一致性评价问答(四) | 药物与人 |  | E5 Not original research |
| 762 | 罗俊; 韦坤璇; 黄天敏; 戚志伟; 刘国萍; 陈依雨 | 伏立康唑原研与仿制品种的安全性、有效性和经济性评价研究 | 2022年中国药学大会 |  | E5 Not original research |
| 765 | 郑青敏 | 你了解仿制药一致性评价吗 | 药物与人 |  | E5 Not original research |
| 770 | 李成刚; 黎浩; 关翘楚 | 健康中国战略背景下，对仿制药一致性评价的思考 | 大健康 |  | E5 Not original research |
| 775 | 马志祥 | 公共采购政策对企业创新的影响研究--基于中国医药行业的实证分析 |  |  | E5 Not original research |
| 776 | 吴丽华; 缪家清; 杜思唯; 谭嘉文; 杨治论; 余冬兰 | 公立医院医用耗材带量集采管理问题与对策研究 | 现代医院 | 10.3969/j.issn.1671-332X.2024.05.027 | E5 Not original research |
| 780 | 任磊; 谢金平; 杨伊凡; 邵蓉 | 典型国家和地区原研药医保支付标准设计对我国的启示 | 中国卫生政策研究 | 10.3969/j.issn.1674-2982.2024.08.009 | E5 Not original research |
| 804 | 陈鹏 | 医疗器械带量采购下M公司在华战略研究 |  |  | E5 Not original research |
| 805 | 刘三霞; 王昊 | 医疗实践中的”技术中心”与”患者中心”——种植牙技术应用的辩证思考 | 医学与哲学 | 10.12014/j.issn.1002-0772.2025.23.12 | E5 Not original research |
| 814 | 苏会璇; 饶苑平; 陈英 | 医院医保网格化管理的实践与思考 | 保险职业学院学报 | 10.3969/j.issn.1673-1360.2025.02.008 | E5 Not original research |
| 825 | 周思茹; 丘岳; 申伟培; 黄振光; 陈晓宇; 张宏亮 | 口服二氢吡啶类钙通道阻滞剂治疗心血管疾病的快速综合评价广西专家共识 | 中国药房 | 10.6039/j.issn.1001-0408.2025.07.01 | E5 Not original research |
| 827 | 刘晓夫 | 合同物流企业运力采购流程优化设计--以HL物流为例 |  |  | E5 Not original research |
| 832 | 范成君 | 四川省高值医用耗材集中采购系统的设计与实现 |  |  | E5 Not original research |
| 838 | 潘文娟; 沈怡雯 | 国家及省级药品集中带量采购分析 | 现代医药卫生 | 10.3969/j.issn.1009-5519.2025.06.033 | E5 Not original research |
| 1226 | 王竹章 | 角膜塑形镜儿童近视控制效果及KAP现状调查分析 |  |  | E5 Not original research |
| 1410 | Zhu, Z; Wang, Q; Sun, Q; Lexchin, J; Yang, L | Improving access to medicines and beyond: the national volume-based procurement policy in China | BMJ GLOBAL HEALTH | 10.1136/bmjgh-2022-011535 | E5 Not original research |
| 1425 | Wu, F; Zhao, XL; Guo, SJ; Ni, SY; Dai, YY; Han, Y | A Bioequivalence Study With Pharmacokinetic Endpoints for Azithromycin Eye Drops | CLINICAL PHARMACOLOGY IN DRUG DEVELOPMENT | 10.1002/cpdd.1226 | E5 Not original research |
| 1428 | Storm, NE; Chang, W; Lin, TC; Lange, JL; Bradbury, B; Critch | A Novel Case Study of the Use of Real-World Evidence to Support the Registration of an Osteoporosis Product in China | THERAPEUTIC INNOVATION & REGULATORY SCIENCE | 10.1007/s43441-021-00342-4 | E5 Not original research |
| 1466 | Wu, J; Fan, YR; Zheng, L; Wang, ZQ; Liu, YY; Shen, CL | An Open-Label, Randomized Pharmacokinetic Study of 2 Formulations of Eldecalcitol Capsules Following Single Oral Administration in Healthy Chinese Vol | CLINICAL PHARMACOLOGY IN DRUG DEVELOPMENT | 10.1002/cpdd.1144 | E5 Not original research |
| 1467 | Lai, XP; Ye, SW; Chen, LH; Chen, JY; Zhang, N; Zhang, YW | An Open-Label, Randomized, 2-Way, Crossover Bioequivalence Study of Cefradine Capsules in Healthy Chinese Volunteers | CLINICAL PHARMACOLOGY IN DRUG DEVELOPMENT | 10.1002/cpdd.991 | E5 Not original research |
| 1709 | Ma, HP; Zhao, NP; Luo, SX; Zhang, YQ; Yuan, ZJ; Sun, H | Pharmacokinetic and bioequivalence study of two capecitabine tablets in Chinese patients with breast, colorectal or gastric cancer under fed condition | EUROPEAN JOURNAL OF PHARMACEUTICAL SCIENCES | 10.1016/j.ejps.2023.106373 | E5 Not original research |
| 70 | 岳小林; 付娜; 赵艳玲; 封宇飞; 果伟; 林阳 | 国家集中带量采购中选药品疗效与安全性的真实世界研究 | 临床药物治疗杂志 | 10.3969/j.issn.1672-3384.2022.06.008 | E6 Duplicate report of an included study |
| 74 | 国家组织药品集中采购中选仿制药疗效与安全性真实世界研究课题组 | 国家集采仿制药疗效与安全性评价真实世界研究报告——以第二、三批集采为例 | 中国医疗保险 | 10.19546/j.issn.1674-3830.2023.10.001 | E6 Duplicate report of an included study |
| 79 | 孙静雯; 林添; 蔡鸿福; 林碧娟; 刘茂柏; 曾晓芳 | 国家集采药品头孢呋辛在妇产科预防使用的真实世界研究 | 海峡药学 | 10.27019/j.cnki.1006-3765.2025.11.020 | E6 Duplicate report of an included study |
| 87 | 程瑶瑶 | 基于多中心真实世界数据的集采与非集采注射用头孢他啶的临床综合评价 |  | 10.27202/d.cnki.gkmyc.2024.001358 | E6 Duplicate report of an included study |
| 88 | 董艳珍 | 基于多中心真实世界数据的集采与非集采注射用头孢曲松钠临床综合评价 |  | 10.27811/d.cnki.gdixy.2023.000569 | E6 Duplicate report of an included study |
| 221 | 赵强; 石秀锦; 张翼; 林阳 | 仿制和原研氯吡格雷在冠状动脉介入术后有效性、安全性和经济性的对比研究 | 中国药物应用与监测 | 10.3969/j.issn.1672-8157.2021.02.002 | E6 Duplicate report of an included study |
| 227 | 廖荣琼; 林晓静; 李向龙; 倪勋; 罗文丰; 赵攀 | 国产仿制硼替佐米治疗多发性骨髓瘤的近期疗效及安全性观察 | 中国实验血液学杂志 | 10.19746/j.cnki.issn1009-2137.2021.01.022 | E6 Duplicate report of an included study |
| 256 | 邢晓璇; 张晓彤; 王可; 王之舟; 冯英楠; 李晓曦 | 阿托伐他汀仿制药与原研药LDL-C达标率和安全性评价的多中心回顾性队列研究 | 中国医院用药评价与分析 | 10.14009/j.issn.1672-2124.2025.09.007 | E6 Duplicate report of an included study |
| 311 | Zhao B; Wu J; Feng XL | Testing the unintended cost effects of health policies for generic substitutions: the case of China's National Volume-Based Procurement (NVBP) policy. | Health policy and planning | 10.1093/heapol/czae101 | E6 Duplicate report of an included study |
| 598 | 吴琪 | 带量采购中仿制药阿奇霉素治疗肺炎支原体肺炎的有效性与安全性分析 | 常州实用医学 |  | E7 Full text not obtainable |
| 295 | Sun X; Qian D; Zhao W; Hu X; Zhang Y | Lifecycle regulation of narrow therapeutic index drugs: Chinese practices, international benchmarks, and optimization pathways. | Naunyn-Schmiedeberg's archives of pharmacology | 10.1007/s00210-026-04996-5 | E8 Published outside the search window |
| 419 | Dean L; Kane M | Clopidogrel Therapy and CYP2C19 Genotype. |  |  | E8 Published outside the search window |
| 522 | Wang J; Zhang W | Pharmacokinetics and bioequivalence of acetylcysteine granules among Chinese healthy volunteers under fasting and postprandial conditions. | Clinical pharmacology in drug development | 10.1002/cpdd.1605 | E8 Published outside the search window |
| 523 | Yin X; Xia R; Song H; Zhang Y; Guo D; Gan F | Pharmacokinetics and bioequivalence of two N-acetylcysteine tablets in healthy Chinese volunteers under fasting and fed conditions. | Clinical pharmacology in drug development | 10.1002/cpdd.1619 | E8 Published outside the search window |
| 594 | 刘珊珊; 李婕; 王斌; 任艳萍 | 国家组织药品集中采购政策下奥氮平片中标药与原研药的真实世界研究 | 中国现代医药杂志 | 10.3969/j.issn.1672-9463.2026.03.003 | E8 Published outside the search window |
| 595 | 刘慧慧; 袁明辉; 荣凌燕; 高文政; 杨少辉; 许红霞 | 国家集中采购下依诺肝素钠注射液的疗效与安全性研究 | 中南医学 |  | E8 Published outside the search window |
| 711 | 刘书蒙; 龙 桓; 夏静雅; 张 芹 | 三唑类抗真菌药预防真菌感染肺炎的疗效与安全性分析 | 临床医学前沿 | 10.37155/2661-4766-0802-18 | E8 Published outside the search window |
| 748 | 赵福坤; 罗敏; 王远敏; 李美燕; 苏穆; 杨俊 | 仿制与原研利伐沙班对非瓣膜性房颤抗凝治疗的临床效应对比研究 | 遵义医科大学学报 | 10.3969/j.issn.2096-8159.2026.02.009 | E8 Published outside the search window |
| 763 | 裴彤; 王之舟; 董宪喆; 张兰 | 伏立康唑片仿制药与原研药疗效和安全性的倾向性评分匹配分析 | 中国医院用药评价与分析 | 10.14009/j.issn.1672-2124.2026.03.007 | E8 Published outside the search window |
| 1405 | Katikireddy, KR; Schmedt, T; Price, MO; Price, FW; Jurkunas, | Existence of Neural Crest-Derived Progenitor Cells in Normal and Fuchs Endothelial Dystrophy Corneal Endothelium | AMERICAN JOURNAL OF PATHOLOGY | 10.1016/j.ajpath.2016.06.011 | E8 Published outside the search window |
| 1411 | Tian, S; Feng, L; Sun, Z; Miao, RS; Zhang, YZ; Jiang, P | Optimizing antibiotic prophylaxis for type III open fractures in China: a cost-effectiveness analysis | HEALTH AND QUALITY OF LIFE OUTCOMES | 10.1186/s12955-026-02517-9 | E8 Published outside the search window |
| 1413 | Waits, S; Sheetz, K; Kanitz, W; Englesbe, M | Quantifying Improvement in Donor Travel Safety: Utilization of Centralized Procurement Facilities and outside Surgeon Organ Procurement. | AMERICAN JOURNAL OF TRANSPLANTATION |  | E8 Published outside the search window |
| 1418 | Atiku, S; Okoh, A; Olakotan, O | The Contingent Payoff of Transparency: The Mediating Roles of Citizen Participation and Enforcement in Health Service Delivery Improvement | JOURNAL OF EVALUATION IN CLINICAL PRACTICE | 10.1111/jep.70401 | E8 Published outside the search window |
| 1421 | Chen, K; Luo, Q; Li, YF; Zhong, Y; Gao, B | The impact of centralized volume-based procurement policy on dental implant procurement volume in medical institutions of a Western Province in China- | BMC PUBLIC HEALTH | 10.1186/s12889-026-26451-z | E8 Published outside the search window |
| 1442 | Zeng, J; Qi, YR; Wang, L; Wang, Z; Zhang, LW; Yan, H | A multidimensional fingerprint-based quality evaluation strategy integrating chromatographic and spectroscopic fingerprints: a case study of Rhus typh | NEW JOURNAL OF CHEMISTRY | 10.1039/d6nj01401a | E8 Published outside the search window |
| 1470 | Xu, JF; Luo, YY; Sun, D; Yu, YX; Zhou, J; Yang, J | BIOEQUIVALENCE AND EFFICACY STUDY OF MB-102 WITH TRANSDERMAL GFR MEASUREMENT IN CHINESE SUBJECTS | CRITICAL CARE MEDICINE | 10.1097/01.ccm.0001185120.87027.5d | E8 Published outside the search window |
| 1475 | Chen, J; Jiang, B; Lou, H; Yu, L; Ruan, Z | Bioequivalence Evaluation of Cefdinir in Healthy Fasting Subjects | ARZNEIMITTEL-FORSCHUNG-DRUG RESEARCH | 10.1055/s-0031-1291361 | E8 Published outside the search window |
| 1478 | Chen, J; Jiang, B; Lou, H; Yu, L; Ruan, Z | Bioequivalence Studies of 2 Oral Cefaclor Capsule Formulations in Chinese Healthy Subjects | ARZNEIMITTELFORSCHUNG-DRUG RESEARCH | 10.1055/s-0031-1298012 | E8 Published outside the search window |
| 1488 | Ying, YY; Huang, CQ; Xu, L; Wu, Y; Cai, SF; Ye, LJ | Bioequivalence Study of Two Ibuprofen Arginine Formulations in Healthy Chinese Subjects Under Fed Conditions | CLINICAL PHARMACOLOGY IN DRUG DEVELOPMENT | 10.1002/cpdd.70083 | E8 Published outside the search window |
| 1492 | Zhang, Q; Tao, YF; Zhu, YB; Zhu, DC | Bioequivalence and Pharmacokinetic Comparison of Two Mycophenolate Mofetil Formulations in Healthy Chinese Male Volunteers: An Open-Label, Randomized- | CLINICAL THERAPEUTICS | 10.1016/j.clinthera.2010.01.013 | E8 Published outside the search window |
| 1493 | Zou, JJ; Ji, HJ; Wu, DW; Yao, J; Hu, Q; Xiao, DW | Bioequivalence and Pharmacokinetic Comparison of a Single 200-mg Dose of Meclofenoxate Hydrochloride Capsule and Tablet Formulations in Healthy Chines | CLINICAL THERAPEUTICS | 10.1016/j.clinthera.2008.09.013 | E8 Published outside the search window |
| 1494 | Fu, KJ; Lu, CC; Zhang, YP; Pan, H; Wang, ZY; Chen, XJ | Bioequivalence and Pharmacokinetic Evaluation of Gliclazide Modified-Release Tablets in Healthy Chinese Volunteers | CLINICAL PHARMACOLOGY IN DRUG DEVELOPMENT | 10.1002/cpdd.70021 | E8 Published outside the search window |
| 1497 | Li, CY; Xu, J; Zheng, Y; Chen, GL; Wang, JM; Ma, LY | Bioequivalence and Pharmacokinetic Profiles of Agomelatine 25-mg Tablets in Healthy Chinese Subjects: A Four-Way Replicate Crossover Study Demonstrati | CHEMICAL & PHARMACEUTICAL BULLETIN | 10.1248/cpb.c16-00866 | E8 Published outside the search window |
| 1691 | Zhou, HL; Wu, GL; Hu, XJ; Zhu, MX; Zhai, Y; Liu, J | Pharmacokinetic Properties and Tolerability of Cycloserine Following Oral Administration in Healthy Chinese Volunteers: A Randomized, Open-Label, Sing | CLINICAL THERAPEUTICS | 10.1016/j.clinthera.2015.03.015 | E8 Published outside the search window |

Totals: included 81; E1 226; E2 16; E3 68; E4 174; E5 263; E6 9; E7 1; E8 24. No report remained awaiting classification.
